# A combined molecular and morphological phylogeny of the Loricariinae (Siluriformes: Loricariidae), with emphasis on the Harttiini and Farlowellini

**DOI:** 10.1371/journal.pone.0247747

**Published:** 2021-03-15

**Authors:** Alejandro Londoño-Burbano, Roberto E. Reis

**Affiliations:** 1 Museu Nacional, Universidade Federal do Rio de Janeiro, Departamento de Vertebrados - Setor de Ictiologia, São Cristóvão, Rio de Janeiro, RJ, Brazil; 2 Pontifícia Universidade Católica do Rio Grande do Sul, PUCRS, Laboratório de Sistemática de Vertebrados, Porto Alegre, RS, Brazil; Universidad de los Andes, COLOMBIA

## Abstract

We present a combined molecular and morphological phylogenetic analysis of the Loricariinae, with emphasis on the Harttiini (*Cteniloricaria*, *Harttia*, and *Harttiella*) and Farlowellini (*Aposturisoma*, *Farlowella*, *Lamontichthys*, *Pterosturisoma*, *Sturisoma*, and *Sturisomatichthys*). Character sampling comprised seven molecular markers (the mitochondrial Cytb, nd2, 12S and 16S, and the nuclear MyH6, RAG1 and RAG2) and 196 morphological characters. A total of 1,059 specimens, and 159 tissue samples were analized, representing 100 species. A Bayesian Inference analysis was performed using the concatenated data matrix, which is comprised of 6,819 characters. The Loricariinae were found to comprise the tribes (Hartiini (Loricariini, Farlowellini)), the latter two elevated from subtribes. A Maximum Parsimony analysis was also performed using the same data matrix in order to reveal phenotypical synapomorphies to diagnose each clade. Two MP trees were found with a length of 14,704 steps, consistency index of 0.29 and retention index of 0.61, which were summarized in a strict consensus tree. Harttiini includes (*Harttiella* (*Cteniloricaria*, *Harttia*), and Farlowellini includes (*Lamontichthys* (*Pterosturisoma* (*Sturisoma* (*Sturisomatichthys*, *Farlowella*)))). *Aposturisoma* was recovered nested within *Farlowella* and is synonymyzed to the latter. *Sturisoma* was corroborated as strictly cis-Andean, while *Sturisomatichthys* encompasses, besides the valid species already included in the genus, the trans-Andean species once belonging to *Sturisoma sensu lato*. Identification keys and phylogenetic diagnoses of family-group taxa and genera of both the Harttiini and the Farlowellini are provided.

## Introduction

### Siluriformes and Loricariidae diversity

Neotropical fishes comprise one of the most diverse and speciose faunas of vertebrates on earth. According to Fricke et al. [1], there are currently 35,768 valid species of fishes. In the Neotropical region, there are more than 5,600 species of fishes [2], with more recent estimates reaching to 9,000 species [3], which represents around 13% of all known vertebrates [4–6].

The order Siluriformes includes representatives in freshwater on all continents except Antarctica, plus two marine families. Currently the order has 39 valid families [1], 19 of which are found in South America [7–9]. One of the main clades of the order Siluriformes is the family Loricariidae, with over 1,000 valid species [1]; it currently has six subfamilies: Lithogeninae, Delturinae, Rhinelepinae, Loricariinae, Hypoptopomatinae, and Hypostominae [10]. Several phylogenetic and taxonomic studies have dealt with the Loricariidae to elucidate their phylogenetic relationships, both interspecific and intergeneric, using either molecular or morphological evidence [10–16]. However, there are still inconsistencies in the number of subfamilies, diagnostic characters for each of its subgroups, and their composition.

### Systematic history of Harttiini and Farlowellini

Loricariinae currently includes 255 valid species in 31 genera [1], distributed from the La Plata basin in Argentina to Costa Rica [17]. The species in this subfamily are characterized by a long and depressed caudal peduncle and the absence of an adipose fin. They usually are benthic species and show marked variations in body shape that correspond to the different environments they inhabit: lotic to lentic systems, organic or inorganic substrates such as rocks, logs, or soft matter [18]. Regan [19] produced the first monograph dealing with the Loricariidae and included *Farlowella*, *Hemiodontichthys*, *Loricaria*, and *Oxyloricaria* in the Loricariinae. At that time, most of the species included in Loricariinae were described as *Loricaria*. The main taxonomic contribution of Regan [19] regarding Loricariinae was the diagnosis of *Oxyloricaria* (= *Sturisoma*), comparing it to *Loricaria*, and the description of new species in that genus. Nevertheless, shortly after, Eigenmann [20] proposed *Oxyloricaria* as a junior synonym of *Sturisoma*, and transferred most *Oxyloricaria* species to the latter. Regan [21] offered a second study regarding Loricariidae, but his results did not differ significantly from his first work and maintained the same classification for the family.

Gosline [22] based his study mainly on osteological characters to diagnose the different subfamilies he recognized. The author included within Loricariidae the Astroblepinae (= Astroblepidae), Lithogeninae, Neoplecostominae, Plecostominae, Hypoptopomatinae, and Loricariinae. Nevertheless, the author focused his study mainly on the Neoplecostominae and Plecostominae, and did not offer an identification key for the genera of Loricariinae, or a classification for the subfamily.

Boeseman [23] offered a study dealing strictly with the Loricariinae from Suriname. The author mostly followed the conclusions offered by Gosline [22], in that Loricariinae shows some indication of being biphyletic, and could be split into two apparently distinct groups based on gill raker structure, pharyngeal tooth development, number of oral teeth, and possibly the presence or absence of a postorbital notch. Boeseman [23] suggested that in some characters the scarce-toothed forms present a much wider range of variation within the group, comparable to that found in the whole series of the comb-toothed genera.

Shortly after, Boeseman [24] published another study dealing with the Loricariinae from Suriname in which redescriptions of *Loricaria cataphracta*, *Loricaria maculata* (= *Loricariichthys maculatus*), and *Loricaria* cf. *stewarti* (= *Rineloricaria stewarti*) were offered, as well as the description of *Harttia nijsseni* (= *Metaloricaria nijsseni*). The author maintained his hypothesis of the Harttiinae and Loricariinae as different and separate lineages.

Subsequently, Isbrücker [25] divided Loricariinae into four tribes: Acestridiini, Farlowellini, Harttiini, and Loricariini. The author did not follow Boeseman [23, 24] in assuming Harttiinae as valid, and maintained Loricariinae as including the Harttiini, which in turn was divided in Harttiina, and Metaloricariina, the Farlowellini, and the Acestridiini, which was comprised only of *Acestridium*, later transferred to the Hypoptopomatinae [26, 27]. In addition, Loricariini was divided into Rineloricariina, Planiloricariina, Reganeliina, Loricariichthyina, and Hemiodontichthyina.

Rapp Py-Daniel [28] presented the first morphology-based phylogenetic analysis aiming to include most of the diversity of the Loricariinae, with 192 characters from both osteology and external morphology. The author found the subfamily to be divided into two tribes, the Harttiini and the Loricariini. The former included *Aposturisoma*, *Cteniloricaria* (assumed as synonym of *Harttia*), *Farlowella*, *Harttia*, *Lamontichthys*, *Sturisoma*, and *Sturisomatichthys*; *Harttiella* and *Pterosturisoma* were not available for examination. Thus, Rapp Py-Daniel [28] split the tribe into two subtribes: the Harttiina and the Farlowellina, the former comprising *Harttia* and *Lamontichthys*, with the remaining genera in the latter. Even though the author offered no diagnosis or identification keys for the genera included, her study produced several diagnostic morphological characters to support that classification.

The most recent study regarding the Loricariinae is that of Covain et al. [15], who performed a molecular phylogenetic analysis of the subfamily, where almost all genera and most species were included. The authors used three markers, two mitochondrial (12S and 16S) and one nuclear (f-rtn4), and 350 terminal taxa. The authors divided Loricariinae into two sister tribes: Harttiini and Loricariini. The Harttiini was found to comprise *Cteniloricaria*, *Harttia*, and *Harttiella*. Within Loricariini, the authors proposed two subtribes, Farlowellina and Loricariina. Within Farlowellina the authors included *Aposturisoma* (as a possible synonym of *Farlowella*), *Farlowella*, *Lamontichthys*, *Pterosturisoma*, *Sturisoma* (restricted to cis-Andean species), and *Sturisomatichthys* (including all trans-Andean *Sturisoma sensu lato*). Within Loricariina, the authors included *Dasyloricaria*, *Fonchiiloricaria*, and *Metaloricaria*, in addition to three different clades, the *Rineloricaria* group, the *Loricariichthys* group, and the *Loricaria*-*Pseudohemiodon* group (for configuration of each group see Covain et al. [15]). Covain et al. [15] also discussed the inconsistencies of the morphological and the molecular classifications so far produced for the Loricariinae.

Notwithstanding the studies mentioned above, the taxonomy of the Harttiini and Farlowellina has not been settled, and a detailed study of interspecific relationships and phylogenetic diagnoses of its genera is needed. Moreover, the use of only morphological or molecular evidence could be one of the causes of the disparity regarding the classifications within Loricariinae. Thus, the objective of the present study is to perform a total evidence analysis of the Loricariinae, with emphasis on Harttiini and Farlowellina, including the available morphological and molecular information to clarify their intraspecific and intergeneric relationships and to provide phylogenetic diagnoses for each genus and family-group taxa.

## Material and methods

### Phylogenetic inference method

The phylogenetic method used for the discovery of interrelationships and proposal of classification of the Loricariinae is the Bayesian Inference (BI) analysis. Nevertheless, a Maximum Parsimony (MP) analysis, following the argumentation of Kluge and Grant [29] was performed in order to reveal phenotypical synapomorphies to diagnose each clade and its results are discussed and compared to those obtained through BI. The analyses follow a Total Evidence approach (as proposed by Kluge [30]), which relies on its ability to implement the scientific principle of severity of test, and discussed by Fitzhugh [31], who demonstrates the logical requirement of total evidence analysis.

We used the tree obtained by the BI analysis to propose a classification for the Loricariinae, because our data matrix has nucleotide substitution rates highly uneven between data partitions, and because several substitution models are employed among markers, when compared to the single model used for the morphological partition. Such factors can easily account for reconstruction artifact, such as long branch attraction [32–35], making MP a less robust approach in this case [32]. MP is effective when rates of change are rather low and/or constant between character states (eg. morphological characters only, or single substitution models between data); otherwise, MP would involve stringent assumptions thus becoming invalid. The MP analysis was used as a source of morphological synapomorphies to diagnose genera and family-group taxa, while relaying on the methodologically stronger Bayesian topology for classification. In order to reveal synapomorphies for the two clades not common to MP and BI analyses (*Metaloricaria* as sister to remaining Loricariini and *Harttiella* as sister to remaining Harttiini), a MP analysis constrained to the BI topology was performed.

### Ingroup selection

The ingroup selected aimed to contain all genera historically included in Harttiini and Farlowellina: *Aposturisoma*, *Cteniloricaria*, *Farlowella*, *Harttia*, *Harttiella*, *Lamontichthys*, *Metaloricaria*, *Pterosturisoma*, *Sturisoma* and *Sturisomatichthys*. The present study includes 78 of the 96 valid species (81%) of these 10 genera [1]. In parentheses we give the number of species-terminals included in this study followed by the number of valid species in that genus: *Aposturisoma* (1/1), *Cteniloricaria* (2/2), *Farlowella* (23/31), *Harttia* (19/27), *Harttiella* (5/7), *Lamontichthys* (5/7), *Metaloricaria* (2/2), *Pterosturisoma* (1/1), *Sturisoma* (9/10) and *Sturisomatichthys* (11/13). In addition, 13 species of Loricariina were included: *Dasyloricaria filamentosa*, *D*. *latiura*, *D*. *paucisquama*, *Hemiodontichthys acipenserinus*, *Limatulichthys griseus*, *Loricaria lundbergi*, *Loricariichthys anus*, *L*. *platymetopon*, *Pseudohemiodon lamina*, *Rineloricaria cadeae*, *R*. *lanceolata*, *R*. *quadrensis*, and *Spatuloricaria puganensis*. Terminals with only morphological information or DNA data were included as well, in order to populate as much of the diversity contained in these genera as possible and to determine the composition of the group and their position within Loricariinae. Specimens and tissue samples identified as *Farlowella* aff. *amazonum* were originally identified as *F*. *platorhynchus* in collections, which is currently a synonym of *F*. *amazonum*.

### Outgroup selection

Outgroup taxa selection was based on studies dealing with phylogenetic analyses of Loricariidae, Loricariinae, and specific genera of the latter. Armbruster [11], Cramer et al. [13], Lujan et al. [14], and Pereira and Reis [16] suggested different sister-groups to Loricariinae. For this reason, three species belonging to Hypostominae were included: *Ancistrus brevipinnis*, *Chaetostoma breve*, and *Pterygoplichthys lituratus*; five species belonging to the Hypoptopomatinae: *Acestridium scutatum*, *Hisonotus laevior*, *Neoplecostomus microps*, *Pareiorhaphis calmoni*, and *Parotocinclus maculicauda*, and *Hemipsilichthys gobio*, which was selected to root the trees because the Delturinae is the sister group to all loricariids except *Lithogenes* [16].

### Museum acronyms

The institutions visited to examine material and/or carry out extractions for DNA amplification or that sent specimens on loan are those in the list of material examined and whose acronyms are listed by the American Society of Ichthyologists and Herpetologists at (https://asih.org/sites/default/files/2019-04/Sabaj_2019_ASIH_Symbolic_Codes_v7.1.pdf).

### Morphological characters

Characters included here were mainly obtained from Rapp Py-Daniel [28], Ghazzi [36], Fichberg [37], Paixão and Toledo-Piza [38], and Provenzano [39]; additional characters were obtained from studies dealing with other members of the Loricariidae [11, 27, 40, 41], or are proposed for the first time. All characters obtained from previous studies were either maintained as originally proposed or modified to suit the scope of this study and its composition regarding terminals included. Character description follows Sereno [42]. Thus, character statements are composed of four fundamental functional components identified as locator, variable, variable qualifier, and character states. External morphology and coloration were studied based on alcohol-preserved specimens. Bone and cartilage were studied based on double stained and cleared specimens according to Taylor and Van Dyke [43]. Osteological nomenclature follows Paixão and Toledo-Piza [38] and Schaefer [41]. The software Mesquite 3.0.2 [44] was used for the construction of the character matrix. Inapplicable characters were coded as “-”, while missing data was coded as “?”.

### Molecular characters

Covain et al. [15] included almost all loricariine genera in their molecular phylogeny of the subfamily. They used three molecular markers: the mitochondrial 12S and 16S, and the nuclear f-rtn4. Sequences of those markers are available in Genbank (see Table 1 here, Table 2 in [15]), and data from 12S and 16S were included in the present study. The f-rtn4 sequences were not used because the region amplified has introns that make the marker difficult to align. Attemps using the f-rtn-4 resulted in highly unrealistic trees. Lujan et al. [14] performed a molecular phylogenetic analysis of the Loricariidae, and included primers from Li et al. [45] or developed specific primers for loricariids (Table 2). From that study, genes and primers included here are: Cytochrome b (Cytb) from the mitochondrial genome, and Recombination activating genes 1 and 2 (RAG1 and RAG2), and the Myosin Heavy Chain 6 (MyH6) from the nuclear genome. Finally, the NADH dehydrogenase 2 (nd2) from the mitochondrial genome, which proved to be efficient for loricariids [46], was also included.

**Table 1:** Vouchers included for DNA extraction, amplification, and sequencing.

Terminal taxa	Voucher Specimens	GenBank Accession Number					
		12S 16S	Cytb	MyH6	RAG1	RAG2	nd2
**OUTGROUP**							
**Delturinae**							
*Hemipsilichthys gobio*	MCP 42452		MK155309	MK155394	MK155578		MK155447
**Hypoptopomatinae**							
*Acestridium scutatum*	MCP 37785			MK155364	MK155534		MK155414
*Hisonotus laevior*	MCP 23005		MK155344	MK155395	MK155579		
*Parotocinclus maculicauda*	MCP 41911		MK155351	MK155400	MK155589	MK155519	MK155460
*Neoplecostomus microps*	MCP 42432	KR478211^a^			MK155587	MK155517	MK155458
*Pareiorhaphis calmoni*	MCP 41275		MK155352	MK155401	MK155590		MK155461
**Hypostominae**							
*Ancistrus brevipinnis*	MCP 25167		MK155311		MK155535	MK155475	
*Chaetostoma breve*	AUM 4063		MK155314	MK155367	MK155538	MK155476	MK155417
*Pterygoplichthys lituratus*	MCP 35757			MK155402	MK155591	MK155520	
**INGROUP**							
**Loricariinae**							
*Aposturisoma myriodon*	MHNG 2710.035	2435 KR477910^a^	MK155310		MK155523	MK155474	MK155413
*Cteniloricaria napova*	MHNG 2704.030	2440 KR477882^a^	MK155312	MK155365	MK155536		MK155415
*Cteniloricaria platystoma*	AUM 3890	2439 KR477888^a^	MK155313	MK155366	MK155537		MK155416
*Dasyloricaria filamentosa*	CZUT 5104			MK155369	MK155539	MK155477	MK155418
*Dasyloricaria latiura*	STRI 1559	2424 KR477966^a^		MK155371	MK155541	MK155479	MK155420
*Dasyloricaria paucisquama*	CZUT 5105			MK155370	MK155540	MK155478	MK155419
*Farlowella acus*	STRI MER95T-23	2440 KR477936^a^		MK155372		MK155480	MK155421
*Farlowella amazonum*	MCP 45943	2432 KR477937^a^	MK155315	MK155373	MK155542	MK155481	
*Farlowella* aff. *amazonum*	STRI MER95T-26	2435 KR477949^a^	MK155322		MK155551		
*Farlowella curtirostra*	STRI MER95T-15	2435 KR477938^a^	MK155316	MK155374	MK155543	MK155482	MK155422
*Farlowella hahni*	STRI 2205	2437 KR477941^a^	MK155317	MK155375	MK155544	MK155483	MK155423
*Farlowella hasemani*	MCP 36626				MK155545		
*Farlowella knerii*	MHNG 2710.052	2437 KR477954^a^	MK155318		MK155546	MK155484	MK155424
*Farlowella mariaelenae*	STRI MER95T-2	2439 KR477939^a^			MK155547	MK155425	
*Farlowella martini*	STRI VZ-126	2436 KR477940^a^	MK155319		MK155548	MK155485	MK155426
*Farlowella nattereri*	MHNG 2650.099	2439 KR477952^a^					
*Farlowella oxyrryncha*	MCP 44240	2437 KR477960^a^	MK155320	MK155376	MK155549	MK155486	
*Farlowella paraguayensis*	LBP 5217	2434 KR477962^a^	MK155321		MK155550	MK155487	
*Farlowella reticulata*	AUM 3642	2438 KR477942^a^	MK155323		MK155552	MK155488	
*Farlowella rugosa*	AUM 3648	2435 KR477948^a^	MK155324		MK155553	MK155489	
*Farlowella schreitmuelleri*	MHNG 2601.087	2437 KR477943^a^	MK155325	MK155377	MK155554	MK155490	MK155427
*Farlowella smithi*	ANSP 180541	2436 KR477945^a^					
*Farlowella taphorni*	STRI VZ-89	2433 KR477946^a^	MK155326	MK155378	MK155555	MK155491	MK155428
*Farlowella vittata*	AUM 3607	2438 KR477947^a^	MK155327		MK155556	MK155492	MK155429
*Farlowella yarigui*	ICNMHN 17789		MK155328		MK155557	MK155493	
*Harttia carvalhoi*	LBP 2115	2432 KR477891^a^	MK155329	MK155379	MK155558	MK155494	MK155430
*Harttia dissidens*	LBP 5859	2435 KR477892^a^	MK155330	MK155380	MK155559		MK155431
*Harttia duriventris*	LBP 7505	2432 KR477915^a^					
*Harttia fluminensis*	MHNG 2690.013	2435 KR477884^a^					
*Harttia fowleri*	MHNG 2643.022	2442 KR477880^a^	MK155332	MK155382	MK155561	MK155496	MK155433
*Harttia gracilis*	LBP 6331	2433 KR477916^a^	MK155333		MK155562	MK155497	MK155434
*Harttia guianensis*	MHNG 2757.008	2438 KR477885^a^	MK155334	MK155383	MK155563	MK155498	
*Harttia kronei*	MCP 42440	2424 KR477900^a^	MK155335	MK155384	MK155564	MK155499	MK155435
*Harttia leiopleura*	LBP 6847	2435 KR477918^a^	MK155336		MK155565		MK155436
*Harttia longipinna*	DZSJRP 2819	2429 KR477903^a^	MK155337	MK155385	MK155566	MK155500	MK155437
*Harttia loricariformis*	LBP 2121	2435 KR477896^a^			MK155567		MK155438
*Harttia novalimensis*	LBP 5836	2429 KR477897^a^	MK155338	MK155386	MK155568	MK155501	MK155439
*Harttia punctata*	MHNG 2645.053	2431 KR477893^a^	MK155339	MK155387	MK155569		MK155440
*Harttia surinamensis*	MHNG 2674.042	2438 KR477883^a^	MK155340	MK155388	MK155570	MK155502	MK155441
*Harttia torrenticola*	LBP 5835	2433 KR477913^a^	MK155341	MK155389	MK155571	MK155503	MK155442
*Harttia tuna*	MHNG 2704.029	2437 KR477909^a^	MK155342	MK155390	MK155572	MK155504	MK155443
*Harttiella crassicauda*	AUM 4198	2418 KR478145^a^		MK155391	MK155573		
*Harttiella intermedia*	MHNG 2713.087	2418 KR478164^a^			MK155574	MK155505	MK155444
*Harttiella longicauda*	MHNG 2699.070	2419 KR478159^a^	MK155343	MK155392	MK155575	MK155506	
*Harttiella lucifer*	MHNG 2754.082	2414 KR478153^a^			MK155576		MK155445
*Harttiella pilosa*	MHNG 2682.055	2419 KR478138^a^		MK155393	MK155577	MK155507	MK155446
*Hemiodontichthys acipenserinus*	MCP 28819	2424 KR478142^a^					
*Lamontichthys filamentosus*	AUM 4024	2433 KR477930^a^	MK155346	MK155397	MK155580		MK155450
*Lamontichthys llanero*	MHNG 2749.019	2434 KR477928^a^	MK155347	MK155398	MK155581	MK155510	MK155451
*Lamontichthys stibaros*	AUM 57480	2433 KR477931^a^	KP960068.1^c^	KP960374.1^c^	KP959909^c^	KP960219.1^c^	
*Limatulichthys griseus*	MCP 46112	1689 EU310450^b^		MK155399	MK155582	MK155511	MK155452
*Loricaria lundbergi*	MCP 46205				MK155585	MK155514	MK155455
*Loricariichthys anus*	MCP 28415	2430 KR478175^a^			MK155583	MK155512	MK155453
*Loricariichthys platymetopon*	MCP 21614	2427 KR478118^a^			MK155584	MK155513	MK155454
*Metaloricaria nijsseni*	MHNG 2756.054	2437 KR477967^a^	MK155348		MK155586	MK155515	MK155456
*Metaloricaria paucidens*	MHNG 2757.023	2439 KR477932^a^	MK155349			MK155516	MK155457
*Pseudohemiodon lamina*	MCP 36579			MK155368			
*Pterosturisoma microps*	MHNG 2677.072	2439 KR477921^a^	MK155350		MK155588	MK155518	MK155459
*Rineloricaria cadeae*	MCP 21217	2424 KR477987^a^			MK155592	MK155521	
*Rineloricaria lanceolata*	MCP 34465		MK155345	MK155396		MK155509	MK155449
*Rineloricaria quadrensis*	MCP 21195				MK155593	MK155522	MK155462
*Spatuloricaria puganensis*	AUM 4067	2421 KR478043^a^	MK155357	MK155406	MK155597	MK155526	MK155466
*Sturisoma guentheri*	ANSP 182587	2446 KR477926^a^					
*Sturisoma monopelte*	AUM 3616	1707 EU310461^a^	MK155353	MK155403	MK155594		
*Sturisoma nigrirostrum*	ANSP 178322	2444 KR478162^a^	MK155354	MK155404		MK155523	MK155463
*Sturisoma robustum*	MHNG 2677.002	2443 KR478161^a^	MK155355		MK155595	MK155524	MK155464
*Sturisoma* aff. *tenuirostre*	MCP 34083		MK155356	MK155405	MK155596	MK155525	MK155465
*Sturisomatichthys aureus*	MHNG 2684.019	2442 KR478160^a^	MK155358	MK155407	MK155598	MK155527	MK155467
*Sturisomatichthys citurensis*	STRI 3587	1703 EU310462^b^	MK155359				MK155468
*Sturisomatichthys dariensis*	STRI 26795	2440 KR477922^a^	MK155360	MK155408	MK155599	MK155528	MK155469
*Sturisomatichthys festivus*	STRI MER95T-20	2443 KR477923^a^					
*Sturisomatichthys frenatus*	STRI 872		MK155361	MK155409	MK155600	MK155529	MK155470
*Sturisomatichthys leightoni*	MPUJ 7865	2440 KR477927^a^		MK155410	MK155601	MK155530	MK155471
*Sturisomatichthys panamensis*	MHNG 2674.058	2443 KR478163^a^	MK155362	MK155411	MK155602	MK155531	MK155472
*Sturisomatichthys tamanae*	ANSP 198426		MK155363	MK155412	MK155603	MK155532	MK155473

Accession numbers marked with “a” generated by Covain et al. [15]; “b” generated by Covain et al. [18]; “c” generated by Lujan et al. [14]. Unmarked accession numbers generated in this study.

**Table 2:** Primers and annealing temperatures used in amplification of molecular markers.

DNA marker	Primer Name	Primer Sequences (5’–>3’)	Annealing temperatura	Reference	Notes
**Cytb**	CytbFa	TCCCACCCGGACTCTAACCGA	Touchdown 50°C—62°C	Lujan et al. [14]	
	CytbRa	CCGGATTACAAGACCGGCGCT	Touchdown 50°C—62°C	Lujan et al. [14]	
**MyH6**	myh6_F459	CATMTTYTCCATCTCAGATAATGC	Touchdown 53°C—62°C	Li et al. [45]	1st PCR forward primer
	myh6_F507	GGAGAATCARTCKGTGCTCATCA	Touchdown 53°C—62°C	Li et al. [45]	2nd PCR forward primer
	myh6_R1325	ATTCTCACCACCATCCAGTTGAA	Touchdown 53°C—62°C	Li et al. [45]	1st PCR reverse primer
	myh6_R1322	CTCACCACCATCCAGTTGAACAT	Touchdown 53°C—62°C	Li et al. [45]	2nd PCR reverse primer
**RAG1**	RAG1Fa	CCTGGTTTTCATGCATTTGAGTGGCA	Touchdown 48°C—60°C	Lujan et al. [14]	
	RAG1Ra	AGGGCATCTAATGTGGGCTGTGT	Touchdown 48°C—60°C	Lujan et al. [14]	
**RAG2**	RAG2Fc	ATGGAGGCCGAACACCCAACA	Touchdown 53°C—58°C	Lujan et al. [14]	
	RAG2R961	CGCTGCTGWACTCCATTT	Touchdown 53°C—58°C	Lujan et al. [14]	
**nd2**	nd2_Dist_f	AGCTTTTGGGCCCATACCCCA	58°C	Arroyave et al. [46]	
	nd2_Dist_r	AGGRACTAGGAGATTTTCACTCCTGCT	58°C	Arroyave et al. [46]	

Tissues were fixed in absolute ethanol and stored in a freezer. Samples were obtained from museum specimens and vouchers were analyzed to certify identifications. See Table 1 for voucher numbers and collections in which they are deposited.

Total DNA extractions were obtained from muscle or fin samples using DNeasy Blood and Tissue kit (Qiagen), following the manufacturer’s protocol. Polymerase Chain Reaction (PCR) was used to amplify molecular fragments in a total reaction volume of 25μl: 2μl for mitochondrial markers and 4μl for nuclear markers of DNA template; 1.25μl of primer at 10μM (primers detailed in Table 2); 8μl (for mitochondrial markers) and 12.5μl (for nuclear markers) of PCR Master Mix 2X (Hotstartaq Master Mix Kit, Qiagen), and completed with nuclease-free water. A nested PCR protocol for the nuclear marker MyH6 was employed following Lujan et al. [14]; 1μl from the first PCR product was employed to perform a second PCR, with half the volume for the remaining reagents of those listed above. Cycles of amplification were programmed following the conditions recommended by the Taq DNA polymerase manufacturer, plus published protocols [9, 14, 45, 46], and those created here. Annealing temperatures are shown in Table 2.

A sample of 2μl of PCR product plus 2μl of BlueJuice (Invitrogen) diluted with nuclease-free water and mixed with 0.8μl of GelRed (Invitrogen) was loaded into agarose gel to run an electrophoresis. Successful DNA amplifications were corroborated by visual observation in the electrophoresis gel of colored fragments using an ultraviolet light box. Size fragments were compared with standard sizes of the Low DNA MassLadder (Invitrogen) to corroborate the correct length of the amplified fragment.

Functional Biosciences (USA) sequenced PCR products. Forward and reverse contigs were assembled and edited using the software Geneious 9.1, and subsequently checked to identify ambiguous sequence portions. Identity of vouchers of GenBank data of ingroup terminals, deposited by Covain et al. [15, 18] were corroborated at MHNG; see Table 1 for vouchers of data from Genbank. Vouchers of Genbank data deposited by Lujan et al. [14] (Table 1), belonging to *Lamontichthys stibaros*, were not verified.

Sequences were aligned using the MUSCLE algorithm [47] with default parameters as implemented by the software Geneious 9.1. For coding genes (all except 12S and 16S), codon positions were visualized using Mesquite 3.0.2 [44] and corroborated using Geneious 9.1 for posterior partitions. Models of nucleotide substitution for each gene and codon partition were obtained with the software PartitionFinder v1.1.1 [48]. See Table 3 for the best model of each position of each gene. Finally, the concatenation of both the morphological and molecular matrices was achieved using SequenceMatrix 1.8 software [49], and exported as NEXUS and TNT files.

**Table 3:** Nucleotide substitution models by gene and codon position.

Subset	Scheme of partition	Partitions	Best-fit model
1	12S16S	197–2698	GTR+I+G
2	Cytb first position	2699-3755/3	TVMEF+I+G
3	Cytb second position	2700-3753/3	GTR+I+G
4	Cytb third position	2701-3754/3	GTR+G
5	MyH6 first position	3756-4392/3	GTR+G
6	MyH6 second position	3757-4390/3	GTR+I+G
7	MyH6 third position	3758-4391/3	GTR+I+G
8	RAG1 first position	4393-5104/3	TRNEF+I+G
9	RAG1 second position	4394-5105/3	TVM+I+G
10	RAG1 third position	4395-5106/3	GTR+I+G
11	RAG2 first position	5107-5884/3	SYM+G
12	RAG2 second position	5108-5885/3	TVM+I+G
13	RAG2 third position	5109-5883/3	HKY+I+G
14	nd2 first position	5886-6819/3	TRN+I+G
15	nd2 second position	5887-6817/3	GTR+G
16	nd2 third position	5888-6818/3	TVM+G
lnL: -65297.069397		Parameters: 312	

### Tree searching methods

Two different methods were used to analyze the matrix. A Bayesian Inference analysis (BI) was conducted in MrBayes v3.1.2 [50, 51] via the CIPRES web portal [52], including all best-fit models as detailed in Table 3. MrBayes was programmed to run for 100 million generations, with two runs of four independent MCMC chains (three heated, one cold), sampling one tree every one thousand generations. The first 25% generations were discarded as burn-in, as indicated by TRACER v.1.6 [53]. A less parameterized, Maximum Likelihood analysis was also performed in RAxML, using the CIPRES web portal [52], in order to compare results with the Bayesian inference. In addition, an analysis using Maximum Parsimony (MP) was performed with the software TNT (Tree analysis using New Technology, version 1.1) [54], where gaps were treated as missing data and all characters had equal weights. Five runs of a combination of Ratchet (400 iterations), Tree Drift (150 iterations) and Tree Fusing (15 rounds) were used to generate trees, followed by an extensive branch swapping using TBR. A strict consensus of the most parsimonious trees was generated in order to summarize the phylogenetic hypotheses. Bremer support values were calculated through TNT by sequencially searching suboptimal trees one, two, …, “n” steps longer than the shortest trees, where “n” went up to 100, and saving up to 1,000 trees per search.

The classification of the Loricariinae presented below and in S1 File is based on the BI tree topology and was produced by sequencing of taxa, as proposed by Wiley [55], where an asymmetrical part of a cladogram is placed in the same categorical rank and sequenced in phylogenetic order. Thus, a list of taxa with the same category forms a completely dichotomous sequence, with the first taxon being the sister group to all subsequent taxa and so on. The morphological synapomorphies for each clade as shown in S1 File are from the MP analysis. In addition, a list of all molecular transformations for subfamily, tribes, and genera is given in S2 File.

## Results

### Character description

#### Morphological characters

Character descriptions are grouped by anatomical units. Words such as most or some refer only to those species of the taxa examined. Data matrix of morphological characters as S3 File.

#### Neurocranium

1. Mesethmoid, anterior portion, lateral expansion: (0) present; (1) absent. CI = 0.11. [28] Ch. 1, [36] Ch. 1, [37] Ch. 1.

**Fig 1:**
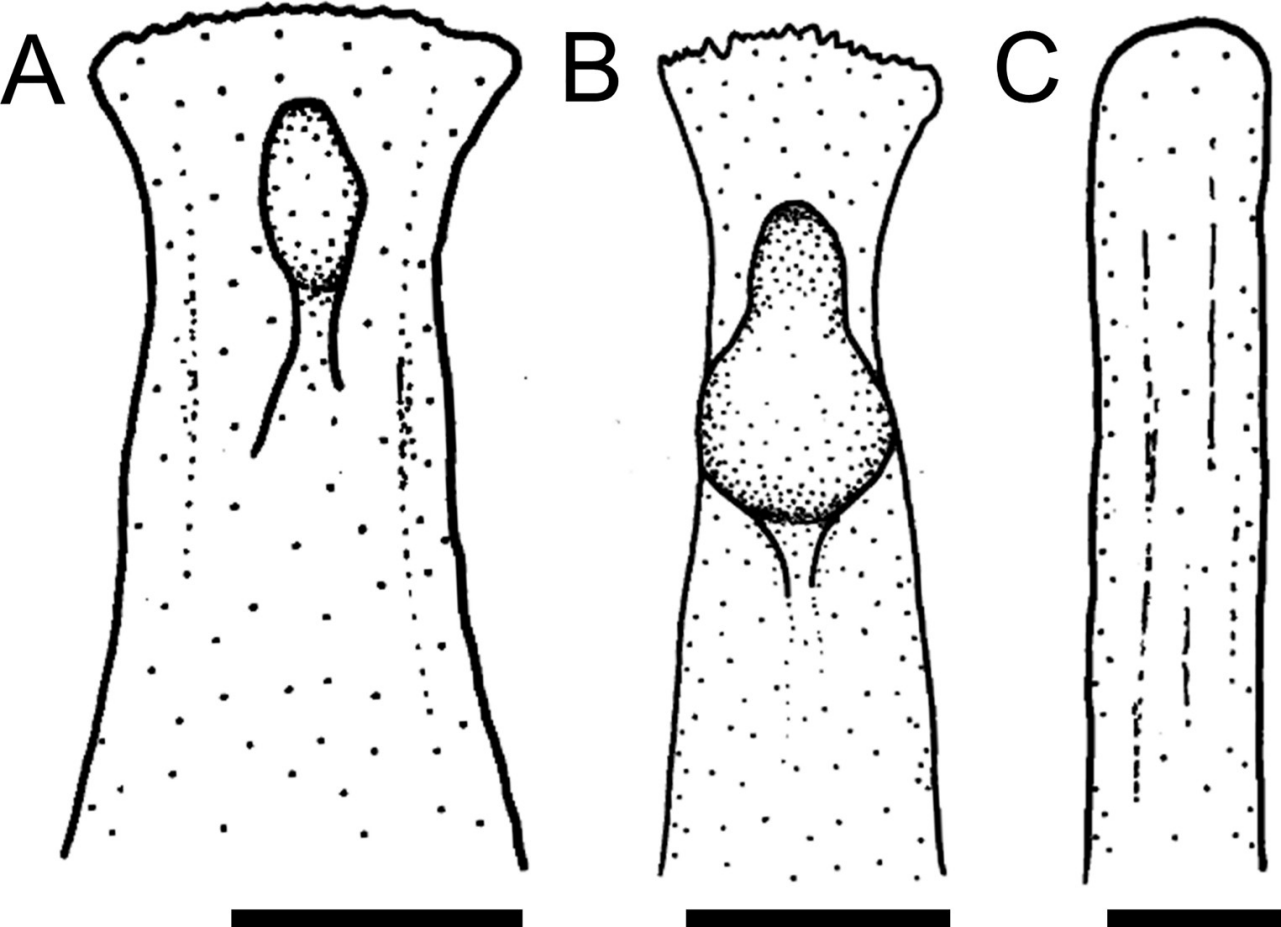
Anterior portion of mesethmoid in ventral view. **A.** *Rineloricaria cadeae*, MCP 25920; **B.** *Harttia guianensis*, MHNG 2643.033; **C.** *Farlowella henriquei*, MCP 41992.Scale bar 1 mm.

The anterior tip of the mesethmoid can be laterally expanded (state 0; Fig 1A and 1B) as in most loricariids, or straight, not expanded (state 1; Fig 1C) in members of the Farlowellini, except *Lamontichthys* (except *L*. *filamentosus*).

2. Mesethmoid, anterior tip, ventral depression: (0) absent; (1) present. CI = 0.16. [37] Ch. 7, [38] Ch. 1.

Paixão and Toledo-Piza [38] stated that the ventral depression at anterior tip of the mesethmoid was only present in *Lamontichthys filamentosus*, *L*. *llanero*, and *L*. *maracaibero* (state 1; [38: fig 15A]). Additionally, that depression was observed in *Metaloricaria*, *Harttia carvalhoi*, *Harttiella longicauda*, *Pseudohemiodon lamina*, and *Limatulichthys griseus*. *Lamontichthys maracaibero* was not examined.

3. Mesethmoid, anterior portion, coverage: (0) naked skin; (1) plates. CI = 0.06. [28] Ch. 8, [37] Ch. 5 and 6, [38] Ch. 2.

**Fig 2:**
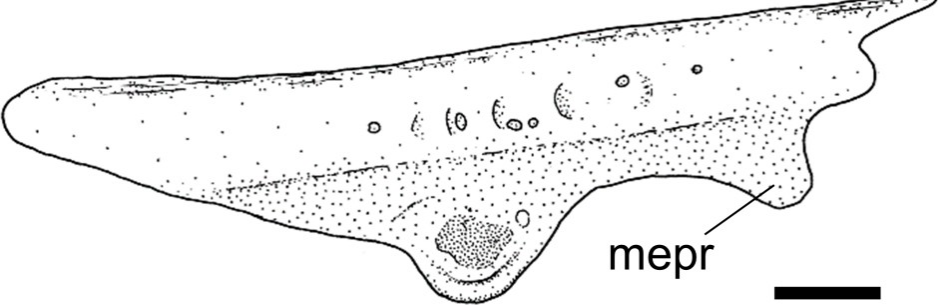
Mesethmoid in lateral view. **A.** *Sturisomatichthys festivus*, CAS 168512. **Abbreviations:** md = mesethmoid disk; mpr = mesethmoid process. Scale bar 1 mm.

**Fig 3:**
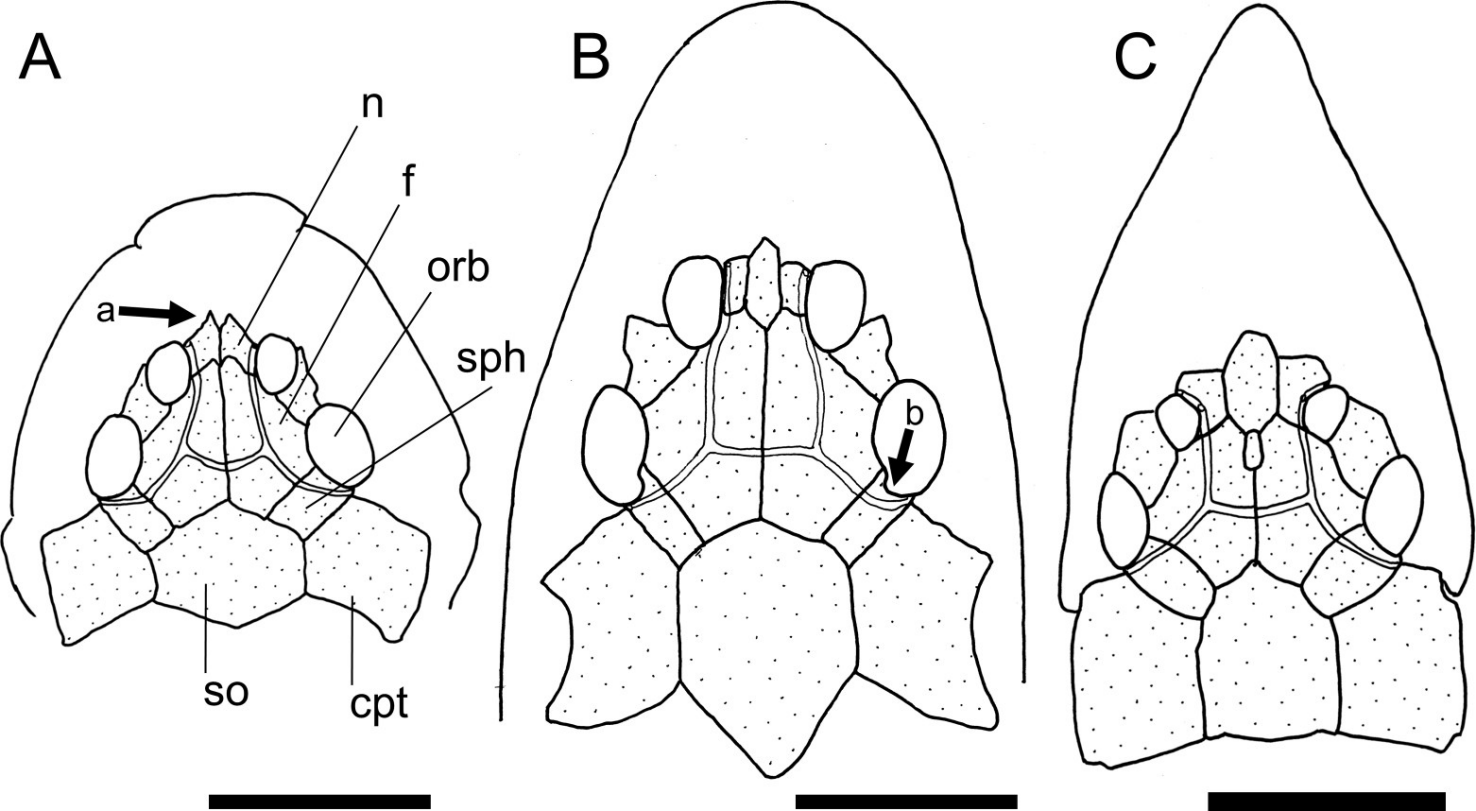
Neurocranium in dorsal view. **A.** *Harttiella crassicauda*, AUM 50387; **B.** *Rineloricaria lanceolata*, MCP 36454; **C.** *Aposturisoma myriodon*, MHNG 2710.035. **Abbreviations:** cpt = compound pterotic; f = frontal; n = nasal; orb = orbit; so = supraoccipital; sph = sphenotic. Arrows: see text. Scale bar 5 mm.

**Fig 4:**
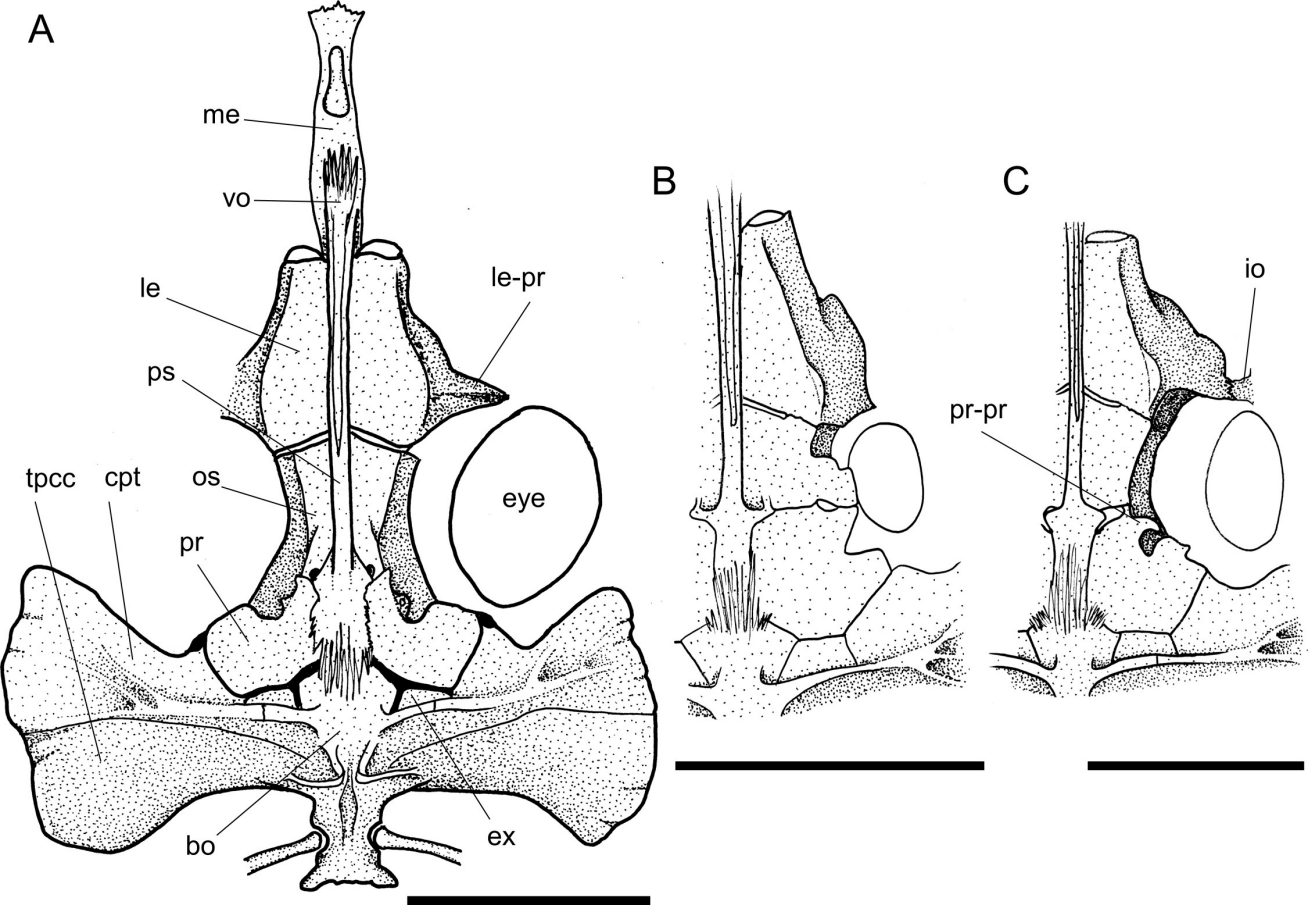
Neurocranium in ventral view. **A.** *Harttia guianensis*, MHNG 2643.033; **B.** *Pterosturisoma microps*, MZUSP 79909; **C.** *Sturisomatichthys panamensis*, USNM 316293. **Abbreviations:** bo = basioccipital; cpt = compound pterotic; ex = exoccipital; io = infraorbital; le = lateral ethmoid; le-pr = lateral ethmoid process; me = mesethmoid; os = orbitosphenoid; pr = prootic; pr-pr = prootic process; ps = parasphenoid; tpcc = transverse process of complex centrum; vo = vomer. Scale bar 5 mm.

The absence (state 0; [56: fig 2; 81: fig 3A]) of plates covering the snout tip at the anterior border of the mesethmoid was found mainly in *Harttia*, *Sturisoma*, *Sturisomatichthys*, some Loricariini, and outgroups. On the other hand, presence of plates (state 1; [56: fig 4; 81: fig 3B-3F) was found to be variable within *Farlowella* and other members of Loricariinae, including species of the above-mentioned genera, except *Harttia*.

4. Mesethmoid, ventral process: (0) absent; (1) present; CI = 0.20.

The mesethmoid of loricariids has a ventral disk, which shows different degrees of development and a variety of shapes. A mesethmoid ventral process posterior to that disk was observed in most members of the Farlowellini. In outgroups, *Lamontichthys*, Harttiini, and Loricariini that structure is absent (state 0). A variably-developed process occurs in *Aposturisoma*, *Pterosturisoma*, *Sturisomatichthys*, and *Sturisoma* (state 1; Fig 2).

5. Mesethmoid, ventral disk, shape: (0) broad disk with dorsal fenestra; (1) robust, circular in lateral view, without fenestra; (2) circular lamina; (3) keel-shaped lamina. CI = 0.30. [37] Ch. 4, [38] Ch. 3, [39] Ch. 2 (modified).

In contrast to the findings of Fichberg [37], Paixão and Toledo-Piza [38], and Provenzano [39], less variation regarding the mesethmoid ventral disk shape was found here. A broad disk with a fenestra was only found in outgroups (state 0); a robust disk, circular in lateral view, without a fenestra (state 1; Fig 2), is present in genera of both Harttiini and Farlowellini; a ventral disk as a circular lamina (state 2) was found in *Sturisoma* and among some *Farlowella* species; and a keel-shaped lamina (state 3) was observed in some Loricariini.

6. Mesethmoid, ventral disk, position: (0) terminal; (1) sub-terminal; (2) posterior. CI = 0.20. [36] Ch. 3, [38] Ch. 4, [39] Ch. 3.

The position of the ventral disk of the mesethmoid also showed variation across the Loricariidae. A terminal disk (state 0) was variably present among genera of the Loricariini and outgroups, while a sub-terminal disk (state 1; Fig 2) was found across the Loricariini, the Harttiini, and outgroups. Finally, a posteriorly-displaced disk (state 2) was found in the Farlowellini, *Hemiodontichthys acipenserinus* and, outside the Loricariinae, in *Acestridium scutatum*.

7. Mesethmoid, ventrolateral crest (0) narrow lamina; (1) short, on margin of proximal portion; (2) well-developed on posterior margin; (3) well-developed along entire mesethmoid. CI = 0.13. [28] Ch. 7, [36] Ch. 6 (modified).

**Fig 5:**
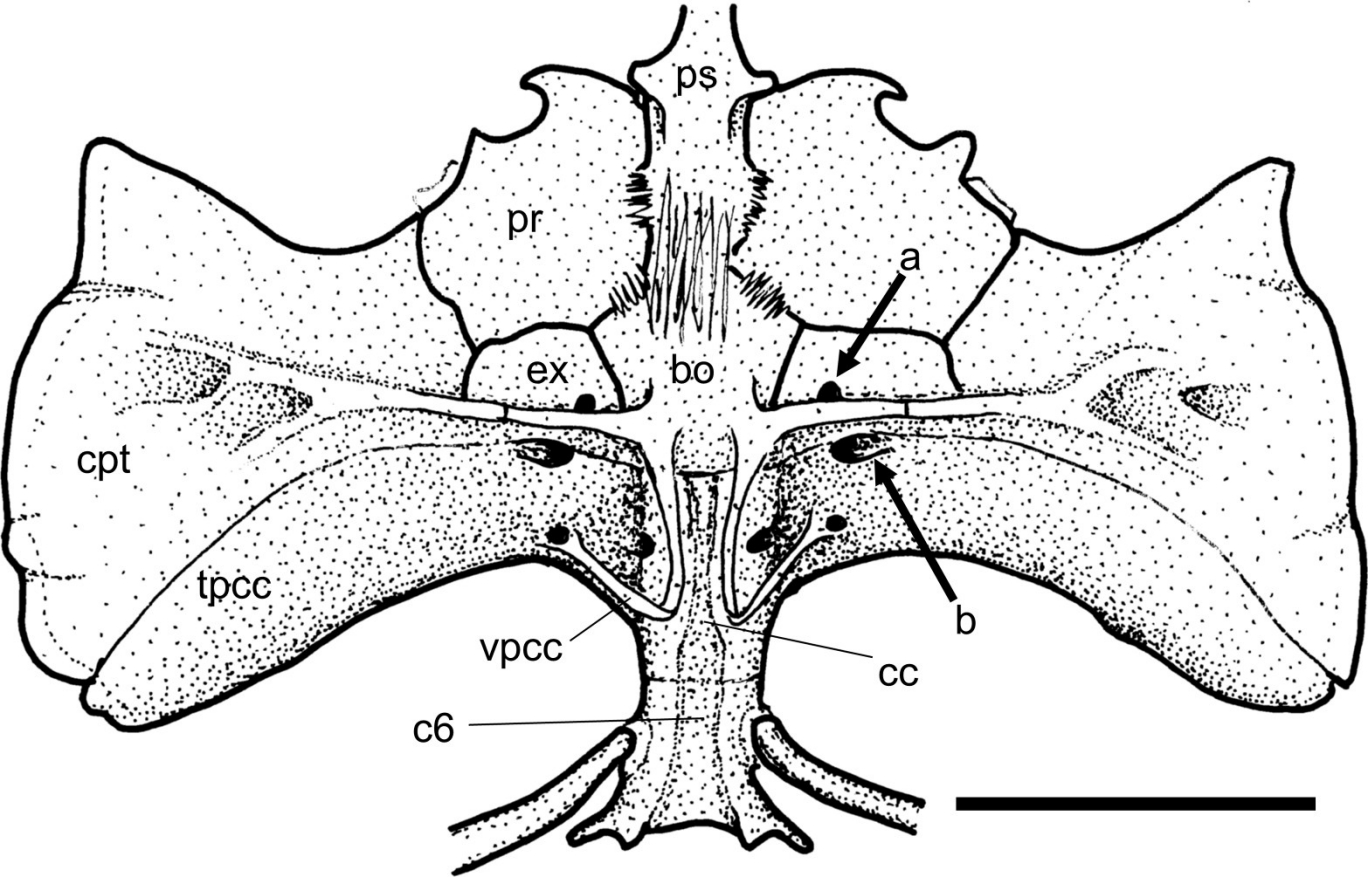
Posterior portion of cranium in ventral view. *Sturisomatichthys festivus*, CAS 168512. **Abbreviations:** bo = basioccipital; c6 = vertebral centrum 6; cc = complex centrum; cpt = compound pterotic; ex = exoccipital; pr = prootic; ps = parasphenoid; tpcc = transverse process of complex centrum; vpcc = ventralprocess of complex centrum. Arrows: see text. Scale bar 5 mm.

**Fig 6:**
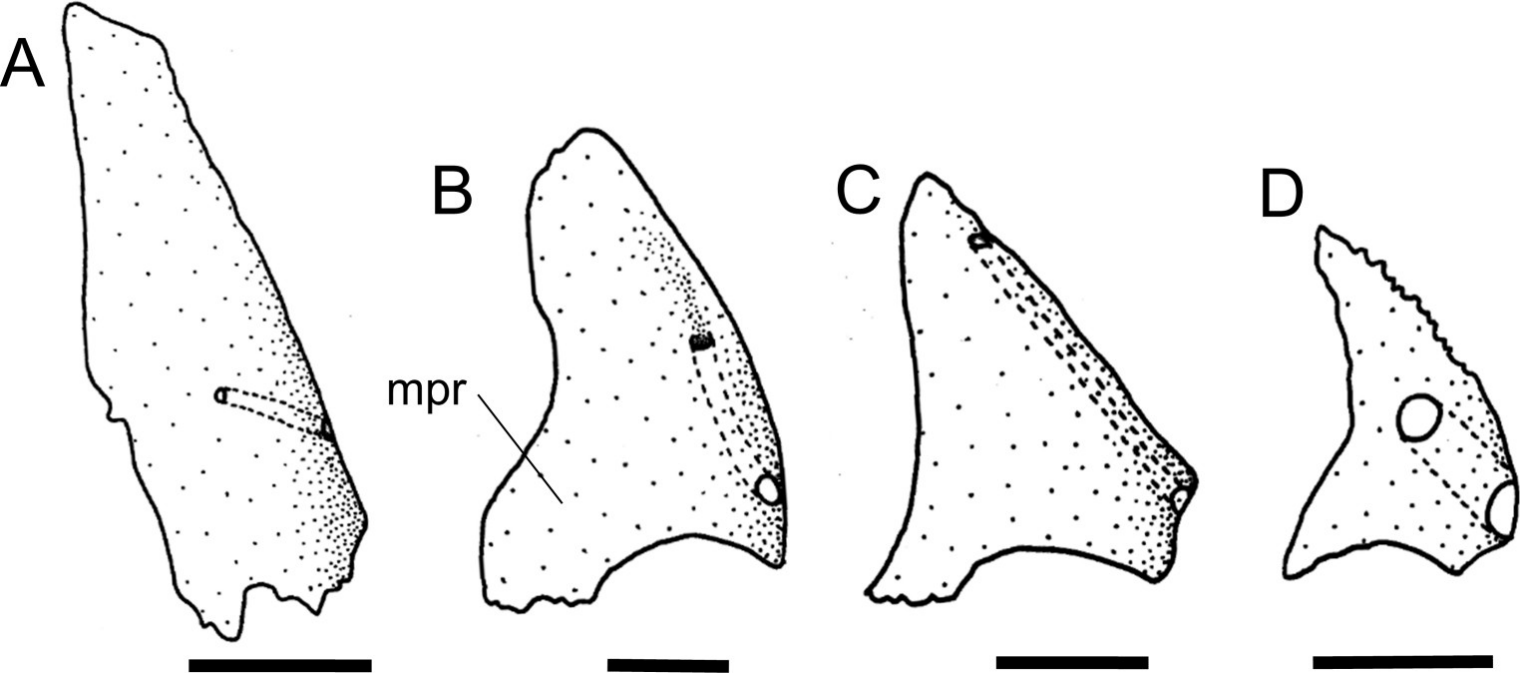
Canal-bearing cheek plate in ventral view, left side. **A.** *Farlowella rugosa*, AUM 48805; **B.** *Cteniloricaria platystoma*, AUM 48174; **C.** *Harttia guianensis*, MHNG 2643.033; **D.** *Harttiella crassicauda*, AUM 50387. Anterior towards top. **Abbreviations:** mpr = medial process. Scale bar 1 mm.

The ventrolateral crest was found to be either narrow (state 0; [36: fig 3A]) mostly in *Harttia*, *Lamontichthys*, and some outgroups, short (state 1; [36: fig 6]) in some *Harttia*, most *Sturisomatichthys*, and some Loricariini, well developed on the posterior margin of the mesethmoid (state 2; [36: fig 5A]) mainly in *Farlowella* and *Sturisoma*, or well developed along the entire mesethmoid (state 3; [36: fig 2]) in some Loricariini. Its development was herein found to be informative within the Harttiini and Farlowellini.

8. Sphenotic, lateral process: (0) short; (1) reduced or absent; (2) thin and long; (3) broad and long. CI = 0.13. [11] Ch. 116, [37] Ch. 28, [38] Ch. 8 (modified).

The sphenotic contributes to the posterior border of the orbital rim. Its contribution can vary depending on the absence (state 1; [36: fig 10B]) of a lateral process as in *Farlowella*, *Harttiella*, and some *Harttia*, as well as on its degree of development, being short (state 0; [36: fig 8]) in *Sturisoma*, most *Sturisomatichthys*, and some outgroups; thin and long (state 2; [36: fig 9]) in a few *Sturisomatichthys*, some *Harttia* and some Loricariini, or broad and long (state 3; [36: fig 10A]) mostly in Loricariini and outgroups. The type of the sphenotic process is useful to diagnose the species of *Sturisoma*.

9. Orbit, posterior notch: (0) absent; (1) present. CI = 1.00. [38] Ch. 9.

The presence (state 1; Fig 3B, arrow a) of an orbital posterior notch was found to be an exclusive synapomorphy of the Loricariini.

10. Sphenotic, width: (0) narrow, less than half frontal length; (1) wide, more than half frontal length. CI = 0.20. [28] Ch. 14, [36] Ch. 12 (modified).

Within the Harttiini, *Cteniloricaria platystoma* and *Harttia* have a narrow sphenotic, less than half length of the frontal (state 0; Fig 3A). A wide sphenotic (state 1; Fig 3B and 3C) can be found across the remaining Loricariinae and in outgroups.

11. Vomer, anterior process, suture: (0) short, interdigitate suture; (1) long, interdigitate suture; (2) absent. CI = 0.14. [28] Ch. 9, [36] Ch. 8 (modified).

The anterior process of the vomer has short or long sutures with the mesethmoid, the latter due to the presence of long spike-like sutures in ventral view. Short suture (state 0; [38: fig 15A]) is the general condition for the Harttiini, while for the Loricariini the vomer suture is either short, long (state 1; [38: fig 16B]), or absent (state 2).

12. Lateral ethmoid, contact with metapterygoid: (0) broad suture, in contact along entire lateroventral border of lateral ethmoid; (1) contact through anterior and posterior ends; (2) contact through anterior end only; (3) contact through posterior end only; (-) inapplicable. CI = 0.15. [27] Ch. 4, [28] Ch. 10 and 11, [36] Ch. 9, [37] Ch. 10, [38] Ch. 7, [39] Ch. 8, [40], [41] Ch. 1 (modified).

Within Loricariinae, a broad suture in contact along the entire lateroventral border of the lateral ethmoid is the general condition for the Loricariini (state 0; [38: fig 18B]). On the other hand, contact through anterior and posterior ends (state 1; [38: fig 18C]), anterior end only (state 2), or posterior end only (state 3; [38: fig 18A]), are found throughout Loricariidae.

13. Lateral ethmoid, laterodorsal lamina: (0) well-developed fold; (1) pointed; (2) expanded; (3) strut-like; (4) absent. CI = 0.33. [27] Ch. 6, [28] Ch. 12, [36] Ch. 11, [37] Ch. 13.

**Fig 7:**
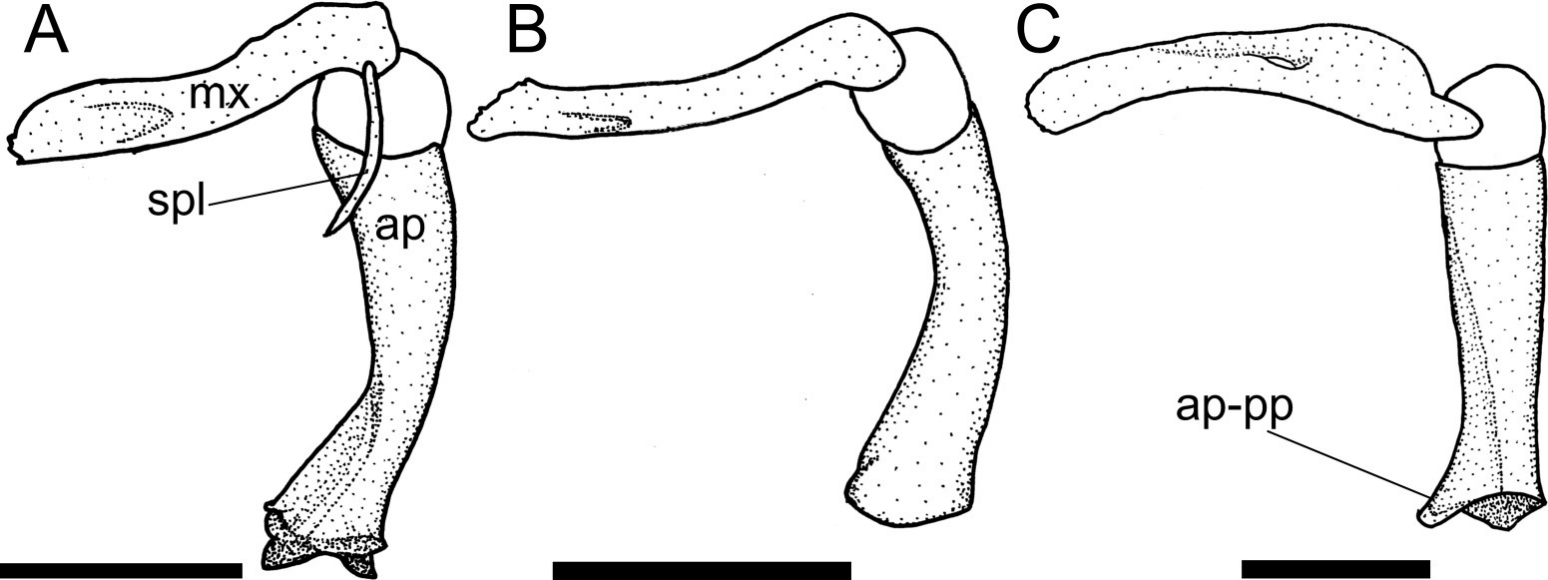
Autopalatine and maxilla, dorsal view. **A.** *Sturisomatichthys reinae*, NRM 15155; **B.** *Farlowella mariaelenae*, USNM 349392; **C.** *Harttia gracilis*, MZUSP 99678. **Abbreviations:** ap = autopalatine; spl = autopalatine splint; mx = maxilla; ap-pp = posterior process of autopalatine. Scale bar 2 mm.

The lateral ethmoid have a dorsolateral lamina that contributes to the encapsulation of the nasal organ. Such lamina was found either as a well-developed fold (state 0; [28: fig 7A]) in some *Lamontichthys* and outgroups, a pointed process (state 1; [28: fig 7B]) in Harttiini, and most *Sturisoma*, an expanded process (state 2; Fig 3A) in *Farlowella*, *Sturisomatichthys*, and some Loricariini, or a strut-like process (state 3; Fig 4A) in Hypoptopomatinae. Such lamina can also be absent (state 4; Fig 3B) in most Loricariini.

14. Lateral ethmoid, posterolateral border: (0) not reaching to orbital rim; (1) well-developed, reaching to orbital rim. CI = 0.14. [28] Ch. 13, [37] Ch. 14.

Across Loricariidae the posterolateral border of the lateral ethmoid is either poorly developed or broad, but not reaches the orbital rim (state 0; Fig 4A and 4B) in most taxa examined, or well developed and reaches to the orbital rim, articulating to the infraorbital 4 (state 1; Fig 4C) in *Dasyloricaria filamentosa*, *Farlowella isbruckeri*, *F*. *mariaelenae*, *Limatulichthys griseus*, *Loricariichthys anus*, *Metaloricaria*, and *Pseudohemiodon lamina*.

15. Nasal bone, proportions: (0) longer than broad; (1) broader than long. CI = 0.33. [37] Ch. 57 (modified).

The general condition observed among the Loricariinae is a nasal bone longer than broad (state 0; Fig 3A and 3B). The only exceptions observed were *Aposturisoma* and *Loricariichthys* (state 1; Fig 3C).

16. Nasal bone, shape: (0) slender rectangle; (1) irregular. CI = 0.04. [28] Ch. 171, [37] Ch. 56.

Two different states were found regarding the shape of the nasal bone, which can be an elongate, slender rectangular (state 0; Fig 3B) in outgroups, some Loricariini, and some Harttiini and Farlowellini, or an irregular squarish bone (state 1; Fig 3A and 3C) on most Farlowellini.

17. Nasal bone, anterior margin, anterior extension: (0) absent; (1) present. CI = 0.04.

Besides the general shape of the nasal, this bone either lacks an anterior extension in some Loricariini and most *Farlowella* and *Harttia* (state 0; Fig 3B), or possesses a pointed extension on the anterior margin (state 1; Fig 3A, arrow b) in *Sturisoma*, most *Sturisomatichthys*, and some *Harttia*, Loricariini and outgroups.

18. Orbitosphenoid, lateral expansion: (0) reduced, much narrower than prootic; (1) well developed, almost as wide as prootic. CI = 0.07. [38] Ch. 4.

The orbitosphenoid has a lateral expanded lamina variably in size. In Harttiini, *Sturisomatichthys*, some *Sturisoma*, and *Farlowella* the lateral lamina is reduced and much narrower that the prootic (state 0; Fig 4A and 4C). In *Pterosturisoma*, some Loricariini, and outgroups the orbitosphenoid is expanded laterally, becoming almost as wide as the prootic (state 1; Fig 4B).

19. Frontal, dorsal border of orbit, participation: (0) without participation; (1) reduced participation; (2) contributing 1/3 of dorsal border of orbit; (3) extensive participation, half of dorsal border of orbit; (4) contributing to almost entire dorsal border of orbit rim. CI = 0.13. [28] Ch. 15, [37] Ch. 17, [39] Ch. 30 (modified).

The participation of the frontal in the dorsal border of the orbit is quite variable. The frontal can be distant from the orbital rim (state 0), have a small participation in the orbital border, with only a tip contacting the orbit (state 1); it can contribute to about 1/3 of dorsal border of orbit (state 2; Fig 3C), have an extensive participation, covering half or slightly more of the orbit rim, as in Harttiini (state 3; Fig 3A), or it can compose almost entirely the dorsal border of the orbit, as in Loricariini (state 4; Fig 3B). In the Harttiini the frontal has an extensive participation, covering half or slightly more of the dorsal border of orbit (Fig 3A).

20. Basioccipital, lateral process: (0) absent; (1) small, shorter than basioccipital length; (2) large, longer than basioccipital length. CI = 0.08. [28] Ch. 21, [37] Ch. 26, [39] Ch. 19.

Among the taxa examined the basioccipital bears a lateral process that varies in length. The process can be either absent (state 0; [28: fig 3A]) in most *Sturisoma*, some *Sturisomatichthys*, and outgroups, shorter than the length of the basioccipital (state 1; [28: fig 7A]) mostly in *Farlowella* and *Harttia*, or longer than the length of the latter bone (state 2; [28: fig 7B]) in most Loricariini and outgroups.

21. Exoccipital, ventral lamina, ventral expansion: (0) thick lamina, slightly expanded ventrally; (1) thin lamina, conspicuosly expanded ventrally; (2) lamina absent. CI = 0.16. [39] Ch. 21 (modified).

According to Provenzano [39], the exoccipital of loricariids may have a ventral lamina that intervenes in the contact between the exoccipital and the compound pterotic. Across the taxa examined here, a variation was observed in such a lamina, which can be thick and little expanded ventally (state 0) in some outgroups, thin and conspicually expanded ventrally (state 1; [39: fig 70]) in Loricariini, *Cteniloricaria*, *Farlowella*, *Sturisoma*, *Sturisomatichthys*, and most *Lamontichthys*, or be absent (state 2) in *Harttia*, *Harttiella*, *Pterosturisoma*, *Lamontichthys filamentosus*, and some outgroups.

22. Exoccipital, ventral lamina, contact with transcapular ligament: (0) not in contact with transcapular ligament; (1) partially in contact with transcapular ligament; (2) in contact along its entire length; (-) inapplicable. CI = 0.18.

A ventral lamina of the exoccipital varies in length and thus in the extension of contact with the transcapular ligament. Such lamina showed no contact at all with the transcapular ligament (state 0) in *Hemipsilichthys gobio* and the Hypoptopomatinae, partial contact (state 1) in most *Farlowella*, *Lamontichthys*, Loricariini, and some *Sturisoma* and *Sturisomatichthys*, or contact along the entire length of the ligament (state 2) in a few *Farlowella*, and some *Sturisoma*, *Sturisomatichthys*, and Loricariini. Moreover, the absence of such extension in *Harttia*, *Harttiella*, *Lamontichthys filamentosus*, *Pterosturisoma microps*, and some outgroups, rendered this character as inapplicable for such taxa.

23. Exoccipital, shape, ventral view: (0) expanded, squarish; (1) narrow, rectangular. CI = 0.09. [37] Ch. 24.

Two different shapes of the exoccipital were found in the taxa examined. The exoccipital can be expanded and squarish (state 0; [28: fig 3B]) in outgroups and most Harttiini, or narrow and rectangular (state 1; Fig 4) on most Farlowellini.

24. Exoccipital, fenestra between posterior margin and lateral projection of basioccipital: (0) present; (1) absent. CI = 0.07.

A small fenestra between the posterior margin of the exoccipital and the lateral projection of the basioccipital is generally present in most Loricariidae examined (state 0; Fig 5, arrow a).

25. Basioccipital, articulation to transcapular ligament: (0) absent; (1) present. CI = 0.10. [28] Ch. 18, [36] Ch. 18, [37] Ch. 22, [38] Ch. 38.

An articulation between the basioccipital lateral process and the transcapular ligament can be either absent (state 0; [28: fig 26A]), or present (state 1; [28: fig 26B]). Most Loricariini and *Aposturisoma myriodon*, *Farlowella knerii*, *Harttia punctata*, *H*. *rombocephala*, *Harttiella crassicauda*, and *Ha*. *Longicauda* were observed to lack the suture. On the other hand, the Farlowellini and the Harttiini (except the taxa above) have a suture between the basioccipital and the transcapular ligament.

26. Basioccipital, lateral process, angle: (0) transversal; (1) anterolaterally oriented; (2) posterolaterally oriented. CI = 0.13. [36] Ch. 20 (modified).

Within most Harttiini and Farlowellini, a transversal orientation of the basioccipital lateral process (state 0; [28: fig 26B]) was observed as the generalized state; among most Loricariini, the process is anterolaterally oriented (state 1; [28: fig 3B]). A posterolaterally oriented process (state 2) occurs in *Loricariichthys*, *Farlowella acus*, *F*. *henriquei*, *F*. *isbruckeri*, *F*. *jauruensis*, *F*. *venezuelensis*, and across all *Sturisoma*.

27. Transcapular ligament, ventral expansion: (0) ventrally and posteriorly expanded; (1) only ventrally expanded. CI = 0.07. [37] Ch. 23.

An ossified transcapular ligament represents a synapomorphy of the Siluriformes [57]. Additionally, within Loricariidae a posteroventral expansion (state 0) was observed on most Harttiini and Farlowellini, or a simple ventral expansion (state 1) in Loricariini and few taxa of the above-mentioned groups, which could be related to different types of pectoral girdles.

28. Prootic, antero-lateral process: (0) absent; (1) present. CI = 0.50.

The prootic is located between the orbitosphenoid (anteriorly), the basioccipital and exoccipital (posteriorly), and the compound pterotic (laterally). This structure can either have (state 1), or lack (state 0) an anterolateral process. In *Harttia*, *Harttiella*, *Cteniloricaria*, *Lamontichthys*, *Pterosturisoma*, *Sturisoma lyra*, and Loricariini, the process is absent or very short (Fig 4A and 4B), while in *Aposturisoma*, *Sturisomatichthys*, *Farlowella*, and the remaining *Sturisoma* species, the process is present (Fig 4C).

29. Hyomandibula, articulation with cranium, contribution of compound pterotic: (0) extended, same length as prootic; (1) reduced, shorter than prootic; (2) reduced or absent, articulation almost exclusively with prootic. CI = 0.22. [11] Ch. 34, [28] Ch. 44, [36] Ch. 36, [37] Ch. 65, [38] Ch. 30, [58] Ch. 4 (modified).

The comparison of the contribution of each bone to the articulation is made relative to prootic length, as shown by Fichberg [37], contrary to other authors (see references above). The hyomandibula has the same length as the prootic (state 0) in *Farlowella*, *Harttiella longicauda*, *Lamontichthys avacanoeiro*, *Sturisoma*, *Sturisomatichthys*, and some outgroups; reduced, shorter than prootic (state 1) in Harttiini (except *Harttiella longicauda*), *Sturisoma barbatum*, the remaining *Lamontichthys*, and most Loricariini; or reduced or absent, in which the articulation was almost exclusive with the prootic (state 2), in *Pterosturisoma*.

30. Compound pterotic, size of perforations: (0) small; (1) large. CI = 0.05. [28] Ch. 20.

The compound pterotic is related to swimbladder hydrostatic communication in loricariids, with its perforations allowing a direct contact of the latter to the environment. Perforations in the bone surface can be either small, as simple needle punctures (state 0), as seen in outgroups and a few *Farlowella*, *Sturisoma*, and *Sturisomatichthys*, or clearly larger (state 1; [76: fig 9]), as in most Harttiini, Farlowellini, and Loricariini.

31. Compound pterotic, junction of hyomandibula: (0) large contribution, almost same as contribution of prootic; (1) reduced contribution, less than half that of prootic. CI = 0.06. [28] Ch. 44, [36] Ch. 36 (modified).

There is variation regarding the extent of the contact between the hyomandibula and the compound pterotic, which can be either large, almost the same as the prootic (state 0; [38: fig 33]) mainly in *Farlowella*, some other Farlowellini and Harttiini, or reduced, being smaller than half that of the prootic (state 1) mainly in Loricariini.

### Sensory canals

32. Infraorbitals, passage of preopercular canal: (0) canal passing directly from sphenotic to preopercle; (1) canal passing through infraorbital five; (2) canal passing through infraorbital six. CI = 0.16. [28] Ch. 163, [36] Ch. 119.

The preopercular canal usually passes directly from the sphenotic to the preopercle in most loricariids, without entering any of the infraorbitals (state 0). In *Acestridium scutatum*, *Farlowella oxyrryncha*, *F*. aff. *amazonum*, *Sturisoma* aff. *tenuirostre*, and *St*. *graffini* it can pass through the fifth infraorbital between the sphenotic and the preopercle (state 1), or pass through the sixth infraorbital (state 2), as observed in *Dasyloricaria filamentosa*, *Farlowella hasemani*, *F*. *isbruckeri*, *F*. *paraguayensis*, *F*. *smithi*, *Harttia dissidens*, *H*. *punctata*, *Harttiella*, and *Lamontichthys parakana*.

33. Preopercle, sensory canal shape: (0) straight with two exits; (1) curved but not branched, two exits; (2) curved and branched, three exits; (3) straight and branched, three exits; (4) sensory canal absent. CI = 0.10. [28] Ch. 54, [36] Ch. 120.

Great diversity in shapes of the sensory canal of the preopercle was detected. Throughout Loricariidae, the canal can be either straight (state 0) in some Loricariini, *Farlowella*, *Sturisoma*, and most outgroups, curved but not branched (state 1) in most *Farlowella* and *Sturisomatichthys*, and a few *Harttia* and Loricariini, curved and branched (state 2) in *Cteniloricaria*, most *Harttia*, and *Lamontichthys llanero*, straight and branched, with three exits (state 3) in *Farlowella paraguayensis* and *Lamontichthys*, except *L*. *llanero*, or absent (state 4) in *Pseudohemiodon lamina*.

34. Canal-bearing cheek plate, position on head: (0) lateral; (1) ventrolateral; (2) ventral. CI = 0.40. [36] Ch. 117, [38] Ch. 34, [59] (modified).

Schaefer [59] described the canal-bearing cheek plate as being an element of the loricariids which appears to be homologous with a dermal cheek plate, rather than the interopercle. The author goes on and states that the second of two preopercle canal exits passes into and forms a canal in what appears to be a dermal cheek plate [59] (see his Figs 3C and 4). It was found that the ventral position (state 2) of the plate is a synapomorphy of the Harttiini and the Farlowellini, with a reversal for state 1 in *Sturisomatichthys citurensis* and *S*. *tamanae*, which have a ventrolateral plate.

35. Canal-bearing cheek plate, medial process: (0) absent; (1) short and broad; (2) long and broad; (3) long and thin. CI = 0.16.

The canal-bearing cheek plate of the Loricariinae can have a medial process in the ventral surface of the head, with different shapes. The process can be absent (state 0; Fig 6A) in *Farlowella*, except *F*. aff. *amazonum*, some *Sturisoma* and *Sturisomatichthys*, and most Loricariini and outgroups, or have a broad process (state 1; Fig 6B and 6C) as observed in *Cteniloricaria*, *Harttia*, *Lamontichthys*, most *Sturisoma*, and most *Sturisomatichthys*, or even a thin, pointed process (state 2; Fig 6D) as in *Harttiella*. The latter was found to be a synapomorphy of *Harttiella*.

36. Canal-bearing cheek plate, orientation of canal: (0) from posterolateral to anterolateral portion; (1) from dorsal to ventral border. CI = 0.25. [28] Ch. 161, [37] Ch. 47, [38] Ch. 35, [59] (modified).

The orientation of the sensory canal in the canal-bearing cheek plate can be from the posterolateral to the anterolateral portion of the plate (state 0; Fig 6B and 6C) in most loricariids, or from the dorsal to the ventral border of the plate (state 1; Fig 6A and 6D) in *Farlowella henriquei*, *F*. *rugosa*, *Harttiella*, and *Sturisomatichthys panamensis*. Points of origin and terminus of the cheek plate canal were coded differently from previous authors (see references above), who used the states as ventrally, laterally, or dorsally oriented.

37. Posteriormost infraorbital, canal: (0) one exit, dorsally oriented; (1) two exits, ventrolateral and dorsal. CI = 0.14. [36] Ch. 121.

The generalized condition observed within Loricariinae was that of one dorsally oriented exit (state 0). Nevertheless, a canal with two exits, laterodorsal and dorsal (state 1) was observed in *Farlowella reticulata*, *F*. *vittata*, *Rineloricaria lanceolata*, *Sturisoma guentheri*, *St*. *lyra*, *St*. *robustum*, and *Sturisomatichthys kneri*.

38. Parietal branch, terminal exit: (0) on frontal; (1) on frontal/supraoccipital border; (2) on frontal/sphenotic border; (3) on supraoccipital; (4) on sphenotic. CI = 0.37. [28] Ch. 174.

The parietal branch possesses a great variability regarding its terminal exit, which can be located on the frontal (state 0) in *Hemipsilichthys gobio*, the Hypoptopomatinae, and *Harttia loricariformis*, at the frontal/supraoccipital border (state 1) in *Hemiodontichthys acipenserinus*, at the frontal/sphenotic border (state 2) in *Farlowella acus*, *F*. *amazonum*, *Harttia fowleri*, *Sturisomatichthys festivus*, and hypostomine outgroups, on the supraoccipital (state 3) in *Dasyloricaria filamentosa*, *Rineloricaria*, and some Harttiini and Farlowellini, or on the sphenotic (state 4) in most *Harttia* and *Sturisomatichthys*.

39. Parietal branch, shape: (0) straight; (1) curved; (2) sinuous. CI = 0.18. [28] Ch. 172, [37] Ch. 58.

Similarly as its terminal exit, the shape of the parietal branch is variable, being either straight (state 0) in a few Farlowellini, most Loricariini, and outgroups, curved once (state 1), the generalized condition among Harttiini and Farlowellini, or curved more than one time and becoming sinuous (state 2), as observed in *Ancistrus brevipinnis* and *Pterygoplichthys lituratus*.

40. Lateral line, length: (0) truncated, ending one to several plates before supracaudal plates; (1) complete, up to last plate of caudal peduncle; (2) extended, lateral line ending on one a supracaudal plate. CI = 0.08. [27] Ch. 45, [28] Ch. 138, [36] Ch. 98.

The extension of the lateral line varies among the Loricariidae examined, being truncated, ending one to several plates before the supracaudal plates (state 0) in outgroups of the Hypoptopomatinae and Hypostominae, complete, reaching up to last caudal peduncle plate (state 1; [38: fig 35B]) in *Pterosturisoma*, *Lamontichthys*, a few *Farlowella* and some *Sturisomatichthys*, or extended, entering one supracaudal plate (state 2; [38: fig 35A]) as the generalized condition among most Harttiini, Loricariini, and Farlowellini.

### Maxilla and mandible

41. Autopalatine, posterior process, length: (0) extending beyond anterior condyle of lateral ethmoid; (1) not extending beyond anterior condyle of lateral ethmoid; (2) posterior process absent. CI = 0.09. [38] Ch. 18 (modified).

Within Harttiini, the autopalatine of *Harttiella* has a long posterior process, extending beyond the anterior condyle of the lateral ethmoid, the same occurring in some Loricariini (state 0). *Cteniloricaria* and *Harttia* have a short posterior process, not extending beyond the anterior condyle of the lateral ethmoid (state 1; Fig 7C), although it is not an exclusive synapomorphy of any of the genera. An absence of such process (state 2) was observed in some Loricariini, as well as representatives of the outgroup.

42. Autopalatine, lateral flange: (0) present and incomplete; (1) present and complete; (2) lateral flange absent. CI = 0.10. [38] Ch. 19.

For most *Sturisoma* species, except *St*. *barbatum*, *St*. *monopelte*, and *St*. *nigrirostrum*, an incomplete lateral flange in the autopalatine (state 0; [36: fig 11]) was observed. In most Loricariini, a complete lateral flange (state 1; [28: fig 10A]) is present in the autopalatine. The absence of that flange (state 2; [28: fig 10D]) was observed in *Sturisomatichthys*, except *S*. *kneri* and *S*. *panamensis*, and *Farlowella* except *F*. *henriquei*, *F*. *jauruensis*, *F*. *oxyrryncha*, *F*. *paraguayensis*, and *F*. *smithi*.

43. Autopalatine, anterior process: (0) absent; (1) present. CI = 0.16. [28] Ch. 26, [36] Ch. 23, [37] Ch. 68.

The presence of the autopalatine anterior process (state 1; [28: fig 10C]) was generally observed among members of the Harttiini and Farlowellini, except *Aposturisoma*, *Lamontichthys avacanoeiro*, *L*. *parakana*, *Farlowella acus*, and *F*. *paraguayensis*. Absence of such process (state 0; [28: fig 10A]) is the general condition among the Loricariini and the taxa named above.

44. Autopalatine, shape: (0) rod-like, irregular; (1) rectangular, straight. CI = 0.14. [7] Ch. 18, [21, 22], [28] Ch. 23, [36] Ch. 22 (modified).

Previous authors included both the shape of the autopalatine and extension of the process in the same character. Because they do not co-vary, these features were treated as two separate characters in the present study. The autopalatine can be rod-like, irregular (state 0; Fig 7) as the generalized condition among Harttiini, Farlowellini and, within *Sturisomatichthys*, in *S*. *dariensis*, *S*. *tamanae*, and *S*. *reinae*, or rectangular, straight (state 1), invariably in Loricariini and the remaining *Sturisomatichthys*.

45. Autopalatine, palatal splint: (0) absent; (1) present. CI = 0.07. [28] Ch. 24, [37] Ch. 73, [38] Ch. 20, [41].

The palatal splint is a thin, thread-like bone homologous with the lacrimal-antorbital of other catfishes, which articulates with the premaxilla anteriorly and is ligamentously connected to the soft tissues in the anterior rim of the nares. It can be absent (state 0) in Loricariini and most Farlowellini, or present (state 1; Fig 7A) in Harttiini, except *Harttia duriventris*, *H*. *fowleri*, and *H*. *longipinna*, *Lamontichthys*, except *L*. *llanero*, and *Sturisoma lyra*, *Sturisomatichthys aureus*, *S*. *festivus*, and *S*. *reinae*. For an extensive discussion of its homology, refer to Schaefer [41, 60, 61].

46. Premaxilla, cup-shaped portion, length: (0) similar to cup-shaped portion of dentary; (1) distinctly longer than cup-shaped portion of dentary; (2) distinctly shorter than cup-shaped portion of dentary. CI = 0.18. [38] Ch. 22 (modified).

The cup-shaped portion of the premaxilla is herein compared to the cup-shaped portion of the dentary, while Paixão and Toledo-Piza [38] compared the length to the width of the cup-shaped portion of the premaxilla itself. The cup-shaped portion was observed to be similar to that on the dentary (state 0) in outgroups, the Harttiini, *Lamontichthys*, *Aposturisoma*, *Farlowella schreitmuelleri*, *F*. *vittata*, *Sturisoma guentheri*, *St*. *monopelte*, *S*. *robustum*, *Sturisomatichthys aureus*, *S*. *leightoni*, and *S*. *varii*, distinctly longer than cup-shaped portion of dentary (state 1) in remaining *Farlowella*, *Sturisoma*, and *Sturisomatichthys*, or distinctly shorter than cup-shaped portion of dentary (state 2) as the generalized condition in Loricariini.

47. Dentary, coronoid process: (0) large, with small developed area; (1) large, with large developed area; (2) small; (3) absent. CI = 0.17. [38] Ch. 23, [39] Ch. 36 (modified).

**Fig 8:**
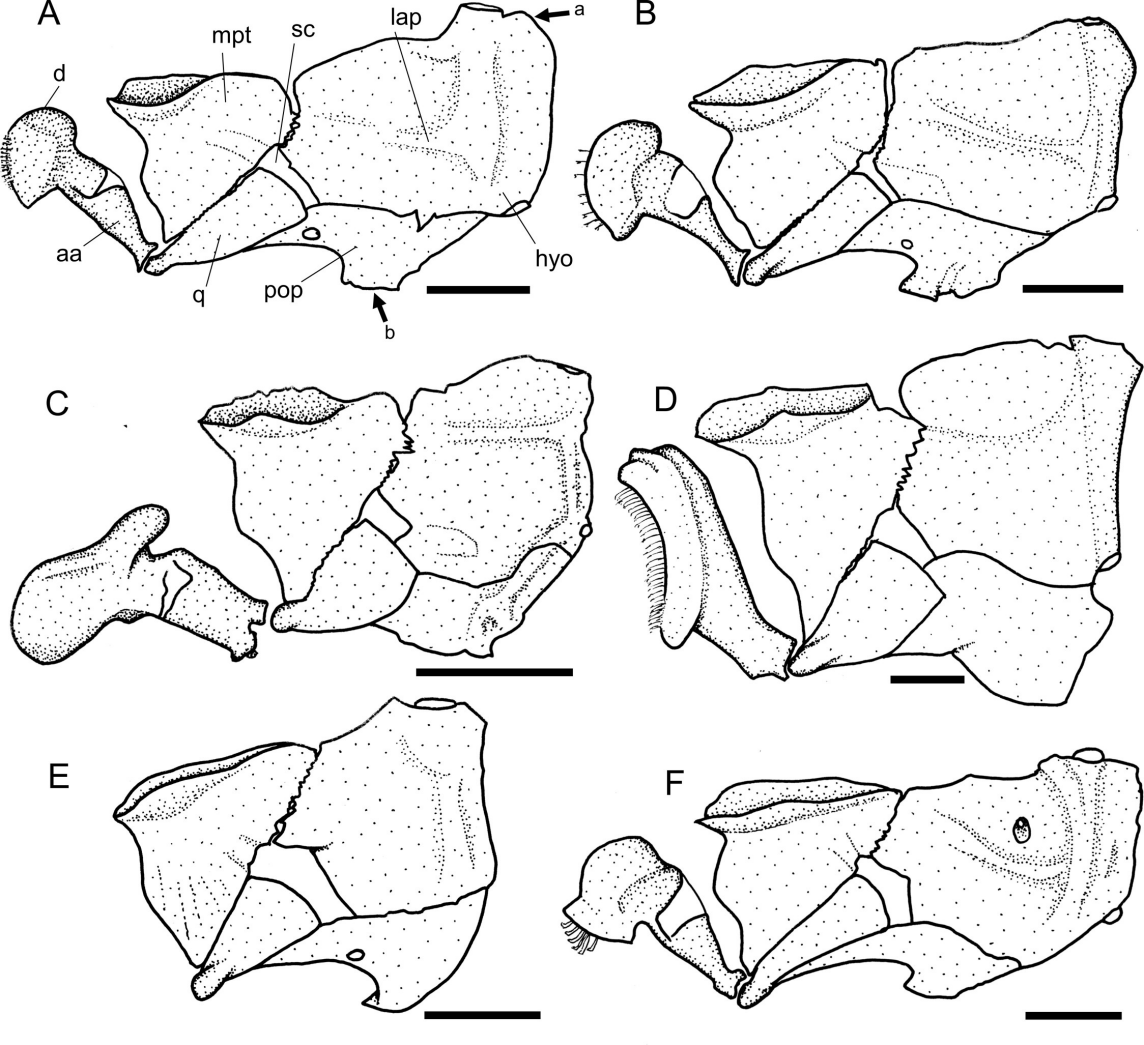
Suspensorium and mandible in lateral view. **A.** *Sturisoma robustum*, MCP 15812; **B.** *Sturisomatichthys reinae*, NRM 15155; **C.** *Harttiella crassicauda*, AUM 50837; **D.** *Harttia gracilis*, MZUSP 99678; **E.** *Pterosturisoma microps*, MZUSP 79909; **F.** *Farlowella mariaelenae*, USNM 349392. **Abbreviations:** aa = anguloarticular; d = dentary; hyo = hyomandibula; lap = *levator arcus palatine* crest; mpt = metapterygoid; pop = preopercle; q = quadrate; sc = symphiseal cartilage. Arrows: see trext. Scale bar 2 mm.

The dentary has a dorsal coronoid process which can be variable among loricariids, and serves for insertion of the *adductor* muscles [38, 41]. *Harttia* invariably has a large coronoid process, with small developed area (state 0; [38: fig 31A]) although not exclusive for the genus. *Harttiella*, *Lamontichthys*, *Cteniloricaria*, *Pterosturisoma*, and *Farlowella* have a large coronoid process, with large developed area (state 1; [38: fig 30]), except in *F*. *oxyrryncha*, *F*. *rugosa*, and *F*. *venezuelensis*. Among members of *Sturisoma*, *Sturisomatichthys*, and the Loricariini a small coronoid process (state 2; Fig 8A) or its absence (state 3; Fig 8B) were observed.

48. Dentary, posteroventral lamina: (0) present; (1) absent. CI = 0.25. [38] Ch. 24.

The dentary of loricariids usually has a long posteroventral lamina of bone that overlies the posterior face of the anguloarticular [38, 41]. In Harttiini and Farlowellini the lamina is invariably present (state 0; [38: fig 31]); in members of the Loricariini it is absent (state 1), as already described by Paixão and Toledo-Piza [38].

49. Dentary, posteroventral lamina, process: (0) present, relatively distant from main body of dentary; (1) present, close to main body of dentary; (2) absent or very narrow. CI = 0.12. [38] Ch. 25 (modified).

The dentary of loricariids is composed of the cup-shaped main body, which contain the teeth, and a posteroventral lamina, which articulates with the anguloarticular. The posteroventral lamina of the dentary usually has a bony process that can be close or distant from the main body. *Farlowella* invariably has a process relatively distant from the main body of dentary (state 0), while a process close to the main body of dentary (state 1) was observed in several taxa of Harttiini and Farlowellini. Absence of such a process, or a narrow, almost vestigial one (state 2) was observed in the Loricariini.

50. Dentary, teeth: (0) more than 20 teeth; (1) less than 20 teeth; (-) inapplicable. CI = 0.50. [37] Ch. 169 (modified).

For the Harttiini and Farlowellini, more than 20 dentary teeth (state 0) is the generalized condition; this corroborates observations of previous authors while defining groups within Loricariinae [19, 21, 22, 25, 62–66] as a phylogenetically informative character to diagnose these groups from the Loricariini. The latter was observed to have invariably fewer than 20 dentary teeth (state 1); although for *Hemiodontichthys*, this character is inapplicable due to the absence or vestigial state of the dentary bone, and thus, absence of teeth.

51. Tooth cusps, shape: (0) slightly rounded, oval; (1) rounded; (2) pointed; (-) inapplicable. CI = 0.11. [28] Ch. 184, [37] Ch. 143 (modified).

For Harttiini and *Sturisoma*, except *St*. *rostratum*, *St*. *robustum*, *St*. aff. *tenuirostre* and *St*. *graffini*, the generalized state is that of teeth with the longer cusp straight (state 0). For *Aposturisoma*, *Lamontichthys*, *Pterosturisoma*, *Farlowella acus*, *F*. *venezuelensis*, and *F*. *vittata*, the above-mentioned *Sturisoma* species, *Sturisomatichthys* except *S*. *dariensis*, *S*. *frenatus* and *S*. *kneri*, rounded cusps (state 1) were observed. For the other species of *Farlowella*, slightly pointed cusps are present (state 2), while in the Loricariini there are strongly pointed teeth (state 3). For *Hemiodontichthys* this character is inapplicable.

52. Dentary teeth, cusp size: (0) cusps of same size or approximately same size; (1) inner cusp slightly longer than outer; (2) inner cusp distinctly longer than outer; (3) only inner cusp present; (-) inapplicable. CI = 0.12. [37] Ch. 145 (modified).

The relative cusp size of dentary teeth is variable across genera examined. Cusps were observed to have the same size or approximately same size (state 0) in *Hemipsilichthys gobio*, most *Harttia*, *Sturisomatichthys*, *Aposturisoma myriodon*; the inner cusp slightly longer than outer (state 1) in some outgroups, *Harttiella*, *Lamontichthys*, *Sturisoma*, *Farlowella oxyrryncha*, *F*. aff. *amazonum*, *F*. *schreitmuelleri*, *F*. *smithi*, *F*. *venezuelensis*, *Sturisomatichthys citurensis*, a few *Harttia*, and *Pseudohemiodon lamina*; inner cusp distinctly longer than outer (state 2) in remaining *Farlowella*, *Sturisomatichthys dariensis*, *S*. *panamensis*, and most Loricariini; only inner cusp present (state 3) in *Spatuloricaria puganensis* and *Chaetostoma breve*. The remaining taxa were coded as innaplicable (-) due to the absence or poorly developed teeth.

53. Dentary, size: (0) approximately same size as anguloarticular; (1) dentary almost twice the size of anguloarticular; (2) dentary half or a little less than half the size of anguloarticular. CI = 0.28. [39] Ch. 38 (modified).

The dentary in examined members of Loricariidae can have almost the same size as the anguloarticular (state 0) in Delturinae, Hypoptopomatinae, *Sturisoma* except *St*. *guentheri*, and *Lamontichthys llanero*, or it can be almost twice the size (state 1) in other outgroups, *Aposturisoma*, *Cteniloricaria*, *Harttia*, *Sturisomatichthys*, *St*. *guentheri*, and remaining *Lamontichthys*, or even half or a little less than half the size of the anguloarticular (state 2) as the generalized condition in Loricariini.

54. Premaxilla, tooth number: (0) more than 20; (1) fewer than 20. CI = 0.25.

In Loricariinae, the Harttiini and the Farlowellini are characterized by having more than 20 teeth on each premaxilla (state 0). The Loricariini have fewer than 20 teeth (state 1).

55. Premaxilla, tooth shape: (0) robust, relatively short and wide, straight; (1) delicate, long and narrow, tooth cusp forming angle of approximately 45° with longer axis of tooth; (-) inapplicable. CI = 0.50. [38] Ch. 26 (modified).

In the Loricariini and other subfamilies examined robust teeth (state 0) was the generalized condition, while in the Harttiini and Farlowellini delicate teeth (state 1) was invariably observed. For *Hemiodontichthys* this character is inapplicable.

56. Premaxilla, tooth, cusp size: (0) almost equal; (1) inner cusp visibly larger than outer; (-) inapplicable. CI = 0.14. [23], [28] Ch. 184, [37] Ch. 168, [41] (modified).

Previous authors included conical and symmetric teeth. Considering the taxa included herein, only three character-states were found. Cusp subequal in size (state 0) was the generalized condition in Harttiini and Farlowellini, and a few Loricariini, while inner cusp visibly larger than the outer (state 1) on most Loricariini and outgroups.

57. Premaxilla, shape: (0) thick, quadrangular; (1) bony lamina; (2) vestigial. CI = 0.40. [28] Ch. 28, [36] Ch. 25, [39] Ch. 31 (modified).

**Fig 9:**
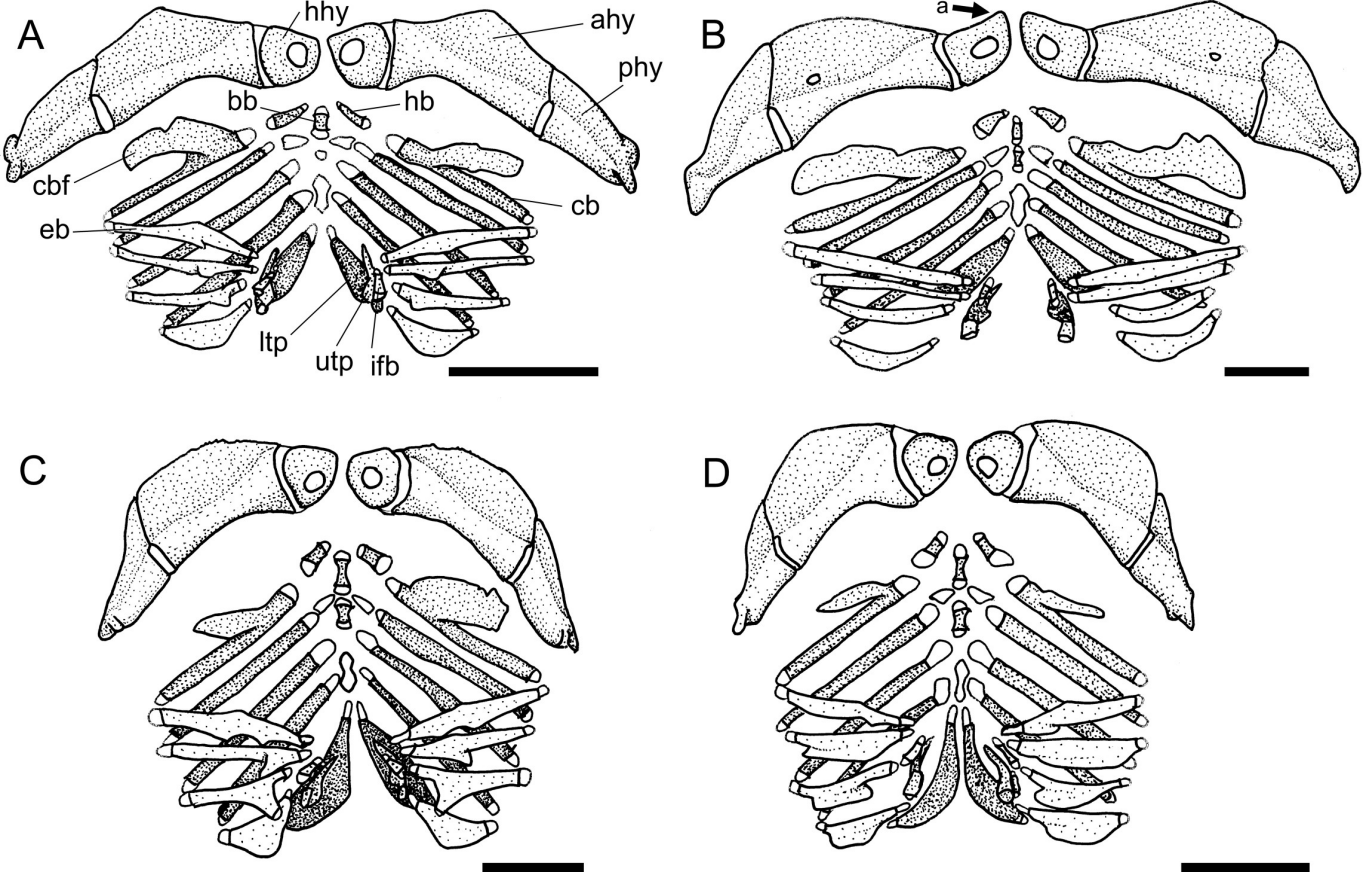
Hyoid and branchial arches in ventral view. **A.** *Harttiella crassicauda*, AUM 50837; **B.** *Harttia gracilis*, MZUSP 99678; **C.** *Sturisomatichthys reinae*, NRM 15155; **D.** *Farlowella mariaelenae*, USNM 349392. **Abbreviations:** ahy = anterohyal; bb = basibranchial; cb = ceratobranchial; cbf = ceratobranchial flange; eb = epibranchial; hb = hypobranchial; hhy = hypohyal; ifb = infrapharyngobranchial; ltp = lower pharyngeal tooth plate; phy = posterohyal; utp = upper pharyngeal tooth plate. Arrow: see text. Scale bar 2 mm.

Harttiini and Farlowellini, as well as *Rineloricaria*, invariably have a thick, quadrangular or cup-shaped premaxilla (state 0; [28: fig 9C, 9D]). Most Loricariini have the premaxillary cup reduced to a thin lamina of bone (state 1; [28: fig 11C]), or very reduced, vestigial (state 2; [28: fig 11E]).

58. Premaxilla, cup-shaped region, length: (0) two to three times longer than wide; (1) length and width equivalent; (-) inapplicable. CI = 0.50. [38] Ch. 21 (modified).

In Harttiini and Farlowellini the length of the cup-shaped region of the premaxilla is two to three times longer than wide (state 0), while in the Loricariini length and width are invariably equivalent (state 1). This character is inapplicable for *Hemiodontichthys*.

59. Premaxilla, size: (0) same size as autopalatine; (1) larger than autopalatine; (2) smaller than autopalatine. CI = 0.16.

**Fig 10:**
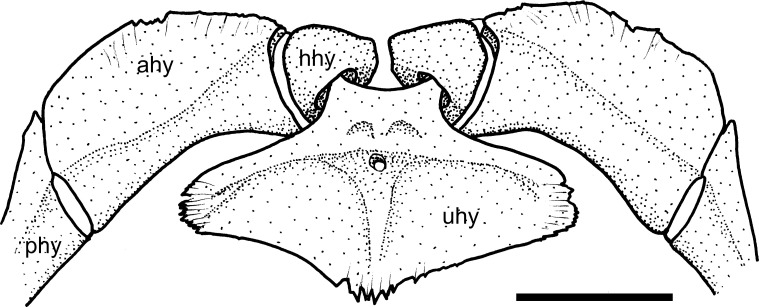
Hyoid arch in ventral view. *Sturisomatichthys reinae*, NRM 15155. **Abbreviations:** ahy = anterohyal; hhy = hypohyal; phy = posterohyal; uhy = urohyal. Scale bar 2 mm.

**Fig 11:**
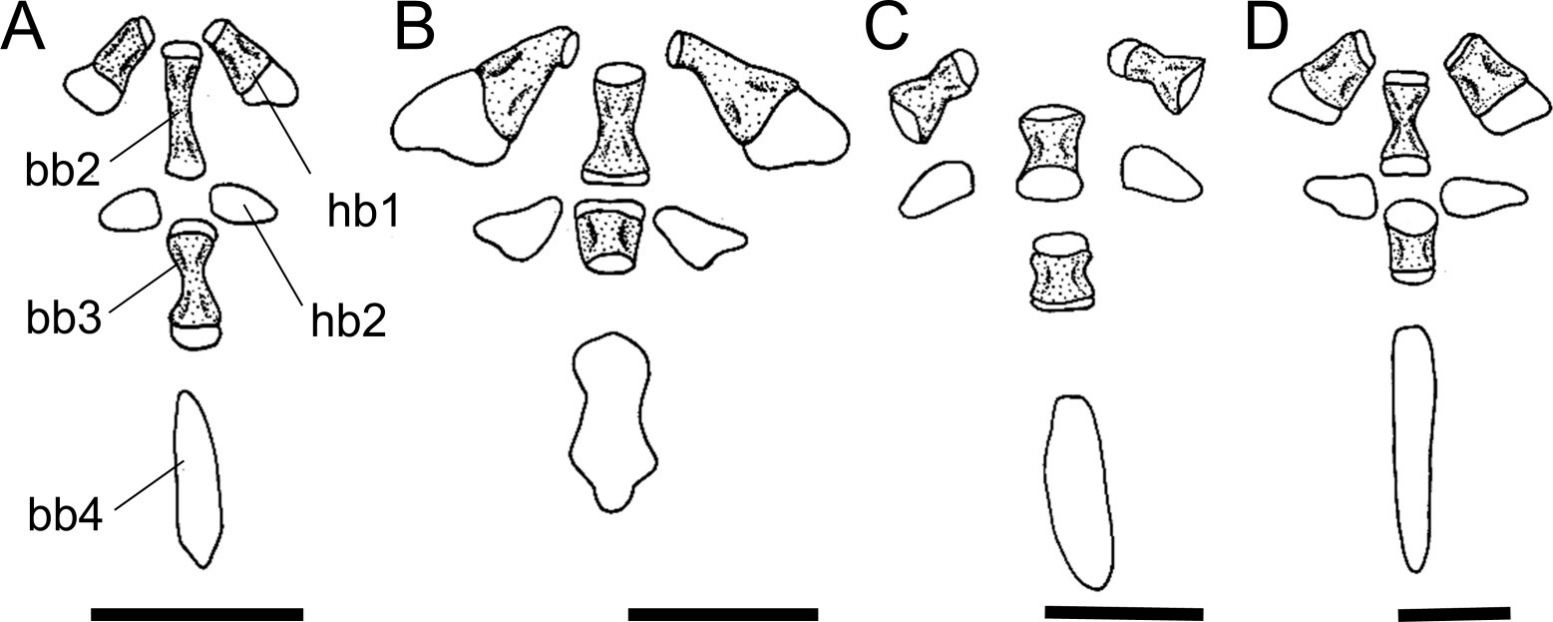
Basibranchials in dorsal view. **A.** *Farlowella henriquei*, MCP 41992; **B.** *Harttia guianensis*, MHNG 2643.033; **C.** *Sturisomatichthys panamensis*, USNM 316293; **D.** *Sturisoma robustum*, MCP 15812. **Abbreviations:** bb = basibranchial; hb = hypobranchial. Scale bar 2 mm.

A premaxilla the same size as the autopalatine (state 0; [28: fig 9A]), or larger, (state 1; [28: fig 11D]) was present in several taxa among the groups studied. On the other hand, in *Aposturisoma*, *Farlowella*, *Sturisoma*, and *Sturisomatichthys*, except *S*. *tamanae*, the premaxilla is smaller compared to the autopalatine (state 2; [28: fig 10D]).

60. Maxilla, size: (0) slightly longer than autopalatine; (1) same size as autopalatine; (2) shorter than autopalatine. CI = 0.07. [28] Ch. 31, [39] Ch. 33.

The development of the maxilla varies among members of the Loricariidae, and it can be related to the development of the autopalatine. Across taxa examined here, the maxilla was found to be slightly longer (state 0) in some outgroups, *Harttia fluminensis*, *H*. *fowleri*, *H*. *gracilis*, and *H*. *rhombocephala*, same size (state 1) in *Lamontichthys*, *Pterosturisoma*, *Loricariichthys*, and some Harttiini and Farlowellini, or smaller than the autopalatine (state 2) in most Harttiini, Farlowellini, and Loricariini.

61. Maxilla, posterolateral flange, width: (0) wider than autopalatine; (1) similar to autopalatine. CI = 0.33. [37] Ch. 74.

The generalized condition for the Loricariinae is having a posterolateral flange of the maxilla similar to the dorsoventral thickness of the autopalatine (state 1), as found in most taxa examined. A maxillary posteroventral flange wider than the autopalatine dorsoventral thickness (state 0) was observed in the outgroups *Hemipsilichthys gobio*, *Ancistrus brevipinnis*, *Chaetostoma breve*, and *Pareiorhaphis calmoni*.

### Suspensorium

62. Metapterygoid, canal shape: (0) shallow, inconspicuous; (1) deep, partially covered by bony shelf; (2) deep, totally covered by bony shelf. CI = 0.08. [27] Ch. 15, [28] Ch. 36, [36] Ch. 29, [37] Ch. 70, [40], [41] Ch. 2 (modified).

As described by previous authors [41, 67, 68] a horizontal crest, can be present on the metapterygoid, which functions as insertion area for the *adductor mandibulae* muscle, and its development could be directly related to the development of that muscle. A correlation was found regarding the dentary size and the type of canal; since the canal is related to the insertion of the *adductor mandibulae* muscle, it could be related to the dentary size, and hence, feeding behavior. For the Loricariini, the generalized, but not exclusive condition is that of a shallow, inconspicuous canal (state 0; [28: fig 15B]); while for members of the Harttiini and the Farlowellini, both a deep, partially covered canal (state 1; [28: fig 15A]), and a deep, totally covered canal (state 2; [28: fig 15D]) were observed. For the Loricariini a weaker, smaller dentary is observed, and thicker and larger dentary are characteristic of the Harttiini and Farlowellini.

63. Metapterygoid, articulation to lateral ethmoid, condyle: (0) absent; (1) present. CI = 0.03. [39] Ch. 44.

The contact between the metapterygoid and the lateral ethmoid was already described above (see character 12). Nevertheless, the metapterygoid might lack (state 0) or have (state 1) a condyle as part of that contact. Contrary to what Provenzano [39] described, the condyle was not observed to be present in all members of *Farlowella* and Harttiini.

64. Metapterygoid, interdigitated suture to hyomandibula: (0) short, less than half the contact area between both structures; (1) long, more than half contact area between both structures. CI = 0.05. [27] Ch. 16, [28] Ch. 39, [36] Ch. 32, [69].

The presence of an interdigitated suture between the metapterygoid and the hyomandibula was demonstrated by previous authors [41, 69] to occur in loricariids. It was found that such suture can be either short (state 0; Fig 8A–8C) in Loricariini, except *Spatuloricaria puganensis*, in *Aposturisoma myriodon*, *Cteniloricaria*, most *Farlowella* and *Sturisoma*, *Harttia loricariformis*, *H*. *rhombocepahala*, *Sturisomatichthys reinae*, and *S*. *varii*; or long (state 1; Fig 8D–8F) related to the length of structures themselves, in *Farlowella hahni*, *F*. *hasemani*, *F*. *isbruckeri*, *F*. *oxyrryncha*, *F*. *paraguayensis*, *F*. *venezuelensis*, *F*. *vittata*, remaining *Harttia*, and remaining Farlowellini.

65. Metapterygoid, dorsal margin: (0) not expanded; (1) strongly expanded anteriorly. CI = 0.06. [28] Ch. 37 (modified).

Among species of *Harttia* the dorsal margin of the metapterygoid which carries the metapterygoid canal is strongly expanded anteriorly (state 1; Fig 8D). Such expansion is also observed among several species of the Loricariini.

66. Quadrate, shape in lateral view: (0) expanded anteriorly, roughly rectangular (1) narrow anteriorly, triangular. CI = 0.33. [39] Ch. 46 (modified).

In *Hemipsilichthys*, *Chaetostoma* and *Pareiorhaphis* the quadrate is expanded anteriorly, becoming roughly rectangular em lateral view (state 0). In most species examined, however, the quadrate is narrow anteriorly, becoming clearly triangular in lateral view (state 1; Fig 8A–8F).

67. Symphyseal cartilage, size: (0) long and thin, as deep as quadrate posterior border; (1) large and broad, as deep or deeper than quadrate posterior border; (2) short, half quadrate posterior border; (3) highly reduced or absent. CI = 0.25.

The symphyseal cartilage is variable in size. *Hemipsilichthys*, *Sturisoma* and *Sturisomatichthys* have a long and thin symphyseal cartilage, as deep as the posterior border of the quadrate (state 0; Fig 8A and 8B); some Hypoptopomatinae and Loricariini, *Farlowella*, and *Pterosturisoma* have a large and broad symphyseal cartilage, which can surpass quadrate height (state 1; Fig 8E and 8F). On the other hand, *Limatulichthys*, Harttiini and remaining Farlowellini invariably have a symphyseal cartilage short, small, half the quadrate height (state 2; Fig 8C and 8D). Finally, Hypostominae, *Hemiodontichthys acipenserinus*, and *Metaloricaria* have a highly reduced or absent symphyseal cartilage (state 3).

68. Hyomandibula, surface type: (0) large concavity area; (1) reduced concavity area. CI = 0.05. [27] Ch. 17, [28] Ch. 41, [36] Ch. 34 (modified).

Previous authors proposed intermediate states for the size of the concavity, but only two states were considered here. Taxa examined have a large concavity area (state 0) in a few outgroups, *Aposturisoma*, *Dasyloricaria*, most *Farlowella*, *Harttia fluminensis*, *H*. *fowleri*, *H*. *kronei*, *H*. *punctata*, *H*. *rhombocephala*, *Limatulichthys griseus*, *Loricaria lundbergi*, *Metaloricaria*, *Pterosturisoma*, *Rineloricaria*, *Spatuloricaria puganensis*, *Sturisoma*, *Sturisomatichthys* except *S*. *aureus*, and *S*. *leightoni*, or a reduced concavity (state 1) in *Cteniloricaria*, *Farlowella hahni*, *F*. *hasemani*, *F*. *henriquei*, *F*. *knerii*, *F*. *nattereri*, *F*. *rugosa*, and remaining *Harttia*, *Harttiella*, and *Lamontichthys*.

69. Hyomandibula, posterior extension: (0) absent; (1) present. CI = 0.16. [28] Ch. 40, [36] Ch. 38, [70] (modified).

The absence of an extension or process on the posterior portion of the hyomandibula is characteristic of *Harttia*, *Cteniloricaria*, *Lamontichthys*, *Pterosturisoma*, *Sturisomatichthys*, and the Loricariini (state 0; Fig 8B, 8D and 8E). Species examined of *Aposturisoma*, *Farlowella*, *Harttiella*, and *Sturisoma* possess such an extension (state 1; Fig 8A, 8C and 8F, arrow a).

70. Hyomandibula, articulation with neurocranium: (0) including sphenotic, prootic and compound pterotic; (2) including only sphenotic and prootic. CI = 0.07. [28] Ch. 43, [36] Ch. 35, [39] Ch. 52, [69, 70].

The extension of contact of the hyomandibula with the neurocranium can include the bones sphenotic, prootic, and compound pterotic (state 0) in *Aposturisoma*, *Cteniloricaria*, *Farlowella*, most *Harttia*, *Harttiella longicauda*, *Hemiodontichthys acipenserinus*, *Lamontichthys avacanoeiro*, *L*. *parakana*, *Limatulichthys griseus*, *Pterosturisoma*, and most *Sturisoma* and *Sturisomatichthys*, or the sphenotic and prootic only (state 1) in *Harttia dissidens*, *H*. *duriventris*, *H*. *gracilis*, *H*. *leiopleura*, *H*. *torrenticola*, *H*. *trombetensis*, *Harttiella crassicauda*, *Lamontichthys filamentosus*, *L*. *llanero*, *Sturisoma barbatum*, *St*. *guentheri*, *St*. *lyra*, *St*. aff. *tenuirostre*, *Sturisomatichthys citurensis*, *S*. *festivus*, *S*. *kneri*, *S*. *tamanae*, and remaining Loricariini.

71. Hyomandibula, *levator arcus palatine* crest: (0) present; (1) absent. CI = 0.04. [28] Ch. 40, [39] Ch. 50 (modified).

Previous authors included the size of the *levator arcus palatine* crest in this character. However, only presence of the crest (state 0) in a few outgroups, *Aposturisoma*, *Cteniloricaria*, *Farlowella amazonum*, *F*. *curtirostra*, *F*. *mariaelenae*, *F*. *oxyrryncha*, *Harttia garavelloi*, *H*. *kronei*, *H*. *novalimensis*, *Harttiella longicauda*, *Lamontichthys filamentosus*, *L*. *llanero*, *Loricaria lundbergi*, *Pterosturisoma*, *Sturisoma robustum*, *St*. *rostratum*, and most *Sturisomatichthys*; or absence (state 1) of the crest in remaining *Farlowella*, *Harttia*, *Harttiella*, and *Sturisoma*, *Sturisomatichthys festivus*, *S*. *frenatus*, *S*. *kneri*, *S*. *panamensis*, and remaining Loricariini, was found to be informative here.

72. Hyomandibula, posterior border, type of articulation with compound pterotic: (0) synchondral only; (1) sutural and synchondral. CI = 0.06. [28] Ch. 47, [39] Ch. 53.

The type of contact of the posterior border of the hyomandibula with the compound pterotic varied as purely synchondral (state 0) on Loricariini and most Hartiini, or sutural and synchondral (state 1) on most Farlowellini.

73. Preopercle, anterior process: (0) short, reaching only posterior border of quadrate; (1) long, reaching at least half of quadrate length; (2) process absent. CI = 0.22.

A short process (state 0) was observed in outgroups and some *Sturisomatichthys*. For the Harttiini and the remainder Farlowellini, a long process (state 1; Fig 8A–8F) is the general condition. *Sturisoma rostratum* and the Loricariini lack the process (state 2).

74. Preopercle, shape: (0) rectangular; (1) irregular, ventral border rounded; (2) inverted triangle, pointed ventral process; (3) thin lamina, occupying almost entire length of suspensorium base. CI = 0.37. [28] Ch. 50, [36] Ch. 41 (modified).

The shape of the preopercle varies among studied species, being rectangular (state 0; Fig 8D) in Delturinae, Hypoptopomatinae, *Aposturisoma*, *Harttia*, *Harttiella*, *Lamontichthys*, and *Pterosturisoma*; irregular, with the ventral border rounded (state 1; Fig 8A and 8F) in *Cteniloricaria*, *Farlowella*, *Metaloricaria*, *Sturisoma*, and *Sturisomatichthys*; an inverted triangle, with a pointed ventral process (state 2) in *Chaetostoma breve*, *Ancistrus brevipinnis*, and *Pseudohemiodon lamina*; or a thin lamina, occupying almost the entire length of the suspensorium base (state 3) in *Dasyloricaria*, *Hemiodontichthys acipenserinus*, *Limatulichthys griseus*, *Loricaria*, *Loricariichthys*, *Pterygoplichthys lituratus*, *Rineloricaria*, and *Spatuloricaria puganensis*. A higher diversity of shapes of the preopercle was found in this study. Previous studies proposed this character to show only elongate or robust shapes.

75. Preopercle, size: (0) deeper than quadrate; (1) as deep as quadrate; (2) shorter than quadrate. CI = 0.25.

As well as its shape, the size of the preopercle is highly variable in the groups examined. The outgroup, *Harttia*, *Lamontichthys*, and *Pterosturisoma* have the preopercle deeper than the quadrate (state 0; Fig 8D); in Farlowellini and Hypostominae examined the preopercle is as deep as the quadrate (state 1; Fig 8E); and in Loricariini of the preopercle is shorter that the quadrate (state 2; [76: fig 10B]).

76. Preopercle, ventral process: (0) absent; (1) present. CI = 0.50. [28] Ch. 53, [36] Ch. 43, [38] Ch. 32.

The ventral margin of the preopercle of most species examined lacks a ventral process (state 0). In both the Farlowellini and Harttiini, however, the preopercle has a broad ventral process (state 1; Fig 8A, arrow b). Rapp Py-Daniel [28] discussed the presence of a process on the preopercle as a synapomorphy of the Harttiini (her Fig 16A and 16B), and included the genera *Sturisomatichthys*, *Farlowella*, *Aposturisoma*, *Sturisoma* (her Farlowellina) and *Harttia*, *Cteniloricaria*, *Lamontichthys*, *Harttiella* and *Pterosturisoma* (her Harttiina) as members of the Harttiini. In fact, that is a synapomorphy of those genera within the Loricariinae, but is not useful to distinguish the Harttiini from the Farlowellini (following the classification proposed here).

77. Preopercle, exposed surface: (0) not exposed; (1) small area, up to 1/3 of second infraorbital; (2) large area, half or equal to second infraorbital. CI = 0.10. [28] Ch. 51, [36] Ch. 42, [38] Ch. 33.

Variable exposure of the preopercle occurs in the loricariids, the bone being not exposed on the surface (state 0) in most Hypoptopomatinae examined, most *Harttia*, *Lamontichthys*, *Pterosturisoma*, *Sturisoma robustum*, *St*. *rostratum*, *Sturisomatichthys aureus*, *S*. *dariensis*, *S*. *festivus*, and *S*. *frenatus*, partially exposed (state 1) in *Farlowella jauruensis*, *F*. *smithi*, *Hartia novalimensis*, *H*. *torrenticola*, *Harttiella*, *Sturisoma barbatum*, *St*. *graffini*, *Sturisomatichthys panamensis*, *S*. *reinae*, *S*. *varii*, *Loricariichthys*, *Rineloricaria*, *Dasyloricaria filamentosa*, *D*. *paucisquama*, *Hemiodontichthys acipenserinus*, *Limatulichthys griseus*, *Spatuloricaria puganensis*, *Acestridium scutatum*, and *Hisonotus laevior*, or largely exposed (state 2) in Hypostominae, remaining *Farlowella*, *Sturisoma*, and *Sturisomatichthys*, *Dasyloricaria latiura*, *Loricaria lundbergi*, and *Pseudohemiodon lamina*.

78. Preopercle, connection to dermal plates: (0) loosely connected to dermal plates; (1) partially sutured at dorsal ridge; (2) preopercle strongly sutured to dermal plates; (3) no contact. CI = 0.27. [28] Ch. 52 (modified).

The degree of connection of the preopercle with dermal plates was found to be either loosely connected (state 0) on *Farlowella*, *Harttia*, *Harttiella*, and most *Sturisomatichthys*, partially sutured (state 1) on *Lamontichthys avacanoeiro*, *L*. *parakana*, and *Pterosturisoma microps*, strongly sutured (state 2) on most Loricariini, or without connection to dermal plates (state 3) among most species of *Sturisoma* and some *Stursisomatichthys*.

79. Suspensorium, overall shape: (0) square; (1) rectangular. CI = 0.20. [37] Ch. 62 (modified).

The Harttiini, *Aposturisoma*, *Lamontichthys*, and *Pterosturisoma* have a square suspensorium (state 0; Fig 8C–8E), while *Farlowella*, *Sturisoma*, *Sturisomatichthys*, and some Loricariini have a rectangular and more elongated suspensorium (state 1; Fig 8A, 8B and 8F).

80. Infraorbital and supraorbital canals, point of bifurcation: (0) on sphenotic; (1) at limit between sphenotic and compound pterotic. CI = 0.12. [28] Ch. 166, [68].

For the Harttiini, except *Cteniloricaria*, the condition observed was the bifurcation of the infraorbital and supraorbital canals on the sphenotic (state 0). In *Sturisomatichthys*, *Farlowella*, and the Loricariini, except *Loricaria lundbergi*, the bifurcation occurs at the limit between the sphenotic and the compound pterotic (state 1). Both states were variable across remaining taxa.

### Hyoid and branchial arches

81. Anterohyal, anterior border, relative width of ventral laminar expansion: (0) widens gradually from medial to lateral portion; (1) widens abruptly in lateral portion. CI = 0.20.

Different degrees of widening were observed along the anterohyal which can widen gradually (state 0; [63: fig 7A]) as in most taxa examined, or widen abruptly (state 1; [63: fig 7B]) medial to lateral portion in *Farlowella acus*, *F*. *curtirostra*, *F*. *hasemani*, *F*. *henriquei*, and *Acestridium scutatum*.

82. Anterohyal, anterior margin, shape: (0) serrate on proximal and distal portions; (1) serrate on middle portion of bone; (2) serrate along entire margin; (3) margin smooth. CI = 0.20. [37] Ch. 80.

The anterohyal can have different parts of the anterior margin irregular, slightly serrated. This can be in the proximal and distal portions (state 0) in *Hemipsilichthys gobio*, *Neoplecostomus microps*, and *Pareiorhaphis calmoni*, in the middle portion (state 1; Fig 10) in *Hisonotus laevior*, *Parotocinclus maculicauda*, *Aposturisoma*, *Lamontichthys*, most *Sturisomatichthys*, and most *Sturisoma*, along its entire margin (state 2) in *Ancistrus brevipinnis*, *Farlowella*, *Pterosturisoma*, *Dasyloricaria*, *Limatulichthys griseus*, *Loricaria lundbergi*, *Loricariichthys*, *Pseudohemiodon lamina*, *Rineloricaria cadeae*, *R*. *quadrensis*, and *Spatuloricaria puganensis*, or the anterior margin of the anterohyal is regular and smooth (state 3) in *Chaetostoma breve*, *Pterygoplichthys lituratus*, *Acestridium scutatum*, Harttiini, *Sturisoma monopelte*, *St*. *robustum*, *Sturisomatichthys aureus*, and *Hemiodontichthys acipenserinus*.

83. Anterohyal, anterior margin, expansion: (0) greatly expanded; (1) slightly expanded; (2) not expanded. CI = 0.13. [28] Ch. 63, [37] Ch. 84, [41] Ch. 28.

The anterior margin of the anterohyal can be greatly expanded (state 0; Fig 9D) in Hypoptopomatinae, *Ancistrus brevipinnis*, *Aposturisoma*, most *Farlowella*, *Sturisomatichthys aureus*, and *Dasyloricaria latiura*, slightly expanded (state 1; Fig 9A–9C) in Harttiini, remaining *Sturisomatichthys* and *Dasyloricaria*, and *Loricaria lundbergi*, or not expanded at all (state 2) in remaining outgroups, most Loricariini, *Lamontichthys*, *Sturisoma*, *Farlowella oxyrryncha*, *F*. *rugosa*, *F*. *schreitmuelleri*, and *F*. *smithi*.

84. Anterohyal, connection to hypohyals: (0) synchondral and sutural; (1) only synchondral. CI = 0.06. [28] Ch. 62, [36] Ch. 47, [37] Ch. 83, [70].

The anterohyal can be connected to the hypohyals via both a suture and a synchondral joint (state 0; Fig 9B) in *Hemipsilichthy gobio*, *Ancistrus brevipinnis*, most Hypoptopomatinae, *Cteniloricaria*, *Harttia dissidens*, *H*. *fowleri*, *H*. *guianensis*, *H*. *puncata*, *H*. *rhombocephala*, *Lamontichthys llanero*, *Sturisoma nigrirostrum*, *Sturisomatichthys citurensis*, *S*. *kneri*, *S*. *leightoni*, and *Loricariichthys*, or via a synchondral joint only (state 1; Fig 9A, 9C and 9D) in other outgroups, *Aposturisoma myriodon*, *Farlowella*, *Pterosturisoma*, and remaining *Harttia*, *Lamontichthys*, *Sturisoma*, *Sturisomatichthys*, and Loricariini.

85. Hypohyal, anterior projection: (0) absent; (1) present. CI = 0.20.

The hypohyal is most commonly rounded or straight anteriorly (state 0) as in all outgroups, Loricariini, and Farlowellini. In *Ctenioloricaria*, some *Harttia* and *Hartiella* the hypohyal has an anterior projection (state 1; Fig 9B, arrow a).

86. Urohyal, shape, dorsal view: (0) triangular with convex sides; (1) elliptical; (2) rectangular; (3) equilateral triangle. CI = 0.42. [37] Ch. 77 (modified).

The urohyal is a median, single bone at the base of the hyobranchial apparatus, and is connected anteriorly to the hypohyals. It is variable in shape among examined species, and can be triangular with convex sides (state 0) in *Hemipsilichthys gobio*, Hypoptopomatinae, and Hypostominae, elliptical (state 1; Fig 10) in *Farlowella*, *Sturisomatichthys*, and *Sturisoma nigrirostrum*, rectangular (state 2) in Harttiini and *Aposturisoma*, or a equilateral triangle (state 3) in Loricariini and remaining Farlowellini. Fichberg [37] proposed more diverse shapes for the urohyal. However, fewer states were detected here.

87. Posterohyal, dorsal hook: (0) large, projected; (1) short, not projected. CI = 0.12. [28] Ch. 61, [36] Ch. 46, [37] Ch. 82.

The posterohyal is located laterally to the anterohyal, partially separated by a cartilage. A dorsal process was observed on the hypohyal lateral tip, which varied in size. For the Loricariinae the generalized condition is that of a short, not projected hook (state 0). Nevertheless, for some Loricariini such as *Loricaria lundbergi*, *Pseudohemiodon lamina*, *Loricariichthys anus*, and *Dasyloricaria*, a large, projected hook was observed (state 1).

88. Hypobranchial 1, shape: (0) hourglass-like; (1) fan-like; (2) squarish. CI = 0.28. [7] Ch. 147, [28] Ch. 71, [36] Ch. 51; [38] Ch. 12 (modified).

Hypobranchial 1 is located posterior to hypohyal and laterally to basibranchial. Only the hypobranchial 1 is ossified in the Loricariidae. In outgroup taxa and *Harttia garavelloi*, and *Sturisomatichthys panamensis* an hourglass-shaped hypobranchial 1 was observed (state 0; Fig 11C). Fan-like hypobranchial 1 (state 1; Fig 11B) is present in *Cteniloricaria* and some *Harttia* within the Harttiini, in addition to *Loricariichthys*, *Loricaria lundbergi*, and *Limatulichthys griseus*. A squarish hypobranchial 1 (state 2) was the most common state observed within the Loricariinae (11A, D).

89. Basibranchials, ossification, number: (0) one; (1) two. CI = 0.25. [7], [28] Ch. 64, [36] Ch. 48.

Basibranchial 1 is lacking in all catfishes and loricariids have basibranchials 2, 3 and 4. The degree of ossification also varies across the family, but basibranchial 2 is always ossified [16]. It was found that either only basibranchial 2 is ossified (state 0; Fig 9A) in *Hemipsilichthys gobio*, Hypoptopomatinae, Hypostominae, *Harttia kronei*, *Harttiella*, and *Hemiodontichthys acipenserinus*, or basibranchials 2 and 3 are ossified (state 1; Fig 9B–9D) in Farlowellini, and remaining Harttiini, Loricariini and outgroups.

90. Basibranchial 3, development: (0) nodular, vestigial; (1) equal or subequal to basibranchial 2. CI = 0.07.

The basibranchial 3 can be vestigial, nodular (state 0) in outgroups, and most Harttiini, Farlowellini and Loricariini, or elongated, equal or subequal to basibranchial 2 (state 1; Fig 11A–11D) in most *Farlowella*, *Harttia dissidens*, *H*. *gracilis*, *Lamontichthys llanero*, *Sturisoma guentheri*, *St*. *monopelte*, *St*. *nigrirostrum*, *Sturisomatichthys dariensis*, *Dasyloricaria latiura*, *D*. *paucisquama*, *Loricaria lundbergi*, *Rineloricaria lanceolata*, and *R*. *quadrensis*.

91. Basibranchial 2, shape: (0) rod-like, elongate; (1) short, squarish; (2) vestigial CI = 0.18. [28] Ch. 67, [36] Ch. 50.

The basibranchial 2 also varies regarding its shape. It can be rod-like and elongate (state 0; Fig 11A, 11B and 11D) in most of the taxa examined, short and squarish (state 1; Fig 11C) in *Pareiorhaphis calmoni*, *Harttia garavelloi*, *H*. *kronei*, *H*. *leiopleura*, *H*. *longipinna*, *H*. *loricariformis*, *H*. *torrenticola*, *Harttiella crassicauda*, *Lamontichthys filamentosus*, *Sturisoma barbatum*, *St*. *lyra*, *St*. *rostratum*, and *Sturisomatichthys varii*, or vestigial (state 2) in *Pterygoplichthys lituratus* and *Acestridium scutatum*.

92. Ceratobranchial 1, accessory flange, size: (0) median, reaching half ceratobranchial length; (1) large, same size or larger than ceratobranchial; (2) absent or very small, inconspicuous. CI = 0.28. [28] Ch. 72, [36] Ch. 52, [37] Ch. 91, [39] Ch. 55, [41] (modified).

Ceratobranchial 1 bears an anterior accessory flange that supports branchial filaments and varies in size. A medium accessory flange, covering half the ceratobranchial length (state 0; Fig 9C and 9D) is present in Farlowellini, except *Pterosturisoma*. In Harttiini there is a large accessory flange, with the same size or larger than the ceratobranchial (state 1; Fig 9A and 9B) is an exclusive synapomorphy of the tribe. On *Pterosturisoma* and the Loricariini, a very small, inconspicuous or even absent accessory flange (state 2) was observed.

93. Ceratobranchial 1, anterior flange, width: (0) wider than ceratobranchial; (1) same width as ceratobranchial; (-) inapplicable. CI = 1.00. [36] Ch. 53, [37] Ch. 92, [38] Ch. 14.

A broad accessory flange, wider than ceratobranchial 1 (state 0) was found in outgroups and in Loricariini. A flange with the same width as the ceratobranchial (state 1) occurs in Harttiini and Farlowellini. Taxa without the anterior flange on ceratobranchial 1 were coded as inapplicable.

94. Pharyngobranchial 4, shape: (0) thick, large ossified shelf; (1) reduced ossified shelf, nodular, with thick cartilage around; (2) nodular cartilage. CI = 0.13. [28] Ch. 81, [36] Ch. 64 (modified).

The pharyngobranchial 4 is located dorsally to the upper pharyngeal plate. The pharyngobranchial 4 can be thick, as a large ossified shelf (state 0) in Loricariini, *Sturisomatichthys*, and most *Farlowella*; a reduced ossified shelf, nodular, with thick cartilage around (state 1) in *Pterygoplichthys lituratus*, *Aposturisoma myriodon*, *Harttia guianensis*, *Harttiella*, and *Hemiodontichthys acipenserinus*; or as a nodular cartilage (state 2) in remaining *Harttia*, and in *Sturisoma*, *Cteniloricaria*, *Farlowella hahni*, *F*. *knerii*, *F*. *nattereri*, *F*. aff. *amazonum*, *F*. *reticulata*, *F*. *rugosa*, and *Lamontichthys filamentosus*.

95. Lower pharyngeal tooth plate, shape: (0) triangular; (1) rod-like, with an expansion in the middle; (2) trapezoidal; (3) *L*-shaped. CI = 0.25. [36] Ch. 65.

**Fig 12:**
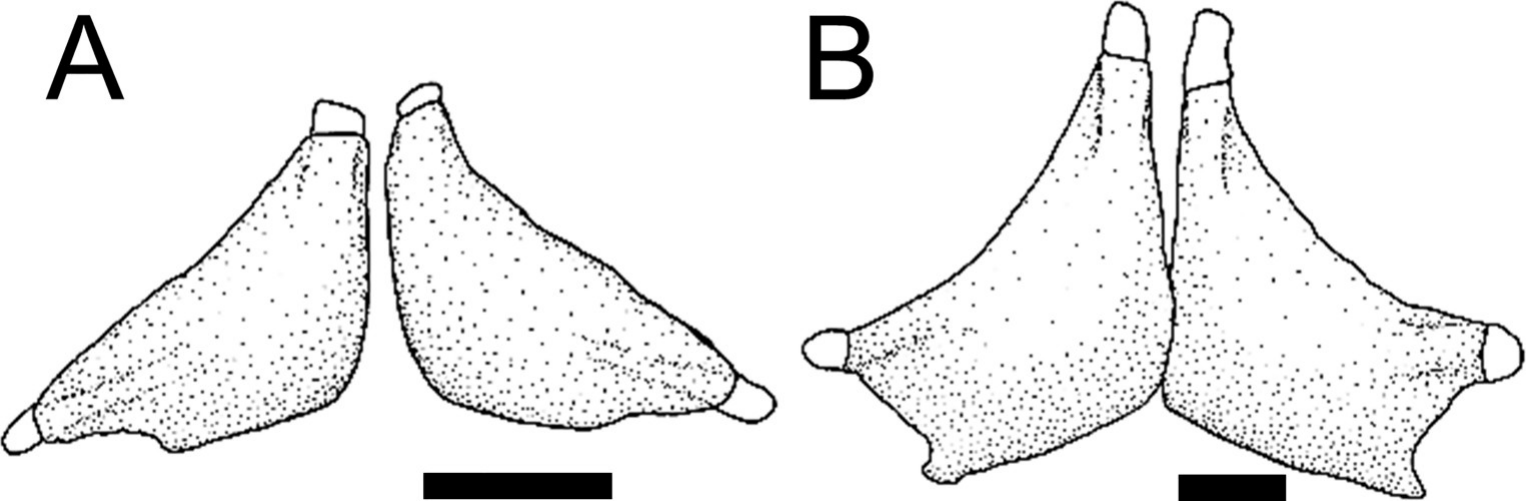
Lower pharyngeal tooth plate in ventral view. **A.** *Sturisomatichthys panamensis*, USNM 316293; **B.** *Sturisoma robustum*, MCP 15812. Scale bar 1 mm.

The lower pharyngeal tooth plate, or Ceratobranchial 5, bears teeth that are related to the feeding mechanism. Besides the variation in such teeth (see following characters), the shape of the plate also varies, and was observed to be triangular (state 0; Fig 12A) in Loricariini, *Cteniloricaria*, *Farlowella*, *Harttia*, and most *Sturisomatichthys*, rod-like (state 1) in Hypostominae, *Harttiella*, *Lamontichthys filamentosus*, and *Sturisomatichthys citurensis*, trapezoidal (state 2; Fig 12B) in *Aposturisoma myriodon*, *Sturisoma*, remaining *Lamontichthys*, and *Hemiodontichthys acipenserinus*, or *L*-shaped (state 3) in *Pseudohemiodon lamina* and *Spatuloricaria puganensis*. A trapezoidal lower pharyngeal plate (state 2) was found to be synapomorphic for *Sturisoma*.

96. Lower pharyngeal tooth plate, teeth, shape: (0) small, pointed; (1) long, pointed; (2) long, broad. CI = 1.00. [37] Ch. 112 (modified).

The difference in tooth shape on the lower pharyngeal tooth plate (or ceratobranchial 5) is probably related to feeding ecology of the Loricariidae. For most Harttiini, Farlowellini and *Metaloricaria*, small, pointed teeth (state 0) is the general condition. In other Loricariini, except *Loricaria* and *Pseudohemiodon*, long pointed teeth (state 1) are present. Long, large and broad teeth (state 2) were observed only in *Loricaria* and *Pseudohemiodon*.

97. Lower pharyngeal tooth plate, teeth, distribution: (0) restricted to mesial area of plate; (1) restricted to central area; (2) plate almost complete to completely toothed; (3) teeth lacking. CI = 0.30. [28] Ch. 85.

Distribution of teeth on lower pharyngeal tooth plate varies as among taxa examined. In outgroups and most Farlowellini and Harttiini, the pharyngeal teeth are restricted to the mesial area (state 0); most Loricariini have teeth restricted to the center of the plate (state 1); in *Dasyloricaria filamentosa*, *Pseudohemiodon lamina*, *Loricaria lundbergi*, *Loricariichthys*, *Rineloricaria lanceolata*, and *Spatuloricaria puganensis* the plate is almost complete to completely toothed (state 2), and in *Aposturisoma myriodon*, *Farlowella hahni*, *Harttiella crassicauda*, *Ha*. *longicauda*, and *Pterosturisoma microps*, teeth are absent from the lower pharyngeal plate (state 3).

98. Epibranchial 1, posterior process: (0) present; (1) absent. CI = 0.07. [28] Ch. 73, [36] Ch. 54, [37] Ch. 93.

**Fig 13:**
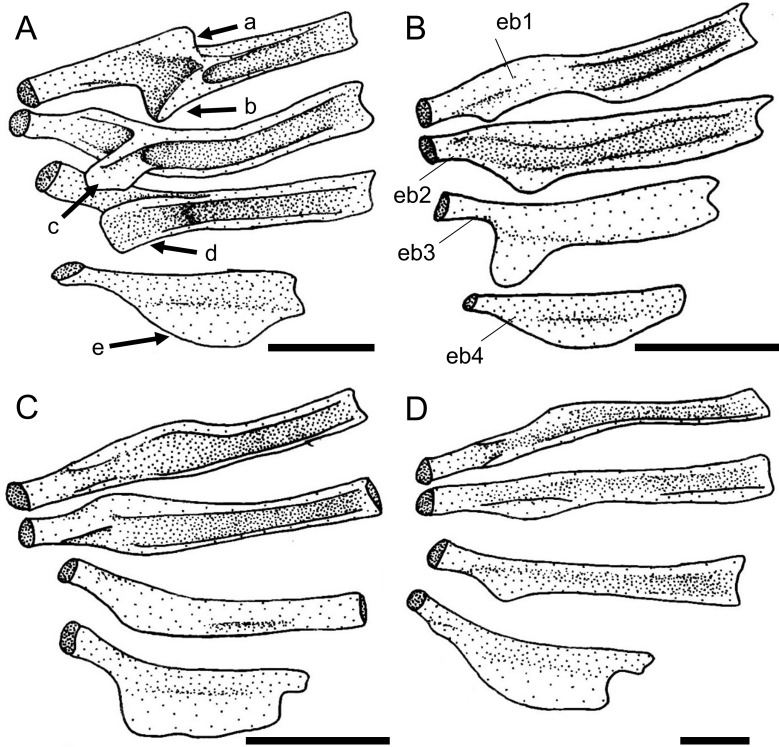
Epibranchials in dorsal view. **A.** *Lamontichthys parakana*, MNRJ 13300; **B.** *Sturisomatichthys panamensis*, USNM 316293; **C.** *Harttia guianensis*, MHNG 2643.033; **D.** *Harttiella crassicauda*, AUM 50387. **Abbreviations:** eb = anterior process epibranchial 1; ep2-pr = epibranchial 2 process; ep3-pr = epibranchial 3 process; ep4-pr = epibranchial 4 process; ppr-ep1 = posterior process epibranchial 1; ep4 = epibranchial 4. Arrows: see text. Scale bar 1 mm.

The epibranchial 1 either have a posterior process (state 0; Fig 13A and 13B, arrow b) in outgroups, Loricariini, *Lamontichthys*, *Sturisomatichthys*, *Sturisoma nigrirostrum*, most *Harttia*, and most *Farlowella*, or lack the process (state 1; Fig 13C and 13D), as observed in *Aposturisoma myriodon*, *Farlowella henriquei*, *F*. *isbruckeri*, *F*. *jauruensis*, *F*. *knerii*, *F*. *nattereri*, *F*. *paraguayensis*, *F*. *smithi*, *F*. *venezuelensis*, *Harttia dissidens*, *H*. *duriventris*, *H*. *fowleri*, *H*. *guianensis*, *H*. *leiopleura*, *H*. *puncata*, *H*. *rhombocephala*, *H*. *trombetensis*, *Harttiella*, and remaining *Sturisoma*.

99. Epibranchial 1, posterior process, shape: (0) elongated and rounded; (1) relatively elongated and triangular; (2) reduced, as a triangle; (-) inapplicable. CI = 0.11. [28] Ch. 73 (modified).

The epibranchial 1 may bear a posterior process that can be elongated and rounded (state 0; Fig 13A, arrow b) in *Hemipsilichthys gobio*, *Hisonotus laevior*, *Lamontichthys*, *Cteniloricaria*, *Farlowella* aff. *amazonum*, *Harttia fluminensis*, *Sturisoma nigrirostrum*, *Hemiodontichthys acipenserinus*, and *Limatulichthys griseus*, relatively elongated but triangular (state 1) in *Farlowella oxurryncha*, *F*. *reticulata*, *Harttia carvalhoi*, *H*. *kronei*, *H*. *loricariformis*, *H*. *torrenticola*, *Pterosturisoma*, *Sturisomatichthys citurensis*, *S*. *festivus*, *S*. *leightoni*, *S*. *tamanae*, and remaining Loricariini, or reduced in size and triangular (state 2; Fig 13B) in other outgroups, *Farlowella acus*, *F*. *amazonum*, *F*. *curtirostra*, *F*. *hahni*, *F*. *hasemani*, *F*. *mariaelenae*, *F*. *rugosa*, *F*. *schreitmuelleri*, *F*. *vittata*, *Harttia garavelloi*, *H*. *gracilis*, *H*. *longipinna*, *H*. *novalimensis*, and remaning *Sturisomatichthys*. The presence of a posterior process on epibranchial 1 (see character 102) was variable among taxa examined, and a wide variation was observed within the monophyletic groups revealed here. The same degree of variation was found regarding the difference in shape of that same process.

100. Epibranchial 1, anterior process: (0) present; (1) absent. CI = 0.07. [36] Ch. 55, [37] Ch. 94 (modified).

Previous authors used both the epibranchial shape and the presence/absence of posterior and/or anterior processes in the same character. As these features do not co-vary in the studies species, both types of information were kept separated and additional characters were included for each of the epibranchials, dealing with presence/absence and shape of processes. The anterior process of the epibranchial 1 can be either present (state 0; Fig 13A, 13C and 13D, arrow a) in most taxa examined, or absent (state 1; Fig 13B) in *Farlowella amazonum*, *F*. *isbruckeri*, *F*. *jauruensis*, *F*. *knerii*, *F*. *oxyrryncha*, *F*. *vittata*, *Harttia carvalhoi*, *H*. *gracilis*, *Sturisomatichthys*, *Sturisoma graffini*, *Loricaria lundbergi*, *Metaloricaria*, *Pseudohemiodon lamina*, *Rineloricaria*, and *Spatuloricaria puganensis*.

101. Epibranchial 1, anterior process, shape: (0) small, inconspicuous; (1) long, pointed; (2) large, laminar; (-) inapplicable. CI = 0.13. [36] Ch. 55 (modified).

An anterior process on the epibranchial 1 is observed, besides the Loricariidae, in the Astroblepidae [28, 36]. Such process can be small (state 0; Fig 13C and 13D) in outgroups, *Aposturisoma myriodon*, *Farlowella acus*, *F*. *hasemani*, *F*, *henriquei*, *F*. *mariaelenae*, *F*. *nattereri*, *F*. aff. *amazonum*, *F*. *rugosa*, *F*. *smithi*, *F*. *venezuelensis*, *Harttia garavelloi*, *H*. *guianensis*, *H*. *longipinna*, *H*. *torrenticola*, *Harttiella*, *Sturisoma robustum*, *Sturisoma* aff. *tenuirostre*, and *Hemiodontichthys acipenserinus*, long and pointed (state 1) in *Cteniloricaria*, most *Harttia*, and *Lamontichthys filamentosus*, or large and laminar (state 2; Fig 13A, arrow a) in *Farlowella curtirostra*, *F*. *hahni*, *F*. *paraguayensis*, *F*. *reticulata*, *F*. *schreitmuelleri*, *Dasyloricaria*, *Loricariichthys*, *Limatulichthys griseus*, and remaining *Lamontichthys* and *Sturisoma*. An anterior process can also be absent, and taxa having such a condition were coded as inapplicable.

102. Epibranchial 2, posterior process, shape: (0) laminar flap; (1) absent. CI = 0.10. [36] Ch. 56 (modified).

A posterior process on the epibranchial 2 occurs in a few taxa examined, being a laminar flap (state 0; Fig 13A and 13B, arrow c) in Loricariini, some Farlowellini, and in outgroups; such process was absent (state 1; Fig 13C and 13D) in the Harttiini and some Farlowellini.

103. Epibranchial 2, anterior process: (0) absent; (1) reduced; (2) small, uncinate, base expanded; (3) large, laminar; (4) elongated and laminar, expanded along its entire length. CI = 0.17. [28] Ch. 74, [36] Ch. 57, [37] Ch. 96 (modified).

*Sturisomatichthys*, *Harttiella*, *Lamontichthys*, *Metaloricaria*, *Hemiodontichthys*, *Limatulichthys griseus*, and *Hemipsilichthys gobio* lack an anterior process on the epibranchial 2 (state 0). A reduced process (state 1; Fig 13C) was only observed in *Harttia*, *Farlowella nattereri*, *F*. *rugosa*, *F*. *venezuelensis*, *Spatuloricaria puganensis*, and members of the Hypostominae examined. *Aposturisoma*, some *Farlowella*, and remaining outgroups, have a small, uncinate process, expanded at the base (state 2). A large, laminar process (state 3; Fig 13B) was observed in *Harttiella longicauda*, *Sturisoma*, and some *Farlowella*. Finally, in *Cteniloricaria* and *Pterosturisoma* an elongated and laminar process, expanded along its entire length is found (state 4; Fig 13A).

104. Epibranchial 3, posterior process: (0) uncinate, laterally expanded; (1) elongated, laterally expanded; (2) small, short; (3) absent. CI = 0.42. [28] Ch. 76, [36] Ch. 58, [37] Ch. 97 (modified).

Outgroups have an uncinate, laterally expanded process (state 0). Members of the Harttiini and Farlowellini, except *Farlowella venezuelensis*, have an elongated, laterally expanded process (state 1; Fig 13A and 13B, arrow d). *Loricariichthys*, *Hemiodontichthys*, *Farlowella venezuelensis*, *Pseudohemiodon lamina*, *Limatulichthys griseus* and the Hypostominae have a small and short process (state 2). The absence of a process was observed in the remaining Loricariini examined (state 3).

105. Epibranchial 4, shape: (0) curved, expanded posteriorly; (1) straight, expanded posteriorly; (2) curved, rod shaped; (3) straight, rod shaped. CI = 0.21. [37] Ch. 114 (modified).

Harttiini and *Farlowella henriquei* have a curved epibranchial 4, with its posterior margin expanded (state 0; Fig 13C and 13D). *Lamontichthys*, *Pterosturisoma*, *Sturisomatichthys*, and Hypostominae have the epibranchial 4 straight, with the postrior margin expanded in a lamina (state 1; Fig 13A and 13B, arrow e). *Aposturisoma*, most *Farlowella*, *Sturisomatichthys* and some Loricariini have the epibranchial 4 shaped as a curved rod (state 2), while an epibranchial 4 as a straight rod (state 3) was observed in some *Farlowella*, all *Sturisoma*, and some Loricariini.

106. Epibranchial 4, gill filaments: (0) strongly attached; (1) weakly attached; (2) without filaments. CI = 0.50. [28] Ch. 79, [36] Ch. 61, [37] Ch. 101 (modified).

In Harttiini, Farlowellini and examined members of subfamilies outside the Loricariinae, the gill filaments on epibranchial 4 are strongly attached (state 0). In Loricariini, both weakly attached filaments (state 1) and the absence of them (state 2), were observed.

107. Upper pharyngeal plate, shape: (0) round or oval; (1) *L*-shaped; (2) club-shaped; (3) triangular. CI = 0.42. [28] Ch. 87, [36] Ch. 68, [37] Ch. 108 (modified).

**Fig 14:**
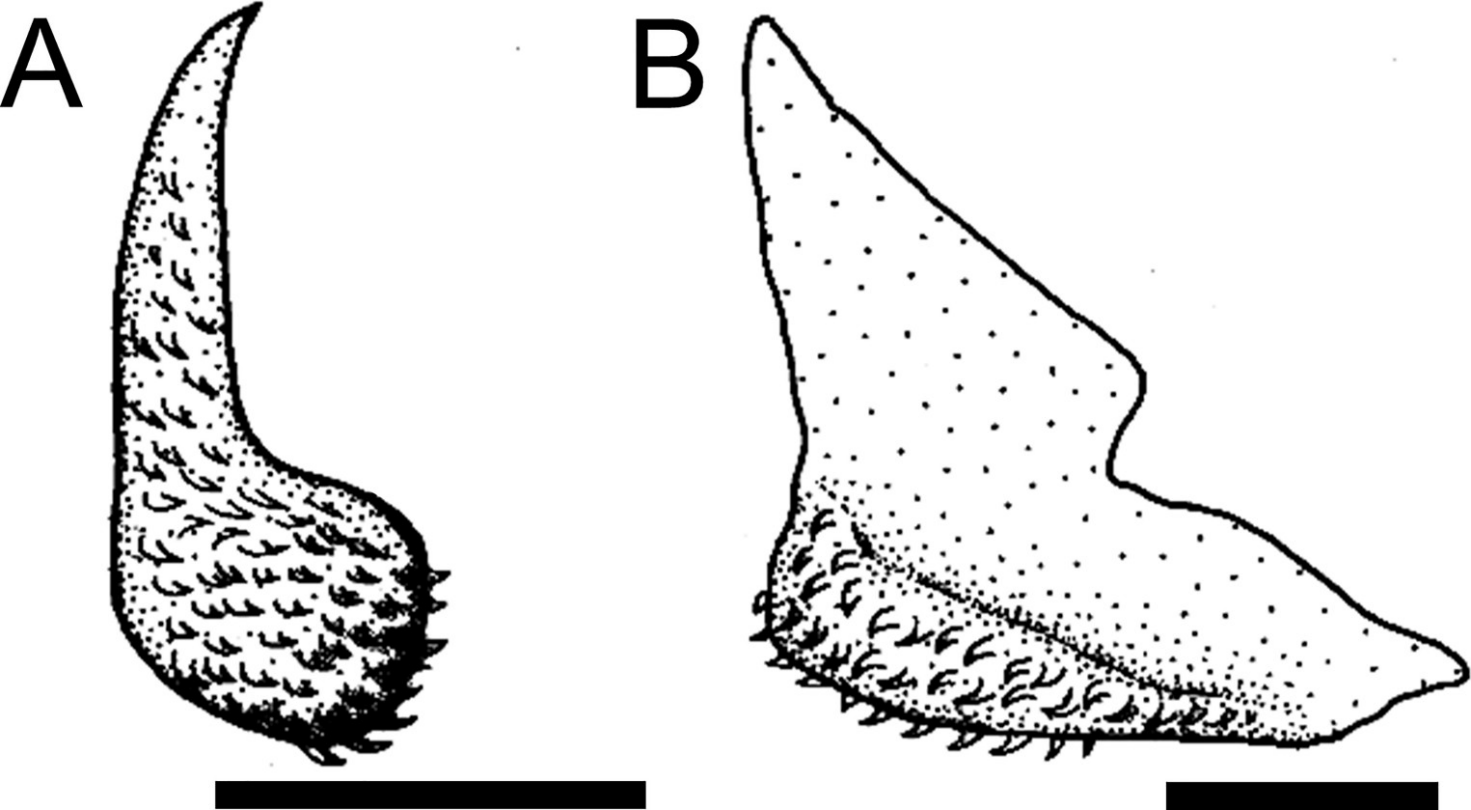
Upper pharyngeal tooth plate in ventral view. **A.** *Sturisomatichthys panamensis*, USNM 316293; **B.** *Sturisoma robustum*, MCP 15812. Right side. Scale bar 1 mm.

Loricariini have round or oval upper pharyngeal plate (state 0), while Harttiini have *L*-shaped upper pharyngeal plate (state 1). A club-shaped upper pharyngeal plate (state 2; Fig 14A) occurs in Farlowellini, except *Sturisoma*. For the latter, a triangular upper pharyngeal plate (state 3; Fig 14B) was observed.

108. Upper pharyngeal tooth plate, dentition: (0) complete; (1) incomplete. CI = 0.20. [28] Ch. 88, [36] Ch. 70, [37] Ch. 110 (modified).

In Loricariini, *Sturisomatichthys*, *Lamontichthys filamentosus* and *L*. *llanero* the upper pharyngeal plate is completely toothed (state 0). In Harttiini and the remainder Farlowellini, that plate is incompletely toothed (state 1).

109. Branchiostegal rays, number: (0) four; (1) fewer than four. CI = 0.50. [11] Ch. 5, [28] Ch. 55, [36] Ch. 44, [37] Ch. 79 (modified).

In Loricariinae, the presence of four branchiostegal rays is the general condition (state 0). Nevertheless, *Farlowella* has fewer (state 1).

### Weberian apparatus and axial skeleton

110. Transcapular ligament, connection to parapophysis of complex centrum: (0) connected; (1) not connected. CI = 0.33. [28] Ch. 92.

In *Aposturisoma*, *Farlowella*, *Pterosturisoma*, *Sturisoma*, *Sturisomatichthys*, and Loricariini the transcapular ligament, or Baudelot´s ossified ligament [38], contacts the parapophysis of the complex centrum (state 0). In the Harttiini and *Lamontichthys* the transcapular ligament does not contact the parapophysis (state 1).

111. Transverse process of complex centrum, fenestra with laminar process at limit with basioccipital: (0) absent; (1) present. CI = 1.00.

The Weberian apparatus of loricariids comprises the first five fused vertebrae and associated ribs [71, 72], and its origin has been extensively studied [73–75]. The apparatus has a lateral expansion, the transverse process of the complex centrum, which articulates anteriorly with the basioccipital and compound pterotic and encapsulates the swim bladder [41, 76]. A fenestra with a small laminar process between the transverse process and the basioccipital (state 1; Fig 5, arrow b), was observed as an exclusive synapomorphy of *Sturisomatichthys*. This condition is absent in the remaining taxa examined.

112. Transverse process of complex centrum, type of contact to compound pterotic: (0) abutting; (1) suture. CI = 0.25. [28] Ch. 93.

The contact between the compound pterotic and the transverse process of the complex centrum was found to be either abutting (state 0; [28: fig 26A]) in most taxa examined, or as a suture (state 1; [28: fig 26B]) in *Harttia*, *Lamontichthys*, and *Sturisomatichthys panamensis*.

113. Transverse process of complex centrum, length: (0) short, not reaching compound pterotic lateral border; (1) approximately same size as compound pterotic; (2) long, surpassing compound pterotic lateral border; (3) long, reaching sixth vertebral rib. CI = 0.23. [28] Ch. 94, [37] Ch. 32.

The transverse process of complex centrum was observed to be short, not reaching the level of the compound pterotic lateral border (state 0; [28: fig 26B]) in most taxa examined, approximately the same size as the compound pterotic (state 1) in *Neoplecostomus microps*, *Pareiorhaphis calmoni*, *Farlowella isbruckeri*, *F*. *paraguayensis*, *F*. aff. *amazonum*, *F*. *venezuelensis*, *Harttiella*, *Sturisoma monopelte*, *St*. *nigrirostrum*, *Loricariichthys*, and *Spatuloricaria puganensis*, surpassing the compound pterotic laterally (state 2; [28: fig 26A]) in *Metaloricaria*, *Dasyloricaria*, *Limatulichthys griseus*, *Loricaria lundbergi*, *Rineloricaria cadea*, and *R*. *quadrensis*, or reaching the sixth vertebral rib (state 3) observed only in *Rineloricaria lanceolata*.

114. Sixth vertebra, neural spine, lamina for articulation with ventral surface of supraoccipital: (0) anteriorly inclined; (1) straight and upright; (2) reduced. CI = 0.28. [39] Ch. 24.

*Harttia loricariformis*, *H*. *gracilis*, *H*. *leiopleura*, *H*. *novalimensis*, *H*. *longipinna*, *H*. *rhombocepahala*, *Metaloricaria*, and outgroups show an anteriorly inclined neural spine on the sixth vertebra (state 0). *Farlowella* shows a straight and upright lamina (state 1) as an exclusive synapomorphy, contrary to what was observed by Provenzano [39] regarding the genus. A reduced lamina (state 2), was observed on the remaining species of *Harttia*, *Harttiella*, *Lamontichthys*, *Pterosturisoma*, *Sturisoma*, *Sturisomatichthys*, the Loricariini, and *Aposturisoma*; the present analysis agrees with Provenzano [39] regarding the state of the latter.

115. Aortic canal, extension: (0) reaching sixth or seventh vertebral centrum; (1) reaching eighth centrum; (2) reaching ninth through eleventh centrum. CI = 0.18. [28] Ch. 98, [37] Ch. 36 (modified).

The aortic canal occurs on the ventral surface of the complex vertebra and gives passage to the aorta artery initially passing through the channel that closes at a given vertebra forming the hemal arch [37]. The aortic canal reaches the sixth or seventh vertebral centrum (state 0) on most taxa examined; reaches the eighth centrum (state 1) in *Harttia* (except *H*. *leiopleura*, and *H*. *rhombocephala*), *Lamontichthys*, *Metaloricaria*, most *Sturisoma*, and most *Sturisomatichthys*; or reaches from ninth through eleventh centrum (state 2) in *Sturisoma monopelte*, *Sturisomatichthys kneri*, and *S*. *leightoni*. Ranges of vertebral number used by previous authors were different from those found as informative in the present study.

116. Seventh vertebra, lateral portion, flange: (0) absent; (1) present. CI = 0.16. [38] Ch. 39.

The present analysis agrees with Paixão and Toledo-Piza [38] in that, in general, most Loricariidae lack a flange on the seventh vertebral centrum (state 0; [38: fig 38B]). Nevertheless, in *Farlowella knerii*, *F*. *jauruensis*, *Limatulichthys griseus*, *Spatuloricaria puganensis*, *Sturisomatichthys citurensis*, and all *Lamontichthys* examined (including *L*. *avacanoeiro*, according to Paixão and Toledo-Piza [38]) the flange is present (state 1; [38: fig 38A]).

117. Seventh vertebra, pleural rib: (0) present and complete; (1) present but short; (2) absent. CI = 0.22. [28] Ch. 113.

In loricariids the pleural rib associated to the seventh vertebra can be either present and complete (state 0) in some outgroups, Loricariini, and most Harttiini; present and short (state 1) in most Farlowellini and some Harttiini; or absent (state 2) in most *Farlowella*.

118. Connecting bone, dorsal contact: (0) contacting lateral process of second dorsal-fin pterygiophore; (1) contacting lateral process of first dorsal-fin pterygiophore; (2) not contacting pterygiophores; (3) absent. CI = 0.18. [28] Ch. 119, [36] Ch. 83, [38] Ch. 59, [77] (modified).

Conditions regarding *Harttia*, *Farlowella* and *Lamontichthys* were confirmed when compared to those described by Paixão and Toledo-Piza [38]. Some *Harttia*, all *Lamontichthys*, some *Sturisomatichthys*, *Cteniloricaria*, *Pterosturisoma*, and *Metaloricaria*, have the connecting bone contacting the lateral process of the second dorsal-fin pterygiophore (state 0; [38: fig 46A]). In some *Sturisoma* and Loricariini, the connecting-bone contacts the lateral process of the first dorsal-fin pterygiophore (state 1). In remaining *Sturisoma* and *Sturisomatichthys*, the connecting-bone lacks a contact with the dorsal pterygiophores (state 2; [38: fig 46B]). Finally, *Aposturisoma*, *Harttiella*, *Farlowella*, and the remaining *Harttia* species lack a connecting bone (state 3).

119. Complex centrum, ventral process, position: (0) at anterior margin of complex centrum; (1) at middle of complex centrum. CI = 1.00. [28] Ch. 96, [37] Ch. 34 (modified).

The complex centrum bears a pair of ventral processes, which can vary in shape, size, position, and degree of development [28, 41, 71]. Fewer states were used here compared to previous authors [28, 37] regarding the position of the ventral process. The complex centrum ventral process can be at the anterior margin of the complex centrum (state 0) on most taxa examined, or at the middle of complex centrum (state 1) exclusively in *Sturisoma*, except *St*. aff. *tenuirostre*, and *St*. *graffini*.

120. Dorsal fin, spinelet, shape: (0) *V*-shaped; (1) plate-like; (2) absent. CI = 0.20. [11] Ch. 149, [28] Ch. 114, [36] Ch. 80, [37] Ch. 37, [38] Ch. 52.

According to Armbruster [11] and Pereira and Reis [16], in loricariids and most other catfishes, the first dorsal-fin spine is a short, *V*-shaped structure often termed spinelet. That structure is located in front of the second, much longer, dorsal-fin spine and slips under the nuchal plate to lock the dorsal-fin spine in an upright position. The *V*-shaped spinelet and the resulting functional locking mechanism (state 0; [11: fig 28A]) is absent in Loricariinae, but a plate-like spinelet is instead present in most species (state 1; [11: fig 28B]). Many species of *Harttia*, *Harttiella*, *Farlowella*, *Aposturisoma*, and Loricariini completely lack a spinelet (state 2).

121. Dorsal fin, pterygiophores, number: (0) eight; (1) fewer than eight; (2) more than eight. CI = 0.25. [28] Ch. 116, [36] Ch. 82.

The number of pterygiophores of the dorsal fin is functionally related to the number of rays in that fin, as stated by Rapp Py-Daniel [28]. Eight is the general number to be found across loricariids (state 0) in outgroups, *Harttia*, *Lamontichthys*, *Sturisoma robustum*, and most Loricariini, but variation was found in both directions, to fewer than eight (state 1) in *Pterygoplichthys lituratus*, *Farlowella*, *Harttiella*, remaining *Sturisoma*, and *Rineloricaria lanceolata*, and to more than eight pterygiophores (state 2) in *Sturisomatichthys* and *Limatulichthys griseus*.

122. Dorsal fin, pterygiophores, extent of contact: (0) all pterygiophores separated; (1) first and second pterygiophores in contact, others separated; (2) second and third pterygiophore separated, all others in contact; (3) all pterygiophores in contact. CI = 0.15. [28] Ch. 117.

The dorsal-fin pterygiophores are not fused to each other, but demonstrate variation in the degree of contact. Such structures were observed to be separated (state 0) in outgroups, *Aposturisoma myriodon*, *Sturisomatichthys dariensis*, *S*. *reinae*, and in most *Sturisoma* and Loricariini, only first and second in contact (state 1) in *Cteniloricaria*, *Farlowella isbruckeri*, *F*. *vittata*, *Harttia*, *Harttiella*, *Lamontichthys*, *Sturisomatichthys citurensis*, *S*. *frenatus*, *S*. *kneri*, and *S*. *varii*, only the second and third pterygiopohore separated, while the others are in contact (state 2) in *Farlowella jauruensis*, *F*. *reticulata*, *F*. *venezuelensis*, and *Sturisomatichthys festivus*, or contact between all pterygiophores (state 3) in remaining *Farlowella* and *Sturisomatichthys*, and in *Loricariichthys*.

123. Dorsal fin, second pterygiophore, lateral process, orientation: (0) straight, directed laterally; (1) strongly curved, anterolaterally oriented. CI = 0.11. [28] Ch. 118, [38] Ch. 55.

*Aposturisoma*, *Harttia* (except *H*. *garavelloi*), *Farlowella*, *Sturisomatichthys*, some *Sturisoma*, and some Loricariini have the lateral process of second dorsal pterygiophore straight and directed laterally (state 0; [38: fig 46C]). A strongly curved, anterolaterally oriented process (state 1; [38: fig 46B]) is found in *Lamontichthys*, *Cteniloricaria*, *Pterosturisoma*, some *Sturisoma*, and some Loricariini. Present results partially agree with those of Rapp Py-Daniel [28] and Paixão and Toledo-Piza [38].

124. Dorsal fin, spine, type of articulation with second dorsal-fin pterygiophore: (0) through condyle on distal end of pterygiophore; (1) through a circular hollow structure. CI = 0.20. [28] Ch. 115, [38] Ch. 53, [39] Ch. 63.

As discussed by Armbruster [11], Paixão and Toledo-Piza [38], and Schaefer [41], the dorsal-fin spine of the Loricariinae articulates with the second pterygiophore via a chain-like structure (referred to here as a circular hollow structure). The articulation through a condyle (state 0; [38: fig 45A]) was observed in most outgroups, *Ctenoloricaria*, *Harttia*, and *Harttiella*. All other taxa examined has the articulation of the dorsal-fin spine through a circular hollow structure (state 1; [38: fig 45B]).

125. Dorsal fin, first pterygiophore, transverse process, length: (0) shorter than process of second pterygiophore; (1) same size as process of second pterygiophore; (2) longer than process of second pterygiophore; (3) transverse process absent. CI = 0.33. [38] Ch. 54.

The dorsal-fin pterygiophores of loricariids variably possess a pair of transverse processes involved in the support of the nuchal plate and first rays of the dorsal fin [38]. The transverse process on the first pterygiophore can be shorter than the process in the second pterygiophore (state 0) in outgroups, *Pterosturisoma*, *Sturisomatichthys citurensis*, *S*. *tamanae*, and *Metaloricaria*, as long as the second (state 1) in *Cteniloricaria*, *Harttia punctata*, and most *Lamontichthys*, longer than the second (state 2) in *Farlowella knerii* and *Sturisomatichthys festivus*, or absent (state 3) in *Aposturisoma myriodon*, *Harttiella*, Loricariini, and remaining Farlowellini and Harttiini.

126. Dorsal fin, fifth pterygiophore, transverse process: (0) present; (1) absent. CI = 0.05. [38] Ch. 57.

The fifth dorsal-fin pterygiophore bears a transverse process in most loricariids examined (state 0; [38: fig 44]). Contrary to Paixão and Toledo-Piza [38], the lack of such process is observed on some *Sturisomatichthys* species, but also on some *Harttia* and *Farlowella*.

127. Dorsal fin, sixth pterygiophore, transverse process: (0) present; (1) absent. CI = 0.05. [38] Ch. 58, [39] Ch. 57.

Present results partially agree with Paixão and Toledo-Piza [38] in that *Harttia* and *Sturisomatichthys* lack the transverse process in the sixth dorsal-fin pterygiophore (state 1), while *Lamontichthys*, *Farlowella*, and *Sturisoma* possess it (state 0; [38: fig 44]).

128. Precaudal vertebrae, number: (0) more than 14; (1) 13; (2) 12; (3) 11. CI = 0.12. [28] Ch. 110, [36] Ch. 78, [38] Ch. 50, [39] Ch. 58 (modified).

More than 14 precaudal vertebrae was not observed in the Loricariinae, which agrees with previous authors [28, 38, 41]. A reduction to 11 precaudal vertebrae occurs in *Sturisomatichthys* and some Harttiini and Loricariini.

129. Anteriormost paraneural spines in contact with dorsal plates, shape: (0) long; (1) short. CI = 1.00. [36] Ch. 76.

**Fig 15:**
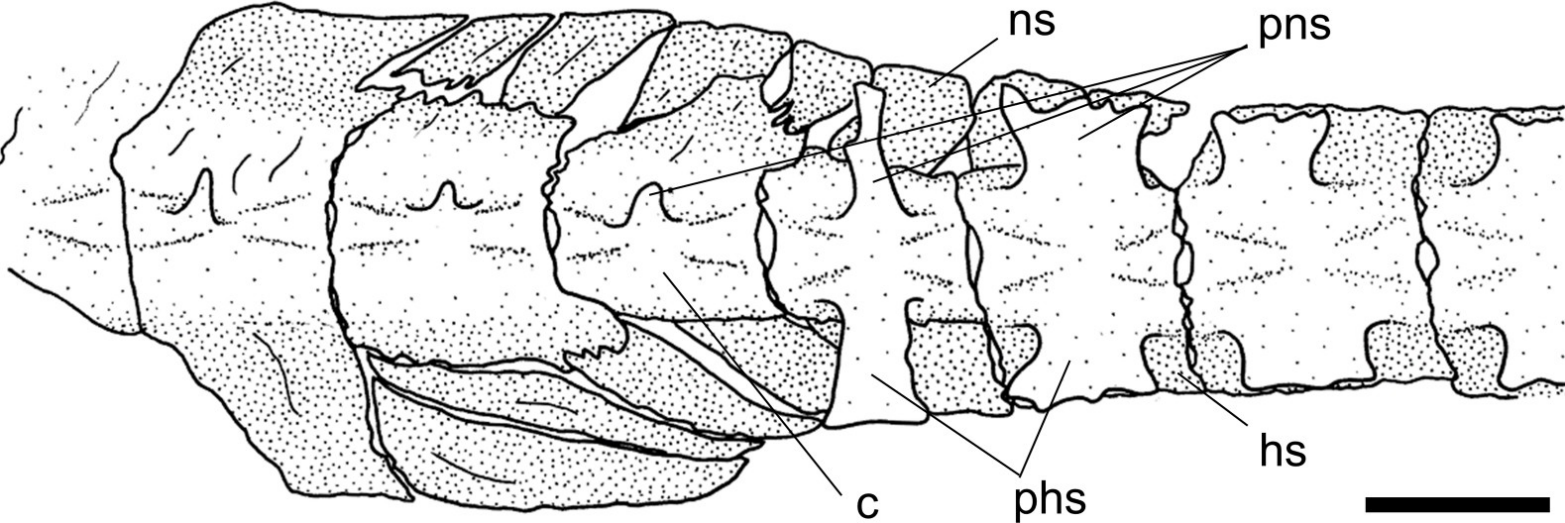
Anterior caudal vertebrae in lateral view. *Farlowella venezuelensis*, USNM 163179. **Abbreviations:** c = vertebral centrum; hs = hemal spine; ns = neural spine; pns = paraneural spines. Scale bar 2 mm.

Paraneural spines branch out of the vertebral centra in some caudal vertebrae of Loricariinae and articulate with plates of the dorsal lateral series of plates, reinforcing the caudal-peduncle structure. Short anteriormost paraneural spines are a synapomorphy of *Aposturisoma* and *Farlowella* (state 1; Fig 15).

130. First caudal vertebra, bifid hemal spine: (0) short, almost inconspicuous; (1) medium, length approximately half of corresponding vertebra; (2) long, approximately as long as corresponding vertebra. CI = 0.09. [38] Ch. 41.

Length of bifid hemal spine on first caudal vertebra as described by Paixão and Toledo-Piza [38] was partially corroborated here. Most *Farlowella* species, *Sturisoma nigrirostrum*, and outgroups have a very short, almost inconspicuous bifid hemal spine (state 0). On the other hand, medium (state 1; [38: fig 41]), and long (state 2; [38: fig 40]) bifid hemal spines were observed among remaining taxa of the Loricariinae.

131. Caudal vertebral centra, paraneural and parahemal spines: (0) absent; (1) present. CI = 1.00. [28] Ch. 106, [38] Ch. 43.

As described by Rapp Py-Daniel [28] and Paixão and Toledo-Piza [38], the presence of bilateral projections on vertebrae, or paraneural and parahemal spines, is unique within Loricariinae, representing an unreversed synapomorphy (Fig 15).

132. Second caudal vertebra, ventrally directed bilateral projections, orientation: (0) slightly anteriorly or posteriorly directed; (1) distinctly posteriorly directed. CI = 0.14. [38] Ch. 44.

Most taxa examined have the ventrally directed bilateral projection on the second caudal vertebra, slightly anteriorly or posteriorly directed (state 0; [38: fig 42B]). *Harttia loricariformis*, *H*. *garavelloi*, *H*. *longipinna*, *H*. *fluminensis*, *H*. *kronei*, *Harttiella crassicauda*, *Cteniloricaria platystoma*, and *Farlowella schreitmuelleri* have distinctly posteriorly directed bilateral projections (state 1; [38: fig 42A]), which partially agrees with Paixão and Toledo-Piza [38] regarding *Harttia* species.

133. Second preural centrum, posterior process of hemal spine, length: (0) long; (1) short. CI = 0.10. [38] Ch. 46.

*Harttia*, *Harttiella*, *Sturisomatichthys*, and *Metaloricaria*, as well as some outgroup members, have a long posterior process on the hemal spine on second preural centrum (state 0; [38: fig 43]). A short process (state 1) was the most common condition within the Loricariinae and in Hypostominae.

134. Second preural centrum, hemal spine, cartilage on posterior tip: (0) present; (1) absent. CI = 0.33. [28] Ch. 47, [38] Ch. 47 (modified).

Our results agree with Rapp Py-Daniel [28], and Paixão and Toledo-Piza [38] in that the presence of a cartilage on the posterior tip of the hemal spine of the second preural centrum is only observed in *Harttia* among the Loricariinae.

135. Second preural centrum, neural spine, relative length: (0) reaches vertical through half length of hypural plate; (1) reaches vertical through one third length of hypural plate; (2) reaches vertical through posterodorsal tip of hypural plate. CI = 0.40. [28] Ch. 134, [38] Ch. 48.

Among loricariids, the neural spine of the second preural centrum can reach half length (state 0; [38: fig 43B]) in outgroups, *Harttia punctata*, and most Loricariini, one third length (state 1; [38: fig 43A]) in Farlowellini and remaining Harttiini, or the posterodorsal tip of the hypural plate (state 2; [38: fig 43C]) in *Chaetostoma breve*, *Pterygoplichthys lituratus*, and *Metaloricaria*.

136. Caudal peduncle, cross-section: (0) cylindrical or ovoid; (1) depressed. CI = 0.50. [28] Ch. 108, [36] Ch. 77, [38] Ch. 49.

As proposed by several authors [19, 22, 41, 66] a depressed caudal peduncle is a synapomorphy of the Loricariinae (state 1; [62: fig 3A], even though this characteristic is also found convergently in the hypoptomatines *Acestridium*, *Niobichthys*, and *Oxyropsis*. The plesiomorphic condition of cylindrical or ovoid caudal peduncle, was found in most loricariids (state 0; [62: fig 3B]).

### Pectoral girdle

137. Pectoral fin, branched rays, number: (0) six; (1) seven. CI = 1.00. [28] Ch. 139, [36] Ch. 99, [37] Ch. 132, [38] Ch. 60.

Seven branched rays in the pectoral fin was found to be an exclusive synapomorphy of *Lamontichthys* among the Loricariinae.

138. Cleithrum, anterior margin: (0) curved, expanded anteriorly; (1) slightly curved; (2) straight. CI = 0.50. [28] Ch. 140, [36] Ch. 100, [37] Ch. 128 (modified).

**Fig 16:**
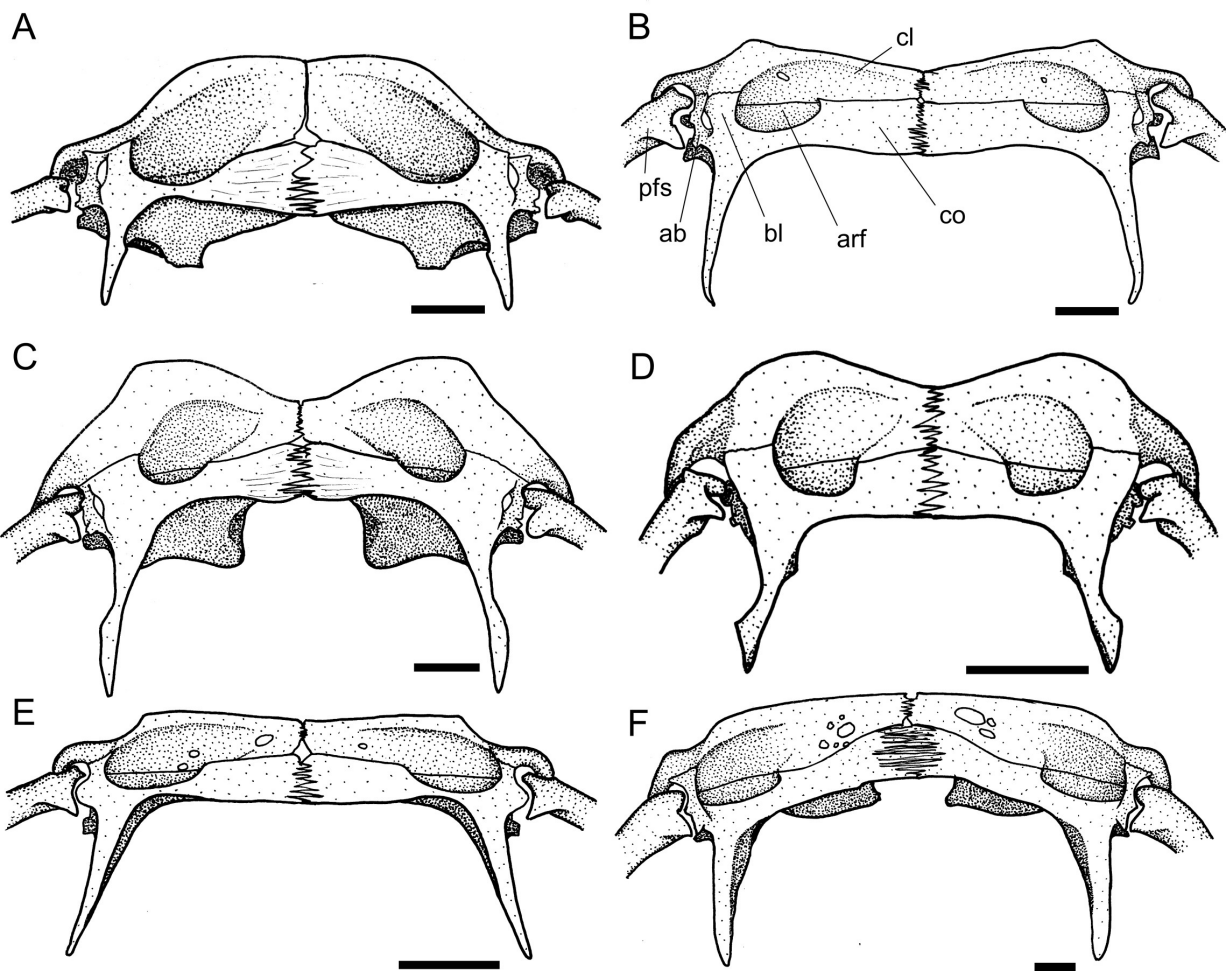
Pectoral girdle in ventral view. **A.** *Rineloricaria quadrensis*, MCP 11039; **B.** *Sturisomatichthys tamanae*, CAS 67414; **C.** *Sturisoma robustum*, MCP 15812; **D.** *Farlowella mariaelenae*, USNM 349392; **E.** *Harttiella longicauda*, MHNG 2723.042; **F.** *Harttia loricariformis*, MCP 11707. **Abbreviations:** ab = arrector bridge; arf = arrector fossa; bl = bony lamina; cl = cleithrum; co = coracoid; pfs = pectoral-fin spine. Scale bar 2 mm.

The anterior margin of the cleithrum can be curved, expanded anteriorly (state 0; Fig 16A, 16C and 16D) in outgroups, Loricariini, and most Farlowellini; slightly curved, not much expanded (state 1; Fig 16B) in Harttiini and *Pterosturisoma*; or straight (state 2; Fig 16E and 16F) in *Ancistrus brevipinnis* and *Chaetostoma breve*. Due to a different codification scheme adopted here, distinct and fewer states were coded regarding shapes of the anterior margin of the cleithrum (see references above).

139. Cleithrum, symphysis, type of articulation: (0) sutural, interdigitated; (1) simple, not interdigitated. CI = 0.25. [28] Ch. 143, [36] Ch. 103 (modified).

Harttiini, Farlowellini, and *Pseudohemiodon lamina*, have the contralateral cleithra sutured with interdigitations along the entire or most of symphyseal contact (state 0; Fig 16B–16F). In all remaining Loricariini the contralateral cleithra articulate without an interdigitate suture (state 1; Fig 16A).

140. Cleithrum, orientation of vertical walls: (0) straight; (1) posteriorly inclined. CI = 0.06. [28] Ch. 144, [36] Ch. 104.

The vertical walls of the cleithrum articulate with the compound pterotic dorsally, and form the posterior walls of the branchial apparatus and cardiac chamber [28]. The cleithrum vertical walls can be straight (state 0) in outgroups, *Aposturisoma myriodon*, *Lamontichthys*, *Sturisoma monopelte*, *St*. *nigrirostrum*, *St*. aff. *tenuirostre*, *St*. *graffini*, and most *Sturisomatichthys* and *Farlowella*, or posteriorly inclined (state 1) in Harttiini, *Farlowella curtirostra*, *F*. *hahni*, *F*. *isbruckeri*, *F*. *mariaelenae*, *F*. *nattereri*, *F*. *smithi*, *Pterosturisoma*, *Sturisomatichthys citurensis*, *S*. *dariensis*, *S*. *leightoni*, *S*. *varii*, remaining *Sturisoma*, and Loricariini.

141. Pectoral girdle *arrector* fossa: (0) large, open; (1) encapsulated by cleithrum and coracoid laminae. CI = 0.50. [27] Ch. 41, [28] Ch. 146, [36] Ch. 106 (modified).

The *arrector fossa* of loricariids is a bony depression on the ventral portions of the cleithrum and coracoid that serves as attachment site for the muscles *arrector ventralis superficialis* and the *arrector ventralis profundus*, responsible for the abducting of the pectoral fin [16, 38, 41]. Most Hypoptopomatinae possess the *arrector fossa* encased by horizontal, laminar projections of both the cleithrum and the coracoid (state 1). All remaining loricariids have an open *arrector fossa* (state 0).

142. Cleithrum, posterior process, shape: (0) long, narrow portion exposed; (1) short, narrow portion exposed; (2) short, broad portion exposed. CI = 0.12. [36] Ch. 109.

Within Farlowellini, *Sturisomatichthys* and *Lamontichthys filamentosus* are the only taxa that have a long, narrow posterior cleithral process that appears laterally above the pectoral-fin insertion (state 0; [36: fig 37A]), as cited by Ghazzi [36]. Some *Harttia*, *Harttiella*, the remaining *Lamontichthys*, *Aposturisoma*, *Pterosturisoma*, *Cteniloricaria*, some *Farlowella*, and some Loricariini have a short, narrow process (state 1). The remaining *Harttia*, *Farlowella*, and Loricariini, and all *Sturisoma*, have a short, broad process (state 2; [36: fig 37B]); the observations regarding the latter agree with those of Ghazzi [36].

143. Cleithrum, symphysis, length: (0) similar to coracoid symphysis; (1) half that of coracoid symphysis; (2) twice as long as coracoid symphysis. CI = 0.09. [28] Ch. 142, [38] Ch. 61.

The length of the cleithrum symphysis varies across the taxa examined. The mid-ventral symphysis can be either similar (state 0) as seen in some *Farlowella* and Loricariini, half length (state 1) as in Harttiini and most Farlowellini, or twice as long (state 2) as the coracoid symphysis, which was present in outgroups and most Loricariini.

144. Cleithrum, anterolateral process: (0) small; (1) large. CI = 0.14. [28] Ch. 141.

Loricariids have an anterolateral processes on the cleithrum. This structure can be small (state 0; Fig 16E and 16F) as seen in Harttiini and some outgroups; or large and anteriorly oriented (state 1; Fig 16A–16D) in most Loricariinae.

145. Coracoid, ventrolateral portion, bony lamina: (0) absent; (1) present. CI = 0.33. [38] Ch. 62.

*Hemipsilichthys* and the Harttiini lack a bony lamina in the lateroventral portion of the coracoid, that runs parallel to the arrector bridge and articulates to the cleithrum (state 0; Fig 16E and 16F); which is present in other Loricariinae (state 1; Fig 16A–16D).

146. Coracoid and cleithrum, symphysis, fenestra: (0) absent; (1) present. CI = 0.04. [28] Ch. 143, [37] Ch. 131.

A small fenestra at the midventral symphysis between the coracoid and the cleithrum is absent (state 0; Fig 16D) in outgroups, some Loricariini and *Farlowella*, and a few *Harttia* and *Sturisomatichthys*, or is present (state 1; Fig 16A–16C, 16E and 16F) in remaining taxa examined.

### Pelvic girdle

147. Basipterygium, symphysis cartilage plug, size: (0) large; (1) small. CI = 0.50. [28] Ch. 148.

**Fig 17:**
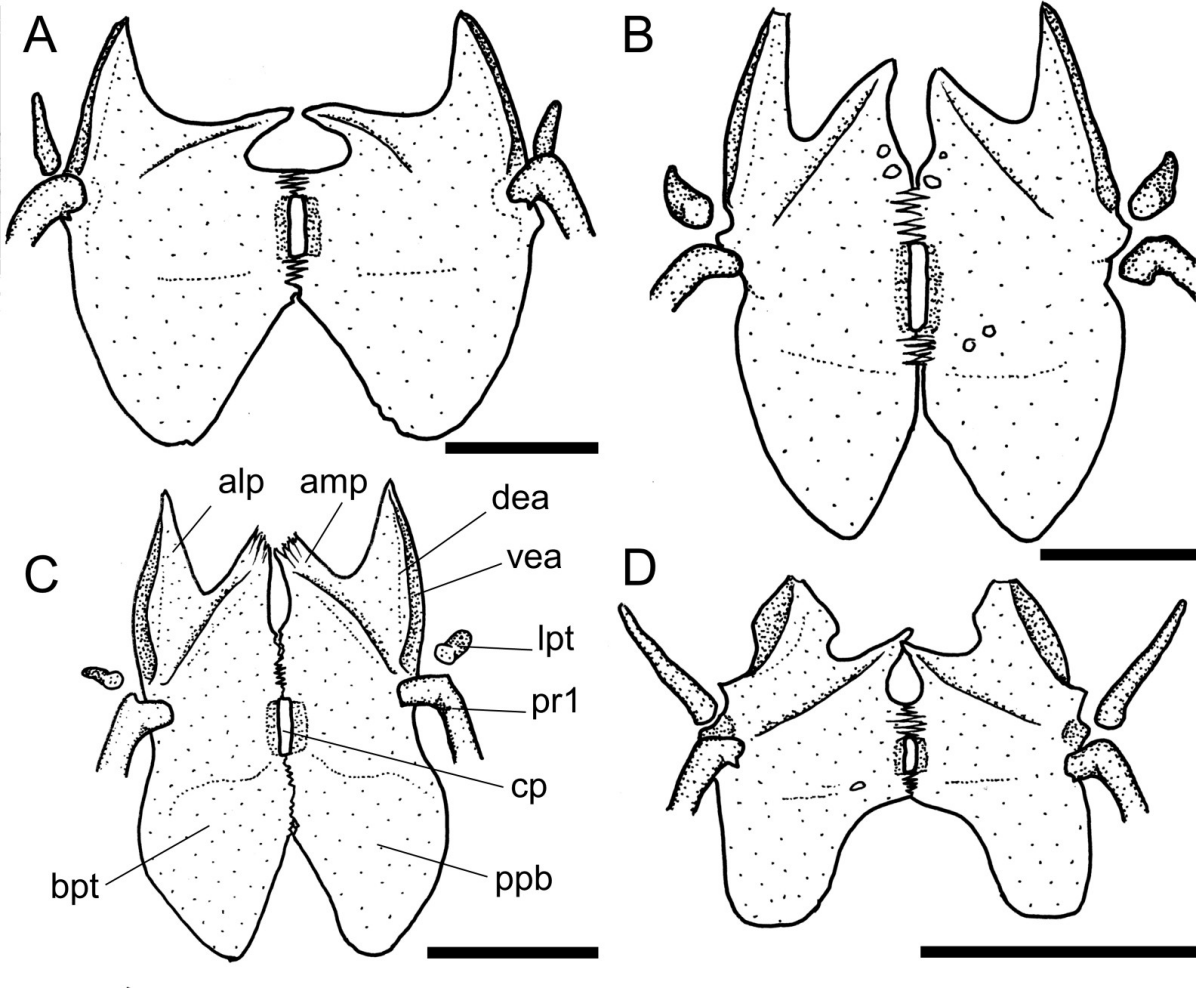
Pelvic girdle in ventral view. **A.** *Harttia gracilis*, MZUSP 99678; **B.** *Sturisoma nigrirostrum*, ANSP 199936; **C.** *Sturisomatichthys reinae*, NRM 15155; **D.** *Harttiella crassicauda*, AUM 50837. **Abbreviations:** alp = anterolateral process of basipterygium; amp = anteromesial process of basipterygium; bpt = basipterygium; cp = cartilage plug; dea = dorsal expansion of anterolateral process; ltp = lateropterygium; ppb = posterior process of basipterygium; pr1 = first pelvic-fin ray; vea = ventral expansion of anterolateral process. Scale bar 5 mm.

In *Harttia* and *Ctenoloricaria*, most Loricariini, and outgroups, except for some Hypoptopomatinae, part of the connection between contralateral basipterygia is made through a large cartilage plug (state 0; Fig 17A–17C). In *Harttiella* and *Metaloricaria* the cartilage plug is small (state 1; Fig 17D).

148. Basipterygium, anteromesial processes, anterior contact: (0) in contact along their entire medial margins; (1) in contact anteriorly and posteriorly at midline, with small fenestra in between; (2) distal tip of each process close to each other but not in contact; (-) inapplicable. CI = 0.20. [28] Ch. 150, [38] Ch. 63.

The shape and orientation of the anteromesial processes of the basypterigium result in a variation of degree of contact between these contralateral structures. They can be in contact along the entire medial margins (state 0) in outgroups, *Sturisoma*, and *Metaloricaria*, in contact anteriorly and posteriorly, resulting in a fenestra in the middle (state 1; Fig 17D) in *Farlowella*, *Harttiella*, *Dasyloricaria*, *Limatulichthys griseus*, *Loricaria lundbergi*, and *Rineloricaria lanceolata*, or with tips close to each other but not in contact (state 2; Fig 17A–17C) in *Harttia*, *Lamontichthys*, *Pterosturisoma*, *Sturisomatichthys*, and remaining Loricariini. *Hemipsilichthys gobio* lacks anteromesial processes [16], and thus was coded as inapplicable.

149. Basipterygium, symphysis, anterior and posterior bony articulations: (0) sutures anterior and posterior to cartilage plug; (1) suture posterior to cartilage plug only; (2) suture anterior to cartilage plug only. CI = 0.28. [28] Ch. 149 (modified).

Harttiini, Farlowellini except some *Farlowella*, Loricariini (except *Loricariichthys* and *Metaloricaria*), and most outgroups, have sutures anterior and posterior to the cartilage plug in the basipterygia symphysis (state 0; Fig 17A–17D). *Farlowella hasemani*, *F*. *henriquei*, *F*. *nattereri*, and *F*. *venezuelensis* are the only taxa that have a sutural contact only posterior to the cartilage (state 1). *Sturisomatichthys leightoni*, and *Loricariichthys* have a sutural contact only anterior to the cartilage (state 2).

150. Basipterygium, cartilage, shape: (0) squarish; (1) short rectangle; (2) long rectangle; (3) reduced. CI = 0.15. [28] Ch. 148, [37] Ch. 117.

Depending on the length of the symphysis of the basipterygia, the length of the associated cartilage plug varies as well. Such cartilage was found to be approximately square (state 0) in *Hemipsilichthys gobio*, *Pterygoplichthys lituratus*, and *Sturisomatichthys aureus*, a short rectangle (state 1; Fig 17D) in *Ancistrus brevipinnis*, *Chaetostoma breve*, *Neoplecostomus microps*, Harttiini, *Farlowella henriquei*, *F*. *knerii*, *F*. *mariaelenae*, *F*. *reticulata*, *F*. *rugosa*, *F*. *smithi*, *Pterosturisoma*, *Sturisomatichthys citurensis*, *S*. *festivus*, *S*. *leightoni*, *S*. *reinae*, *S*. *tamanae*, and *S*. *varii*, a long rectangle (state 2; Fig 17A–17C) in *Aposturisoma myriodon*, *Lamontichthys*, *Sturisoma*, and remaining *Farlowella*, *Sturisomatichthys*, and in Loricariini, or reduced (state 3) in *Metaloricaria*.

151. Basipterygium, anteromesial process, orientation: (0) anteromesial; (1) mesial; (-) inaplicable. CI = 0.50. [28] Ch. 152, [38] Ch. 64.

*Harttia* and *Cteniloricaria* have a strong mesial orientation of the anteromesial process (state 1; Fig 17A). The remaining taxa examined have an anteromesial orientation (state 0; Fig 17B–17D). *Hemipsilichthys gobio* was coded as inapplicable for this character since the anteromesial process is lacking [16].

152. Basipterygium, anterolateral process, shape: (0) long and thin; (1) round and broad. CI = 0.12.

Most Loricariini and outgroups, have long, thin anterolateral processes of the basipterygium (state 0; Fig 17B and 17C). Harttiini, *Farlowella oxyrryncha*, *Sturisomatichthys citurensis*, *Rineloricaria lanceolata*, *Pseudohemiodon lamina*, and Hypoptopomatinae have broad anterolateral process (state 1; Fig 17A and 17D).

153. Basipterygium, anterolateral processes, orientation: (0) remote from each other; (1) converging mesially, but not articulated; (2) converging mesially, contacting each other in the middle, forming a fenestra. CI = 0.50. [28] Ch. 151, [36] Ch. 113, [78] Ch. 49.

Harttiini, Loricariini (except *Loricaria lundbergi* and *Pseudohemiodon lamina*), and *Pterygoplichthys lituratus* have the anterolateral processes remote from each other (state 0; Fig 17A–17D). *Ancistrus brevipinnis* and *Chaetostoma breve* have the anterolateral processes converging mesially, but not articulated (state 1). Hypoptopomatinae, *L*. *lundbergi*, and *P*. *lamina* have the processes converging mesially with their tips fused and forming one or more fenestrae (state 2).

154. Basipterygium, anterolateral process, laminar expansions, relative width: (0) poorly-developed; (1) dorsal wider than ventral; (2) dorsal and ventral of similar width; (3) ventral wider than dorsal. CI = 0.30. [28] Ch. 155, [36] Ch. 115, [37] Ch. 125, [38] Ch. 65.

*Acestridium scutatum*, *Hemipsilichthys gobio*, and *Hisonotus laevior* have poorly-developed laminar expansions in the anterolateral process of the basipterygium (state 0). Among the Harttiini and Farlowellini, as well as the Hypostominae, and *Parotocinclus maculicauda*, the dorsal expansion is wider than ventral (state 1), or dorsal and ventral expansions are of similar width (state 2). Only the Loricariini have the ventral wider than dorsal (state 3).

155. Basipterygium, posterior process, shape: (0) round; (1) lanceolate; (2) long, thin and pointed; (3) short, slightly triangular. CI = 0.14. [28] Ch. 153, [37] Ch. 122.

The posterolateral process of the basipterygium varies in shape, being round (state 0; Fig 17B and 17D) in some outgroups, *Cteniloricaria*, *Harttia carvalhoi*, *H*. *duriventris*, *H*. *guianensis*, *H*. *kronei*, *H*. *longipinna*, *H*. *novalimensis*, *Harttiella*, *Lamontichthys*, and most *Sturisoma*, *Sturisomatichthys*, and Loricariini, lanceolate (state 1; Fig 17C) in *Farlowella*, *Sturisoma barbatum*, *St*. *guentheri*, *St*. *robustum*, *St*. *rostratum*, *Sturisomatichthys citurensis*, *S*. *dariensis*, *S*. *panamensis*, *S*. *reinae*, *S*. *varii*, and *Loricaria lundbergi*, long, thin, and pointed (state 2) in *Acestridium scutatum*, *Hisonotus laevior*, and *Parotocinclus maculicauda*, or short and slightly triangular (state 3; Fig 17A) in remaining *Harttia*, *Limatulichthys griseus*, and *Pseudohemiodon lamina*.

156. Lateropterygium: (0) present; (1) absent. CI = 0.06. [7] Ch. 227, [28] Ch. 157, [37] Ch. 124, [38] Ch. 66, [79] (modified).

The lateropterygium can be present (state 0; Fig 17A–17D) in most taxa examined, or absent (state 1) in *Cteniloricaria*, *Farlowella acus*, *F*. *amazonum*, *F*. *curtirostra*, *F*. *isbruckeri*, *F*. *reticulata*, *Harttia fluminensis*, *H*. *fowleri*, *H*. *guianensis*, *H*. *trombetensis*, *Acestridium scutatum*, *Dasyloricaria latiura*, *Hemiodontichthys acipenserinus*, *Limatulichthy griseus*, *Rineloricaria cadeae*, and *R*. *lanceolata*. Previous authors included both the presence and the shape of the lateropterygium in the same character. These features were treated as two separate characters here.

157. Lateropterygium, shape: (0) stick-shaped: (1) mushroom-shaped; (2) triangular; (-) inapplicable. CI = 0.12. [7] Ch. 227, [28] Ch. 157, [37] Ch. 124, [38] Ch. 66, [74] (modified).

The lateropterygium is a bony structure associated with the lateral musculature and first pelvicfin ray [28]. Such structure, present in both the Astroblepidae and the Loricariidae, shows great variation being stick-shaped (state 0; Fig 17A and 17D) in most outgroups, *Harttiella*, *Lamontichthys*, *Metaloricaria*, and most *Harttia*, mushroom-shaped (state 1; Fig 17C) in *Farlowella hasemani*, *F*. *jauruensis*, *F*. *oxyrryncha*, *F*. *paraguayensis*, *F*. aff. *amazonum*, *F*. *smithi*, *F*. *vittata*, *Sturisoma robustum*, *Sturisomatichthys aureus*, *S*. *dariensis*, *S*. *frenatus*, S. *leightoni*, *S*. *reinae*, *S*. *tamanae*, *S*. *varii*, *Loricariichthys*, *Dasyloricaria filamentosa*, and *D*. *pauciquama*, or triangular (state 2; Fig 17B) in Harttiini and remaining Farlowellini. Some taxa lack a lateropterygium, and they were coded as inapplicable.

### Caudal and anal fins

158. Anal fin, first pterygiophore, centrum of articulation: (0) centrum 15; (1) centra 12, 13 or 14. CI = 1.00. [38] Ch. 68.

Outside Loricariinae the articulation of the first anal-fin pterygiophore occurs on the 15th vertebral centrum (state 0). In the Loricariinae, as an exclusive synapomorphy, the articulation occurs more anteriorly, on the 12^th^ to 14^th^ vertebral centrum (state 1), a result in agreement with those of Paixão and Toledo-Piza [38].

159. Anal fin, anterior three pterygiophores, adjacent proximal portions, relative distance: (0) well separated; (1) relatively close; (2) in contact. CI = 0.11. [28] Ch. 122, [38] Ch. 69.

*Harttiella*, *Sturisoma*, most Loricariini, and outgroups have well-separated proximal portions of first three anal-fin pterygiophores (state 0). Most Harttiini and Farlowellini have the proximal portions of first three anal-fin pterygiophores relatively close (state 1) or in contact (state 2).

160. Hypural plate, upper and lower lobes, shape: (0) symmetric, posterior border vertically aligned; (1) asymmetric, lower lobe extending beyond posterior margin of upper lobe; (2) symmetric, posterior border *V*-shaped. CI = 0.50. [38] Ch. 71, [41] Ch. 21 (modified).

In *Farlowella* and outgroups except Hypostominae, the general condition is upper and lower lobes of the hypural plate symmetric, with a vertically aligned posterior border (state 0; [28: fig 35A]). Hypostominae have asymmetric upper and lower lobes, with the lower lobe extending beyond the margin of the upper lobe (state 1; [28: fig 36C]). In the Harttiini, Loricariini, and most Farlowellini, a symmetric, *V*-shaped hypural plate, is present (state 2; [28: fig 39E]).

161. Hypurapophysis, shape: (0) relatively elongate, keel-shaped; (1) broad, laminar, wing-shaped; (2) robust, approximately triangular; (3) robust, approximately square. CI = 0.10. [28] Ch. 129, [38] Ch. 72, [41 80].

The hypurapophysis is a result of the fusion of elements related to the hypural plates. In loricariids, due to the high degree of fusion of bony elements, there is considerable variation regarding the shape of such structure. The hypurapophysis was observed to be relatively elongate, keel-shaped (state 0) in most outgroups, *Cteniloricaria*, *Farlowella henriquei*, *F*. *jauruensis*, *F*. *knerii*, *F*. *nattereri*, *F*. *paraguayensis*, *F*. aff. *amazonum*, *F*. *reticulata*, *F*. *rugosa*, *F*. *schreitmuelleri*, *F*. *venezuelensis*, *F*. *vittata*, *Harttia dissidens*, *H*. *duriventris*, *H*. *fowleri*, *H*. *gracilis*, *H*. *guianensis*, *H*. *rhombocephala*, *Harttiella longicauda*, *Metaloricaria*, *Pterosturisoma*, *Sturisoma robustum*, *Sturisomatichthys dariensis*, and most Loricariini, broad, laminar, wing-shaped (state 1) in *Aposturisoma myriodon*, *Farlowella acus*, *F*. *amazonum*, *F*. *curtirostra*, *F*. *hahni*, *F*. *isbruckeri*, *F*. *mariaelenae*, *F*. *smithi*, *Sturisomatichthys*, most *Sturisoma*, *Limatulichthys griseus*, *Rineloricaria lanceolata*, and *Spatuloricaria puganensis*, robust, triangular (state 2) in *Harttiella crassicauda*, *Sturisoma barbatum*, *St*. *graffini*, *Dasyloricaria*, and remaining *Farlowella*, or robust, squarish (state 3) in outgroups *Ancistrus brevipinnis* and *Pterygoplichthys lituratus*.

162. Hypurapophysis, relative length: (0) short, not projecting to second preural centrum; (1) long, projecting beyond second preural centrum. CI = 0.06. [38] Ch. 73.

The hypurapophysis among loricariids can be short, not projecting to the second preural centrum (state 0) which was the more generalized state found here, or long, projecting to the second preural centrum (state 1) in a few Harttiini, Farlowellini, and Loricariini.

163. Epural, size: (0) large, length approximately equal to height of lower lobe of hypural plate; (1) small, length shorter than half height of lower lobe of hypural plate; (2) not a separate element. CI = 0.33. [28] Ch. 127, [38] Ch. 75, [41].

Taxa belonging to the outgroup, except Hypostominae, have a large epural, which is approximately equal in height to the lower lobe of the hypural plate (state 0). Hypostominae, *Pseudohemiodon lamina*, *Dasyloricaria*, *Loricariichthys*, *Metaloricaria*, and *Rineloricaria* have a small epural, of length shorter than half the height of the lower lobe of the hypural plate (state 1). Harttiini, Farlowellini, and remaining Loricariini lack the epural as a separate element (state 2).

164. Second preural centrum, apophysis: (0) one, low; (1) two, well developed; (2) absent. CI = 0.10. [28] Ch. 130, [36] Ch. 92.

The second preural centrum can exhibit one or two projections that are called apophyses. When present it can be only one, low (state 0; [28: fig 35B]) in most outgroups, *Farlowella henriquei*, *F*. *isbruckeri*, *F*. *knerii*, *F*. *mariaelenae*, *F*. *oxyrryncha*, *F*. *reticulata*, *F*. *schreitmuelleri*, *F*. *smithi*, *F*. *venezuelensis*, *Sturisomatichthys dariensis*, *S*. *festivus*, *S*. *frenatus*, *Dasyloricaria latiura*, *Limatulichthys griseus*, *Pseudohemiodon lamina*, *Rineloricaria lanceolata*, and *Spatuloricaria puganensis*, or two, well developed (state 1; [28: fig 37B]) in *Farlowella nattereri*, *Lamontichthys*, *Loricaria lundbergi*, and remaining *Dasyloricaria* and *Rineloricaria*, or it can be absent (state 2) in outgroups *Neoplecostomus microps*, *Pareiorhaphis calmoni*, *Parotocinclus maculicauda*, and *Pterygoplichthys lituratus*, and in *Aposturisoma myriodon*, Harttiini, *Metaloricaria*, *Sturisoma*, *Loricariichthys*, *Hemiodontichthys acipenserinus*, and remaining *Sturisomatichthys*.

165. Second preural centrum, neural and hemal spines: (0) dorsally and ventrally expanded; (1) poorly or not expanded. CI = 0.12. [27] Ch. 29, [28] Ch. 133, [36] Ch. 93.

The second preural centrum bears neural and hemal spines, which are respectively dorsally and ventrally expanded (state 0) in outgroups, in most *Harttia*, *Harttiella* and *Lamontichthys*, or poorly expanded or not expanded (state 1) in most Farlowellini and Loricariini.

166. Second preural centrum, length: (0) much shorter than hypural plate; (1) equal to or slightly longer than hypural plate; (2) slightly shorter than hypural plate. CI = 0.11. [28] Ch. 136, [36] Ch. 96, [41].

The second preural centrum itself showed variation regarding its length. It can be much shorter than the hypural plate (state 0) in some outgroups and most *Harttia*, equal to or slightly longer than the hypural plate (state 1) in most Farlowellini, or slightly shorter than the hypural plate (state 2) in Loricariini, *Sturisoma* and few outgroups.

167. Hypural plates, posterior margin: (0) notch, no fenestra; (1) notch and fenestra; (2) notch and fenestra absent. CI = 0.07. [27] Ch. 34, [28] Ch. 126, [36] Ch. 89.

The principal caudal-fin rays articulate to the distal border of the fused hypurals, which usually has a notch at the junction between upper and lower hypural plates [28]. This notch is variably followed internally by a caudal fenestra between upper and lower hypural plates [27, 28]. Among taxa examined a deep notch and no fenestra (state 0) occurs in part of the outgroup, some Harttiini, and *Sturisomatichthys*. A variably deep notch and a fenestra (state 1) occurs in most Farlowellini and some Loricariini, and the lack of a fenestra and notch (or very shallow notch) (state 2) is found in most *Farlowella*, some Loricariini, and some outgroups.

### External morphology

168. Snout shape: (0) short, round; (1) short, triangular; (2) elongate. CI = 0.20. [28] Ch. 185.

**Fig 18:**
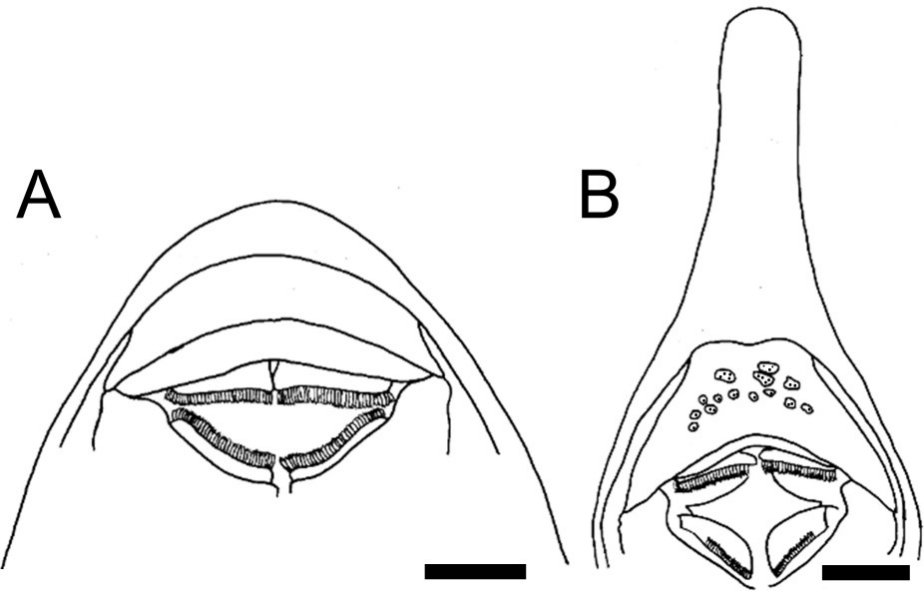
Head in ventral view, upper lip showing absence/presence of dermal plates. **A.** *Cteniloricaria platystoma*, AUM 48174; **B.** *Farlowella acus*, ANSP 130038. Scale bar 2 mm.

The outgroups, Harttiini, *Sturisomatichthys citurensis*, and *Rineloricaria lanceolata* have a round snout in dorsal view (state 0; Figs 3A, 3B and 18A). *Aposturisoma*, *Farlowella curtirostra*, *Pterosturisoma*, *Sturisomatichthys tamanae*, and the remaining Loricariini, have a triangular snout (state 1; Fig 3C). *Sturisoma*, the remaining *Sturisomatichthys* and *Farlowella* have an elongated snout (state 2; Fig 18B).

169. Groove on snout, anterior to nostril: (0) present, anterior portion formed by distinct skin fold; (1) present, anterior portion not formed by skin fold; (2) absent. CI = 0.15. [38] Ch. 79.

Loricariids may possess a groove on the snout, which runs from the anteriormost point of each nostril to the anteroventral border of the snout; the posterior portion of that groove runs between dermal plates or on the plates, and possess enlarged odontodes along its margins [38: fig 52]. Such groove was observed among taxa examined and can be present, with the anterior portion formed by distinct skin fold (state 0) in outgroups, *Harttia*, *Harttiella*, and most *Lamontichthys*, present, with the anterior portion not formed by skin fold (state 1) in *Aposturisoma myriodon*, *Metaloricaria paucidens*, *Sturisoma*, *Acestridium scutatum*, and most *Farlowella*, *Sturisomatichthys*, and Loricariini, or absent (state 2) in *Cteniloricaria*, *Farlowella curtirostra*, *Lamontichthys avacanoeiro*, *Metaloricaria nijsseni*, *Pterosturisoma*, *Sturisomatichthys leightoni*, and *Hemiodontichthys acipenserinus*.

170. Upper lip, margin, central portion: (0) fringed, with papillae, (1) smooth, no papillae, (2) with filaments. CI = 0.40. [36] Ch. 124.

Most Harttiini, Loricariini, and outgroups have a fringed upper lip margin, which is bordered by papillae (state 0). *Lamontichthys avacanoeiro*, *L*. *parakana*, *Sturisoma lyra*, *Loricariichthys*, *Hemiodontichthys acipenserinus*, *Limatulichthys griseus*, and *Spatuloricaria puganensis* have a smooth central margin, without papillae (state 1). Filaments on the upper lip margin were observed in *Loricaria lundbergi*, and *Pseudohemiodon lamina* (state 2).

171. Upper lip, dermal plates: (0) absent; (1) present. CI = 0.50. [28] Ch. 176, [38] Ch. 81 (modified).

Within Loricariinae, only Farlowellini and *Metaloricaria* have small dermal plates on the upper lip (state 1; Fig 18B). Paixão and Toledo-Piza [38] also reported this for the former.

172. Rictal barbel, length: (0) approximately one orbit diameter; (1) half orbit diameter; (2) longer than one orbit diameter; (3) absent. CI = 0.13. [28] Ch. 178, [36] Ch. 125, [37] Ch. 139.

The rictal barbel is located laterally at the junction of the upper and lower lips. Such barbel was found to be equal to or slightly shorter than one orbit diameter (state 0) in most Farlowellini and some outgroups, half length of one orbit diameter (state 1) in Loricariini and most *Sturisoma*, longer than one orbit diameter (state 2) in most Loricariini, or absent (state 3) in most Harttiini and *Farlowella*.

173. Central buccal papillae, size: (0) absent; (1) present, small; (2) present, large. CI = 0.20. [28] Ch. 182, [38] Ch. 82 (modified).

Among the Loricariinae there is great variation regarding the ornamentation, and size of the central buccal papillae. Such structure can vary in size, and number of papillae, and according to several authors [28, 36, 38, 39] such variation is useful for the differentiation of several groups within the subfamily (as observed in this study). Here, the Harttiini, most Loricariini, and outgroups were observed to lack buccal papillae (state 0). *Sturisoma*, *Sturisomatichthys*, except *S*. *citurensis* and *S*. *tamanae*, some *Farlowella*, and *Limatulichthys griseus* have small central buccal papillae (state 1; [38: fig 9]). Large papillae (state 2) were found in *Loricaria lundbergi*, and *Pseudohemiodon lamina*.

174. Lower lip, margin, ornamentation: (0) poorly-developed papillae; (1) well-developed papillae; (2) filaments; (3) smooth. CI = 0.25. [28] Ch. 177.

The margin of the lower lip shows considerable variation among the Loricariidae. The lower-lip margin can bear poor-developed papillae (state 0) in *Harttia*, *Harttiella*, and some *Farlowella*, well-developed papillae (state 1) in Loricariini and Farlowellini, have elongate filaments (state 2) in *Loricaria lundbergi*, and *Pseudohemiodon lamina*, or be smooth, with no papillae or filaments (state 3) as in *Hemiodontichthys acipenserinus*.

175. Nuchal plate, articulation to surrounding plates: (0) not articulated; (1) articulated to lateral plates; (2) articulated to lateral and predorsal plates. CI = 0.50.

**Fig 19:**
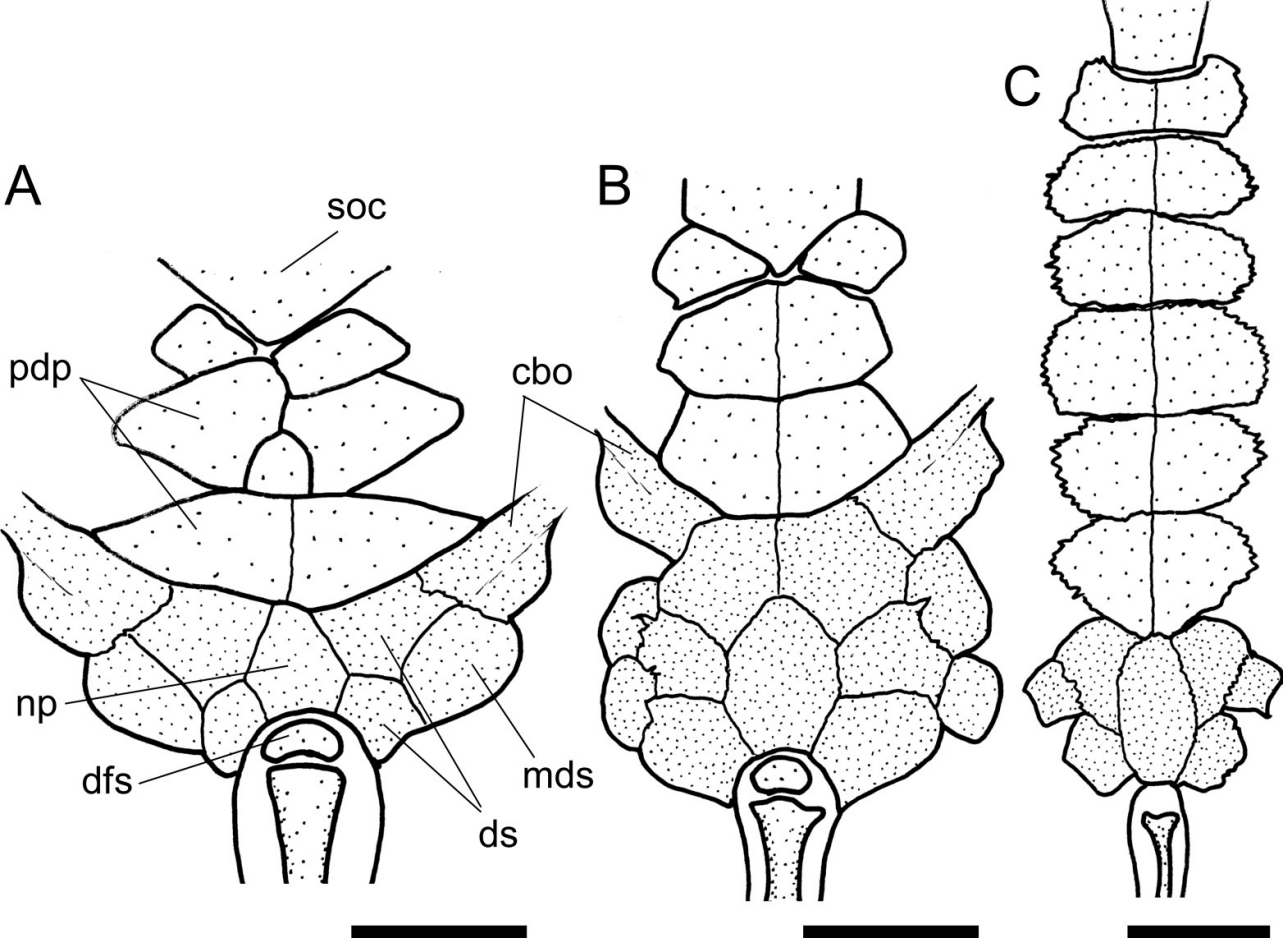
Predorsal region in dorsal view. **A.** *Lamontichthys avacanoeiro*, MNRJ 18553; **B.** *Sturisomatichthys reinae*, NRM 15155; **C.** *Farlowella schreitmuelleri*, FMNH 106985. **Abbreviations:** cbo = connecting bone; dfs = dorsal-fin spinelet; ds = dorsal series of plates; mds = middorsal series of plates; np = nuchal plate; pdp = predorsal plate; soc = parieto-supraoccipital. Scale bar 5 mm.

The nuchal plate is located posterior to the predorsal plates, immediately in front of the dorsal fin, and is connected to the neural spine of the seventh centrum. In most members of the Loricariidae the nuchal plate is not strongly sutured to adjacent plates (state 0). In the Farlowellini, the nuchal plate is firmly sutured to adjacent plates and to the connecting bone (when present), forming a strong shield in front of the dorsal fin. In *Lamontichthys* and *Farlowella* the nuchal plate is sutured to two plates of the dorsal lateral series and one plate of the middorsal series (state 1; Fig 19A and 19C). In *Sturisoma* and *Sturisomatichthys* the posteriormost predorsal plate and two middorsal plates are also firmly sutured to the shield (state 2; Fig 19B).

176. Predorsal plates, shape: (0) rectangular; (1) polygonal; (2) semi-trapezoidal. CI = 0.12. [27], [37] Ch. 151 (modified).

Fewer informative states were found throughout terminal taxa included in this study; previous authors found a higher variation in their samples. Predorsal plates can be rectangular (state 0) in outgroups *Ancistrus brevipinnis*, *Pterygoplichthys lituratus*, and *Parotocinclus maculicauda*, and in *Aposturisoma myriodon*, *Cteniloricaria*, *Pterosturisoma*, *Metaloricaria*, *Sturisomatichthys panamensis*, *Loricariichthys*, and *Rineloricaria lanceolata*; polygonal (state 1) in *Harttia*, *Harttiella crassicauda*, most *Lamontichthys*, *Sturisomatichthys dariensis*, and *S*. *frenatus*; or semi-trapezoidal (state 2) in *Farlowella*, *Harttia kronei*, *Harttiella longicauda*, *Lamontichthys avacanoeiro*, *L*. *parakana*, *Sturisoma*, remaining *Sturisomatichthys*, and Loricariini.

177. Dorsal-fin base, number of flanking plates: (0) five; (1) four; (2) six; (3) three; (4) more than six. CI = 0.23. [37] Ch. 167, [76].

Plates flanking the dorsal-fin base in loricariids can be five (state 0) as in most Loricariini, and some Harttiini and Farlowellini, four (state 1) in *Lamontichthys* and *Sturisomatichthys*, six (state 2) in some *Harttia* and Loricariini, three (state 3) in *Farlowella*, or more than six (state 4) in most outgroups. Three plates flanking the dorsal fin (state 3) is an exclusive synapomorphy of *Farlowella*.

178. Central abdominal plates: (0) absent; (1) present. CI = 0.12. [28] Ch. 189, [37] Ch. 155 (modified).

**Fig 20:**
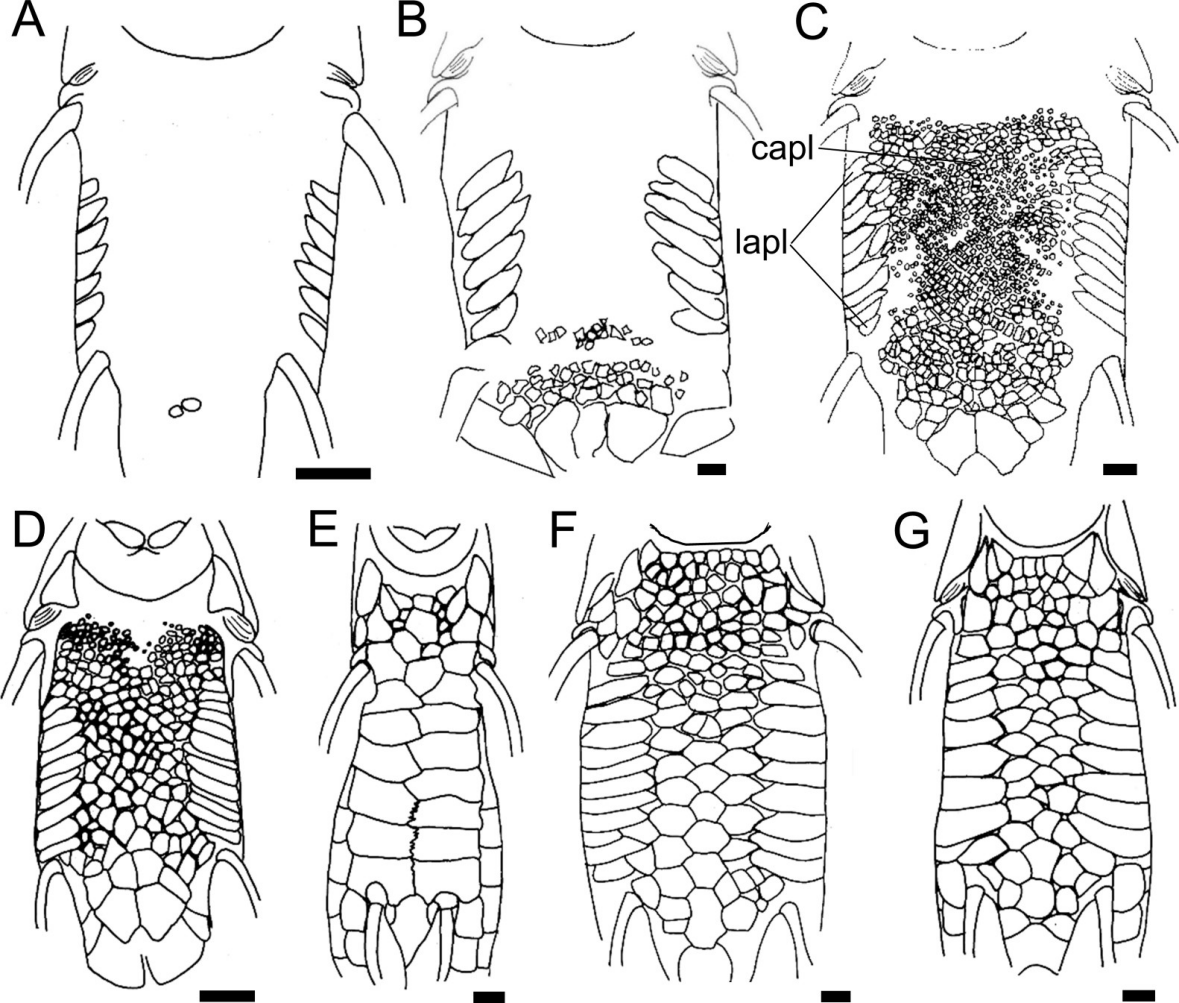
Abdominal plates arrangement. **A.** *Harttiella crassicauda*, AUM 50392; **B.** *Harttia punctata*, MCP 45591; **C.** *Harttia rhombocephala*, MCP 16007; **D.** *Cteniloricaria platystoma*, AUM 48714; **E.** *Farlowella acus*, ANSP 130038; **F.** *Sturisoma graffini*, USNM 319351; **G.** *Sturisomatichthys varii*, FMNH 58333. **Abbreviations:** lapl = lateral abdominal plates; capl = central abdominal plates. Scale bar 5 mm.

Abdominal plates are usually termed lateral abdominal plates, which are transversely elongated plates between the pectoral-fin axilla and the pelvic-fin insertion, and central abdominal plates, which cover the abdomen between the lateral ones. In this character only the absence (state 0; Fig 20A) in *Hemipsilichthys gobio*, *Pareiorhaphis calmoni*, Hypostominae, *Harttia carvalhoi*, *H*. *fluminensis*, *H*. *garavelloi*, *H*. *gracilis*, *H*. *guianensis*, *H*. *kronei*, *H*. *leiopleura*, *H*. *loricariformis*, *H*. *novalimensis*, *H*. *punctata*, *H*. *torrenticola*, and *Harttiella*, or the presence (state 1; Fig 20B–20G) in other outgroups, Farlowellini, Loricariini, and remaining Harttiini of central abdominal plates in different configurations was coded. Previous authors used intermediate states regarding the type of central abdominal covering (i.e. plates partially covering abdomen).

179. Gular plates: (0) absent; (1) present. CI = 1.00.

Gular plates are large, polygonal dermal plates covering the ventral surface of the head behind the lower lip. The outgroup, Loricariini, and Harttiini lack such plates (state 0; Fig 20A–20D). The Farlowellini, on the other hand, have polygonal gular plates that may reach the posterior border of lower lip (state 1; Fig 20E–20G).

180. Central abdominal plates, shape: (0) patches of odontodes only; (1) very small, irregular, closely packed; (2) small, roughly quadrangular but slightly separated; (3) large, quadrangular; (-) inapplicable. CI = 0.30. [28] Ch. 190, [39] Ch. 88 (modified).

Different shapes of plates are used herein when compared to previous studies. Fewer states were found to be informative. The shape of central abdominal plates in species examined vary among the following conditions: patches of lose odontodes on the abdominal skin (state 0) in *Neoplecostomus microps*, *Hisonotus laevior*, *Harttia trombetensis*, *Spatuloricaria puganensis*, and *Pseudohemiodon lamina*; very small, irregular, and closely packed platelets (state 1; Fig 20C and 20D) in *Cteniloricaria*, *Harttia dissidens*, *H*. *duriventrus*, *H*. *fowleri*, *H*. *longipinna*, *H*. *rhombocephala*, and *Lamontichthys*; small, roughly quadrangular but slightly separated plates (state 2; Fig 20F and 20G) in *Acestridium scutatum*, *Parotocinclus maculicauda*, *Pterosturisoma*, *Metaloricaria*, *Dasyloricaria*, *Loricaria lundbergi*, *Loricariichthys anus*, and *Rineloricaria*; and large, quadrangular plates (state 3; Fig 20E) in *Aposturisoma myriodon*, *Farlowella*, *Sturisoma*, *Sturisomatichthys*, *Hemiodontichthys acipenserinus*, *Limatulichthys griseus*, and *Loricariichthys platymetopon*. The character is inapplicable to species without central abdominal plates (see character 178).

181. Central abdominal plates, arrangement: (0) scattered, not arranged in series; (1) arranged in regular series; (-) inapplicable. CI = 0.20. [28] Ch. 191 (modified).

Among the Harttiini and Farlowellini, only *Aposturisoma*, some *Farlowella*, and *Sturisoma* have abdominal plates clearly arranged in series (state 1; Fig 20F). The character is inapplicable to species without central abdominal plates.

182. Lateral abdominal plates, shape: (0) absent; (1) present, flat; (2) present, angled. CI = 0.18. [28] Ch. 188.

The lateral abdominal plates, located between the pectoral-fin axilla and the origin of the pelvic fin, can be either absent (state 0) in *Hemipsilichthys gobio*, Hypostominae, and some Hypoptopomatinae examined, present and flat, not bent (state 1) in *Acestridium scutatum*, *Parotocinclus maculicauda*, *Farlowella* aff. *amazonum*, *Harttia carvalhoi*, *H*. *kronei*, *Harttiella*, *Dasyloricaria latiura*, *Loricaria lundbergi*, *Pseudohemiodon lamina*, *Rineloricaria cadeae*, *R*. *lanceolata*, and *Spatuloricaria puganensis*, or present, angled, bent at the lateral border of the abdomen forming a keel (state 2) in *Aposturisoma myriodon*, *Cteniloricaria*, *Lamontichthys*, *Pterosturisoma*, *Sturisoma*, *Sturisomatichthys*, and remaining *Farlowella*, *Harttia*, and Loricariini.

183. Dorsal-fin unbranched ray, long filament at distal tip: (0) absent; (1) present. CI = 0.33. [38] Ch. 87.

In addition to the taxa reported by Paixão and Toledo-Piza [38] to have a filament on the dorsal fin (*Lamontichthys filamentosus*, *L*. *llanero*, and *Sturisoma robustum*), it was also observed in *Sturisomatichthys festivus*.

184. Pectoral girdle, exposure: (0) covered by skin or plates; (1) exposed on ventral surface. CI = 0.50.

Among the Loricariidae, members of Hypoptopomatinae have a variably exposed pectoral girdle on the ventral surface, which support odontodes. Among the taxa examined *Acestridium scutatum*, *Parotocinclus maculicauda*, and *Hisonotus laevior* have the pectoral girdle exposed ventrally (state 1).

185. Pelvic fin, length: (0) not surpassing anal-fin origin; (1) slightly surpassing anal-fin origin; (2) conspicuously surpassing anal-fin origin. CI = 0.09. [35] Ch. 171.

The distal tip of the pelvic fin may not reach the anal-fin origin (state 0) as in *Harttia*, *Farlowella*, some Loricariini, and outgroups, slightly surpass the anal-fin origin (state 1) in most Loricariini and *Sturisoma*, or conspicuously surpass the anal-fin origin (state 2) in some Farlowellini and Loricariini.

186. Pelvic fin, position: (0) on same vertical as dorsal-fin origin; (1) anterior to dorsal-fin origin; (2) posterior to dorsal-fin origin. CI = 0.22. [37] Ch. 179.

Pelvic fins can be located either on the same vertical as the dorsal-fin origin (state 0) as seen in *Sturisoma*, *Lamontichthys*, and most *Harttia*, anterior to dorsal-fin origin (state 1) in *Farlowella*, *Sturisomatichthys*, Loricariini, and some outgroups, or posterior to that point (state 2) on a few outgroups.

187. Anal plate: (0) absent; (1) present. CI = 0.11. [28] Ch. 192.

Most members of Loricariinae have an enlarged, median anal plate right in front of the anal opening (state 1; Fig 20B–20G). Conversely, some species of *Harttia*, *Hartiella*, *Loricaria lundbergi*, and *Pseudohemiodon lamina* lack an anal plate (state 0; Fig 20A).

188. Adipose fin: (0) present; (1) absent. CI = 0.33. [28] Ch. 123, [37] Ch. 46, [38] Ch. 78, [66].

Although the absence of an adipose fin is diagnostic for Loricariinae (state 1), within Loricariidae there are members of the Hypostominae, the Rhinelepinae, and the Hypoptopomatinae that convergently lack an adipose fin.

189. Caudal peduncle, width: (0) narrows gradually towards caudal-fin base; (1) narrows abruptly towards caudal-fin base. CI = 0.25.

In *Harttia* the caudal peduncle narrows abruptly at a certain point, towards the caudal-fin base (state 1; [87: fig 14]). Nevertheless, it was observed that in *H*. *leiopleura*, *H*. *torrenticola*, *H*. *carvalhoi*, and *H*. *kronei*, such condition is not as visible as in their congeners and they were coded as (state 0; [87: fig 8]).

190. Supracaudal plates; (0) short and numerous; (1) elongate and few. CI = 1.00. [28] Ch. 137, [38] Ch. 86, [41].

Contrary to other loricariids (state 0; [76: fig 16A]), the Loricariinae have few, elongate supracaudal plates (state 1; [76: fig 16B]).

191. Caudal fin, number of branched rays: (0) 14 rays; (1) 12 rays; (2) 11 rays; (3) 10 rays. CI = 0.37. [27] Ch. 35, [28] Ch. 124, [36] Ch. 87, [37] Ch. 44 (modified).

Outgroups, except *Acestridium scutatum*, have 14 branched rays in the caudal fin (state 0). The Harttiini, Farlowellini (except *Farlowella*), *Metaloricaria*, and *A*. *scutatum* have 12 caudal-fin rays (state 1). *Aposturisoma* and *Farlowella* species have either 11 (state 2) or 10 (state 3), and Loricariini has 10 caudal-fin rays.

192. Upper caudal-fin ray, filament: (0) short or no filament; (1) less than half body length; (2) exceeding body length. CI = 0.10. [37] Ch. 161.

Loricariines frequently show a filament on upper and sometimes lower caudal-fin rays; such filament can be short or absent (state 0) in most taxa examined, produced but shorter than half the body length (state 1) in *Farlowella hasemani*, *F*. *henriquei*, *F*. *jauruensis*, *F*. *mariaelenae*, *F*. *nattereri*, *F*. *paraguayensis*, *F*. *rugosa*, *F*. *schreitmuelleri*, *F*. *smithi*, *F*. *vittata*, *Lamontichthys parakana*, *Sturisoma guentheri*, *St*. *monopelte*, *St*. *robustum*, *St*. aff. *tenuirostre*, *St*. *graffini*, *Sturisomatichthys festivus*, *S*. *kneri*, *S*. *reinae*, *Dasyloricaria latiura*, *Loricaria lundbergi*, *Rineloricaria lanceolata*, and *Pseudohemiodon lamina*, or very long, exceeding the body length (state 2) in *Lamontichthys filamentosus*, *L*. *llanero*, *Pterosturisoma*, *Dasyloricaria filamentosa*, and *Spatuloricaria puganensis*.

193. Body coloration, pattern: (0) spots; (1) bars; (2) stripe; (3) plain. CI = 0.42. [36] Ch. 132, [37] Ch. 147 (modified).

Most outgroups, members of the Loricariini, and most Harttiini are spotted (state 0), or possess transverse bars on the body (state 1), the Farlowellini, except *Lamontichthys* and *Pterosturisoma* have longitudinal stripes (state 2), *Pterosturisoma* is uniformly colored, lacking spots, stripes, or bands (state 3). Previous authors further detailed the coloration pattern, resulting in additional states in this character. The simplified states used herein are more informative and illustrate the diversity of color patterns across the taxa examined.

194. Dorsal fin, dark band: (0) absent; (1) present. CI = 0.33.

A dark band on the first two or three dorsal-fin rays, including the unbranched ray (almost entire in *Sturisomatichthys frenatus*), is characteristic of *Sturisomatichthys*, *Pterosturisoma*, and *Cteniloricaria* (state 1). Remaining examined taxa lack such dark band (state 0).

195. Pectoral-fin, coloration: (0) hyaline; (1) dotted; (2) round blotch; (3) band. CI = 0.12.

Among loricariids the pectoral fin can be hyaline (state 0) in outgroups and most *Harttia*, dotted (state 1) in Loricariini, *Farlowella*, and some *Harttia*, with a single round blotch (state 2) in some Loricariini and Farlowellini, or with an enlarged band (state 3) in some *Sturisomatichthys*.

196. Caudal-fin, *V*-shaped band: (0) absent; (1) present. CI = 0.20.

A *V*-shaped band on the caudal fin was either absent (state 0) in most Loricariini, Harttiini, and outgroups, or present (state 1) in Farlowellini and a few Loricariini.

### Molecular characters

**197–2698. 12S and 16S rRNA (mitochondrial):** The 12S and 16S rRNA data includes 66 terminals with 1843–2445 base pairs after editing including only sequences from GenBank, deposited from studies of Covain et al. [15, 18]. The division of the sequences into individual genes followed information provided on the sequences deposited on GenBank, and Covain et al. [15]. The dataset encompases 2502 characters after alignment of which 1502 are conserved, 978 are variable, and 745 are parsimony informative sites.

**2699–3755. Cytb (mitochondrial):** The Cytb data includes 58 terminals with 761–1057 base pairs after editing, including sequences from GenBank. The dataset is comprised of 1057 characters after alignment of which 563 are conserved, 494 are variable, and 432 are parsimony informative sites.

**3756–4392. MyH6 (nuclear):** The MyH6 data includes 51 terminals with 601–637 base pairs after editing, including sequences from GenBank. The dataset encompasses 637 characters after alignment of which 417 are conserved, 220 are variable, and 178 are parsimony informative sites.

**4393–5106. RAG1 (nuclear):** The RAG1 data includes 75 terminals with 648–714 base pairs after editing, including sequences from GenBank. The dataset comprises 714 characters after alignment of which 481 are conserved, 233 are variable, and 152 are parsimony informative sites.

**5107–5885. RAG2 (nuclear):** The RAG2 data includes 61 terminals with 579–779 base pairs after editing, including sequences from GenBank. The dataset contains 779 characters after alignment of which 514 are conserved, 265 are variable, and 172 are parsimony informative sites.

**5886–6819. nd2 (mitochondrial):** The nd2 data includes 63 terminals with 662–934 base pairs after editing; does not include sequences from GenBank. The dataset encompasses 934 characters after alignment of which 407 are conserved, 527 are variable, and 466 are parsimony informative sites.

### Total evidence data matrix

The concatenated matrix was analyzed by Bayesian inference and maximum parsimony, under a total evidence approach. Both analyses included 100 terminals, nine belonging to the outgroup and 91 comprising the ingroup. The total number of characters is 6,819. Molecular characters include 441 sequences belonging to seven markers. Only data from the 12S and 16S mitochondrial markers were taken totally from GenBank [14, 15, 18]; sequences from the remaining markers (Cytb, MyH6, Rag1, Rag2 and nd2) are mostly novel data for the Loricariinae.

### Phylogenetic analysis: Cladistic relationships

**Fig 21:**
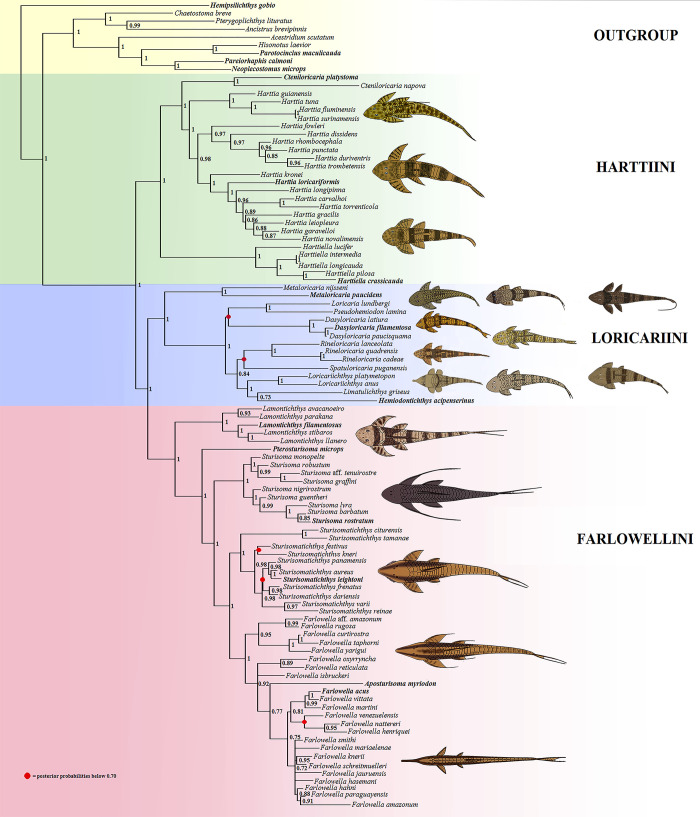
Tree obtained from Bayesian Inference analysis. Posterior Probabilities values at nodes. Type-species names in bold.

The Bayesian inference tree is shown in Fig 21, where numbers on nodes represent Posterior Probabilities (PP). The subfamily Loricariinae is corroborated as monophyletic, as are the three major clades, Harttiini, Farlowellini, and Loricariini, the latter two elevated to tribe level in the present classification. Harttiini comprises (*Harttiella* (*Cteniloricaria*, *Harttia*)), while Farlowellini is configured as (*Lamontichthys* (*Pterosturisoma* (*Sturisoma* (*Sturisomatichthys*, *Farlowella*))), with *Aposturisoma* deeply nested within *Farlowella*. A section with poor taxonomic sampling in the present analysis is the Loricariini, so the genus-level relationships recovered are disregarded. However, *Metaloricaria* was found to be sister to the remaining members of the tribe, corroborating Covain et al. [15].

A summary classification of the subfamily Loricariinae by sequencying of taxa according to Wiley [55] is given below. Genera in tribe Loricariini modified from Covain et al. [15]; new genus-groups were created to make the classification strictly phylogenetic. See (S1 File) for a complete classification with synonymy, composition, synapomorphy list, and distribution of the Harttiini and Farlowellini.

Subfamily Loricariinae Bonaparte, 1831

    Tribe Harttiini Boeseman, 1971
        *Harttiella* Boeseman, 1953
        *Cteniloricaria* Isbrücker and Nijssen, 1979
        *Harttia* Steindachner, 1877
    Tribe Farlowellini Fowler, 1958
        *Lamontichthys* Miranda Ribeiro, 1939
        *Pterosturisoma* Isbrücker and Nijssen, 1978a
        *Sturisoma* Swainson 1838
        *Sturisomatichthys* Isbrücker and Nijssen, 1979
        *Farlowella* Eigenmann and Eigenmann, 1889
    Tribe Loricariini Bonaparte, 1831
        *Metaloricaria* group
            *Metaloricaria* Isbrücker, 1975
        *Dasyloricaria* group
            *Dasyloricaria* Isbrücker and Nijssen, 1979
            *Fonchiiloricaria* Rodriguez, Ortega and Covain, 2011
        *Rineloricaria* group
            *Rineloricaria*, Bleeker, 1862
        *Loricariichthys* group
            *Furcodontichthys* Rapp Py-Daniel, 1981 –*Sedis mutabilis*
            *Loricariichthys* Bleeker, 1862
            *Hemiodontichthys* Bleeker, 1862
            *Limatulichthys* Isbrücker and Nijssen, 1979
            *Pseudoloricaria* Bleeker, 1862
        *Spatuloricaria* group
            *Spatuloricaria* Schultz, 1944
        *Pseudohemiodon* group
            *Dentectus* Martín Salazar, Isbrücker and Nijssen, 1982 –*Sedis mutabilis*
            *Reganella* Eigenmann, 1905 –*Sedis mutabilis*
            *Crossoloricaria* Isbrücker, 1979
            *Planiloricaria* Isbrücker, 1971
            *Pseudohemiodon* Bleeker, 1862
            *Rhadinoloricaria* Isbrücker and Nijssen, 1974
        *Loricaria* Group
            *Pyxiloricaria* Isbrücker and Nijssen, 1984 –*Sedis mutabilis*
            *Ricola* Isbrücker and Nijssen, 1978 –*Sedis mutabilis*
            *Loricaria* Linnaeus, 1758
            *Paraloricaria* Isbrücker, 1979
            *Proloricaria* Isbrücker, 2001
            *Brochiloricaria* Isbrücker and Nijssen, 1979

The Maximum Parsimony analysis found two most parsimonious trees with a length of 14,704 steps, consistency index (CI) of 0.29 and retention index (RI) of 0.61, which were summarized as a strict consensus tree (S1 Fig). Numbers at branches are Bremer Support (BS) values. The subfamily Loricariinae is corroborated as monophyletic and has the three major clades found by the Bayesian analysis, except that *Metaloricaria* was recovered as sister to all remaining Loricariinae. Composition and genus-level relationships within Harttiini and Farlowellini are the same as in the Bayesian tree, except that *Harttiella* appears as sister to the Loricariini.

## Discussion

### Morphological characters

Broad phylogenetic analyses of the Loricariinae are scarce in the literature. There are four studies dealing with the whole subfamily: two morphology-based, which are not published [28, 39], and two molecular-based [15, 18]. There have been studies dealing with phylogenetic analyses of the genera of Loricariinae (e.g. *Sturisoma* by Ghazzi [36]; *Rineloricaria* by Fichberg [37]; *Lamontichthys* by Paixão and Toledo-Piza [38]; *Dasyloricaria* by Londoño-Burbano and Reis [76]; *Farlowella* by Retzer and Page [81]; *Fonchiiloricaria* by Rodriguez et al. [82]; *Loricaria* by Thomas [83]; *Loricariichthys* by Paixão [84]). Nevertheless, studies regarding the taxonomy of the Loricariinae have a long history [19, 21– 25, 62, 64, 66, 85–90] (see also Introduction).

According to Rapp Py-Daniel [28], Harttiini has the following synapomorphies: preopercle with a ventral process (CI = 1.00); second basibranchial vestigial (CI = 0.5); basipterygia cartilage plug short (CI = 0.5); lips with plates (CI = 0.5); more than 50 jaw teeth (CI = 0.43); autopalatine anterior process present (CI = 0.33); upper pharyngeal plate with bony shelf (CI = 0.33); parapophyses of fourth vertebra [complex centrum] abutting posteriorly to transcapular ligament (CI = 0.33); and connecting bone not contacting dorsal pterygiophores (CI = 0.33). All characters described by Rapp Py-Daniel [28] were included here, but resulted either as not synapomorphic, with reversals among Loricariinae, or not informative (see S1 File for list of synapomorphies).

The Farlowellini comprises taxa sharing synapomorphies related to snout and body elongation, as well as body plating (Fig 20), which were corroborated here. Isbrücker et al. [91] in their original description of *Aposturisoma*, proposed that their monotypic genus resembled both *Farlowella* and *Sturisoma*, and that this taxon could possibly represent an intermediate state between those genera. In the present study, *Aposturisoma* was found deeply nested among species of *Farlowella* (Fig 21). On the other hand, *Farlowella* was found as sister to *Sturisomatichthys*, not *Sturisoma* as proposed by Rapp Py-Daniel [28].

Two non-exclusive synapomorphies were found to support the clade including the (Harttiini, Farlowellini) by Provenzano [39]: Limited longitudinal development of lateral laminae of orbitosphenoid (15:1) and hyomandibular rectangular, elongated dorsoventrally, with anterior and posterior borders straight, parallel and vertical (49:2). Two exclusive synapomorphies supported the clade (*Sturisoma* (*Sturisomatichthys* (*Farlowella*))) as proposed by Provenzano [39]: Anterior border of mesethmoid straight or slightly rounded (1:2), and encapsulation of the nares complete, anterior canal narrow, almost as a tunnel (7:3). It was found that the lateral expansion of the anterior margin of the mesethmoid (character 1, see Character Description and Fig 1), is useful to diagnose the Farlowellini. In addition to the absence of the lateral expansion among its members [39], a slightly rounded anterior border can be used as well. On the other hand, an encapsulation, as named by Provenzano [39], was not tested here.

Covain et al. [18] carried out a molecular phylogenetic analysis of the Loricariinae, but used some external morphological features to characterize groups within Loricariinae. The authors discussed the usefulness of counts of branched caudal-fin rays and number of premaxillary and dentary teeth. Those characters are indeed useful to diagnose the clades described here, as Covain et al. [18] indicated: “…from 14 [branched caudal rays] (13 in *Farlowella*) in Sturisomina [(= Farlowellini)], to 12 in Loricariina (13 in *Metaloricaria*)”. In addition “…from Harttiini (bearing 80 premaxillary and 70 dentary teeth) to Loricariini (less than 60 premaxillary and 50 dentary teeth), and then between Sturisomina [(= Farlowellini)] (20 to 60 premaxillary and 15 to 50 dentary teeth) and Loricariina (0 to 15 premaxillary and 3 to 15 dentary teeth)”. Counts of branched caudal-fin rays reported here agree with those given by the authors. Nevertheless, counts of premaxillary and dentary teeth slightly differ from those proposed by the authors. Taxa with higher teeth counts are included in either Harttiini or Farlowellini.

### Molecular characters

Covain et al. [18] did a phylogenetic analysis using the partial 12S and 16S mitochondrial genes from 20 Loricariinae species representing 14 genera. The authors proposed the division of Loricariinae into two tribes, the Harttiini including only *Harttia*, and the Loricariini, divided in two subtribes, Loricariina and Sturisomina (the latter corrected by Covain et al. [92] as Farlowellina). Covain et al. [18] assumed the Farlowellina containing *Farlowella*, *Lamontichthys*, *Sturisoma* and *Sturisomatichthys*, as part of the Loricariini, which was later supported by Rodriguez et al. [82]. As shown in Fig 21 and S1 Fig, the taxa included by the authors as part of Farlowellina were corroborated here.

More recently, Covain et al. [15] published a molecular phylogeny of the Loricariinae using the same mitochondrial genes and the nuclear marker f-rtn4. Their results support the division of the Loricariinae into two tribes, Harttiini and Loricariini. Harttiini included *Cteniloricaria*, *Harttia*, and *Harttiella*, while Farlowellina was included as a subtribe of Loricariini, with *Aposturisoma*, *Farlowella*, *Lamontichthys*, *Pterosturisoma*, *Sturisoma*, and *Sturisomatichthys*.

Results of the present study agree with Covain et al. [15] regarding the division of Loricariinae, including the genus composition of both the Harttiini and Farlowellini. The Farlowellini was found to be supported by several non-exclusive synapomorphies. One of the characters found to diagnose the Farlowellini within Loricariinae and to differentiate it from the Harttiini (except *Cteniloricaria*), is the possession of gular plates reaching the posterior border of the lower lip (character 179:1 in this study), *versus* absence of gular plates (179:0), also observed and used by Covain et al. [15]. The authors stated, “… [In Harttiini] the abdominal cover made of small rhombic platelets can be present or absent, and when present, the abdominal cover never extends to the lower lip margin. The latter condition is, on the contrary, always observed in Farlowellina” [15], which was corroborated here.

On the other hand, Covain et al. [15] found *Aposturisoma* nested within *Farlowella*, and discussed that maintaining *Aposturisoma* would make *Farlowella* paraphyletic, so *Aposturisoma* should be placed as junior synonym of *Farlowella* to avoid this issue. Nevertheless, the authors indicated that such question needed further investigation, and thus did not formally propose that synonymy. Externally, *Aposturisoma myriodon* shows a short, broad snout, and a somewhat stockier body, which, as claimed by Covain et al. [15], could be indicative of rheophilic habits that also could be the case for *Farlowella curtirostra*. As shown on Fig 21, *A*. *myriodon* appeared, in fact, nested within *Farlowella*, and it is formally recognized here as a member of the latter.

Finally, Covain et al. [15] highlighted the fact that *Sturisoma* (including trans-Andean species) is paraphyletic; as it can be seen in Fig 21 and S1 Fig such hypothesis was corroborated. *Sturisoma rostratum*, type species of the genus, collected in rivers of Brazil (description of its type locality by Spix and Agassiz [93]) was here found grouped with the remaining cis-Andean species of the genus. In addition, another clade including *Sturisomatichthys leightoni*, type species of the genus, collected in the upper Magdalena River, in Honda, Colombia [85], grouped with *Sturisomatichthys* species, and trans-Andean species of *Sturisoma sensu lato*. As proposed by Covain et al. [15], *Sturisoma* is here assumed to include only cis-Andean species (see monophyly of the genus below) and *Sturisomatichthys* to include trans-Andean species of both genera, plus the cis-Andean *Sturisomatichthys caquetae* (see comments on monophyly of the genus below).

### Tree comparisons

The comparison of BI and MP analyses (Fig 21, S1 Fig) revealed few important differences. The composition of each tribe and genus, as well as the genus-level relationships, are the same across both trees, except for the position of *Metaloricaria* and *Harttiella*. In the BI tree, *Metaloricaria* is sister to the remaining Loricariini, while in the MP tree it was recoved as sister to all other members of Loricariinae. *Harttiella*, usually placed in the Harttiini, was recovered in that tribe by the BI analysis, but as sister to the Loricariini in the MP tree. The Maximum Likelihood analysis found the same relationships as the BI, for the subfamilies, tribes, genera, and species, except for a few species-level relationships within *Sturisomatichthys* and *Farlowella* (see S2 Fig).

Within genera of Harttiini (PP = 1) all species-level relationships are the same in both analyses, except that *Harttia punctata* is sister to *H*. *duriventris* + *H*. *trombetensis* in the BI tree and sister to *H*. *rhombocephala* in the MP tree. Among genera of Loricariini (PP = 1) species-level relationships are also the same, except that *Loricaria lundbergi* is sister to *Pseudohemiodon lamina* in the BI tree and sister to the remining Loricariini in the MP tree.

Further differences between the BI and MP trees within the Farlowellini (PP = 1, BS = 13) are restricted to species position within genera. In *Lamontichthys* (PP = 1, BS = 6), *L*. *stibaros* is sister to *L*. *llanero* in the BI tree, and sister to *L*. *filamentosus* in the MP tree. *Sturisoma* appeared as a well-supported group (PP = 1, BS = 20) with fully resolved interspecific relationships in the BI analysis, but with *S*. *graffini*, *S*. aff. *tenuirostre*, and the remaining species in a polytomy in the MP tree. Also, *S*. *barbatum* is sister to *S*. *rostratum* in the BI tree, but sister to *S*. *lyra* in the MP tree. As only four of the nine species included had DNA sequences (*S*. *monopelte*, *S*. *nigrirostrum*, *S*. *robustum*, and *S*. aff. *tenuirostre*), the difference in topology could be due to the amount of missing data. The inclusion of additional molecular data for *Sturisoma* is needed, since most species, including the type species (*S*. *rostratum*), lack that type of evidence. *Sturisoma* remains as a poorly studied genus regarding its taxonomy, and additional characters for the proper diagnosis of this group are necessary. The present definition of the genus is a first step towards an understanding of this group, and the first author is currently working on a taxonomic revision of the genus.

*Sturisomatichthys* is a well-supported clade (PP = 1, BS = 10) encompassing *S*. *tamanae* and *S*. *citurensis* as sister to the remaining species in both the BI and MP trees. Within this latter clade (*S*. *festivus*, *S*. *kneri*) are sister to the remaining species in the BI tree, while they are sister to (*S*. *reinae*, *S*. *varii*) in the MP tree.

Finally, the larger amount of differences between the BI and the MP analyses occurred in *Farlowella* (PP = 1, BS = 9), which is formed by two clades. The first one has a medium and low support (PP = 0.95, BS = 1) and shows the same relationships in both analyses except for the addition of *F*. *venezuelensis* in the MP tree. The second clade, despite having medium support in the BI tree (PP = 0.92), has low Bremer support (BS = 1) in the MP tree, and shows various species-level differences compared to the BI tree. In both trees, however, *Aposturisoma myriodon* appears in the same clade, either as sister to all species in the BI tree, or in a clade with (*F*. *henriquei*, *F*. *nattareri*), which in turn is sister to the remaining species, in the MP tree.

With the present study, a morphological component was incorporated in the analyses, which was lacking in recent phylogenetic studies of the Loricariinae [10, 15, 18]. Despite morphology had little influence on the BI analysis, as demonstrated by the DNA-only tree (S3 Fig) being highly congruent with the total evidence hypothesis, it was useful to resolve some unexpected relationships revealed by the DNA-only tree and was instrumental to generate phenotypic synapomorphies to diagnose genera and family-group clades (see list of synapomorphies in S1 File). The DNA-only tree has one important difference with the Total Evidence tree, *Metaloricaria* was recovered as sister to the Loricariini plus Farlowellini, instead of sister to remaining Loricariini. Other smaller differences include a few species in unexpected places and some species-level shifts inside genera. These include *Sturisomatichthys tamanae* clustering with *Sturisoma* aff. *tenuirostre*, deeply nested within the latter genus, and a few differences within *Farlowella* due to a polytomy in the BI tree. Other unexplained incongruency of the DNA-only tree is *Pseudohemiodon lamina* in Farlowellini, deeply nested among the species of *Farlowella*. These differences compared to the TE tree are unexpected and bear no support in literature or in morphology.

A MP analysis of the morphology-only partition yelded 4463 trees (L = 2016, CI = 0.16, RI = 0.70) with deeper nodes of the strict consensus only partially congruent with the BI tree. Despite the monophyly and composition of all genera were preserved, the Loricariini was recovered as sister to Harttiini plus Farlowellini. Differences of the morphology-only MP tree to the TE BI hypothesis include *Pterosturisoma* and *Lamontichthys* in a polytomy with Harttiini and remaining Farlowellini, *Aposturisoma* in a polytomy with *Sturisoma*, *Sturisomatichthys*, and *Farlowella*, and multiple intrageneric polytomies.

### Monophyly of Loricariinae

Loricariinae was recovered as monophyletic under a total evidence approach, using both Bayesian inference (PP = 1; Fig 21), and maximum parsimony analyses (BS = 52; S1 Fig). The subfamily was found to contain three main clades, recognized as the tribes Harttiini, Farlowellini, and Loricariini.

### Identification key for tribes of Loricariinae

1a. Dentary and premaxillary teeth few, typically less than 20 in each ramus; postorbital notch present (except in *Metaloricaria*); mesethmoid ventral disk reduced to a lamina………………………………………………………………………………Loricariini

1b. Dentary and premaxillary teeth many, typically more than 40 in each ramus; postorbital notch absent; mesethmoid ventral disk circular…………………….……………….………2

2a. Gular plates present on ventral surface of head between pectoral girdle and lower lip; nuchal plate articulated to surrounding plates, forming a distinctly solid shield……………………..………………………….……………………………Farlowellini

2b. Gular plates absent on ventral surface of head between pectoral girdle and lower lip; nuchal plate, when present, not articulated to surrounding plates……•.…………. Harttiini

### Monophyly of Harttiini

The Harttiini (Fig 21) resolved as (*Harttiella* (*Cteniloricaria*, *Harttia*)), differs from the Harttiini of Covain et al. [15], where *Harttia* and *Harttiella* where sister to each other and *Cteniloricaria* sister to them.

Rapp Py-Daniel [28] used Maximum Parsimony in her analyses, but did not find the compositions of the Harttiini proposed in this study (Fig 21, S1 File), or by Covain et al. [15]. The author proposed for the Harttiini to be classified as (((*Sturisomatichthys* (*Farlowella*, *Aposturisoma*) *Sturisoma*)) (*Lamontichthys* (*Harttia*)). It is worth noting that in her study neither *Harttiella* nor *Cteniloricaria* were included. On the other hand, Provenzano [39] found Harttiini to include only *Harttia*; the author did not include any *Harttiella* species and assumed *Cteniloricaria* as synonym of *Harttia*.

### Monophyly of *Cteniloricaria*

Results show *Cteniloricaria* to be monophyletic (Fig 21, S1 Fig), and formed by two valid species, *C*. *platystoma* (type species) and *C*. *napova*. On both morphological phylogenetic studies dealing with Loricariinae [28, 39], *Cteniloricaria* was assumed as a synonym of *Harttia*. Provenzano [39] included the type species of *Cteniloricaria* (as *Harttia platystoma*) in his analysis, and recovered it as sister to the species of *Harttia* included in that analysis (*Harttia merevari*, *H*. *surinamensis*). Rapp Py-Daniel [28] did not include *Cteniloricaria* in her analysis. The genus was described by Isbrücker and Nijssen [65], and since then, treated as synonym of *Harttia* [28, 39, 62, 86, 94–96], or as valid [1, 10, 15, 17, 25, 65, 66, 87, 90, 97, 98, this study].

Covain et al. [87] described the second species, *Cteniloricaria napova*, within the genus. The new species was diagnosed from *C*. *platystoma* by its distinctly spotted color pattern, more numerous premaxillary teeth, and body measurements [87]. Paratypes of the species deposited at MHNG and one additional non-type specimen (MPEG 34190) from the Curuá River basin were examined and corroborated those characters when compared to *C*. *platystoma*. Enough material of *C*. *napova* to clear and stain to include osteological characters for the phylogenetic analysis was not available. Nevertheless, external morphology characters, and molecular data were included, and revealed the species to be valid (Fig 21, S1 Fig), and to belong to *Cteniloricaria*.

Externally, *Cteniloricaria* species can be distinguished from *Harttia* and *Harttiella* by the less numerous and larger abdominal plates, reaching the cleithral region (*vs*. when present, usually not reaching cleithral region, or scarce and small); body and head mottled or spotted (*vs*. transversal dorsal bars); and possession of a dorsal-fin spinelet (*vs*. spinelet absent).

### Monophyly of *Harttia*

*Harttia* (Fig 21, S1 File) is herein recovered as monophyletic, corroborating Covain et al. [15], Rapp Py-Daniel [28], and Provenzano [39]. Rapp Py-Daniel [28] found seven synapomorphies to support the monophyly of the genus (her characters 54, 72, 92, 150, 152, 166, 174). All characters were tested here and only one, point of bifurcation of the infraorbital and supraorbital canals on the sphenotic (our character 37) was found to be a non-exclusive synapomorphy of *Harttia*. Within *Harttia*, relationships were fully resolved, mostly highly supported. The clade (*H*. *guianensis* (*H*. *tuna* (*H*. *fluminensis*, *H*. *surinamensis*))) (Fig 21, S1 Fig) shows strong biogeographical congruence because the four species are distributed along the Guiana Shield.

### Monophyly of *Harttiella*

*Harttiella* was found to be monophyletic (Fig 21, S1 File). The genus originally had two species *Harttiella crassicauda* (type species), and *H*. *montebelloi* (= *Rineloricaria steinbachi*) as proposed by Boeseman [23]. Later, Isbrücker [65] transferred *H*. *montebelloi* to *Ixinandria* (= *Rineloricaria*), and since then it was treated as a monotypic genus. It was not until the study of Covain et al. [87] that additional species were included in the genus. The authors described six new species: *Harttiella intermedia*, *H*. *janmoli*, *H*. *longicauda*, *H*. *lucifer*, *H*. *parva*, and *H*. *pilosa*. To date, the genus has seven valid species, five of which were included in this study. Covain et al. [87] diagnosed *Harttiella* mainly by body measurements and by having the abdomen naked with exception of lateral abdominal plates and, rarely, preanal plates; small size (largest known specimen reached 52 mm SL); body densely covered by odontodes; and canal plate (their subpreopercle) not exposed. Covain et al. [87] also suggested that there are two groups within *Harttiella*, the *H*. *crassicauda* group, including the stockier forms, and the *H*. *longicauda* group, including slender species.

Externally, species of *Harttiella* can be distinguished from *Harttia* by having a broad caudal peduncle that homogeneously narrow towards the caudal fin (vs. caudal peduncle subtly becoming narrower at midlength). From *Cteniloricaria* it can be distinguished by the absence of central abdominal plates (*vs*. central abdominal plates present). Furthermore, *Harttiella* has a canal-bearing cheek plate with a long and thin ventral process (*vs*. short, broad process in *Cteniloricaria* and *Harttia*), and fully mature adults of *Harttiella* reach to a maximum of 52 mm SL (*vs*. adults with more than 100 mm SL, except *Harttia absaberi*).

### Identification key for genera of Harttiini

1a. Plates on caudal peduncle smooth, without keels; odontodes usually not well developed on sides of head or predorsal region; canal-bearing cheek plate with short, broad ventral process; caudal peduncle strongly depressed……………………..….………………………2

1b. Plates on caudal peduncle keeled, not smooth; odontodes well developed on sides of head or predorsal region; canal-bearing cheek plate with long and thin ventral process; caudal peduncle oval in cross-section………………………….…………………..*Harttiella*

2a. Dark blotch at caudal-fin base; tip of snout naked, devoid of plates; abdominal plates absent or present as small platelets, partially or completely covering the abdomen………………………………………………………………………………*Harttia*

2b. Dark transverse, half-moon shaped band at caudal-fin base, occupying base of all rays of upper and lower lobes; tip of snout covered with plates; abdominal plates, as medium-sized polygonal plates completely covering the abdomen.…………………….*Ctenilorcaria*

### Monophyly of Farlowellini

Farlowellini is recovered as monophyletic (Fig 21, S1 File), and encompassing *Farlowella*, *Lamontichthys*, *Pterosturisoma*, *Sturisoma* and *Sturisomatichthys*. Rapp Py-Daniel [28] divided the Harttiini into Farlowellina (*Farlowella*, *Aposturisoma*, and *Sturisoma*) and Harttiina (*Harttia* and *Lamontichthys*). The author diagnosed Farlowellina by possessing 7–40 mandibular teeth (183.1), quadrate elongate (48.0), abdominal plates large and quadrangular (190.3), fourth epibranchial bone twisted, without flange (78.2), cleithral anterolateral process absent (141.0), and abdominal plates organized in series (191.1). All these characters were included here (see Characters Description), but none were found to be synapomorphic of the Farlowellini.

### Monophyly of *Farlowella*

More than 20 years have passed since Retzer and Page [81] published the only taxonomic revision of *Farlowella* to date. The authors performed a MP search of 30 morphological, unordered characters for 25 species. Nineteen species were placed in six species groups recognized as monophyletic, and six species were considered as *incertae sedis*. *Aposturisoma myriodon* was used to root the tree so its relationships to *Farlowella* were not actually tested. Retzer and Page [81] suggested the following groups (see [81] for composition of each group): The *F*. *curtirostra*, *F*. *nattereri*, *F*. *acus*, *F*. *amazonum*, and *F*. *knerii* species group. *Farlowella gracilis*, *F*. *hahni*, *F*. *oxyrryncha*, *F*. *paraguayensis*, *F*. *reticulata*, and *F*. *smithi* were regarded as *incertae sedis*. These groups were proposed based mainly on the presence and number of vertical rows of lateral plates (counted from predorsal to lateral abdominal plates), number of rows of abdominal plates, degree of development of odontodes on the snout, and length and breadth of the snout. Subsequent to this study, descriptions of new species [99–103], followed the groups and characters outlined by Retzer and Page [81]. Nevertheless, five of those six species groups were not recovered as monophyletic (except for the *Farlowella acus* group) and the characters proposed to diagnose them are not phylogenetically informative.

According to Rapp Py-Daniel [28], *Farlowella* is part of her Farlowellina and *F*. *amazonum* and *Farlowella* sp. (aquarium specimens) were included in her analysis. Twelve characters found as synapomorphic for *Farlowella* were tested here, but differences regarding her results were found. One character (her character 124), was indeed found as synapomorphic for the genus, the low number of caudal-fin rays (character 191), also shared by *Aposturisoma myriodon* and *Sturisomatichthys guaitipan* [90].

According to Covain et al. [15] *Farlowella* is divided into two groups: “*Farlowella* 1”, including *F*. *acus*, *F*. *hahni*, *F*. *knerii*, *F*. *mariaelenae*, *F*. *martini*, *F*. *nattereri*, *F*. *oxyrryncha*, *F*. *paraguayensis*, *F*. *smithi*, and *F*. *vittata*, as sister to *Aposturisoma myriodon*. We found the same group, except *F*. *oxyrryncha*, and with the addition of *F*. *amazonum*, *F*. *hasemani*, *F*. *henriquei*, *F*. *jauruensis*, *F*. *schreitmuelleri*, *F*. *venezuelensis*, as sister to *Aposturisoma*. The “*Farlowella* 2” group, encompasing the stockier forms (*F*. *amazonum*, *F*. *curtirostra*, *F*. *platorhynchus*, *F*. *rugosa*, and *F*. *taphorni*) was also recovered, with the addition of *F*. *yarigui*. *Farlowella oxyrryncha*, *F*. *reticulata*, and *F*. *isbruckeri* were found in a polytomy, sister to the first group.

*Farlowella* remains a problematic group regarding taxonomy and diagnosis of its species, due to its very conservative morphology and low variation between species. As remarked by Covain et al. [15], “The taxonomy of *Farlowella* is also confused and the group needs further revision”. Even though morphological characters were included, most of the synapomorphies found to support the several groups proposed were osteological, and characters of external morphology showed to be homoplastic and not useful for a diagnosis at the species level. Characters of easy identification for a stable taxonomy of the group were not identified.

### Monophyly of *Lamontichthys*

*Lamontichthys* was described by Miranda Ribeiro [104] based on *Harttia filamentosa* from the Juruá River, a tributary to the Solimões River, Brazil; the author based his description on the presence of seven branched pectoral-fin rays. Here, the genus was found to be monophyletic and sister to all remaining Farlowellini (Fig 21): (*L*. *avacanoeiro*, *L*. *parakana*), (*L*. *filamentosus* (*L*. *llanero*, *L*. *stibaros*)). Paixão and Toledo-Piza [38] found the relationships (*L*. *avacanoeiro*, *L*. *stibaros*), (*L*. *maracaibero* (*L*. *filamentosus*, *L*. *llanero*)); they did not include *L*. *parakana* due to the lack of specimens for osteological observations. On the other hand, *L*. *maracaibero* was not included in the present study due to the lack of both specimens and tissues for DNA extraction.

Rapp Py-Daniel [28] included only *L*. *filamentosus* in her phylogenetic analysis, and placed the genus sister to *Harttia* and belonging to her Harttiina. The author considered the genus diagnosed by: eight pectoral-fin rays (139.1), large and spike-like lateropterygium (157.1), fourth pharyngobranchial nodular and cartilaginous (81.2), lower pharyngeal plate triangular (82.1), and second preural spine short (134.2). All five characters were included here (see Character Description). However, only the possession of seven branched pectoral-fin rays was identified as a synapomorphy for *Lamontichthys*.

Paixão and Toledo-Piza [38] found *Lamontichthys* as monophyletic and included six valid species (see [38] for a synapomorphy list). Here, nine of the ten characters reported by them (characters 47, 95, 98, 127, 128, 137, 159, 164, and 173, see Character Description) were included. One of them, seven branched pectoral-fin rays (our character 137.1), was corroborated as synapomorphic for *Lamontichthys*.

### Genus *Pterosturisoma*

Isbrücker and Nijssen [89] described *Pterosturisoma* with *Harttia microps* (= *Pterosturisoma microps*) as its type species. The authors highlighted the resemblance of *Pterosturisoma* to *Lamontichthys*, from which it differs by the number of branched pectoral-fin rays (six *vs*. seven), size of the orbit (smaller in *Pterosturisoma*), number of lateral plates with lateral-line pores (more numerous in *Pterosturisoma*), and fewer central abdominal plates in *Pterosturisoma*.

*Pterosturisoma* was recovered as monotypic, belonging to the Farlowellini, and sister to (*Sturisoma* (*Sturisomatichthys*, *Farlowella*))). Rapp Py-Daniel [28] did not include *Pterosturisoma* in her analysis. However, she did examine specimens in alcohol and c&s. Based on those specimens she wrote “I agree with its current placement in the Harttiini *sensu* Isbrücker, based on the presence of a well-developed [palatal] splint, lateropterygium, and large premaxillae and dentaries. [*Pterosturisoma* can be distinguished from *Lamontichthys*] by having fewer pectoral-fin rays, and a longer, deeper head” [28]. The author assigned *Pterosturisoma* to her Harttiina based on the possession of the connecting bone contacting the lateral processes of the second dorsal-fin pterygiophore, lack of derived features related to snout elongation, and abdominal plates organized in series. Only the latter was here corrobotated as diagnostic of the Farlowellini (Harttiina [28]).

Paixão and Toledo-Piza [38] found two autapomorphies that supported *Pterosturisoma*, the lack of a crest on the lateral surface of hyomandibula for the insertion of the *levator arcus palatine* muscle (31.0>1), and the elongate and narrow distal portion of the hemal spine of the last precaudal vertebra (40.1>2) [36]. The former was here corroborated as autapomorphic (despite not exclusive) for *Pterosturisoma* (character 71.1). Finally, Covain et al. [15] also found *Pterosturisoma* as sister to a clade comprising (*Sturisoma* (*Sturisomatichthys*, *Farlowella*)) (their Fig 3), and included it in the Farlowellina.

### Monophyly of *Sturisoma*

*Sturisoma* belongs to the Farlowellini, and appears as sister to the clade (*Sturisomatichthys*, *Farlowella*). Interspecific relationships of the genus were found as (*S*. *monopelte* (*S*. *robustum* (*Sturisoma graffini*, *S*. aff. *tenuirostre*))) (*S*. *nigrirostrum* (*S*. *guentheri* (*S*. *lyra* (*S*. *barbatum*, *S*. *rostratum*)))) (Fig 21).

Rapp Py-Daniel [28] included *Sturisoma* sp. (from the Jepurá and Apuré rivers) in her analysis, and found the genus to be sister to the clade (*Farlowella*, *Aposturisoma*) as part of her Farlowellina. The author diagnosed the genus by “highly homoplasious synapomorphies”, which included transverse process of fourth vertebra [complex centrum] reaching beyond compound pterotic border (94.2), parapophysis of fourth vertebra [complex centrum] abutting ventrally to transcapular ligament (92.2), hyomandibula concavity large without foramen (41.1), fourth pharyngobranchial nodular and cartilaginous (81.2), and lower pharyngeal plate triangular (82.1). All four characters were included here but none was found to be synapomorphic of the genus.

Provenzano [39] included only *S*. *tenuirostre* and found it to be sister to his (*Sturisomatichthys* (*Aposturisoma*, *Farlowella*)) clade. According to the author, *S*. *tenuirostre* is diagnosed by two autapomorphies: lateral lamina of orbitosphenoid reaching more than half of its length (15.0), and small cavity at base of lateral lamina of orbitosphenoid (16.2). Neither of the characters were included here.

Ghazzi [36] carried out a morphology-based phylogeny including eight valid species of *Sturisoma sensu lato*, plus five undescribed species, and found a monophyletic *Sturisoma* including only cis-Andean species, plus *S*. *kneri* (= *Sturisomatichthys kneri*), which was found here not to belong to *Sturisoma*, a conclusion in agreement with those of Covain et al. [15], and Londoño-Burbano and Reis [90]. Ghazzi [36] used 20 characters to support that clade [36]; 12 of which were analyzed here (see [36] for the list of Synapomorphies, and Character Description). Of these, only two were corroborated as synapomorphic for the genus: upper pharyngeal tooth plates triangular (character 107.3; Fig 14B) as exclusive for the genus and rictal barbel half of orbit diameter (character 172.1) as non-exclusive.

### Monophyly of *Sturisomatichthys*

Rapp Py-Daniel [28] included *Sturisomatichthys citurensis* in her phylogenetic analysis of the Loricariinae. The author found the genus to be part of her Harttiini, as sister to the remaining genera in that tribe. The author found only one synapomorphy to support the genus, the presence of a parietal branch terminal exit on the border between frontal and sphenotic (174.1). That character was tested here (38.2), but was not found to be synapomorphic for the genus. In addition, in members of *Sturisomatichthys* the terminal exit of the parietal branch ends on the sphenotic (38.4), not at the border between frontal and sphenotic, as stated by Rapp Py-Daniel [28].

Ghazzi [36] included three species of *Sturisomatichthys* in her phylogenetic analysis, *S*. *leightoni*, *S*. *citurensis*, and *S*. *tamanae*. The author found *S*. *citurensis* and *S*. *tamanae* to be part of a clade separate from that including *S*. *leightoni*, while three undescribed species revealed by the author, along with *S*. *panamensis* and *S*. *festivus*, did appear in the clade containing the type species of *Sturisomatichthys*. The author assumed *S*. *dariensis* as a junior synonym of *S*. *panamensis*, and *S*. *aureus* was regarded as *incertae sedis*. The former was found to be valid (see below), and *S*. *aureus* recovered as sister to *S*. *leightoni* (Fig 21).

Regarding molecular analyses of the Loricariinae, both Covain et al. [18] and Rodriguez et al. [82] included *Sturisomatichthys citurensis*. Both studies found the species as sister to *Farlowella*, a topology congruent with our hypothesis. The present study is the first phylogenetic analysis to include 11 of the 13 valid species of the genus and to recover a monophyletic *Sturisomatichthys* (Fig 21).

The separation of *Sturisomatichthys* and *Sturisoma* as respectively trans- and cis-Andean species of Covain et al. [15] was corroborated here, with the exception of *Sturisomatichthys caquetae*, which is cis-Andean. Trans-Andean species of *Sturisoma sensu lato* were found within the clade comprising *Sturisomatichthys sensu stricto*, which includes its type species (*S*. *leightoni*). As proposed by Covain et al. [15] and corroborated here, both the trans-Andean species of *Sturisoma sensu lato*, and the trans-Andean species included in *Sturisomatichthys* in its original description comprise *Sturisomatichthys*. Thus, the genus was found to comprise *S*. *aureus*, *S*. *caquetae* (not included here), *S*. *citurensis*, *S*. *dariensis*, *S*. *festivus*, *S*. *frenatus*, *S*. *guaitipan* (not included here), *S*. *kneri*, *S*. *leightoni*, *S*. *panamensis*, *S*. *reinae*, *S*. *tamanae*, and *S*. *varii*. It is worth noting that Covain et al. [15] suggested that *S*. *caquetae* should be included in *Sturisoma*, since it is a cis-Andean species. Nevertheless, the authors did not include any specimen in their analysis, and their recommendation for transferring the species to *Sturisoma* was solely based on the distribution. Even though specimens of *S*. *caquetae* were not included in this study, the holotype of *Harttia caquetae* (ANSP 71719) was examined and found to have external diagnostic characters of *Sturisomatichthys*. Thus, *Sturisoma caquetae* was transferred back to *Sturisomatichthys* by Londoño-Burbano and Reis [90]. It is worth noting that *S*. *caquetae* is the only fully cis-Andean species in the genus, but non-type material is needed to redescribe and more accurately diagnose this species (see Londoño-Burbano and Reis [90] for a complete taxonomic account of the genus).

*Sturisomatichthys* shows higher species richness than that described by Isbrücker and Nijssen [65]. With 13 valid species [90], *Sturisomatichthys* went from a species-poor genus to one of the most diverse within Farlowellini. To date only *Farlowella*, *Harttia*, *Loricaria*, *Loricariichthys*, *Rineloricaria*, and *Spatuloricaria*, have more species than *Sturisomatichthys* among the Loricariinae. On the other hand, *Sturisomatichthys* surpasses *Sturisoma*, which was considered as one of the largest genera of the subfamily, and which is herein restricted to ten strictly cis-Andean species. Even though the diversity of this group was greatly underappreciated and understudied, there is still much to describe regarding the richness of *Sturisomatichthys*, as its known range of distribution includes poorly studied localities of difficult to access areas (i.e. Pacific slope and upper Amazon in Colombia). Nevertheless, this study and those of Covain et al. [15] and Londoño-Burbano and Reis [90] are efforts to improve the knowledge on this group and its taxonomy. The fact that *Sturisomatichthys* remained obscure for more than 30 years is evidence of the need of taxonomic studies not only for the Loricariinae, but for all Neotropical fishes, and to put effort into the sampling of poorly studied areas.

### Identification key for the genera of Farlowellini

1a. Snout round; rostrum not produced; minute abdominal plates; eye small ……………2

1b. Snout triangular; rostrum generally elongated; medium to large abdominal plates; eye large …………………………………………………………………………………………..3

2a. Seven branched rays in pectoral fin.……………………………..………..*Lamontichthys*

2b. Six branched rays in pectoral fin………………………………………….*Pterosturisoma*

3a. Dorsal-fin origin on same vertical as pelvic-fin origin; laterodorsal longitudinal stripe, when present, passing over eye; lateral abdominal plates separated from central abdominal plates by skin; numerous small central abdominal plates…………………..….……………4

3b. Dorsal-fin origin between pelvic fin and anal-fin origin; laterodorsal longitudinal stripe, when present, passing below eye; lateral abdominal plates contacting central abdominal plates; none to two series of large central abdominal plates.……………………..*Farlowella*

4a. Central abdominal plates not arranged in clearly defined longitudinal series; dark spots usually present on one or more of the dorsal-, pectoral-, pelvic, or anal-fins; 15–18 lateral plates in median series………………………………………… .… .… .… .……*Sturisomatichthys*

4b. Central abdominal plates arranged in three clearly defined longitudinal series; dark spots usually absent from all fins, sometimes present on pectoral fin; 20–21 lateral plates in median series…..………………………………………………………………..…*Sturisoma*

### Monophyly of Loricariini

The focus of this study was the Harttiini and Farlowellini, and members of the Loricariini are comparatively less numerous in the analyses. Thus, we present only a brief discussion of the results regarding the tribe, based on the most recent Loricariinae study of Covain et al. [15]. Loricariini was found to be monophyletic (Fig 21, S1 File). The genera included here are *Dasyloricaria*, *Hemiodontichthys*, *Limatulichthys*, *Loricaria*, *Loricariichthys*, *Metaloricaria*, *Pseudohemiodon*, *Rineloricaria*, and *Spatuloricaria*. See S1 File for the complete composition of the tribe.

Covain et al. [15] divided the Loricariini in several groups: the *Rineloricaria* group (including only *Rineloricaria*, and divided in several subgroups), the *Loricariichthys* group (*Furcodontichthys*, *Hemiodontichthys*, *Limatulichthys*, *Loricariichthys*, and *Pseudoloricaria*), the *Loricaria*–*Pseudohemiodon* group (*Brochiloricaria*, *Crossoloricaria*, *Dentectus*, *Loricaria*, *Paraloricaria*, *Planiloricaria*, *Proloricaria*, *Pseudohemiodon*, *Pyxiloricaria*, *Reganella*, *Rhadinoloricaria*, *Ricola*, and *Spatuloricaria*), while *Dasyloricaria*, *Fonchiiloricaria*, and *Metaloricaria* were not included in any group [15]. Except for *Spatuloricaria*, which did not clustered with *Loricaria* and *Pseudohemiodon*, the relationships recovered in the present analysis (Fig 21) are fully congruent with the groups of Covain et al. [15].

### Concluding remarks

In this study a total evidence phylogenetic analysis of the subfamily Loricariinae of the suckermouth catfishes was performed including available morphological and molecular information. The analysis had an emphasis on the tribes Harttiini and Farlowellini, and was able to clarify their intergeneric and intraspecific relationships. While molecular characters were valuable to provide a robust dataset for the analysis, comparatively fewer morphological characters were instrumental to built phylogenetic diagnoses for the subfamily, its tribes, and each of the genera in Hartiini and Farlowellini.

## Material examined

### Tissue samples used for DNA extraction

**Ingroup: *Cteniloricaria napova*:** MHNG 2704.030, Brazil, Pará, Trombetas, Paru do Oeste River. ***Cteniloricaria platystoma*:** AUM 48174, tissue catalog AUM3890, Guyana, Rupununi River, Essequibo River, Region 8 (Potaro-Siparuni). ***Dasyloricaria filamentosa*:** CZUT 5104, Colombia, Tolima, Magdalena River, Quebrada Sabandija at junction with main channel. ***Dasyloricaria latiura*:** STRI 1559, Colombia, Chocó, Atrato River, Creek 3–27. ***Dasyloricaria paucisquama*:** CZUT 5105, Colombia, Magdalena River, Quebrada Sabandija. ***Farlowella acus*:** STRI MER95T-23, Venezuela, Aragua, Carabobo, Lago Valencia drainage. ***Farlowella amazonum*:** MCP 45943, Brazil, Mato Grosso do Sul, Novo Horizonte do Sul, upper Paraná basin, Guiraí River, tributary of Ivinhema River between Naviraí and Ivinhema. ***Farlowella* aff. *amazonum*:** STRI MER95T-26, upper Amazon River at border Peru-Brazil, aquarium trade. ***Farlowella curtirostra*:** STRI MER95T-15, Venezuela, Maracaibo, Yasa River, Lago Maracaibo basin. ***Farlowella hahni*:** STRI-2205, Paraguay, Paraguay River, Curuguaty Creek. ***Farlowella hasemani*:** MCP 36626, Brazil, Acre, Brasiléia, Purus basin, Entrocamento Creek on road BR-317, ca. 5 km E of Brasiléia. ***Farlowella knerii*:** MHNG 2710.052, Peru, Tocache Province, Huacamayo River basin, Aspuzana River. ***Farlowella mariaelenae*:** STRI MER95T-2, Venezuela, Orinoco River basin, San Carlos River. ***Farlowella martini*:** STRI VZ-126, Venezuela, Falcón, Atlantic drainage, Aroa River, Caripial Creek. ***Farlowella myriodon*:** MHNG 2710.035, Peru, Amazonas, Huacamayo River. ***Farlowella nattereri*:** MHNG 2650.099, Guyana, Demerara-Berbice región, Kurupukari crossing, Essequibo River basin. ***Farlowella oxyrryncha*:** MCP 44240, Peru, Ucayali, Pucallpa, Ucayali River basin, Cashibo Creek, Yarinacocha. ***Farlowella paraguayensis***: LBP 5217, Brazil, Paraná River. ***Farlowella reticulata***: AUM 48169, tissue catalog AUM3642, Guyana, Rupununi River, Essequibo River, Region 8 (Potaro-Siparuni), Burro Burro River at Suraima. ***Farlowella rugosa*:** AUM 47750, tissue catalog AUM3648, Guyana, Branco River, Negro River drainage, Region 9 (Upper Takutu-Upper Essequibo), Takutu River, Garlic landing, beach N of Lethem. ***Farlowella schreitmuelleri*:** MHNG 2601.087, Brazil, Pará, Guamá River. ***Farlowella smithi*:** ANSP 180541, Peru, Madre de Dios, Amazon River basin, Manuripi River. ***Farlowella taphorni*:** STRI VZ-89, Venezuela, Zulia, Lake Maracaibo basin, Muyapa River. ***Farlowella vittata*:** AUM 56693, tissue catalog AUM3607, Venezuela, Amazonas, Ventuari River, Orinoco River basin 105 km E of San Fernando de Atabapo, first rapids 20 minutes by boat from mouth. ***Farlowella yarigui*:** ICNMHN 17789, paratype, Colombia, Santander, El Carmen de Chucurí, Vereda El Topón, Topón River. ***Harttia carvalhoi*:** LBP 2115, Brazil, Paraitinga River, Paraíba do Sul River basin. ***Harttia dissidens*:** LBP 5859, Brazil, Tapajós River. ***Harttia duriventris*:** LBP 7505, Brazil, Tapajós River. ***Harttia fluminensis*:** MHNG 2690.013, Suriname, Coppename River. ***Harttia fowleri*:** MHNG 2643.022, French Guiana, Oyapock River. ***Harttia gracilis*:** LBP 6331, Brazil, Paraná River. ***Harttia guianensis*:** MHNG 2757.008, French Guiana, Saint-Laurent du Maroni, Wayo jump, Maroni River basin. ***Harttia kronei*:** MCP 42440, Brazil, São Paulo, Iporanga, Betari River (or tributary) at Bairro da Serra, between Iporanga and Apiaí, Ribeira de Iguape River basin. ***Harttia leiopleura*:** LBP 6847, Brazil, São Francisco River. ***Harttia longipinna*:** DZSJRP 2819, Brazil, São Francisco River. ***Harttia loricariformis*** LBP 2121, Brazil, Paraíba do Sul River. ***Harttia novalimensis*:** LBP 5836, Brazil, São Francisco River. ***Harttia punctata*:** MHNG 2645.059, Brazil, Goiás, Minaçú, Tocantins River basin, Batéia River, tributary of Tocantins River. ***Harttia surinamensis*:** MHNG 2674.042, Suriname, Atlantic drainage, Sipaliwini, Suriname River. ***Harttia torrenticola*:** LBP 5835, Brazil, São Francisco River. ***Harttia tuna*:** MHNG 2704.029, Suriname-Brazil border, Four Brothers Mountains, Sipaliwini Savannah, Trio Amerindian territory, Paru de Oeste River. ***Harttiella crassicauda*:** AUM 50387, tissue catalog AUM4198, Suriname, Sipaliwini, Paramaka Creek, Marowijne (Maroni) River. ***Harttiella intermedia*:** MHNG 2713.087, French Guiana, Sinnamary, Sinnamary River, Tabular Mountain of Trinité massif. ***Harttiella longicauda*:** MHNG 2699.070, French Guiana, Trinité Mountains, Mana River drainage, in a tributary of Crique Baboune, Crique Aya around 100m in front of Aya Camp. ***Harttiella lucifer*:** MHNG 2754.082, French Guiana, Maripasoula, Maroni River basin, Crique Nouvelle France, towards Saül, tributary of Limonade Creek. ***Harttiella pilosa*:** MHNG 2682.055, French Guiana, Tortue Mountains, Orapu River drainage in Crique Grillon at the ONF camp. ***Hemiodontichthys acipenserinus*:** MCP 28819, Brazil, Acre, Rio Branco, Purus River basin, Iquirí River, tributary of Ituxi River. ***Lamontichthys filamentosus*:** AUM 45589, tissue catalog AUM4024, Peru, Amazonas, Marañón River, pongo above Borja, 35.5 km NE of Juan Velasco (Sta. Maria de Nieva). ***Lamontichthys llanero*:** MHNG 2749.019, Colombia, aquarium trade. ***Lamontichthys stibaros*:** AUM 57480, tissue number T10365, Peru, Amazonas, Madre de Dios, Madre de Dios River, Amazon basin. ***Limatulichthys griseus*:** MCP 46112, Brazil, Roraima, Caroebe, Jauaperi River 4 km of Caroebe, Negro River basin. ***Loricaria lundbergi*:** MCP 46205, Brazil, Roraima, Jaburu Creek at road next to BR-174 between Jundiá and Rorainópolis, Negro River basin. ***Loricariichthys anus*:** MCP 28415, Brazil, Rio Grande do Sul, São Vicente do Sul, Uruguay River basin, Ibicuí-Mirim River, upstream of the river mouth of Santa Maria River. ***Loricariichthys platymetopon*:** MCP 21614, Brazil, Rio Grande do Sul, Uruguaiana, Uruguay River basin, Uruguai River and lateral puddles at Formosa beach, São Marcos. ***Metaloricaria nijsseni*:** MHNG 2756.054, French Guiana, Saramacca, Saint-Laurent-du-Maroni, Station 7, Saramacca River downstream Poesoegronoe. ***Metaloricaria paucidens*:** MHNG 2757.023, French Guiana, Saint-Laurent du Maroni, Maroni River basin, Wayo jump, Marouini River. ***Pseudohemiodon lamina*:** MCP 36579, Brazil, Mato Grosso, Pontes e Lacerda, Madeira River basin, Bugre River about 42 km N of Guaporé River on highway BR-174. ***Pterosturisoma microps*:** MHNG 2677.072, Peru, aquarium trade. ***Rineloricaria cadeae*:** MCP 21217, Brazil, Rio Grande do Sul, Agudo, Jacui River basin, creek about 21 km NNW of Agudo on road to UHE Dona Francisca. ***Rineloricaria lanceolata*:** MCP 34465, Brazil, Acre, Sena Madureira, Purus River basin, creek tributary to Antimari River on highway BR 364, 48 km SE of Sena Madureira. ***Rineloricaria quadrensis*:** MCP 21195, Brazil, Rio Grande do Sul, Cidreira, Tramandai, Lagoa Fortaleza. ***Spatuloricaria puganensis*:** AUM 46520, tissue catalog AUM4067, Peru, Amazonas, Rio Marañón, Utcubamba River 23 km SE of Bagua Chica. ***Sturisoma guentheri*:** ANSP 182587, Peru, Loreto, Iquitos, Nanay River. ***Sturisoma monopelte*:** AUM 44446, tissue catalog AUM3616, Guyana, Essequibo River, Region 9 (Upper Takutu-Upper Essequibo), Rupununi River at Kwatamang. ***Sturisoma nigrirostrum*:** ANSP 178322, Peru, Loreto, Maynas, Amazon River main channel along W bank, 30–45 min upstream from inlet to mouth of Itaya River (Iquitos). ***Sturisoma robustum*:** MHNG 2677.002, Paraguay, Paraguay River. ***Sturisoma* aff. *tenuirostre*:** MCP 34083, Brazil, Acre, Rio Branco, Purus River basin, Iquirí River, a tributary to Ituxi River, Purus River basin. ***Sturisomatichthys aureus*:** MHNG 2684.019, Colombia, aquarium trade. ***Sturisomatichthys citurensis*:** STRI 3587, Panama, Tuira River basin, Chucunaque River. ***Sturisomatichthys dariensis*:** STRI 26795, Panama, Tuira River basin, Chucunaque River. ***Sturisomatichthys festivus*:** STRI MER95T-20, Venezuela, Maracaibo Lake. ***Sturisomatichthys frenatus*:** STRI 872, Colombia, Nariño, San Juan River. ***Sturisomatichthys leightoni*:** MPUJ 7865, Colombia, Cundinamarca, Magdalena River. ***Sturisomatichthys panamensis*:** MHNG 2674.058, Panama, Chepo, Bayano River basin, Ipeti River. ***Sturisomatichthys tamanae*:** ANSP 198426, Colombia, Chocó, San Juan River.

**Outgroup: *Acestridium scutatum*:** MCP 37785, Brazil, Amazonas, Humaitá, Traíra River approx. 35 km E of Madeira River, on Trans Amazon highway. ***Ancistrus brevipinnis*:** MCP 25167, Brazil, Rio Grande do Sul, Pinheiro Machado, São Gonçalo, Arroio dos Pires, next to railroad at Passo dos Pires, Piratini River basin. ***Chaetostoma breve*:** AUM 46515, tissue catalog AUM4063, Peru, Amazonas, Marañón River, Utcubamba River, 23 km SE of Bagua. ***Hemipsilichthys gobio*:** MCP 42452, Brazil, Minas Gerais, Lima Duarte, Paraíba do Sul River basin, Pirapetinga River between Santa Bárbara do Monte Verde and road BR-267, approx. 10 km from BR-267. ***Hisonotus laevior*:** MCP 23005, Brazil, Rio Grande do Sul, Triunfo, Jacui River basin, Bom Jardim Creek. ***Neoplecostomus microps*:** MCP 42432, Brazil, São Paulo, Pindamonhangaba, Paraíba do Sul River basin, Ribeirão Grande River at Ribeirão Grande. ***Pareiorhaphis calmoni*:** MCP 41275, Brazil, Santa Catarina, Águas Mornas, Sudeste, Cubatão River near Queçabas, on road from Águas Mornas to João Bonifácio. ***Parotocinclus maculicauda*:** MCP 41911, Brazil, Santa Catarina, Camboriú, creek about 6.5 km S of road BR-486, at road to Rio do Meio. ***Pterygoplichthys lituratus*:** MCP 35757, Brazil, Mato Grosso, Pontes e Lacerda, creek tributary to Guaporé River on highway BR-174, between Pontes e Lacerda and Comodoro.

### Material examined for phenotypic characters

**Ingroup: *Cteniloricaria napova*:** MHNG 2704.030, 6 alc, paratypes, Suriname, Sipaliwini District, Savannah in Trio Amerindian territory at the Suriname-Brazil border, Four Brothers Mountains in a tributary of the Paru de Oeste River. MPEG 34190, 1 alc, Brazil, Pará State, Óbidos, Curuá River basin, Erepecuru River (also known as Cuminá, or Paru do Oeste). ***Cteniloricaria platystoma*:** AUM 48174, 8 alc, 1 c&s, Guyana, Rupununi-Essequibo River drainage, Region 8 Potaro-Siapruni, Burro Burro River, at Suraima. ***Dasyloricaria filamentosa*:** CP-UCO 1359, 6 alc, 1 c&s, Colombia, Cesar Department, El Paso District, Magdalena River basin, Cesar River. ***Dasyloricaria latiura*:** CAS 13187, 3 of 6 alc, Colombia, Chocó Department, Boca de Certegui District, Atrato River basin. USNM 293296, 1 c&s, Panama, Darien Province, Tuyra River basin, ½ km above Boca de Cupe. ***Dasyloricaria paucisquama*:** MPUJ 6019, holotype, Colombia, Caldas Department, La Dorada, La Española farm at Zona El Gigante, Magdalena River basin, Purrio River. CP-UCO 143, paratype, 1 c&s, Colombia, Antioquia Department, Magdalena River basin, southern Samaná River, tributary to La Miel River in Butantan. MCP 46920, paratype, 1 alc, same data as holotype. ***Farlowella acus*:** ANSP 130038, 55 alc, 1 c&s, Venezuela, Carabobo State, Vigirima River tributary of Guacara River, about 10 km NNW of Guacara. ***Farlowella amazonum*:** MCP 15183, 1 alc, Brazil, Pará State, Itaituba, Tapajós River basin, at Piracuna neighborhood, Itaituba. MCP 29737, 1 alc, Brazil, Amazonas State, Tefé, Solimões River basin, Lake Tefé at headwaters of Lake. MCP 45943, 2 alc, 1 c&s, Brazil, Mato Grosso do Sul State, Novo Horizonte do Sul, upper Paraná River, Guiraí River, tributary of Ivinhema River, between Naviraí and Ivinhema. ***Farlowella* aff. *amazonum*:** FMNH 111528, 1 alc, 1 c&s, Peru, Loreto Province, Amazon River basin, Yanayacu River, about 6–7 km above mouth in Amazon River. UF 33089, paratypes, 2 alc, Peru, Loreto Province, Amazon River basin, within 30 mi of Iquitos; Auigon, Manati, Itaya, and/or Neuse Rivers. ***Farlowella curtirostra*:** UF 30778, 4 alc, Venezuela, Merida State, Lake Maracaibo basin, Chama River just north of El Vigia on the road to Merida. USNM 121081, 3 alc, 1 c&s, Venezuela, Trujillo State, Lake Maracaibo basin, Motatan River system at San Pedro River. ***Farlowella hahni*:** MCP 10982, 3 alc, Argentina, Santa Fé Province, Santa Fé, lower Parana basin. MCP 16461, 1 alc, 1 c&s, Argentina, Santa Fé Province, Santa Fé, Parana River basin. ***Farlowella hasemani*:** MCP 36626, 5 alc, 1 c&s, Brazil, Acre State, Brasiléia, Purus River basin, Entroncamento Creek, about 5 km east of Brasiléia, at BR-317 highway. ***Farlowella henriquei*:** MCP 41992, 3 alc, 1 c&s, Brazil, Goiás State, Montes Claros de Goiás, Tocantins River basin, Água Limpa Creek, tributary of Claro River, tributary of Araguaia River. ***Farlowella isbruckeri*:** MCP 36601, 2 alc, 1 c&s, Brazil, Mato Grosso State, Nova Lacerda, Madeira River basin, Retiro Creek, tributary of Guaporé River at BR-174 highway. ***Farlowella jauruensis*:** MCP 36625, 2 alc, 1 c&s, Brazil, Rondônia State, Ji-Paraná, Madeira River basin, small tributary of right margin of Machado River, about 8 km South of bridge on BR-364 highway at Ji-Paraná. MCP 36588, 2 alc, Brazil, Mato Grosso State, Mirassol d`Oeste, Paraguay River basin, small river tributary of Caeté River, tributary of Jauru River at BR-174 highway, about 72 km northwest from Paraguay River. ***Farlowella knerii*:** FMNH 99143, 6 alc, 1 c&s, Ecuador, Napo Province, Amazon River basin, Capihuara Creek, tributary of Payamino River. ***Farlowella mariaelenae*:** USNM 349392, 11 alc, 1 c&s, Venezuela, Portuguesa State, Guanare, Orinoco River basin, Portuguesa River just upstream highway 5, 11 km NW Guanare. ***Farlowella myriodon*:** MHNG 2710.035, 2 of 17 alc, 1 c&s, Peru, Ucayali Department, Ucayali River basin, Huacamayo River. ***Farlowella nattereri*:** AUM 27707, 2 alc, Venezuela, Portuguesa State, Apure-Orinoco River system, Portuguesa River just upstream hwy 5, 11 km WNW of Guanare. MCP 29715, 1 alc, 1 c&s, Brazil, Amazonas State, Alvarães, Solimões River basin at Içé island. ***Farlowella oxyrryncha*:** MCP 44240, 6 alc, 1 c&s, Peru, Ucayali Province, Pucallpa District, Ucayali River basin, Cashibo channel, Yarinacocha. ***Farlowella paraguayensis*:** FMNH 108585, 11 alc, 1 c&s, Brazil, Mato Grosso State, Corguinho, lagoon next to Chacara da Portela Creek. ***Farlowella reticulata*:** AUM 36208, 4 alc, Guyana, Rupununi-Essequibo drainage, Region 9 upper Takutu and Essequibo River. AUM 36210, 10 alc, 1 c&s, Guyana, Rupununi-Essequibo drainage, Region 9, Takutu River, Branco River, Amazon Basin. ***Farlowella rugosa*:** AUM 48805. 10 alc, 1 c&s. Guyana, Rupununi-Essequibo River drainage, Region 9 upper Takutu and Essequibo Rivers, Rupununi River, at Massara landing. ***Farlowella schreitmuelleri*:** FMNH 106985, 2 alc, 1 c&s, Bolivia, Pando Province, Garape Preto, small river at bridge and above on road to Cobija. ***Farlowella smithi*:** MCP 22491, 6 alc, 1 c&s, Brazil, Pará State, Castanhal, Apeú Creek, at Belém-Brasília BR-010 highway, tributary of Guamá River. ***Farlowella venezuelensis*:** USNM 163179, 2 alc, 1 c&s, Venezuela, Monagas State, Caicara, Guarapiche River. ***Farlowella vittata*:** AUM 27727, 12 alc, 1 c&s, Venezuela, Portuguesa State, Apure-Orinoco River system, Las Marias channel at town of Quebrada Seca, approximately 45 min upstream by car from hwy 5, 22 km NNW of Guanare. ***Harttia carvalhoi*:** MCP 18055, 10 alc, 1 c&s, Brazil, Minas Gerais State, Frei Inocêncio, Suaçuí River, tributary of Doce River, on bridge of BR-116 highway at Frei Inocêncio. ***Harttia dissidens*:** MNRJ 35543, 20 alc, 1 c&s, Brazil, Pará State, Rurópolis, Tapajos River basin, Tamber stream, tributary of Cupari River. ***Harttia duriventris*:** MZUSP 34229, 11 alc, 1 c&s, Brazil, Pará State, Tocantins River basin, Itacaiunas River, Serra dos Carajas. ***Harttia fluminensis*:** FMNH 116944, 22 alc, 1 c&s, Suriname, Rapids of Sidonkrutu. ***Harttia fowleri*:** MHNG 2682.038, 1 alc, 1 c&s of 13 alc, French Guiana, St. Georges-Oyapok, Oyapok River basin, downstream of creek opposite to Roche-Mon-Père at about 1 h canoe downstream of Camopi and 15 min downstream of Sikini Creek. ***Harttia garavelloi*:** MZUSP 94432, 3 alc, 1 c&s, Brazil, Minas Gerais State, Minas Novas, São Francisco River basin, Fanado River at Minas Novas, on bridge at exit from Minas Novas to Turmalina. ***Harttia gracilis*:** MZUSP 99678, 28 alc, 1 c&s, Brazil, São Paulo State, São Bento do Sapucaí, Ribeirão do Lajeado River, tributary of Sapucaí River at San José da Rosa. ***Harttia guianensis*:** ANSP 187328, 24 alc, Suriname, Sipalawini District, Marowijne River drainage, Lawa River, base camp ca 8 km SSW of Anapaike/Kawemhakan airstrip. MHNG 2643.033, 1 c&s, French Guyana, Approuague River. ***Harttia kronei*:** MCP 20148, 25 alc, 1 c&s, Brazil, Paraná State, Rio Branco do Sul, Ribeira do Iguape River basin, Piedade River at road from Rio Branco do Sul to Açungui. ***Harttia leiopleura*:** MNRJ 12140, paratypes, 3 of 12 alc, Brazil, Minas Gerais State, Nova Lima, Velhas River basin, creek tributary to Mutuca Creek. MZUSP 109426, 9 alc, 1 c&s, Brazil, Minas Gerais State, Ouro Preto, São Francisco River basin, da Prata River, tributary of Velhas River. ***Harttia longipinna*:** MCP 16686, 2 alc, Marmelada River on road between Pompeu and Frei Orlando, Abaeté, Minas Gerais, São Francisco River asin MCP 24232, 2 alc, 1 c&s, Brazil, Mato Grosso State, São Francisco River basin, at São Francisco River. ***Harttia loricariformis*:** MCP 11707, 1 alc, 1 c&s, Brazil, Rio de Janeiro State, Barra do Piraí, Paraiba do Sul River at road between Piraí and Vassouras. UFRGS 18816, 4 alc, Brazil, Rio de Janeiro State, Teresópolis, river downstream from Venda Nova at road next to BR-492 highway. ***Harttia novalimensis*:** MNRJ 23962, 21 alc, 1 c&s, Brazil, São Paulo State, Campos do Jordão, Sapuca River. ***Harttia punctata*:** MCP 15857, 1 alc, 1 c&s, Brazil, Goiás State, Uruaçu, Tocantins-Maranhão River system, Tocantins River basin, Passa Três River, approximately 2km N of Uruaçu at Belém-Brasilia hwy (BR-153). MCP 45591, 2 alc, Brazil, Goiás State, Nova Roma, Tocantins River basin, das Pedras I River. ***Harttia rhombocephala*:** MCP 16007, 3 alc, 1 c&s, Brazil, Goiás State, Niquelândia, Tocantins River basin, Arara River, 500m from mouth of Maranhão River at Rosariana. ***Harttia surinamensis*:** FMNH 116942, 4 alc, 1 c&s, Suriname, rapids of middle Coppename River. ***Harttia torrenticola*:** MNRJ 12144, paratypes, 2 of 20 alc, 1 c&s, Brazil, Minas Gerais State, Moeda, Paraopeba River system, stream tributary of Paraopeba River, Pedra Vermelha at km 10 of BR-040 highway. ***Harttia trombetensis*:** MHNG 2551.071, 4 alc, 1 c&s, Brazil, Pará State, Cachoeira Porteira, Trombetas River basin. ***Harttiella crassicauda*:** AUM 50387, 23 alc, 1 c&s, Suriname, Sipaliwini District, Marowijne (Maroni) River, Paramaka Creek, Ijs kreek from road on top of plateau to near base of waterfall after edge, to 3.5 km NE of Suralco Base Camp, Nassau Mountain. ***Harttiella intermedia*:** MHNG 2713.087, paratypes, 2 alc, French Guiana, Sinnamary River basin, Trinité Mountains, Crique Grand Leblond. ***Harttiella janmoli*:** MHNG 2695.059, paratypes, 36 alc, French Guiana, Maroni River basin, Kotika Mountain. ***Harttiella longicauda*:** MHNG 2699.070, paratypes, 23 alc, French Guiana, Trinité Mountains, Mana River basin, in tributary of Crique Baboune, Crique Aya around 100 mts in front of Aya Camp. MHNG 2723.042, 2 of 27 alc, 1 c&s, French Guyana, Approuague River, Cascade Creek & Dam Creek, Arataï River. ***Harttiella lucifer*:** MHNG 2721.088, paratypes, 4 alc, French Guiana, Mana River basin, Lucifer Mountains, West of Crique Cascade. ***Harttiella parva*:** MHNG 2723.093, paratypes, 3 alc, French Guiana, Maroni River drainage, Atachi Bakka Mountains. ***Harttiella pilosa*:** MHNG 2724.004, holotype, French Guiana, Tortue Mountains, Orapu River drainage in Crique Grillon at the ONF camp. MHNG 2682.055, paratypes, 4 alc, same data as holotype. MHNG 2724.002, paratype, 1 alc, French Guiana, Tortue Mountains, Orapu River drainage in Crique Grillon at the ONF camp. ***Hemiodontichthys acipenserinus*:** MCP 21975, 6 alc, 1 c&s, Brazil, Maranhão State, Santa Inês, Norte River basin, Pindaré River, W of Santa Ines at Pará-Maranhão BR-316 highway, tributary of Mearim River. ***Lamontichthys avacanoeiro*:** MNRJ 18553, paratypes, 6 alc, 1 c&s, Brazil, Goiás State, Tocantins River basin, pools below UHE Serra da Mesa. MNRJ 23643, 2 alc, Brazil, Goiás State, Niquelandia, Tocantins River basin, Trairas River. ***Lamontichthys filamentosus*:** AUM 45589, 7 alc, 1 c&s, Peru, Amazonas Province, Amazonas River basin, Marañón River, pongo above Borja, 35.5 km NE of Juan Velasco (Sta Maria de Nieva). ***Lamontichthys llanero*:** AUM 22108, 1 alc, 1 c&s, Venezuela, Portuguesa State, Apure-Orinoco River system, Portuguesa River at highway 5. AUM 22791, 3 alc, Venezuela, Portuguesa State, Apure-Orinoco River system, Portuguesa River at highway 5. ***Lamontichthys parakana*:** MNRJ 13300, 6 alc, 1 c&s, Brazil, Goiás State, Minaçu/Cavalcante, Tocantins River basin, at future location of Serra da Mesa hydroelectric dam. ***Limatulichthys griseus*:** MCP 21987, 12 alc, 1 c&s, Brazil, Pará State, Ourém, Amazonas River basin, Guamá River at Tupinambá on road between São Miguel do Guamá and Ourém. ***Loricaria lundbergi*:** MCP 36565, 5 alc, 1 c&s, Brazil, Mato Grosso State, Pontes e Lacerda, Madeira River basin, Bugre River, about 42 km N of Guaporé River on BR-174 highway. ***Loricariichthys anus*:** MCP 11221, 2 alc, 1 c&s, Brazil, Rio Grande do Sul State, Cidreira, Cidreira Lagoon. ***Loricariichthys platymetopon*:** MCP 36443, 7 alc, 1 c&s, Brazil, Mato Grosso State, Poconé, Paraguay River basin, channel on Transpanteneira road towards Porto Manga, about 16 km from Poconé. ***Metaloricaria nijsseni*:** ROM 98120, 8 alc, 1 c&s, Suriname, Nickerie District, Nickerie River. ***Metaloricaria paucidens*:** ANSP 187327, 1 alc, Suriname, Sipalawini District, Lawa River, Marowijne drainage, large cataract complex in side channel west of base camp (SUR 07–01), about 8 km SW of Anapaike. ROM 97928, 1 alc, 1 c&s, Suriname, Marowijne River. ***Pseudohemiodon lamina*:** MCP 36580, 1 alc, 1 c&s, Brazil, Mato Grosso State, Nova Lacerda, Madeira River basin, Galera River, tributary of Guaporé River at Galera balneary. ***Pterosturisoma microps*:** MCP 33231, 1 alc, Brazil, Amazonas State, Alvarães, Solimões River basin, Caborini beach at confluence of Japurá-Solimões rivers. MHNG 2677.072, 1 of 4 alc, Peru, Amazonas River basin. MZUSP 79909, 1 c&s, Brazil, Amazonas State, Amazonas River basin, Solimões River below Içá River, below Paraná do Jarimirim. ***Rineloricaria cadeae*:** MCP 25920, 30 alc, 1 c&s, Brazil, Rio Grande do Sul State, Lavras do Sul, Camaquã River basin, Mantiqueira Creek. ***Rineloricaria lanceolata*:** MCP 36454, 10 alc, 2 c&s, Brazil, Mato Grosso State, Nova Lacerda, Madeira River basin, Retiro Creek tributary of Guaporé River at BR-174 highway. ***Rineloricaria quadrensis*:** MCP 11039, 40 alc, 1 c&s, Brazil, Santa Catarina State, Gravatal, Capivari River at road Gravatal-Armazém. ***Spatuloricaria puganensis*:** AUM 45638, 7 alc, 1 c&s, Peru, Amazonas Province, Amazonas River basin, Marañón River, pongo Renema, purchased at Bagua Chica fish market. ***Sturisoma barbatum*:** MCP 36446, 2 alc, 1 c&s, Brazil, Rondonia State, Ji-Paraná, Madeira River basin, Machado River. ***Sturisoma brevirostre*:** MCZ 8095, holotype, Brazil, Amazonas State, Amazonas River basin, Iça River, tributary of Solimões River. ***Sturisoma graffini*:** MUSM 58700, holotype, Peru, Amazonas State, Madre de Dios River basin, Picaflor Creek, at Pakitza guard post. ROM 64044, paratype, 1 c&s, same data as holotype. USNM 263920, paratypes, 3 alc, Peru, Amazonas State, Manu Province, Madre de Dios River basin, Tambopata River, opposite boat landing for Explorer’s Inn. ***Sturisoma guentheri*:** USNM 324250, 3 alc, 1 c&s, Peru, Amazonas State, Amazonas River basin, Madre de Dios Region, Manu, Pakitza, Martin Pescador Creek. ***Sturisoma lyra*:** MCP 45730, 2 alc, 1 c&s, Peru, Ucayali Province, Purus River basin, Novia Creek, 2 km above mouth. ***Sturisoma monopelte*:** AUM 47893, 9 alc, 1 c&s, Guyana, Essequibo River drainage, Rupununi River at Yupukari, sidewater bay, Region 9 (upper Takutu, upper Essequibo). ***Sturisoma nigrirostrum*:** ANSP 199936, 1 alc, 1 c&s, Peru, Loreto Province, Amazon River basin, Nanay River, just downstream of sandy beach (Las Camelias) along left bank, 7 km W of Iquitos. ***Sturisoma robustum*:** MCP 15812, 8 alc, 1 c&s, Brazil, Mato Grosso State, Cáceres, Paraguay River basin in Cáceres. ***Sturisoma rostratum*:** MCP 36445, 8 alc, 1 c&s, Brazil, Rondonia State, Ji-Paraná, Miolo Creek 15 km NW of Ji-Paraná on BR-364 highway. ***Sturisoma* aff. *tenuirostre*:** USNM 258280, 4 alc, 1 c&s, Venezuela, Apure State, Orinoco River basin, main channel of Apure River in region of San Fernando de Apure. ***Sturisomatichthys aureus*:** NRM 15150, 4 alc, 1 c&s, Colombia, Chocó Department, Rio Baudó basin, Boca de Pepé, various tributaries and river close to village. ***Sturisomatichthys caquetae*:** ANSP 71719, holotype, Colombia, Caquetá Department, Morelia, upper Amazon basin, Caquetá River. ***Sturisomatichthys citurensis*:** USNM 78364, 7 alc, Panama, Darien Province, Chepo District, Mamon River. USNM 78365, 1 alc, 1 c&s, Panama, Darien Province, Tuyra River at Marrigante. ***Sturisomatichthys dariensis*:** STRI 8386, 1 alc, Panama, Darien Province, Chucunaque River basin, Tupisa River. USNM 78373, 2 alc, Panama, Darien Province, Yape River. USNM 293273, 1 c&s, Panama, Darien Province, Rio Tuyra 2–3 km above Pinogana. ***Sturisomatichthys festivus*:** CAS 136506, paratypes, 4 alc, Venezuela, Trujillo State, Lake Maracaibo basin, Monay River, 35 km N of Trujillo. CAS 168512, 1 c&s, Venezuela, Lake Maracaibo basin, Motatan River. ***Sturisomatichthys frenatus*:** CAS 13643, 3 alc, Colombia, Nariño Department, Patia River between Magui and Telembi rivers. USNM 341993, 2 of 3 alc, 1 c&s, Colombia, Nariño Department, Teresita District, Salado River. ***Sturisomatichthys kneri*:** MCNG 33535, paratype, 1 c&s, Venezuela, Zulia State, Lake Maracaibo basin, creek Urumana, Cataneja farm. ***Sturisomatichthys leightoni*:** FMNH 55136, 6 alc, 1 c&s, Colombia, Paila. ***Sturisomatichthys panamensis*:** USNM 293412, 10 of 14 alc, 1 c&s, Panama, Darien Province, Tuira River between Calle Larga and Pinogana, above El Real. ***Sturisomatichthys reinae*:** ICNMHN 24056, holotype, Colombia, Chocó Department, Baudó River drainage, Boca de Pepé, various tributaries and river close to village. MCP 54152, paratypes, 2 alc, same data as holotype; NRM 15155, paratypes, 10 alc, 1 c&s, same data as holotype. ***Sturisomatichthys tamanae*:** ANSP 198426, 1 alc, Colombia, Chocó Department, San Juan River basin. CAS 67414, 2 of 15 alc, 1 c&s, Colombia, Chocó Department, Istmina, San Juan River basin. ***Sturisomatichthys varii*:** CAS 246603, paratypes, 6 alc, 1 c&s, Colombia, Chocó Department, Istmina, San Juan River basin.

**Outgroup: *Acestridium scutatum*:** MCP 37785, paratypes, 9 alc, 2 c&s, Brazil, Amazonas State, Humaitá, Madeira River basin, Traíra River about 35 km E of Madeira River. ***Ancistrus brevipinnis*:** MCP 21449, 12 alc, 1 c&s, Brazil, Rio Grande do Sul State, Tapera, Jacui River basin, Colorado River at Ibirubá-Tapera road. ***Chaetostoma breve*:** AUM 46515, 40 alc, 1 c&s, Peru, Amazonas Province, Amazon River basin, Marañón River basin, Utcubamba River 23 km SE of Bagua Chica. ***Hemipsilichthys gobio*:** MCP 19780, 7 alc, 2 c&s, Brazil, São Paulo State, Silveiras, Paraiba do Sul River basin, Macaquinho Creek, tributary of Paraitinga River, ca 5 km NW of Bairro dos Macacos. ***Hisonotus laevior*:** 56 alc, 4 c&s, Brazil, Rio Grande do Sul State, Pedro Osório, São Gonçalo River, Arambaré Creek, about 5 km S of Vila Brasílio, at road towards Pedro Osório. ***Neoplecostomus microps*:** MCP 42432, 5 alc, 1 c&s, Brazil, São Paulo State, Pindamonhangaba, Paraiba do Sul River basin, Ribeirão Grande River, at Ribeirão Grande on road of Nova Gokula Hare Krishna Temple. ***Pareiorhaphis calmoni*:** MCP 17276, 15 alc, 1 c&s, Brazil, Santa Catarina State, Águas Mornas, Teresópolis River, tributary of Cubatão River. ***Parotocinclus maculicauda*:** MCP 29086, 17 alc, 2 c&s, Brazil, Santa Cataina State, Itajaí, creek tributary of Meio River, ca 5 km of BR-486 highway towards Meio River. ***Pterygoplichthys lituratus*:** MCP 35757, 2 alc, 1 c&s, Brazil, Mato Grosso State, Pontes e Lacerda, Madeira River basin, river tributary of Guaporé River at BR-174 highway, between Pontes e Lacerda and Comodoro.

## Supporting information

S1 File Classification of the Loricariinae by sequencying (Wiley [55]), based on the Bayesian analysis topology and including synonymy, composition, list of synapomorphies, and distribution. Synapomorphies from the Maximum Parsimony analysis. Characters in bold represent exclusive synapomorphies.
(PDF)

S2 File List of molecular transformations for clades in Loricariinae based on the Maximum Parsimony analysis. Characters in bold represent exclusive molecular synapomorphies.
(PDF)

S3 File Phenotypic partition of the data matrix (osteology and external morphology). Missing taxa in the partition are represented by DNA data only.
(PDF)

S1 Fig Strict consensus cladogram from the Maximum Parsimony analysis. Numbers above branches are Bremer support values. Type-species names in bold.
(PDF)

S2 Fig Total evidence Maximum Likelihood Tree. Numbers at branches are bootstrap frequencies.
(PDF)

S3 Fig DNA-only Bayesian Tree. Numbers at branches are Posterior Probabilities.
(PDF)
