# Supplementary material for: A combined molecular and morphological phylogeny of the Loricariinae (Siluriformes: Loricariidae), with emphasis on the Harttiini and Farlowellini
Source: PLoS One. 2021 Mar 15;16(3):e0247747. doi: 10.1371/journal.pone.0247747 (PMC7959404; doi:10.1371/journal.pone.0247747)
Supplement: S1 File — Synapomorphies from the Maximum Parsimony analysis. Characters in bold represent exclusive synapomorphies. (PDF) [file pone.0247747.s001.pdf]

Supplementary Material 1

Classification of the Loricariinae by sequencying (Wiley [55]) based on the Bayesian analysis topology and including synonymy, composition, list of morphological synapomorphies, and distribution. Synapomorphies from the Maximum Parsimony analysis. Characters in bold represent exclusive synapomorphies.

Loricariinae Bonaparte, 1831

Immediately more inclusive taxon: Loricariidae Rafinesque, 1815

Composition: Three tribes: Harttiini Boeseman, 1971; Loricariini Bonaparte, 1831; Farlowellini Fowler, 1958.

Branch length: 76 synapomorphies diagnose this subfamily.

Synapomorphies: 24 morphological and 52 molecular.

Phenotypic characters that diagnose this node are:

Char. 5: 0→1: Mesethmoid, ventral disk, shape; robust, circular in lateral view, without fenestra.
Char. 19: 0→3: Frontal, dorsal border of orbit, participation; extensive participation, half of dorsal border of orbit.
Char. 29: 0→1: Hyomandibula, articulation with cranium, contribution of compound pterotic; reduced, shorter than prootic.
Char. 34: 0→2: Canal-bearing cheek plate, position on head; ventral.
Char. 38: 0→4: Parietal branch, terminal exit; on sphenotic.
Char. 40: 0→2: Lateral line, length; extended, lateral line ending on one a supracaudal plate.
Char. 83: 0→1: Anterohyal, anterior margin, expansion; slightly expanded.
Char. 114: 0→2: Sixth vertebra, neural spine, lamina for articulation with ventral surface of supraoccipital; reduced.
Char. 128: 0→3: Precaudal vertebrae, number; 11.
Char. 130: 0→2: First caudal vertebra, bifid hemal spine; long, approximately as long as corresponding vertebra.
Char. 131: 0→1: Caudal vertebral centra, paraneural and parahemal spines; present.
Char. 134: 0→1: Second preural centrum, hemal spine, cartilage on posterior tip; absent.
Char. 136: 0→1: Caudal peduncle, cross-section; depressed.
Char. 142: 0→1: Cleithrum, posterior process, shape; short, narrow portion exposed.
Char. 158: 0→1: Anal fin, first pterygiophore, centrum of articulation; centra 12, 13 or 14.
Char. 160: 0→2: Hypural plate, upper and lower lobes, shape; symmetric, posterior border *V*-shaped.
Char. 163: 0→2: Epural, size; not a separate element.
Char. 164: 0→2: Second preural centrum, apophysis; absent.
Char. 167: 0→1: Hypural plates, posterior margin; notch and fenestra.
Char. 182: 0→1,2: Lateral abdominal plates, shape; present, flat; present, angled.
Char. 188: 0→1: Adipose fin; absent.

Char. 190: 0→1: Supracaudal plates; elongate and few.
Char. 191: 0→1: Caudal fin, number of branched rays; 12 rays.
Char. 193: 0→1: Body coloration, pattern; bars.

Distribution: South America, from Panama to Argentina, excluding Chile and Patagonia. South American main river systems: Orinoco, Amazonas, Tocantins, Xingú, Tapajós, São Francisco, Tocantins, Madre de Dios, Essequibo, Oyapok, Cupe, Bayano, and Tuyra Rivers, which drain to the Pacific and Atlantic Ocean in Panama. The Atrato, San Juan, Baudó, Patía, Sinú, Magdalena, Cauca, Catatumbo rivers that drain to the Caribbean and Pacific Ocean in Colombia. Esmeraldas River in Ecuador. Lake Maracaibo and Valencia basin in Venezuela.

Harttiini Boeseman, 1971

Immediately more inclusive taxon: Loricariinae Bonaparte, 1831

Sister group: Loricariini and Farlowellini.

Composition: Three genera: *Cteniloricaria* Isbrücker & Nijssen, 1979; *Harttia* Steindachner 1877; and *Harttiella* Boeseman, 1971.

Branch length: 38 synapomorphies diagnose this tribe.

Synapomorphies: Nine morphological and 29 molecular.

Phenotypic characters that diagnose this node are:
Char. 33: 0→2: Preopercle, sensory canal shape; curved and branched, three exits.
Char. 62: 0→1: Metapterygoid, canal shape; deep, partially covered by bony shelf.
Char. 67: 1→2: Symphyseal cartilage, size; short, half quadrate posterior border.
Char. 92: 0→1: Ceratobranchial 1, accessory flange, size; large, same size or larger than ceratobranchial.
Char. 107: 02→1: Upper pharyngeal plate, shape; *L*-shaped.
Char. 110: 0→1: Transcapular ligament, connection to parapophysis of complex centrum; not connected.
Char. 122: 0→1: Dorsal fin, pterygiophores, extent of contact; first and second pterygiophores in contact, others separated.
Char. 138: 0→1: Cleithrum, anterior margin; slightly curved.
Char. 152: 0→1: Basipterygium, anterolateral process, shape; round and broad.

Distribution: South America, including Brazil, Guiana, French Guiana, Suriname, and Venezuela. River basins: Amazonas, Orinoco, Tapajós, Tocantins, Paraná, São Francisco, Paraíba do Sul, Caura, Marowijne, Essequibo, Oyapock, Maroni, Corantijn, Paru de Oeste, Suriname, and Coppename.

Harttiella Boeseman, 1971

Harttiella Boeseman, 1971: 25. Fem. Type species: *Harttia crassicauda* Boeseman, 1953.

Immediately more inclusive taxon: Tribe Harttiini Boeseman, 1971

Sister group: *Ctenilorícaria* and *Harttia*.

Composition: Seven species: *Harttiella crassicauda* (Boeseman, 1953); *Harttiella intermedia* Covain & Fisch-Muller, 2012; *Harttiella janmoli* Covain & Fisch-Muller,

2012; *Harttiella longicauda* Covain & Fisch-Muller, 2012; *Harttiella lucifer* Covain & Fisch-Muller, 2012; *Harttiella parva* Covain & Fisch-Muller, 2012; and *Harttiella pilosa* Covain & Fisch-Muller, 2012.

Branch length: 137 synapomorphies diagnose this genus.

Synapomorphies: 137 molecular. No phenotypic synapomorphies were found to diagnose this node; see Discussion of the genus for Diagnosis.

Distribution: South America, Guiana, French Guiana and Suriname: River basins: Sinnamary, Essequibo, Approuague, Coppename, Paru de Oeste, Maroni, Marowijne, and Oyapock.

Cteniloricaria Isbrücker & Nijssen, 1979

Ctenilorícaria Isbrücker & Nijssen, 1979: 88. Fem. Type species: *Loricaria platystoma* Günther, 1868

Immediately more inclusive taxon: Tribe Harttiini Boeseman, 1971

Sister group: *Harttia*.

Composition: Two species: *Ctenilorícaria napova* Covain & Fisch-Muller, 2012 and *Ctenilorícaria platystoma* (Günther, 1868)

Branch length: 95 synapomorphies diagnose this genus.

Synapomorphies: 95 molecular.

No phenotypic synapomorphies were found to diagnose this node; see Discussion of the genus for Diagnosis.

Distribution: South America, Brazil and Suriname: River basins: Upper Paru de Oeste, Erepecuru and Suriname.

Harttia Steindachner, 1877

Harttia Steindachner, 1877: 668. Fem. Type species: *Harttia loricariformis* Steindachner, 1877.
Quiritixys Isbrücker, in Isbrücker *et al*., 2001: 21. Fem. Type species: *Harttia leiopleura* Oyakawa, 1993.

Immediately more inclusive taxon: Tribe Harttiini Boeseman, 1971

Sister group: *Ctenilorícaria*.

Composition: 26 species: *Harttia absaberi* Oyakawa, Fichberg & Langeani, 2013; *Harttia carvalhoi* Miranda Ribeiro, 1939; *Harttia depressa* Rapp Py-Daniel & Oliveira, 2001; *Harttia dissidens* Rapp Py-Daniel & Oliveira, 2001; *Harttia duriventris* Rapp Py-Daniel & Oliveira, 2001; *Harttia fluminensis* Covain & Fisch-Muller, 2012; *Harttia fowleri* (Pellegrin, 1908); *Harttia garavelloi* Oyakawa, 1993; *Harttia gracilis* Oyakawa, 1993; *Harttia guianensis* Rapp Py-Daniel & Oliveira, 2001; *Harttia intermontana* Oliveira & Oyakawa, 2019; *Harttia kronei* Miranda Ribeiro, 1908; *Harttia leiopleura* Oyakawa, 1993; *Harttia longipinna* Langeani, Oyakawa & Montoya-Burgos, 2001; *Harttia loricariformis* Steindachner, 1877; *Harttia merevari* Provenzano, Machado-Allison, Chernoff, Willink & Petry, 2005; *Harttia novalimensis* Oyakawa, 1993; *Harttia panara* Oyakawa, Fichberg & Rapp Py-Daniel, 2018; *Harttia punctata* Rapp

Py-Daniel & Oliveira, 2001; *Harttia rhombocephala* Miranda Ribeiro, 1939; *Harttia rondoni* Oyakawa, Fichberg & Rapp Py-Daniel, 2018; *Harttia surinamensis* Boeseman, 1971; *Harttia torrenticola* Oyakawa, 1993; *Harttia trombetensis* Rapp Py-Daniel & Oliveira, 2001; *Harttia tuna* Covain & Fisch-Muller, 2012; *Harttia uatumensis* Rapp Py-Daniel & Oliveira, 2001; and *Harttia villasboas* Oyakawa, Fichberg & Rapp Py-Daniel, 2018.

Branch length: 29 synapomorphies diagnose this genus.

Synapomorphies: Six morphological and 23 molecular.

Phenotypic characters that diagnose this node are:
- Char. 39: 0→1: Parietal branch, shape; curved.
- Char. 47: 1→0: Dentary, coronoid process; large, with small robust area.
- Char. 87: 1→0: Posterohyal, dorsal hook; large, projected.
- Char. 112: 0→1: Transverse process of complex centrum, type of contact to compound pterotic; suture.
- Char. 134: 1→0: Second preural centrum, hemal spine, cartilage on posterior tip; present.
- **Char. 189: 0→1: Caudal peduncle, width; narrows abruptly towards caudal-fin base.**

Distribution: South America, Brazil, French Guiana, Suriname, Venezuela: River basins: Paraíba do Sul, Uatumã, Tapajós, Tocantins, Fanado, Araçuaí, São Joao, São Francisco, Trombetas, Amazon basin; Paraná, La Plata basin; Sinnamary, Approuague, Coppename, Paru de Oeste, Maroni, Marowijne, Oyapock, Suriname and French Guiana; Upper Caura, Orinoco basin.

Farlowellini Fowler, 1958

Immediately more inclusive taxon: Loricariinae Bonaparte, 1831

Composition: Five genera: *Farlowella* Eigenmann & Eigenmann, 1889; *Lamontichthys* Miranda Ribeiro, 1939; *Pterosturisoma* Isbrücker & Nijssen, 1978a; *Sturisoma* Swainson 1838; and *Sturisomatichthys* Isbrücker & Nijssen, 1979.

Branch length: 49 synapomorphies diagnose this subtribe.

Synapomorphies: 11 morphological and 38 molecular.

Phenotypic characters that diagnose this node are:
- Char. 25: 0→1: Basioccipital, articulation to transcapular ligament; present.
- Char. 38: 4→3: Parietal branch, terminal exit; on supraoccipital.
- Char. 62: 0→2: Metapterygoid, canal shape; deep, totally covered by bony shelf.
- Char. 95: 0→2: Lower pharyngeal tooth plate, shape; trapezoidal.
- Char. 123: 0→1: Dorsal fin, second pterygiophore, lateral process, orientation; strongly curved, anterolaterally oriented.
- Char. 146: 0→1: Coracoid and cleithrum, symphysis, fenestra; present.
- Char. 154: 1→2: Basipterygium, anterolateral process, laminar expansions, relative width; dorsal and ventral of similar width.
- Char. 159: 0→1: Anal fin, anterior three pterygiophores, adjacent proximal portions, relative distance; relatively close.
- **Char. 175: 0→1: Nuchal plate, articulation to surrounding plates; articulated to lateral plates.**

Char. 179: 0→1: Gular plates; present.
Char. 185: 0→2: Pelvic fin, length; conspicuously surpassing anal-fin origin.

Distribution: South America, from Panama to Argentina, excluding Chile and Patagonia. River basins: Amazonas, Orinoco, Tocantins, La Plata, Xingú, Guaviare, Paraguay, Paraná, Araguaia, Negro, Madeira, Guaripiche, Ucayali, Madre de Dios, Huallaga, Juruá, and Meta. Magdalena, Cauca, Atrato, Baudó, San Juan, Sinú, Catatumbo, San Jorge, rivers draining to the Pacific and Caribbean oceans in Colombia. Napo, Pastaza, Aguarico, and Pacific Versant Rivers of Ecuador. Essequibo, Marowijne, Oyapock, Corantijn, and Rupununi Rivers, French Guiana and Suriname. Tuyra, Bayano, Chucunaque, Cupe River basins in Panama, including tributaries of the Darien region. Lake Valencia and Maracaibo Basin, and Mavaca River basin in Venezuela.

Lamontichthys Miranda Ribeiro, 1939

Lamontichthys Miranda Ribeiro, 1939: 12. Masc. Type species: *Harttia filamentosa* La Monte, 1935

Immediately more inclusive taxon: Tribe Farlowellini Fowler, 1958

Sister group: *Pterosturisoma*, *Sturisoma*, *Sturosomatichthys*, and *Farlowella*.

Composition: Six species: *Lamontichthys avacanoeiro* Paixão & Toledo-Piza, 2009; *Lamontichthys filamentosus* (Eigenmann & Allen, 1942); *Lamontichthys llanero* Taphorn & Lilyestrom, 1984; *Lamontichthys maracaibero* Taphorn & Lilyestrom, 1984; *Lamontichthys parakana* Paixão & Toledo-Piza, 2009; and *Lamontichthys stibaros* Isbrücker & Nijssen, 1978.

Branch length: 10 synapomorphies diagnose this genus.

Synapomorphies: 10 morphological.

Phenotypic characters that diagnose this node are (exclusive synapomorphies in bold):
Char. 59: 2→1: Premaxilla, size; larger than autopalatine.
Char. 67: 1→2: Symphyseal cartilage, size; short, half quadrate posterior border.
Char. 83: 1→2: Anterohyal, anterior margin, expansion; not expanded.
Char. 110: 0→1: Transcapular ligament, connection to parapophysis of complex centrum; not connected.
Char. 116: 0→1: Seventh vertebra, lateral portion, flange; present.
Char. 122: 0→1: Dorsal fin, pterygiophores, extent of contact; first and second pterygiophores in contact, others separated.
Char. 137: 0→1: Pectoral fin, branched rays, number; seven.
Char. 164: 2→1: Second preural centrum, apophysis; two, well developed.
Char. 166: 2→1: Second preural centrum, length; equal to or slightly longer than hypural plate.
Char. 180: 2→1: Central abdominal plates, shape; very small, irregular, closely packed.

Distribution: South America, Brazil, Ecuador, Peru and Venezuela: River basins: Tocantins, Huallaga, Jurua, Pastaza, Amazon basin; Guanare Viejo, Orinoco basin; Motatán River and Lake Maracaibo basin.

Pterosturisoma Isbrücker & Nijssen, 1978

Pterosturisoma Isbrücker & Nijssen, 1978: 69. Neut. Type species: *Harttia microps* Eigenmann & Allen 1942

Immediately more inclusive taxon: Tribe Farlowellini Fowler, 1958

Sister group: *Sturisoma*, *Sturisomatichthys*, and *Farlowella*.

Composition: One species: *Pterosturisoma microps* (Eigenmann & Allen, 1942)

Branch length: 155 autapomorphies diagnose this genus.

Autapomorphies: 17 morphological and 138 molecular.

Phenotypic characters that diagnose this node are:
- Char. 13: 1→4: Lateral ethmoid, laterodorsal lamina; absent.
- Char. 20: 0→1: Basioccipital, lateral process; small, shorter than basioccipital length.
- Char. 21: 1→2: Exoccipital, ventral lamina, ventral expansion; lamina absent.
- Char. 59: 2→0: Premaxilla, size; same size as autopalatine.
- Char. 78: 0→1: Preopercle, connection to dermal plates; partially sutured at dorsal ridge.
- Char. 92: 0→2: Ceratobranchial 1, accessory flange, size; absent or very small, inconspicuous.
- Char. 97: 0→3: Lower pharyngeal tooth plate, teeth, distribution; teeth lacking.
- Char. 99: 0→1: Epibranchial 1, posterior process, shape; relatively elongated and triangular.
- Char. 102: 0→1: Epibranchial 2, posterior process, shape; absent.
- Char. 128: 3→2: Precaudal vertebrae, number; 12.
- Char. 138: 0→1: Cleithrum, anterior margin; slightly curved.
- Char. 150: 2→1: Basipterygium, cartilage, shape; short rectangle.
- Char. 155: 0→3: Basipterygium, posterior process, shape; short, slightly triangular.
- Char. 176: 3→0: Pre-dorsal plates, shape; rectangular.
- Char. 177: 01→2: Dorsal-fin base, number of flanking plates; six.
- Char. 192: 0→2: Upper caudal-fin ray, filament; exceeding body length.
- Char. 194: 0→1: Dorsal fin, dark band; present.

Distribution: South America, Bolivia and Peru: River basins: Upper Amazonas, Iquitos.

Sturisoma Swainson, 1838

Sturisoma Swainson, 1838: 333. Neut. Type species: *Loricaria rostrata* Spix & Agassiz, 1829
Oxyloricaria Bleeker, 1862:3. Fem. Type species: *Loricaria barbata* Kner 1853
Parasturisoma Miranda Ribeiro, 1911:109. Neut. Type species: *Loricaria brevirostris* Eigenmann & Eigenmann 1889.

Immediately more inclusive taxon: Tribe Farlowellini Fowler, 1958

Sister group: *Sturisomatichthys* and *Farlowella*.

Composition: Ten species: *Sturisoma barbatum* (Kner, 1853); *Sturisoma brevirostre* (Eigenmann & Eigenmann, 1889); *Sturisoma graffini* Londoño-Burbano, 2018; *Sturisoma guentheri* (Regan, 1904); *Sturisoma lyra* (Regan, 1904); *Sturisoma monopelte* Fowler, 1914; *Sturisoma nigrirostrum* Fowler, 1940; *Sturisoma robustum*

(Regan, 1904); *Sturisoma rostratum* (Spix & Agassiz, 1829); and *Sturisoma tenuirostre* (Steindachner, 1910).

Branch length: 59 synapomorphies diagnose this genus.

Synapomorphies: 11 morphological and 48 molecular.
Phenotypic characters that diagnose this node are:

- Char. 5: 1→2: Mesethmoid, ventral disk, shape; circular lamina.
- Char. 26: 0→2: Basioccipital, lateral process, angle; posterolaterally oriented.
- Char. 31: 0→1: Compound pterotic, junction of hyomandibula; reduced contribution, less than half that of prootic.
- Char. 80: 1→0: Infraorbital and supraorbital canals, point of bifurcation; on sphenotic.
- Char. 83: 1→2: Anterohyal, anterior margin, expansion; not expanded.
- **Char. 107: 2→3: Upper pharyngeal plate, shape; triangular.**
- Char. 118: 0→2: Connecting bone, dorsal contact; not contacting pterygiophores.
- **Char. 119: 0→1: Complex centrum, ventral process, position; at middle of complex centrum.**
- Char. 142: 1→2: Cleithrum, posterior process, shape; short, broad portion exposed.
- Char. 148: 2→0: Basipterygium, anteromesial processes, anterior contact; in contact along their entire medial margins.
- Char. 172: 0→1: Rictal barbel, length; half orbit diameter.

Distribution: South America, Argentina, Bolivia, Brazil, French Guiana, Paraguay, Peru, Venezuela: River basins: Solimões, Madre de Dios, Juruá, Ucayali, Amazon basin; Essequibo; Paraguay, La Plata basin; Meta River, Orinoco basin.

Sturisomatichthys Isbrücker & Nijssen, 1979

Sturisomatichthys Isbrücker & Nijssen, 1979: 91. Masc. Type species: *Oxyloricaria leightoni* Regan, 1912

Immediately more inclusive taxon: Tribe Farlowellini Fowler, 1958

Sister group: *Farlowella*.

Composition: 13 valid species: *Sturisomatichthys aureus* (Steindachner, 1900); *Sturisomatichthys caquetae* (Fowler, 1945) **New Combination;** *Sturisomatichthys citurensis* (Meek & Hildebrand, 1913); *Sturisomatichthys dariensis* (Meek & Hildebrand, 1913); *Sturisomatichthys festivus* (Myers, 1942); *Sturisomatichthys frenatus* (Boulenger, 1902); *Sturisomatichthys guaitipan* Londoño-Burbano & Reis, 2019; *Sturisomatichthys kneri* (Ghazzi, 2005); *Sturisomatichthys leightoni* (Regan, 1912); *Sturisomatichthys panamensis* (Eigenmann & Eigenmann, 1889); *Sturisomatichthys reinae* Londoño-Burbano & Reis, 2019; *Sturisomatichthys tamanae* (Regan, 1912); *Sturisomatichthys varii* Londoño-Burbano & Reis, 2019.

Branch length: 28 synapomorphies diagnose this genus.

Synapomorphies: Nine morphological and 19 molecular.

Phenotypic characters that diagnose this node are:

- Char. 7: 2→1: Mesethmoid, ventrolateral crest; short, on margin of proximal portion.
- Char. 41: 1→0: Autopalatine, posterior process, length; extending beyond anterior condyle of lateral ethmoid.
- Char. 100: 0→1: Epibranchial 1, anterior process; absent.

Char. 108: 1→0: Upper pharyngeal tooth plate, dentition; complete.
Char. 111: 0→1: Transverse process of complex centrum, fenestra with laminar process at limit with basioccipital; present.
Char. 121: 1→2: Dorsal fin, pterygiophores, number; more than eight.
Char. 133: 1→0: Second preural centrum, posterior process of hemal spine, length; long.
Char. 142: 1→0: Cleithrum, posterior process, shape; long, narrow portion exposed.
Char. 194: 0→1: Dorsal fin, dark band; present.

Distribution: South America, Colombia, Ecuador, Panama, Venezuela: River basins: Magdalena, Cauca, Catatumbo, Sinú, San Jorge, Atrato, Baudó, San Juan, Caquetá, Patía, Orinoco, Esmeraldas, Bayano, Tuyra, Cupe, Lake Maracaibo basin.

Farlowella Eigenmann & Eigenmann, 1889

Farlowella Eigenmann & Eigenmann, 1889: 32. Fem. Type species: *Acestra acus* Kner, 1853.
Acestra Kner, 1853:93. Fem. Type species: *Acestra acus* Kner, 1853. Invalid, preoccupied by *Acestra* Bonaparte, 1846 in fishes and by Dallas 1852 in Hemiptera, replaced by *Farlowella* Eigenmann & Eigenmann, 1889.
Aposturisoma Isbrücker, Britski, Nijssen & Ortega, 1983: 34. Neut. Type species: *Aposturisoma myriodon* Isbrücker, Britski, Nijssen & Ortega 1983. NEW SYNONYM.

Immediately more inclusive taxon: Tribe Farlowellini Fowler, 1958

Composition: 32 species: *Farlowella acus* (Kner, 153); *Farlowella altocorpus* Retzer, 2006; *Farlowella amazona* (Günther, 1864); *Farlowella azpelicuetae* Terán, Ballen, Alonso, Aguilera & Mirande, 2019; *Farlowella colombiensis* Retzer & Page, 1997; *Farlowella curtirostra* Myers, 1942; *Farlowella gianetii* Ballen, Pastana & Peixoto, 2016; *Farlowella gladiolus* (Günther, 1864); *Farlowella gracilis* Regan, 1904; *Farlowella hahni* Meinken, 1937; *Farlowella hasemani* Eigenmann & Vance, 1917; *Farlowella henriquei* Miranda Ribeiro, 1918; *Farlowella isbruckeri* Retzer & Page, 1997; *Farlowella jauruensis* Eigenmann & Vance, 1917; *Farlowella knerii* (Steindachner, 1882); *Farlowella mariaelenae* Martín Salazar, 1964; *Farlowella martini* Fernández-Yépez, 1972; *Farlowella mitoupibo* Ballen, Urbano-Bonilla & Zamudio, 2016; *Farlowella myriodon* (Isbrücker, Britski, Nijssen & Ortega, 1983) **New Combination**; *Farlowella nattereri* Steindachner, 1910; *Farlowella odontotumulus* Retzer & Page, 1997; *Farlowella oxyrryncha* (Kner, 1853); *Farlowella paraguayensis* Retzer & Page, 1997; *Farlowella platorynchus* Retzer & Page, 1997; *Farlowella reticulata* Boeseman, 1971; *Farlowella rugosa* Boeseman, 1971; *Farlowella schreitmuelleri* Ahl, 1937; *Farlowella smithi* Fowler, 1913; *Farlowella taphorni* Retzer & Page, 1997; *Farlowella venezuelensis* Martín Salazar, 1964; *Farlowella vittata* Myers, 1942; *Farlowella yarigui* Ballen & Mojica, 2014.

Branch length: 22 synapomorphies diagnose this genus.

Synapomorphies: 14 morphological and eight molecular.

Phenotypic characters that diagnose this node are:
Char. 4: 1→0: Mesethmoid, ventral process; absent,
Char. 51: 0→2: Tooth cusps, shape; pointed.
Char. 83: 1→0: Anterohyal, anterior margin, expansion; greatly expanded.

Char. 109: 0→1: Branchiostegal rays, number; fewer than four.
Char. 114: 2→1: Sixth vertebra, neural spine, lamina for articulation with ventral surface of supraoccipital; straight and upright.
Char. 115: 1→0: Aortic canal, extension; reaching sixth or seventh vertebral centra.
Char. 117: 1→2: Seventh vertebra, pleural rib; absent.
Char. 118: 0→3: Connecting bone, dorsal contact; absent.
Char. 120: 1→2: Dorsal fin, spinelet, shape; absent.
Char. 129: 0→1: Anteriormost paraneural spines in contact with dorsal plates, shape; short.
Char. 143: 1→0: Cleithrum, symphysis, length; similar to coracoid symphysis.
Char. 148: 2→1: Basipterygium, anteromesial processes, anterior contact; in contact anteriorly and posteriorly at midline, with small fenestra in between.
Char. 160: 2→0: Hypural plate, upper and lower lobes, shape; symmetric, posterior border vertically aligned.
Char. 185: 1→0: Pelvic fin, length; not surpassing anal-fin origin.

Distribution: South America, Brazil, Colombia, Ecuador, Peru, Suriname, Venezuela: River basins: Tocantins, Araguaia, Paraguay, Ucayali, Huacamayo, Madeira, Amazon basin; La Plata, Paraná, La Plata basin; Meta River, Orinoco basin; Caquetá, Magdalena Rivers, Colombia; Napo, Pastaza, Aguarico Rivers, Ecuador; Essequibo, Suriname; Lake Valencia and Maracaibo basin, Portuguesa, Mavaca River, Venezuela.

Loricariini Bonaparte, 1831

Immediately more inclusive taxon: Loricariinae Bonaparte, 1831

Composition: *Brochiloricaria* Isbrücker and Nijssen, 1979; *Crossoloricaria* Isbrücker, 1979; *Dasyloricaria* Isbrücker and Nijssen, 1979; *Dentectus* Martín Salazar, Isbrücker and Nijssen, 1982; *Fonchiiloricaria* Rodriguez, Ortega and Covain, 2011; *Furcodontichthys* Rapp Py-Daniel, 1981; *Hemiodontichthys* Bleeker, 1862; *Limatulichthys* Isbrücker and Nijssen, 1979; *Loricaria* Linnaeus, 1758; *Loricariichthys* Bleeker, 1862; *Metaloricaria* Isbrücker, 1975; *Paraloricaria* Isbrücker, 1979; *Planiloricaria* Isbrücker, 1971; *Proloricaria* Isbrücker, 2001; *Pseudohemiodon* Bleeker, 1862; *Pseudoloricaria* Bleeker, 1862; *Pyxiloricaria* Isbrücker and Nijssen, 1984; *Reganella* Eigenmann, 1905; *Rhadinoloricaria* Isbrücker and Nijssen, 1974; *Ricola* Isbrücker and Nijssen, 1978b; *Rineloricaria*, Bleeker, 1862; and *Spatuloricaria* Schultz, 1944.

Branch length: 33 synapomorphies diagnose this subtribe.

Synapomorphies: 24 morphological and nine molecular.

Phenotypic characters that diagnose this node are:
Char. 5: 1→3: Mesethmoid, ventral disk, shape; keel-shaped lamina.
Char. 7: 0→3: Mesethmoid, ventrolateral crest; narrow lamina.
Char. 13: 1→4: Lateral ethmoid, laterodorsal lamina; absent.
Char. 26: 0→1: Basioccipital, lateral process, angle; anterolaterally oriented.
Char. 34: 2→1: Canal-bearing cheek plate, position on head; ventrolateral.
Char. 41: 1→2: Autopalatine, posterior process, length; posterior process absent.
Char. 42: 2→1: Autopalatine, lateral flange; present and complete.
Char. 44: 0→1: Autopalatine, shape; rectangular, straight.
Char. 46: 0→2: Premaxilla, cup-shaped portion, length; distinctly shorter than cup-shaped portion of dentary.

Char. 48: 0→1: Dentary, posteroventral lamina; absent.
Char. 50: 0→1: Dentary, teeth; less than 20 teeth.
Char. 52: 1→2: Dentary teeth, cusp size; inner cusp distinctly longer than outer.
Char. 54: 0→1: Premaxilla, tooth number; fewer than 20.
Char. 57: 0→1: Premaxilla, shape; bony lamina.
Char. 58: 0→1: Premaxilla, cup-shaped region, length; length and width equivalent.
Char. 78: 0→2: Preopercle, connection to dermal plates; preopercle strongly sutured to dermal plates.
Char. 79: 0→1: Suspensorium, overall shape; rectangular.
Char. 92: 0→2: Ceratobranchial 1, accessory flange, size; absent or very small, inconspicuous.
Char. 99: 0→1: Epibranchial 1, posterior process, shape; relatively elongated and triangular.
Char. 108: 1→0: Upper pharyngeal tooth plate, dentition; complete.
Char. 113: 0→2: Transverse process of complex centrum, length; long, surpassing compound pterotic lateral border.
Char. 139: 0→1: Cleithrum, symphysis, type of articulation; simple, not interdigitated.
Char. 143: 1→2: Cleithrum, symphysis, length; twice as long as coracoid symphysis.
Char. 165: 0→1: Second preural centrum, neural and hemal spines; poorly or not expanded.

Distribution: South America, from Panama to Argentina, excluding Chile and Patagonia. South American main river systems: Orinoco, Amazonas, Tocantins, Xingú, Tapajós, São Francisco, Tocantins, Madre de Dios, Essequibo, Oyapok, Cupe, Bayano, and Tuyra Rivers, which drain to the Pacific and Atlantic Ocean in Panama. The Atrato, San Juan, Baudó, Patía, Sinú, Magdalena, Cauca, Catatumbo rivers that drain to the Caribbean and Pacific Ocean in Colombia. Esmeraldas River in Ecuador. Lake Maracaibo and Valencia basin in Venezuela.

Classification of genera and species-group modified from Covain et al. [15].

Metaloricaria group

Metaloricaria Isbrücker, 1975

Dasyloricaria group

Dasyloricaria Isbrücker and Nijssen, 1979

Fonchiiloricaria Rodriguez, Ortega and Covain, 2011

Rineloricaria group

Rineloricaria, Bleeker, 1862

Loricariichthys group

Furcodontichthys Rapp Py-Daniel, 1981 – *Sedis mutabilis*

Loricariichthys Bleeker, 1862

Hemiodontichthys Bleeker, 1862

Limatulichthys Isbrücker and Nijssen, 1979

Pseudoloricaria Bleeker, 1862

Spatuloricaria group

Spatuloricaria Schultz, 1944

Pseudohemiodon group

Dentectus Martín Salazar, Isbrücker and Nijssen, 1982 – *Sedis mutabilis*

Reganella Eigenmann, 1905 – *Sedis mutabilis*

Crossoloricaria Isbrücker, 1979

Planiloricaria Isbrücker, 1971

Pseudohemiodon Bleeker, 1862

Rhadinoloricaria Isbrücker and Nijssen, 1974

Loricaria Group

Pyxiloricaria Isbrücker and Nijssen, 1984 – *Sedis mutabilis*

Ricola Isbrücker and Nijssen, 1978 – *Sedis mutabilis*

Loricaria Linnaeus, 1758

Paraloricaria Isbrücker, 1979

Proloricaria Isbrücker, 2001

Brochiloricaria Isbrücker and Nijssen, 1979
