# Supplementary material for: A combined molecular and morphological phylogeny of the Loricariinae (Siluriformes: Loricariidae), with emphasis on the Harttiini and Farlowellini
Source: PLoS One. 2021 Mar 15;16(3):e0247747. doi: 10.1371/journal.pone.0247747 (PMC7959404; doi:10.1371/journal.pone.0247747)
Supplement: S2 File — Characters in bold represent exclusive molecular synapomorphies. (PDF) [file pone.0247747.s002.pdf]

Supplementary Material 2

List of molecular transformations for clades in Loricariinae based on the Maximum Parsimony analysis. Characters in bold represent exclusive molecular synapomorphies.

Loricariinae

Cytb (mitochondrial):
Char. 2725: G → T
Char. 2746: T → G
Char. 2770: G → C
Char. 2813: A → C
Char. 2824: C → G
Char. 2830: C → G
Char. 2926: C → G
Char. 2932: A → G
Char. 2980: G → T
Char. 3046: G → C
Char. 3064: G → C
Char. 3184: C → G
Char. 3193: T → C
Char. 3202: T → G
Char. 3223: T → G
Char. 3256: T → G
Char. 3379: A → G
Char. 3405: G → T
Char. 3478: T → G
Char. 3613: C → G
Char. 3649: T → G
Char. 3688: G → T
Char. 3715: T → C

MyH6 (nuclear):
Char. 3842: C → A
Char. 4307: A → C
Char. 4416: T → G
Char. 4680: A → G

RAG1 (nuclear):
Char. 4746: T → G
Char. 4780: C → A
Char. 4878: T → G
Char. 4928: C → A
Char. 4944: A → C
Char. 5010: C → A

nd2 (mitochondrial):
Char. 5930: G → C
Char. 5969: C → G
Char. 6074: G → T
Char. 6293: T → G
Char. 6311: C → G
Char. 6327: A → C
Char. 6332: C → T
Char. 6446: G → C
Char. 6479: C → G
Char. 6486: A → C
Char. 6505: T → C
Char. 6524: G → T
Char. 6544: A → G
Char. 6548: G → C
Char. 6560: T → C
Char. 6561: C → G
Char. 6569: C → G
Char. 6796: G → T
Char. 6803: C → G

Harttiini

12S and 16S rRNA (mitochondrial):
Char. 328: C → T
Char. 562: A → C
Char. 683: T → G
Char. 946: G → T
Char. 1281: G → T
Char. 1293: G → T
Char. 1294: G → C
Char. 1309: C → GT
Char. 1455: G → C
Char. 1874: G → C
Char. 1899: G → T
Char. 1968: T → G
Char. 2061: T → G
Char. 2090: C → A
Char. 2096: G → T
Char. 2365: C → A
Char. 2379: G → T
Char. 2582: C → A

Cytb (mitochondrial):
Char. 3148: G → C
Char. 3586: C → G

RAG1 (nuclear):
Char. 4431: G → T
Char. 5019: G → A

RAG2 (nuclear):
Char. 5280: G → T
Char. 5290: G → A
Char. 5832: G → A

nd2 (mitochondrial):
Char. 6077: C → G
Char. 6107: G → C
Char. 6395: G → T
Char. 6467: G → T

Harttiella

12S and 16S rRNA (mitochondrial):
Char. 197: A → C
Char. 234: T → G
Char. 238: T → G
Char. 265: A → C
Char. 281: G → T
Char. 298: G → T
Char. 299: A → C
Char. 327: G → C
Char. 330: G → T
Char. 380: C → A
Char. 384: C → A
Char. 386: G → T
Char. 433: G → T
Char. 440: C → A
Char. 516: C → T
Char. 549: G → T
Char. 557: G → C
Char. 560: G → T
Char. 585: C → A
Char. 602: T → C
Char. 624: A → C
Char. 657: G → T
Char. 677: C → A
Char. 678: C → T
Char. 686: C → A
Char. 741: T → G
Char. 753: C → G
Char. 814: G → C
Char. 911: A → C
Char. 914: G → T
Char. 1045: A → C
Char. 1073: T → C
Char. 1101: G → T
Char. 1110: T → C

Char. 1155: G → T
Char. 1160: A → C
Char. 1257: A → C
Char. 1268: G → T
Char. 1299: C → G
Char. 1346: T → G
Char. 1352: A → C
Char. 1383: T → C
Char. 1410: A → C
Char. 1415: C → T
Char. 1422: T → C
Char. 1424: C → A
Char. 1425: C → G
Char. 1505: G → T
Char. 1534: T → C
Char. 1599: T → G
Char. 1622: G → C
Char. 1645: G → T
Char. 1703: G → C
Char. 1709: G → C
Char. 1771: T → C
Char. 1818: G → T
Char. 1867: A → C
Char. 1873: G → T
Char. 1885: T → C
Char. 1924: A → C
Char. 1994: C → A
Char. 2016: A → C
Char. 2030: G → C
Char. 2033: T → G
Char. 2054: A → C
Char. 2062: A → C
Char. 2065: T → G
Char. 2077: GT → C
Char. 2089: G → T
Char. 2097: C → G
Char. 2112: G → T
Char. 2126: G → C
Char. 2174: A → C
Char. 2198: G → A
Char. 2223: C → G
Char. 2271: T → G
Char. 2360: C → T
Char. 2401: C → T
Char. 2553: G → C
Char. 2675: T → A
Char. 2683: A → T

RAG1 (nuclear):
Char. 4537: A → G
Char. 4647: G → A
Char. 4678: A → C
Char. 4739: C → G
Char. 4890: G → T
Char. 4935: A → C
Char. 5070: T → G

nd2 (mitochondrial):
Char. 5898: G → T
Char. 5907: A → T
Char. 5909: G → T
Char. 5939: C → A
Char. 5966: T → A
Char. 6023: T → G
Char. 6059: T → G
Char. 6062: C → G
Char. 6070: G → T
Char. 6096: C → A
Char. 6109: T → G
Char. 6113: C → G
Char. 6121: G → T
Char. 6130: T → G
Char. 6136: T → G
Char. 6137: G → T
Char. 6164: C → G
Char. 6170: C → G
Char. 6188: C → A
Char. 6197: T → G
Char. 6206: C → G
Char. 6217: G → T
Char. 6221: C → T
Char. 6286: G → C
Char. 6299: C → G
Char. 6307: G → T
Char. 6311: G → T
Char. 6321: G → T
Char. 6356: G → T
Char. 6375: C → T
Char. 6398: G → T
Char. 6415: T → G
Char. 6419: T → C
Char. 6437: G → T
Char. 6456: G → T
Char. 6473: G → T
Char. 6483: A → C
Char. 6487: G → T
Char. 6537: G → T
Char. 6543: T → G
Char. 6553: G → T
Char. 6582: C → A
Char. 6623: G → T
Char. 6723: G → T
Char. 6728: C → T
Char. 6776: G → T
Char. 6810: C → T
Char. 6811: G → T
Char. 6818: G → T
Char. 6819: G → C

***Ctenilorica*ria**

12S and 16S rRNA (mitochondrial):
Char. 417: T → G
Char. 564: A → C
Char. 627: C → A
Char. 699: C → T
Char. 750: G → T
Char. 857: G → C
Char. 955: T → G
Char. 1079: T→ G
Char. 1183: A → C
Char. 1193: T → G
Char. 1196: T → G
Char. 1241: G → T
Char. 1277: T → G
Char. 1282: G → C
Char. 1294: C → T
Char. 1321: T → C
Char. 1351: T → G
Char. 1609: A → C
Char. 1705: C → A
Char. 1709: G → T
Char. 1710: G → C
Char. 1725: G → T
Char. 1819: T → G
Char. 1869: G → T
Char. 1882: T → G
Char. 1898: T → C
Char. 1904: T → G
Char. 2126: G → T
Char. 2231: T → G
Char. 2459: G → T
Char. 2465: C → T
Char. 2493: G → C
Char. 2527: C → T
Char. 2550: C → T
Char. 2553: G → T
Char. 2563: T → G

Cytb (mitochondrial):
Char. 2726: C → A
Char. 2738: G → T
Char. 2771: A → C
Char. 2800: C → G
Char. 2857: T → G
Char. 2972: G → T
Char. 3010: C → A
Char. 3094: G → C
Char. 3142: T → G
Char. 3199: G → T
Char. 3223: G → T
Char. 3280: G → T
Char. 3283: G → T
Char. 3286: G → T

Char. 3325: C → G
Char. 3343: C → A
Char. 3382: G → T
Char. 3409: G → T
Char. 3434: G → T
Char. 3457: G → T
Char. 3505: G → T
Char. 3514: T → G
Char. 3568: C → A
Char. 3581: T → G
Char. 3595: C → T
Char. 3631: G → T
Char. 3688: T → G
Char. 3706: G → T
Char. 3748: G → T

MyH6 (nuclear):
Char. 3761: C → A
Char. 3806: T → G
Char. 3842: A → C
Char. 3866: T → G
Char. 3890: G → T
Char. 3893: T → G
Char. 3956: C → A
Char. 3965: C → A
Char. 4010: T → G
Char. 4031: C → A
Char. 4043: C → A
Char. 4088: A → C
Char. 4130: C → G
Char. 4169: A → T
Char. 4202: C → T
Char. 4205: C → G
Char. 4217: T → G
Char. 4304: T → A
Char. 4322: G → T
Char. 4334: T → G
Char. 4346: C → A

RAG1 (nuclear):
Char. 4758: C → A
Char. 4845: T → C
Char. 4980: A → T
Char. 5058: T → G

nd2 (mitochondrial):
Char. 6071: T → G
Char. 6200: G → C
Char. 6383: G → T
Char. 6488: C → G
Char. 6514: A → G

Harttia

12S and 16S rRNA (mitochondrial):
Char. 329: C → G
Char. 486: G → T
Char. 1889: G → T
Char. 2468: C → A

Cytb (mitochondrial):
Char. 2798: G → T
Char. 3001: G → C
Char. 3151: G → T
Char. 3361: G → T
Char. 3560: G → T
MyH6 (nuclear):
Char. 3920: T → G
Char. 4028: A → C
Char. 4235: A → C
Char. 4280: T → A

nd2 (mitochondrial):
Char. 5906: T → G
Char. 6089: C → T
Char. 6134: C → T
Char. 6221: C → G
Char. 6293: G → T
Char. 6298: G → T
Char. 6419: T → G
Char. 6459: C → A
Char. 6746: G → T
Char. 6771: C → T

Farlowellini

12S and 16S rRNA (mitochondrial):
Char. 510: C → T
Char. 682: T → G
Char. 709: C → A
Char. 714: G → T
Char. 750: G → T
Char. 1181: C → A
Char. 1196: T → G
Char. 1320: C → G
Char. 1327: C → G
Char. 1697: T → C
Char. 1698: T → G
Char. 1701: T → G
Char. 1813: T → G
Char. 1842: G → T
Char. 2125: G → C
Char. 2478: C → G
Char. 2600: T → G

Cytb (mitochondrial):
Char. 2759: T → A
Char. 3178: G → T
Char. 3407: T → A
Char. 3454: T → G

MyH6 (nuclear):
Char. 3854: G → T
Char. 3953: T → C
Char. 3959: G → T

RAG1 (nuclear):
Char. 4473: G → C
Char. 5061: T → G

RAG2 (nuclear):
Char. 5421: C → G
Char. 5472: G → T
Char. 5761: G → C
Char. 5873: G → A

nd2 (mitochondrial):
Char. 6224: C → G
Char. 6233: C → A
Char. 6269: C → GT
Char. 6392: C → G
Char. 6419: T → G
Char. 6425: T → G
Char. 6459: C → A
Char. 6575: T → G

Lamontichthys

No molecular characters diagnose this genus.

Pterosturisoma

12S and 16S rRNA (mitochondrial):
Char. 217: T → G
Char. 228: C → A
Char. 255: C → A
Char. 286: G → T
Char. 298: G → T
Char. 303: C → A
Char. 320: G → A
Char. 327: T → G
Char. 330: G → T
Char. 362: G → T
Char. 576: C → A
Char. 677: C → T
Char. 955: T → G
Char. 1078: G → T
Char. 1112: T → C

Char. 1277: T → G
Char. 1282: G → T
Char. 1299: C → A
Char. 1518: T → C
Char. 1660: C → A
Char. 1677: T → G
Char. 1680: G → T
Char. 1725: G → T
Char. 1780: C → A
Char. 1876: C → G
Char. 1994: C → A
Char. 2020: G → A
Char. 2087: C → G
Char. 2088: T → G
Char. 2097: C → A
Char. 2099: G → C
Char. 2150: C → A
Char. 2356: G → T
Char. 2440: A → C
Char. 2449: T → G
Char. 2550: C → A

Cytb (mitochondrial):
Char. 2713: C → A
Char. 2716: C → G
Char. 2722: C → A
Char. 2725: T → G
Char. 2726: C → A
Char. 2785: G → T
Char. 2839: C → A
Char. 2848: G → T
Char. 2881: G → T
Char. 2890: G → T
Char. 2965: G → T
Char. 2974: CT → G
Char. 3010: C → A
Char. 3034: G → A
Char. 3046: C → T
Char. 3070: G → T
Char. 3118: G → C
Char. 3133: G → C
Char. 3211: C → A
Char. 3223: G → T
Char. 3226: C → A
Char. 3238: G → T
Char. 3253: G → T
Char. 3271: C → G
Char. 3283: G → T
Char. 3292: C → A
Char. 3298: C → A
Char. 3374: G → T
Char. 3379: G → T
Char. 3394: C → A
Char. 3469: C → G
Char. 3487: G → T
Char. 3526: G → T
Char. 3529: C → A
Char. 3556: C → A
Char. 3560: G → T
Char. 3566: G → T
Char. 3581: T → G
Char. 3586: C → T
Char. 3587: G → T
Char. 3598: G → T
Char. 3640: C → A
Char. 3653: C → G
Char. 3679: G → T
Char. 3684: T → G
Char. 3685: C → T
Char. 3736: T → G
Char. 3751: T → G

RAG1 (nuclear):
Char. 4416: G → T
Char. 4470: T → G
Char. 4471: G → C
Char. 4634: A → T
Char. 4648: G → A
Char. 4755: A → C
Char. 4869: G → T
Char. 4950: G → C
Char. 5044: C → A
Char. 5070: T → C

RAG2 (nuclear):
Char. 5172: G → C
Char. 5212: G → C
Char. 5352: A → C
Char. 5473: T → C
Char. 5484: A → C
Char. 5502: G → T
Char. 5598: A → C
Char. 5623: A → T
Char. 5694: G → T
Char. 5763: C → A
Char. 5813: A → C
Char. 5883: A → C

nd2 (mitochondrial):
Char. 5933: C → A
Char. 5936: C → A
Char. 5996: C → A
Char. 6020: T → G
Char. 6024: G → T
Char. 6110: C → A
Char. 6131: T → G
Char. 6149: C → A
Char. 6152: G → C
Char. 6188: C → A
Char. 6218: G → C
Char. 6239: C → G
Char. 6257: C → G
Char. 6266: C → A
Char. 6308: G → T
Char. 6344: C → A
Char. 6350: C → A
Char. 6365: C → A
Char. 6368: C → A
Char. 6374: C → A
Char. 6417: C → A
Char. 6458: C → A
Char. 6476: C → A
Char. 6536: C → A
Char. 6573: A → C
Char. 6635: C → A
Char. 6641: T → G
Char. 6644: C → A
Char. 6677: G → T
Char. 6731: C → A
Char. 6746: G → T
Char. 6796: T → G

Sturisoma

12S and 16S rRNA (mitochondrial):
Char. 256: G → T
Char. 330: G → C
Char. 591: G → T
Char. 1106: T → G
Char. 1109: G → C
Char. 1267: C → G
Char. 1313: G → C
Char. 1321: T → G
Char. 1558: C → A
Char. 1633: T → A
Char. 1782: G → T
Char. 1848: G → T
Char. 2020: G → C
Char. 2249: G → T
Char. 2563: T → G

Cytb (mitochondrial):
Char. 2962: G → T
Char. 3034: G → T
Char. 3038: C → A
Char. 3073: G → C
Char. 3169: T → G
Char. 3178: T → G
Char. 3394: C → T
Char. 3398: G → T
Char. 3433: G → T
Char. 3457: G → T
Char. 3508: G → T
Char. 3724: C → G

MyH6 (nuclear):
Char. 4169: A → T
Char. 4358: G → T
Char. 4385: T → A

RAG2 (nuclear):
Char. 5652: A → C

nd2 (mitochondrial):
Char. 5987: G → T
Char. 6125: G → T
Char. 6161: C → A
Char. 6230: T → G
Char. 6239: C → T
Char. 6251: T → G
Char. 6300: G → T
Char. 6310: G → T
Char. 6314: C → A
Char. 6351: G → T
Char. 6353: C → A
Char. 6407: G → T
Char. 6531: G → T
Char. 6554: G → T
Char. 6557: G → C
Char. 6575: G → T
Char. 6578: G → C

Sturisomatichthys

12S and 16S rRNA (mitochondrial):
Char. 288: G → T
Char. 326: G → T
Char. 327: T → C
Char. 516: C → A
Char. 604: C → A
Char. 1978: G → T

Cytb (mitochondrial):
Char. 2713: C → A
Char. 2785: G → T
Char. 2794: G → C
Char. 3304: G → T
Char. 3602: G → T
Char. 3712: T → G

RAG2 (nuclear):
Char. 5352: A → C
Char. 5613: G → T

nd2 (mitochondrial):
Char. 6098: G → T
Char. 6170: C → G
Char. 6197: G → T
Char. 6298: G → T
Char. 6815: CA → T

Farlowella

12S and 16S rRNA (mitochondrial):
Char. 185: G → C
Char. 509: C → A
Char. 1898: G → T

Cytb (mitochondrial):
Char. 2803: C → A
Char. 2806: T → G
Char. 3160: G → T
Char. 3385: G → T
Char. 3403: C → G
Char. 3421: G → T

Loricariini

12S and 16S rRNA (mitochondrial):
Char. 234: T → G
Char. 328: C → G
Char. 966: A → C
Char. 983: G → T
Char. 2065: T → G

Cytb (mitochondrial):
Char. 3587: G → T

RAG1 (nuclear):
Char. 5019: G → C

nd2 (mitochondrial):
Char. 5906: T → G
Char. 6221: C → G
