# Supplementary material for: A combined molecular and morphological phylogeny of the Loricariinae (Siluriformes: Loricariidae), with emphasis on the Harttiini and Farlowellini
Source: PLoS One. 2021 Mar 15;16(3):e0247747. doi: 10.1371/journal.pone.0247747 (PMC7959404; doi:10.1371/journal.pone.0247747)
Supplement: S3 File — Missing taxa in the partition are represented by DNA data only. (PDF) [file pone.0247747.s003.pdf]

Supplementary Material 3

**Phenotypic partition of the data matrix (osteology and external morphology).** Missing taxa in this partition are represented by DNA data only.

| **Taxon/Character** | **1** | **2** | **3** | **4** | **5** | **6** | **7** | **8** | **9** | **10** | **11** | **12** | **13** | **14** | **15** | **16** | **17** | **18** | **19** | **20** | **21** | **22** | **23** | **24** | **25** | **26** |
|---|---|---|---|---|---|---|---|---|---|---|---|---|---|---|---|---|---|---|---|---|---|---|---|---|---|---|
| ***Hemipsilichthys gobio*** | 0 | 0 | 0 | 0 | 0 | 0 | 0 | 0 | 0 | 0 | 0 | 0 | 0 | 0 | 0 | 0 | 0 | 0 | 0 | 0 | 0 | 0 | 0 | 0 | 0 | 0 |
| ***Acestridium scutatum*** | 1 | 0 | 0 | 0 | 0 | 2 | 0 | 0 | 0 | 0 | 0 | ? | 3 | 0 | 0 | 0 | 0 | 0 | 0 | 1 | 0 | 0 | 0 | 0 | ? | 0 |
| ***Ancistrus brevipinnis*** | 0 | 0 | 0 | 0 | 0 | 0 | 1 | 1 | 0 | 1 | 0 | 1 | 0 | 0 | 1 | 1 | 1 | 1 | 2 | 0 | 1 | 2 | 0 | 0 | 1 | 0 |
| ***Chaetostoma breve*** | 0 | 0 | ? | 0 | 0 | 1 | 0 | 2 | 0 | 1 | 0 | 1 | 1 | 0 | 0 | 1 | 1 | 1 | 4 | 0 | 2 | - | 0 | 1 | 1 | 1 |
| ***Pseudohemiodon lamina*** | 1 | 1 | 1 | 0 | 3 | 1 | 3 | 0 | 1 | 1 | 2 | 0 | 4 | 1 | 0 | 1 | 1 | 1 | 4 | 2 | 2 | - | 1 | 0 | 0 | 1 |
| ***Dasyloricaria filamentosa*** | 0 | 0 | 0 | 0 | 3 | 0 | 3 | 3 | 1 | 1 | 1 | 0 | 1 | 1 | 0 | 0 | 0 | 1 | 4 | 2 | 1 | 2 | 1 | 1 | 0 | 1 |
| ***Dasyloricaria latiura*** | 1 | 0 | 0 | 0 | 3 | 0 | 3 | 3 | 1 | 1 | 1 | 0 | 1 | 0 | 0 | 0 | 0 | 1 | 4 | 2 | 2 | - | 1 | 0 | 0 | 1 |
| ***Dasyloricaria paucisquama*** | 0 | 0 | 0 | 0 | 3 | 0 | 3 | 3 | 1 | 1 | 1 | 0 | 2 | 0 | 0 | 1 | 1 | 1 | 4 | 2 | 2 | - | 1 | 0 | 0 | 1 |
| ***Hemiodontichthys acipenserinus*** | 1 | 0 | 0 | 0 | 3 | 2 | 2 | 2 | 1 | 0 | 1 | 0 | 4 | 0 | 0 | 1 | 1 | 1 | 4 | 1 | 1 | 1 | 1 | 0 | 0 | 0 |
| ***Hisonotus laevior*** | 0 | 0 | 1 | 0 | 0 | 1 | 0 | 0 | 0 | 1 | 0 | 0 | 3 | 0 | 0 | 0 | 0 | 0 | 1 | 1 | 0 | 0 | 0 | 0 | 0 | 0 |
| ***Limatulichthys griseus*** | 0 | 1 | 1 | 0 | 3 | 1 | 2 | 0 | 1 | 1 | 1 | 0 | 4 | 1 | 0 | 1 | 1 | 1 | 4 | 1 | 1 | 2 | 1 | 1 | 1 | 1 |
| ***Loricaria lundbergi*** | 0 | 0 | 0 | 0 | 3 | 1 | 3 | 0 | 1 | 1 | 1 | 0 | 4 | 0 | 0 | 0 | 0 | 0 | 4 | 2 | 1 | 1 | 1 | 1 | 0 | 1 |
| ***Loricariichthys anus*** | 0 | 0 | 0 | 0 | 3 | 0 | 3 | 3 | 1 | 1 | 1 | 0 | 4 | 1 | 1 | 1 | 1 | 1 | 4 | 2 | 1 | 1 | 0 | 0 | 1 | 2 |
| ***Loricariichthys platymetopon*** | 0 | 0 | ? | 0 | 3 | 1 | 3 | 3 | 1 | 1 | 1 | 0 | 2 | 0 | 1 | 1 | 1 | 1 | 4 | 2 | 1 | 1 | 1 | 0 | 1 | 2 |
| ***Neoplecostomus microps*** | 0 | 0 | 0 | 0 | 0 | 0 | 0 | 0 | 0 | 0 | 0 | 0 | 0 | 0 | 0 | 0 | 0 | 1 | 2 | 1 | 0 | 0 | 0 | 0 | 0 | 1 |
| ***Pareiorhaphis calmoni*** | 0 | 0 | 0 | 0 | 0 | 1 | 0 | 0 | 0 | 0 | 0 | 0 | 0 | 0 | 0 | 1 | 1 | 1 | 3 | 0 | 1 | 1 | 0 | 0 | 1 | 0 |
| ***Parotocinclus maculicauda*** | 0 | 0 | 1 | 0 | 0 | 1 | 1 | 0 | 0 | 0 | 0 | 0 | 3 | 0 | 0 | 0 | 0 | 0 | 2 | 1 | 1 | 1 | 0 | 0 | 0 | 0 |
| ***Pterygoplichthys lituratus*** | 1 | 0 | 0 | 0 | 2 | 0 | 3 | 1 | 0 | 1 | 1 | 1 | 0 | 0 | 0 | 1 | 1 | 1 | 0 | 0 | 1 | 1 | 0 | 0 | 1 | 0 |
| ***Rineloricaria cadeae*** | 0 | 0 | 0 | 0 | 3 | 1 | 3 | 2 | 1 | 0 | 2 | 0 | 4 | 0 | 0 | 1 | 1 | 0 | 4 | 2 | 1 | 1 | 1 | 0 | 0 | 0 |
| ***Rineloricaria lanceolata*** | 0 | 0 | 1 | 0 | 3 | 1 | 3 | 2 | 1 | 0 | 1 | 0 | 4 | 0 | 0 | 1 | 1 | 1 | 4 | 2 | 1 | 1 | 1 | 1 | 0 | 1 |

| **Taxon/Character** | **27** | **28** | **29** | **30** | **31** | **32** | **33** | **34** | **35** | **36** | **37** | **38** | **39** | **40** | **41** | **42** | **43** | **44** | **45** | **46** | **47** | **48** | **49** | **50** |
|---|---|---|---|---|---|---|---|---|---|---|---|---|---|---|---|---|---|---|---|---|---|---|---|---|
| ***Hemipsilichthys gobio*** | 0 | 0 | 0 | 0 | 0 | 0 | 0 | 0 | 0 | 0 | 0 | 0 | 0 | 0 | 0 | 0 | 0 | 0 | 0 | 0 | 0 | 0 | 0 | 0 |
| ***Acestridium scutatum*** | 0 | 0 | ? | 0 | ? | 1 | 0 | 0 | 0 | 0 | 0 | 0 | 0 | 0 | 1 | 0 | 0 | 0 | 0 | 0 | 0 | 0 | 0 | 0 |
| ***Ancistrus brevipinnis*** | 0 | 0 | 0 | 1 | 0 | 0 | 1 | 1 | 0 | 0 | 0 | 2 | 2 | 0 | 1 | 2 | 0 | 0 | 0 | 0 | 1 | 0 | 1 | 0 |
| ***Chaetostoma breve*** | 1 | 0 | ? | 1 | ? | ? | 0 | 1 | 0 | 0 | 0 | 2 | 0 | 1 | 2 | 2 | 0 | 0 | 1 | 0 | 1 | 0 | 2 | 0 |
| ***Pseudohemiodon lamina*** | 1 | 0 | 1 | 1 | 0 | 0 | 4 | 1 | 0 | 0 | 0 | 4 | 1 | 1 | 2 | 0 | 0 | 1 | 0 | 2 | 3 | 1 | 2 | 1 |
| ***Dasyloricaria filamentosa*** | 1 | 0 | 1 | 1 | 1 | 2 | 0 | 1 | 0 | 0 | 0 | 3 | 1 | 2 | 2 | 2 | 0 | 1 | 0 | 2 | 2 | 1 | 2 | 1 |
| ***Dasyloricaria latiura*** | 1 | 0 | 0 | 1 | 1 | 0 | 0 | 1 | 0 | 0 | 0 | 4 | 1 | 2 | 2 | 1 | 0 | 1 | 0 | 2 | 2 | 1 | 2 | 1 |
| ***Dasyloricaria paucisquama*** | 1 | 0 | 0 | 1 | 1 | 0 | 0 | 1 | 0 | 0 | 0 | 4 | 1 | 2 | 2 | 1 | 0 | 1 | 0 | 2 | 2 | 1 | 2 | 1 |
| ***Hemiodontichthys acipenserinus*** | 0 | 0 | ? | 1 | 1 | 0 | 0 | 1 | 0 | 0 | 0 | 1 | 1 | 1 | 0 | 1 | 0 | 1 | 0 | - | 3 | 1 | 2 | - |
| ***Hisonotus laevior*** | 0 | 0 | 0 | 0 | 1 | 0 | 0 | 1 | 1 | 0 | 0 | 0 | 0 | 0 | 0 | 0 | 0 | 0 | 1 | 0 | 1 | 0 | 0 | 0 |
| ***Limatulichthys griseus*** | 0 | 0 | 1 | 1 | 1 | 0 | 0 | 1 | 0 | 0 | 0 | 4 | 0 | 2 | 2 | 1 | 0 | 1 | 0 | 2 | 3 | 1 | 2 | 1 |
| ***Loricaria lundbergi*** | 1 | 0 | 1 | 1 | 1 | 0 | 0 | 1 | 0 | 0 | 0 | 4 | 1 | 2 | 0 | 1 | 0 | 1 | 0 | 2 | 2 | 1 | 2 | 1 |
| ***Loricariichthys anus*** | 1 | 0 | 1 | 1 | 1 | 0 | 1 | 1 | 0 | 0 | 0 | 4 | 0 | 2 | 1 | 1 | 0 | 1 | 0 | - | 3 | 1 | 2 | 1 |
| ***Loricariichthys platymetopon*** | 1 | 0 | 1 | 1 | 1 | 0 | 1 | 1 | 0 | 0 | 0 | 4 | 1 | 2 | 1 | 1 | 0 | 1 | 0 | - | 3 | 1 | 2 | 1 |
| ***Neoplecostomus microps*** | 0 | 0 | 0 | 0 | 1 | 0 | 0 | 0 | 1 | 0 | 0 | 0 | 0 | 1 | 1 | 0 | 0 | 0 | 0 | 0 | 1 | 1 | 2 | 0 |
| ***Pareiorhaphis calmoni*** | 1 | 0 | 0 | 0 | 0 | 0 | 0 | 0 | 0 | 0 | 0 | 0 | 0 | 0 | 1 | 0 | 0 | 0 | 1 | 0 | 0 | 0 | 0 | 0 |
| ***Parotocinclus maculicauda*** | 0 | 0 | 0 | 1 | 0 | 0 | 1 | 1 | 1 | 0 | 0 | 0 | 0 | 0 | 0 | 0 | 0 | 0 | 1 | 0 | 1 | 0 | 0 | 0 |
| ***Pterygoplichthys lituratus*** | 0 | 0 | 0 | 0 | 0 | ? | 2 | 1 | 0 | 0 | 0 | 4 | 2 | 0 | 2 | 2 | 0 | 1 | 0 | 0 | 1 | 1 | 2 | 0 |
| ***Rineloricaria cadeae*** | 1 | 0 | ? | 1 | 1 | 0 | 1 | 1 | 0 | 0 | 0 | 3 | 1 | 1 | 2 | 1 | 0 | 1 | 0 | 2 | 3 | 1 | 2 | 1 |
| ***Rineloricaria lanceolata*** | 1 | 0 | 1 | 1 | 1 | 0 | 1 | 1 | 0 | 0 | 1 | 3 | 1 | 1 | 1 | 1 | 0 | 1 | 0 | 2 | 3 | 1 | 2 | 1 |

| **Taxon/Character** | **51** | **52** | **53** | **54** | **55** | **56** | **57** | **58** | **59** | **60** | **61** | **62** | **63** | **64** | **65** | **66** | **67** | **68** | **69** | **70** | **71** | **72** | **73** | **74** |
|---|---|---|---|---|---|---|---|---|---|---|---|---|---|---|---|---|---|---|---|---|---|---|---|---|
| ***Hemipsilichthys gobio*** | 0 | 0 | 0 | 0 | 0 | 0 | 0 | 0 | 0 | 0 | 0 | 0 | 0 | 0 | 0 | 0 | 0 | 0 | 0 | 0 | 0 | 0 | 0 | 0 |
| ***Acestridium scutatum*** | 0 | 1 | 0 | 1 | 0 | 1 | 0 | 0 | 2 | 2 | 1 | 1 | 0 | 1 | 0 | 1 | 1 | 0 | 0 | ? | 0 | ? | 2 | 0 |
| ***Ancistrus brevipinnis*** | 0 | 2 | 1 | 0 | 0 | 0 | 0 | 0 | 0 | 0 | 0 | 2 | 1 | 1 | 1 | 1 | 3 | 1 | 0 | 0 | 1 | 1 | 2 | 2 |
| ***Chaetostoma breve*** | 0 | 3 | 1 | 0 | 0 | 1 | 0 | 0 | 1 | 0 | 0 | 1 | 1 | 1 | 1 | 0 | 3 | 1 | 0 | ? | 1 | ? | 2 | 2 |
| ***Pseudohemiodon lamina*** | 1 | 1 | 2 | 1 | 0 | 0 | 1 | 1 | 2 | 2 | 1 | 1 | 1 | 0 | 1 | 1 | 3 | 1 | 0 | 1 | 1 | 1 | 2 | 2 |
| ***Dasyloricaria filamentosa*** | 2 | 2 | 2 | 1 | 0 | 1 | 1 | 1 | 2 | 2 | 1 | 0 | 0 | 0 | 1 | 1 | 1 | 0 | 0 | 1 | 1 | 0 | 2 | 3 |
| ***Dasyloricaria latiura*** | 2 | 2 | 2 | 1 | 0 | 1 | 1 | 1 | 2 | 2 | 1 | 0 | 0 | 0 | 1 | 1 | 1 | 0 | 0 | 1 | 1 | 0 | 2 | 3 |
| ***Dasyloricaria paucisquama*** | 2 | 2 | 2 | 1 | 0 | 1 | 1 | 1 | 2 | 2 | 1 | 0 | 1 | 0 | 1 | 1 | 1 | 0 | 0 | 1 | 1 | 0 | 2 | 3 |
| ***Hemiodontichthys acipenserinus*** | - | ? | 2 | - | ? | - | 2 | ? | 2 | 2 | 1 | 0 | 0 | 0 | 1 | 1 | 3 | 1 | 0 | 0 | 1 | 0 | 2 | 3 |
| ***Hisonotus laevior*** | 0 | 1 | 0 | 1 | 0 | 1 | 0 | 0 | 2 | 1 | 1 | 0 | 1 | 1 | 1 | 1 | 1 | 1 | 0 | 0 | 1 | 1 | 0 | 0 |
| ***Limatulichthys griseus*** | 2 | 2 | 2 | 1 | 0 | 0 | 1 | 1 | 2 | 2 | 1 | 0 | 1 | 0 | 1 | 1 | 2 | 0 | 1 | 0 | 1 | 0 | 2 | 3 |
| ***Loricaria lundbergi*** | 2 | 2 | 2 | 1 | 0 | 0 | 2 | 1 | 2 | 2 | 1 | 0 | 0 | 0 | 1 | 1 | 1 | 0 | 1 | 1 | 0 | 0 | 2 | 3 |
| ***Loricariichthys anus*** | 2 | ? | 2 | - | ? | 1 | 1 | 1 | 2 | 1 | 1 | 0 | 0 | 0 | 0 | 1 | 1 | 1 | 0 | 1 | 1 | 0 | 2 | 3 |
| ***Loricariichthys platymetopon*** | 2 | ? | 2 | - | ? | 1 | 1 | 1 | 2 | 1 | 1 | 0 | 0 | 0 | 0 | 1 | 1 | 1 | 0 | 1 | 1 | 0 | 2 | 3 |
| ***Neoplecostomus microps*** | 0 | 2 | 0 | 0 | 0 | 0 | 0 | 0 | 2 | 2 | 1 | 2 | 1 | 1 | 0 | 1 | 1 | 1 | 0 | 0 | 1 | 0 | 0 | 0 |
| ***Pareiorhaphis calmoni*** | 0 | 2 | 0 | 0 | 0 | 0 | 0 | 0 | 0 | 0 | 0 | 2 | 1 | 1 | 1 | 0 | 1 | 0 | 0 | 0 | 0 | 0 | 0 | 0 |
| ***Parotocinclus maculicauda*** | 0 | 1 | 0 | 1 | 0 | 1 | 0 | 0 | 2 | 1 | 1 | 0 | 1 | 1 | 0 | 1 | 1 | 1 | 0 | 0 | 0 | 1 | 0 | 0 |
| ***Pterygoplichthys lituratus*** | 2 | 2 | 1 | 0 | 0 | 1 | 0 | 0 | 2 | 2 | 1 | 0 | 1 | 0 | 1 | 1 | 1 | 0 | 0 | 0 | 1 | 1 | 2 | 3 |
| ***Rineloricaria cadeae*** | 2 | 1 | 2 | 1 | 0 | 0 | 0 | 1 | 2 | 2 | 1 | 0 | 0 | 0 | 1 | 1 | 1 | 1 | 0 | 1 | 1 | 0 | 2 | 3 |
| ***Rineloricaria lanceolata*** | 2 | 2 | 2 | 1 | 0 | 0 | 0 | 1 | 2 | 2 | 1 | 2 | 1 | 0 | 1 | 1 | 1 | 1 | 1 | 1 | 1 | 0 | 2 | 3 |

| Taxon/Character | 75 | 76 | 77 | 78 | 79 | 80 | 81 | 82 | 83 | 84 | 85 | 86 | 87 | 88 | 89 | 90 | 91 | 92 | 93 | 94 | 95 | 96 | 97 | 98 | 99 |
|---|---|---|---|---|---|---|---|---|---|---|---|---|---|---|---|---|---|---|---|---|---|---|---|---|---|
| *Hemipsilichthys gobio* | 0 | 0 | 0 | 0 | 0 | 0 | 0 | 0 | 0 | 0 | 0 | 0 | 0 | 0 | 0 | 0 | 0 | 0 | 0 | 0 | 0 | 0 | 0 | 0 | 0 |
| *Acestridium scutatum* | 0 | 0 | 1 | 0 | 1 | 0 | 1 | 3 | 0 | 1 | 0 | 0 | 1 | 0 | 0 | 0 | 2 | 2 | - | 0 | 0 | 0 | 0 | 0 | 2 |
| *Ancistrus brevipinnis* | 1 | 0 | 2 | 0 | 0 | 1 | 0 | 2 | 0 | 0 | 0 | 0 | 1 | 2 | 0 | 0 | 0 | 1 | 1 | 0 | 1 | 0 | 0 | 0 | 2 |
| *Chaetostoma breve* | 1 | 0 | 2 | ? | 0 | 1 | 0 | 3 | 2 | 1 | 0 | 0 | 1 | ? | ? | ? | 0 | 1 | 1 | 0 | 1 | 0 | 0 | 0 | 2 |
| *Pseudohemiodon lamina* | 1 | 0 | 2 | 2 | 0 | 1 | 0 | 2 | 2 | 1 | 0 | 3 | 1 | 2 | 1 | 0 | 0 | 2 | - | 2 | 3 | 2 | 2 | 0 | 1 |
| *Dasyloricaria filamentosa* | 2 | 0 | 1 | 2 | 1 | 1 | 0 | 2 | 1 | 1 | 0 | 3 | 0 | 2 | 1 | 0 | 0 | 2 | - | 0 | 0 | 1 | 2 | 0 | 1 |
| *Dasyloricaria latiura* | 2 | 0 | 2 | 2 | 1 | 1 | 0 | 2 | 0 | 1 | 0 | 3 | 0 | 2 | 1 | 1 | 0 | 2 | - | 0 | 0 | 1 | 1 | 0 | 1 |
| *Dasyloricaria paucisquama* | 2 | 0 | 1 | 2 | 1 | 1 | 0 | 2 | 1 | 1 | 0 | 3 | 0 | 2 | 1 | 1 | 0 | 2 | - | 0 | 0 | 1 | 1 | 0 | 1 |
| *Hemiodontichthys acipenserinus* | 2 | 0 | 1 | 2 | 1 | 1 | 0 | 3 | 2 | 1 | 0 | 3 | 0 | 2 | 0 | 0 | 0 | 2 | - | 1 | 2 | 1 | 1 | 0 | 0 |
| *Hisonotus laevior* | 0 | 0 | 1 | 0 | 0 | 0 | 0 | 1 | 0 | 0 | 0 | 2 | 1 | 2 | 0 | 0 | 0 | 0 | 0 | 0 | 0 | 0 | 0 | 0 | 0 |
| *Limatulichthys griseus* | 2 | 0 | 1 | 2 | 1 | 1 | 0 | 2 | 2 | 1 | 0 | 3 | 1 | 1 | 1 | 0 | 0 | 2 | - | 0 | 0 | 1 | 1 | 0 | 0 |
| *Loricaria lundbergi* | 2 | 0 | 2 | 2 | 1 | 0 | 0 | 2 | 1 | 1 | 0 | 3 | 1 | 1 | 1 | 1 | 0 | 2 | - | 0 | 3 | 2 | 2 | 0 | 1 |
| *Loricariichthys anus* | 2 | 0 | 1 | 2 | 1 | 1 | 0 | 2 | 2 | 0 | 0 | 3 | 1 | 1 | 1 | 0 | 0 | 2 | - | 0 | 0 | 1 | 2 | 0 | 1 |
| *Loricariichthys platymetopon* | 2 | 0 | 1 | 2 | 1 | 1 | 0 | 2 | 2 | 0 | 0 | 3 | 1 | 1 | 1 | 0 | 0 | 2 | - | 0 | 0 | 1 | 2 | 0 | 1 |
| *Neoplecostomus microps* | 0 | 0 | 0 | 0 | 0 | 0 | 0 | 0 | 0 | 0 | 0 | 0 | 0 | 0 | 0 | 0 | 0 | 0 | 0 | 0 | 0 | 0 | 2 | 0 | 2 |
| *Pareiorhaphis calmoni* | 0 | 0 | 0 | 0 | 0 | 0 | 0 | 0 | 0 | 0 | 0 | 0 | 0 | 0 | 0 | 0 | 1 | 0 | 0 | 0 | 0 | 0 | 0 | 0 | 1 |
| *Parotocinclus maculicauda* | 0 | 0 | 0 | 0 | 0 | 0 | 0 | 1 | 0 | 0 | 0 | 0 | 0 | 0 | 0 | 0 | 0 | 0 | 0 | 0 | 0 | 0 | 0 | 0 | 1 |
| *Pterygoplichthys lituratus* | 2 | 0 | 2 | 2 | 0 | 1 | 0 | 3 | 2 | 1 | 0 | 0 | 1 | 2 | 0 | 0 | 2 | 2 | - | 1 | 1 | 0 | 0 | 0 | 1 |
| *Rineloricaria cadeae* | 2 | 0 | 1 | ? | 1 | 1 | 0 | 2 | 0 | 1 | 0 | 3 | 0 | 2 | 1 | 0 | 0 | 2 | - | 0 | 0 | 1 | 1 | 0 | 1 |
| *Rineloricaria lanceolata* | 2 | 0 | 1 | 2 | 1 | 1 | 0 | 3 | 0 | 0 | 0 | 3 | 0 | 2 | 1 | 1 | 0 | 2 | - | 0 | 0 | 1 | 2 | 0 | 1 |

| Taxon/Character | 100 | 101 | 102 | 103 | 104 | 105 | 106 | 107 | 108 | 109 | 110 | 111 | 112 | 113 | 114 | 115 | 116 | 117 | 118 | 119 | 120 | 121 | 122 | 123 |
|---|---|---|---|---|---|---|---|---|---|---|---|---|---|---|---|---|---|---|---|---|---|---|---|---|
| ***Hemipsilichthys gobio*** | 0 | 0 | 0 | 0 | 0 | 0 | 0 | 0 | 0 | 0 | 0 | 0 | 0 | 0 | 0 | 0 | 0 | 0 | 0 | 0 | 1 | 0 | 0 | 0 |
| ***Acestridium scutatum*** | 0 | 0 | 0 | 2 | 0 | 2 | 0 | 0 | 1 | 0 | 0 | 0 | 0 | 0 | 0 | 0 | 0 | 0 | 0 | 0 | 2 | 0 | 0 | 0 |
| ***Ancistrus brevipinnis*** | 0 | 0 | 0 | 1 | 2 | 1 | 0 | 2 | 1 | ? | 0 | 0 | 0 | 0 | 0 | 0 | 0 | 0 | 0 | 0 | 0 | 0 | 0 | 1 |
| ***Chaetostoma breve*** | 0 | 0 | 0 | 1 | 2 | 1 | 0 | 2 | 1 | 0 | 0 | 0 | 0 | 0 | 0 | 0 | 0 | 0 | 0 | 0 | 0 | 0 | 0 | 1 |
| ***Pseudohemiodon lamina*** | 1 | - | 0 | 2 | 2 | 3 | 2 | 0 | 0 | ? | 0 | 0 | 0 | 0 | 2 | 0 | 0 | 0 | 2 | 0 | 2 | 0 | 0 | 0 |
| ***Dasyloricaria filamentosa*** | 0 | 2 | 0 | 3 | 3 | 3 | 1 | 0 | 0 | 0 | 0 | 0 | 0 | 2 | 2 | 0 | 0 | 0 | 2 | 0 | 2 | 0 | 0 | 0 |
| ***Dasyloricaria latiura*** | 0 | 2 | 0 | 3 | 3 | 3 | 1 | 0 | 0 | 0 | 0 | 0 | 0 | 2 | 2 | 0 | 0 | 0 | 2 | 0 | 2 | 0 | 0 | 0 |
| ***Dasyloricaria paucisquama*** | 0 | 2 | 0 | 3 | 3 | 3 | 1 | 0 | 0 | 0 | 0 | 0 | 0 | 2 | 2 | 0 | 0 | 0 | 2 | 0 | 2 | 0 | 0 | 0 |
| ***Hemiodontichthys acipenserinus*** | 0 | 0 | 0 | 0 | 2 | 2 | 2 | 0 | 0 | 0 | 0 | 0 | 0 | 0 | 2 | 0 | 0 | 0 | 2 | 0 | 2 | 0 | 0 | 0 |
| ***Hisonotus laevior*** | 0 | 0 | 0 | 2 | 0 | 0 | 0 | 2 | 1 | 0 | 0 | 0 | 0 | 0 | 0 | 0 | 0 | 0 | 0 | 0 | 1 | 0 | 0 | 0 |
| ***Limatulichthys griseus*** | 0 | 2 | 0 | 0 | 2 | 2 | 2 | 0 | 0 | 0 | 0 | 0 | 0 | 2 | 2 | 0 | 1 | 0 | 0 | 0 | 2 | 2 | 0 | 0 |
| ***Loricaria lundbergi*** | 1 | - | 0 | 3 | 3 | 3 | 2 | 0 | 0 | ? | 0 | 0 | 0 | 2 | 2 | 0 | 0 | 0 | 2 | 0 | 2 | 0 | 0 | 0 |
| ***Loricariichthys anus*** | 0 | 2 | 0 | 3 | 2 | 3 | 1 | 0 | 0 | 0 | 0 | 0 | 0 | 1 | 2 | 0 | 0 | 0 | 2 | 0 | 2 | 0 | 3 | 0 |
| ***Loricariichthys platymetopon*** | 0 | 2 | 0 | 3 | 2 | 3 | 1 | 0 | 0 | 0 | 0 | 0 | 0 | 1 | 2 | 0 | 0 | 0 | 2 | 0 | 2 | 0 | 3 | 0 |
| ***Neoplecostomus microps*** | 0 | 0 | 0 | 2 | 1 | 0 | 0 | 0 | 1 | 0 | 0 | 0 | 0 | 1 | 0 | 0 | 0 | 0 | 0 | 0 | 1 | 0 | 0 | 0 |
| ***Pareiorhaphis calmoni*** | 0 | 0 | 0 | 2 | 1 | 0 | 0 | 2 | 1 | 0 | 0 | 0 | 0 | 1 | 0 | 0 | 0 | 0 | 0 | 0 | 1 | 0 | 0 | 0 |
| ***Parotocinclus maculicauda*** | 0 | 0 | 0 | 2 | 0 | 0 | 0 | 2 | 1 | 0 | 0 | 0 | 0 | 0 | 0 | 0 | 0 | 0 | 0 | 0 | 0 | 0 | 0 | 1 |
| ***Pterygoplichthys lituratus*** | 0 | 0 | 0 | 1 | 2 | 1 | 0 | 2 | 1 | 0 | 0 | 0 | 0 | 0 | 0 | 0 | 0 | 0 | 0 | 0 | 0 | 1 | 0 | 0 |
| ***Rineloricaria cadeae*** | 1 | - | 0 | 3 | 3 | 3 | 1 | 0 | 0 | 0 | 0 | 0 | 0 | 2 | 2 | 1 | 0 | 0 | 1 | 0 | 1 | 0 | 0 | 1 |
| ***Rineloricaria lanceolata*** | 1 | - | 0 | 3 | 3 | 3 | 2 | 0 | 0 | 0 | 0 | 0 | 0 | 3 | 2 | 0 | 0 | 0 | 1 | 0 | 1 | 1 | 0 | 1 |

| Taxon/Character | 124 | 125 | 126 | 127 | 128 | 129 | 130 | 131 | 132 | 133 | 134 | 135 | 136 | 137 | 138 | 139 | 140 | 141 | 142 | 143 | 144 | 145 | 146 | 147 |
|---|---|---|---|---|---|---|---|---|---|---|---|---|---|---|---|---|---|---|---|---|---|---|---|---|
| ***Hemipsilichthys gobio*** | 0 | 0 | 0 | 0 | 0 | 0 | 0 | 0 | 0 | 0 | 0 | 0 | 0 | 0 | 0 | 0 | 0 | 0 | 0 | 0 | 0 | 0 | 0 | 0 |
| ***Acestridium scutatum*** | 1 | 0 | 0 | 0 | 0 | 0 | 0 | 0 | 0 | 0 | 0 | 0 | 1 | 0 | 0 | 0 | 1 | 1 | 0 | 2 | 1 | 1 | 1 | 0 |
| ***Ancistrus brevipinnis*** | 0 | 0 | 0 | 0 | 1 | 0 | 0 | 0 | 0 | 1 | 1 | 0 | 0 | 0 | 2 | 1 | 1 | 0 | 0 | 0 | 0 | 1 | 0 | 0 |
| ***Chaetostoma breve*** | 0 | 0 | 0 | 0 | 1 | 0 | 0 | 0 | 0 | 1 | 0 | 2 | 0 | 0 | 2 | 1 | 1 | 0 | ? | 1 | 0 | 1 | 0 | 0 |
| ***Pseudohemiodon lamina*** | 1 | 3 | 0 | 0 | 3 | 0 | 2 | 1 | 0 | 1 | 1 | 0 | 1 | 0 | 0 | 0 | 1 | 0 | 2 | 2 | 1 | 1 | 0 | 0 |
| ***Dasyloricaria filamentosa*** | 1 | 3 | 0 | 0 | 3 | 0 | 1 | 1 | 0 | 1 | 1 | 0 | 1 | 0 | 0 | 1 | 1 | 0 | 2 | 2 | 1 | 1 | 0 | 0 |
| ***Dasyloricaria latiura*** | 1 | 3 | 0 | 0 | 3 | 0 | 2 | 1 | 0 | 1 | 1 | 0 | 1 | 0 | 0 | 1 | 1 | 0 | 1 | 2 | 1 | 1 | 1 | 0 |
| ***Dasyloricaria paucisquama*** | 1 | 3 | 0 | 0 | 3 | 0 | 1 | 1 | 0 | 1 | 1 | 0 | 1 | 0 | 0 | 1 | 1 | 0 | 2 | 2 | 1 | 1 | 0 | 0 |
| ***Hemiodontichthys acipenserinus*** | 1 | 3 | 0 | 0 | 0 | 0 | 1 | 1 | 0 | 1 | 1 | 0 | 1 | 0 | 0 | 0 | 1 | 0 | ? | 2 | 1 | 1 | 1 | 0 |
| ***Hisonotus laevior*** | 1 | 0 | 0 | 0 | 0 | 0 | 0 | 0 | 0 | 0 | 0 | 0 | 0 | 0 | 0 | 0 | 1 | 1 | 0 | 1 | 0 | 1 | 0 | 0 |
| ***Limatulichthys griseus*** | 1 | 3 | 0 | 0 | 3 | 0 | 2 | 1 | 0 | 1 | 1 | 0 | 1 | 0 | 0 | 1 | 1 | 0 | 1 | 2 | 1 | 1 | 0 | 0 |
| ***Loricaria lundbergi*** | 1 | 3 | 0 | 0 | 3 | 0 | 2 | 1 | 0 | 1 | 1 | 0 | 1 | 0 | 0 | 1 | 1 | 0 | 1 | 2 | 1 | 1 | 0 | 0 |
| ***Loricariichthys anus*** | 1 | 3 | 0 | 0 | 3 | 0 | 2 | 1 | 0 | 1 | 1 | 0 | 1 | 0 | 0 | 1 | 1 | 0 | 0 | 2 | 1 | 1 | 0 | 0 |
| ***Loricariichthys platymetopon*** | 1 | 3 | 0 | 0 | 3 | 0 | 2 | 1 | 0 | 1 | 1 | 0 | 1 | 0 | 0 | 1 | 1 | 0 | 0 | 2 | 1 | 1 | 0 | 0 |
| ***Neoplecostomus microps*** | 0 | 0 | 0 | 0 | 0 | 0 | 0 | 0 | 0 | 0 | 0 | 0 | 0 | 0 | 0 | 0 | 0 | 0 | 0 | 0 | 0 | 1 | 0 | 0 |
| ***Pareiorhaphis calmoni*** | 0 | 0 | 0 | 0 | 0 | 0 | 0 | 0 | 0 | 0 | 0 | 0 | 0 | 0 | 0 | 0 | 0 | 0 | 0 | 1 | 0 | 1 | 0 | 0 |
| ***Parotocinclus maculicauda*** | 1 | 0 | 0 | 0 | 0 | 0 | 0 | 0 | 0 | 0 | 0 | 0 | 0 | 0 | 0 | 0 | 1 | 1 | 0 | 2 | 0 | 1 | 0 | 0 |
| ***Pterygoplichthys lituratus*** | 0 | 0 | 1 | 1 | 0 | 0 | 0 | 0 | 0 | 1 | 1 | 2 | 0 | 0 | 0 | 1 | 1 | 0 | 1 | 0 | 1 | 1 | 1 | 0 |
| ***Rineloricaria cadeae*** | 1 | 3 | 0 | 0 | 3 | 0 | 2 | 1 | 0 | 1 | 1 | 0 | 1 | 0 | 0 | 1 | 1 | 0 | 1 | 0 | 0 | 1 | 1 | 0 |
| ***Rineloricaria lanceolata*** | 1 | 3 | 0 | 0 | 3 | 0 | 2 | 1 | 0 | 1 | 1 | 0 | 1 | 0 | 0 | 1 | 1 | 0 | 0 | 1 | 0 | 1 | 0 | 0 |

| Taxon/Character | 148 | 149 | 150 | 151 | 152 | 153 | 154 | 155 | 156 | 157 | 158 | 159 | 160 | 161 | 162 | 163 | 164 | 165 | 166 | 167 | 168 | 169 | 170 | 171 |
|---|---|---|---|---|---|---|---|---|---|---|---|---|---|---|---|---|---|---|---|---|---|---|---|---|
| *Hemipsilichthys gobio* | - | 0 | 0 | - | 0 | 0 | 0 | 0 | 0 | 0 | 0 | 0 | 0 | 0 | 0 | 0 | 0 | 0 | 0 | 0 | 0 | 0 | 0 | 0 |
| *Acestridium scutatum* | 0 | 0 | 2 | 0 | 1 | 2 | 0 | 2 | 1 | - | 0 | 1 | 0 | 0 | 0 | 0 | 0 | 0 | 0 | 0 | 2 | 1 | 0 | 0 |
| *Ancistrus brevipinnis* | 0 | 0 | 1 | 0 | 0 | 1 | 1 | 0 | 0 | 0 | 0 | 0 | 1 | 3 | 0 | 1 | 0 | 0 | 0 | 0 | 0 | 0 | 0 | 0 |
| *Chaetostoma breve* | 0 | 0 | 1 | 0 | 0 | 1 | 1 | 0 | 0 | 0 | 0 | 0 | 1 | 0 | 0 | 1 | 0 | 0 | 0 | 0 | 0 | 0 | 0 | 0 |
| *Pseudohemiodon lamina* | 2 | 0 | 2 | 0 | 1 | 2 | 3 | 3 | 1 | - | 1 | 0 | 2 | 0 | 0 | 1 | 0 | 1 | 1 | 2 | 1 | 1 | 2 | 0 |
| *Dasyloricaria filamentosa* | 1 | 0 | 2 | 0 | 0 | 0 | 3 | 0 | 0 | 1 | 1 | 0 | 2 | 2 | 1 | 1 | 1 | 1 | 1 | 0 | 1 | 1 | 0 | 0 |
| *Dasyloricaria latiura* | 1 | 0 | 2 | 0 | 0 | 0 | 3 | 0 | 1 | - | 1 | 0 | 2 | 2 | 1 | 1 | 0 | 1 | 2 | 1 | 1 | 0 | 0 | 0 |
| *Dasyloricaria paucisquama* | 1 | 0 | 2 | 0 | 0 | 0 | 3 | 0 | 0 | 1 | 1 | 0 | 2 | 2 | 1 | 1 | 1 | 1 | 1 | 0 | 1 | 1 | 0 | 0 |
| *Hemiodontichthys acipenserinus* | 2 | 0 | 2 | 0 | 0 | 0 | 3 | 0 | 1 | - | 1 | 2 | 2 | 0 | 1 | 2 | 2 | 0 | 1 | 0 | 2 | 2 | 1 | 0 |
| *Hisonotus laevior* | 0 | 0 | 2 | 0 | 1 | 2 | 0 | 2 | 0 | 0 | 0 | 1 | 0 | 0 | 0 | 0 | 0 | 0 | 0 | 0 | 0 | 1 | 0 | 0 |
| *Limatulichthys griseus* | 1 | 0 | 2 | 0 | 0 | 0 | 3 | 3 | 1 | - | 1 | 0 | 2 | 1 | 1 | 2 | 0 | 1 | 2 | 2 | 1 | 1 | 1 | 0 |
| *Loricaria lundbergi* | 1 | 0 | 2 | 0 | 0 | 2 | 3 | 1 | 0 | 0 | 1 | 0 | 2 | 0 | 0 | 2 | 1 | 1 | 1 | 1 | 1 | 1 | 2 | 0 |
| *Loricariichthys anus* | 2 | 2 | 2 | 0 | 0 | 0 | 3 | 0 | 0 | 1 | 1 | 0 | 2 | 0 | 1 | 1 | 2 | 1 | 2 | 0 | 1 | 1 | 1 | 0 |
| *Loricariichthys platymetopon* | 2 | 2 | 2 | 0 | 0 | 0 | 3 | 0 | 0 | 1 | 1 | 0 | 2 | 0 | 1 | 1 | 2 | 1 | 2 | 0 | 1 | 1 | 1 | 0 |
| *Neoplecostomus microps* | 0 | 0 | 1 | 0 | 0 | 2 | 1 | 0 | 0 | 0 | 0 | 0 | 0 | 0 | 0 | 0 | 0 | 0 | 0 | 0 | 0 | 0 | 0 | 0 |
| *Pareiorhaphis calmoni* | 0 | 0 | 2 | 0 | 0 | 2 | 1 | 0 | 0 | 0 | 0 | 0 | 0 | 0 | 0 | 0 | 2 | 0 | 0 | 0 | 0 | 0 | 0 | 0 |
| *Parotocinclus maculicauda* | 0 | 0 | 2 | 0 | 1 | 2 | 1 | 2 | 0 | 0 | 0 | 0 | 0 | 0 | 0 | 0 | 2 | 0 | 0 | 0 | 0 | 1 | 0 | 0 |
| *Pterygoplichthys lituratus* | 2 | 0 | 0 | 0 | 0 | 0 | 1 | 0 | 0 | 0 | 0 | 0 | 1 | 3 | 0 | 1 | 2 | 0 | 0 | 2 | 0 | 0 | 0 | 0 |
| *Rineloricaria cadeae* | 2 | 0 | 2 | 0 | 0 | 0 | 3 | 0 | 1 | - | 1 | 0 | 2 | 0 | 0 | 1 | 1 | 1 | 1 | 0 | 1 | 1 | 0 | 0 |
| *Rineloricaria lanceolata* | 1 | 0 | 2 | 0 | 1 | 0 | 3 | 0 | 1 | - | 1 | 0 | 2 | 1 | 0 | 2 | 0 | 1 | 2 | 1 | 0 | 1 | 0 | 0 |

| Taxon/Character | 172 | 173 | 174 | 175 | 176 | 177 | 178 | 179 | 180 | 181 | 182 | 183 | 184 | 185 | 186 | 187 | 188 | 189 | 190 | 191 | 192 | 193 | 194 | 195 | 196 |
|---|---|---|---|---|---|---|---|---|---|---|---|---|---|---|---|---|---|---|---|---|---|---|---|---|---|
| ***Hemipsilichthys gobio*** | 0 | 0 | 0 | 0 | 0 | 0 | 0 | 0 | - | - | 0 | 0 | 0 | 0 | 0 | 0 | 0 | 0 | 0 | 0 | 0 | 0 | 0 | 0 | 0 |
| ***Acestridium scutatum*** | 0 | 0 | 1 | 0 | 3 | 1 | 1 | 0 | 2 | 1 | 1 | 0 | 1 | 0 | 1 | 1 | 1 | 0 | 0 | 2 | 0 | 2 | 0 | 0 | 0 |
| ***Ancistrus brevipinnis*** | 0 | 0 | 1 | 0 | 0 | 2 | 0 | 0 | - | - | 0 | 0 | 0 | 2 | 2 | 0 | 0 | 0 | 0 | 0 | 0 | 0 | 0 | 0 | 0 |
| ***Chaetostoma breve*** | 0 | 0 | 1 | 0 | 3 | 4 | 0 | 0 | - | - | 0 | 0 | 0 | 1 | 2 | 0 | 0 | 0 | 0 | 0 | 0 | 0 | 0 | 0 | 0 |
| ***Pseudohemiodon lamina*** | 2 | 2 | 2 | 0 | 3 | 0 | 1 | 0 | 0 | 0 | 1 | 0 | 0 | 0 | 1 | 0 | 1 | 0 | 1 | 3 | 1 | 0 | 0 | 1 | 0 |
| ***Dasyloricaria filamentosa*** | 1 | 0 | 0 | 0 | 3 | 0 | 1 | 0 | 2 | 1 | 2 | 0 | 0 | 0 | 1 | 1 | 1 | 0 | 1 | 3 | 2 | 1 | 0 | 1 | 1 |
| ***Dasyloricaria latiura*** | 1 | 0 | 1 | 0 | 3 | 0 | 1 | 0 | 2 | 1 | 1 | 0 | 0 | 1 | 1 | 1 | 1 | 0 | 1 | 3 | 1 | 1 | 0 | 1 | 1 |
| ***Dasyloricaria paucisquama*** | 1 | 0 | 0 | 0 | 3 | 0 | 1 | 0 | 2 | 1 | 2 | 0 | 0 | 0 | 1 | 1 | 1 | 0 | 1 | 3 | 0 | 1 | 0 | 1 | 1 |
| ***Hemiodontichthys acipenserinus*** | 2 | 0 | 3 | 0 | 3 | 0 | 1 | 0 | 3 | 1 | 2 | 0 | 0 | 0 | 0 | 1 | 1 | 0 | 1 | 3 | 0 | 1 | 0 | 2 | 0 |
| ***Hisonotus laevior*** | 0 | 0 | 1 | 0 | 3 | 0 | 1 | 0 | 0 | 0 | 0 | 0 | 1 | 0 | 1 | 1 | 1 | 0 | 0 | 0 | 0 | 0 | 0 | 1 | 0 |
| ***Limatulichthys griseus*** | 0 | 1 | 0 | 0 | 3 | 0 | 1 | 0 | 3 | 1 | 2 | 0 | 0 | 0 | 1 | 1 | 1 | 0 | 1 | 3 | 0 | 0 | 0 | 1 | 0 |
| ***Loricaria lundbergi*** | 2 | 2 | 2 | 0 | 3 | 0 | 1 | 0 | 2 | 0 | 1 | 0 | 0 | 2 | 1 | 0 | 1 | 0 | 1 | 3 | 1 | 1 | 0 | 2 | 1 |
| ***Loricariichthys anus*** | 0 | 0 | 0 | 0 | 0 | 2 | 1 | 0 | 2 | 1 | 2 | 0 | 0 | 1 | 1 | 1 | 1 | 0 | 1 | 3 | 0 | 3 | 0 | 3 | 0 |
| ***Loricariichthys platymetopon*** | 2 | 0 | 0 | 0 | 0 | 0 | 1 | 0 | 3 | 1 | 2 | 0 | 0 | 1 | 1 | 1 | 1 | 0 | 1 | 3 | 0 | 3 | 0 | 1 | 0 |
| ***Neoplecostomus microps*** | 0 | 0 | 1 | 0 | 3 | 0 | 1 | 0 | 0 | 0 | 0 | 0 | 0 | 0 | 1 | 0 | 0 | 0 | 0 | 0 | 0 | 0 | 0 | 1 | 0 |
| ***Pareiorhaphis calmoni*** | 0 | 0 | 1 | 0 | 3 | 0 | 0 | 0 | - | - | 0 | 0 | 0 | 0 | 1 | 0 | 0 | 0 | 0 | 0 | 0 | 0 | 0 | 1 | 0 |
| ***Parotocinclus maculicauda*** | 0 | 0 | 2 | 0 | 0 | 0 | 1 | 0 | 2 | 0 | 1 | 0 | 1 | 0 | 1 | 1 | 0 | 0 | 0 | 0 | 0 | 0 | 0 | 0 | 0 |
| ***Pterygoplichthys lituratus*** | 1 | 0 | 1 | 0 | 0 | 4 | 0 | 0 | - | - | 0 | 0 | 0 | 2 | 2 | 0 | 0 | 0 | 0 | 0 | 0 | 0 | 0 | 1 | 0 |
| ***Rineloricaria cadeae*** | 1 | 0 | 1 | 0 | 3 | 0 | 1 | 0 | 2 | 0 | 1 | 0 | 0 | 0 | 1 | 1 | 1 | 0 | 1 | 3 | 0 | 1 | 0 | 1 | 1 |
| ***Rineloricaria lanceolata*** | 2 | 0 | 1 | 0 | 0 | 0 | 1 | 0 | 2 | 0 | 1 | 0 | 0 | 1 | 1 | 1 | 1 | 0 | 1 | 3 | 1 | 1 | 0 | 3 | 1 |

| **Taxon/Character** | **1** | **2** | **3** | **4** | **5** | **6** | **7** | **8** | **9** | **10** | **11** | **12** | **13** | **14** | **15** | **16** | **17** | **18** | **19** | **20** | **21** | **22** | **23** | **24** | **25** | **26** |
|---|---|---|---|---|---|---|---|---|---|---|---|---|---|---|---|---|---|---|---|---|---|---|---|---|---|---|
| ***Rineloricaria quadrensis*** | 1 | 0 | 0 | 0 | 3 | 1 | 3 | 2 | 1 | 0 | 1 | 0 | 4 | 0 | 0 | 1 | 1 | 1 | 4 | 2 | 1 | 1 | 1 | 0 | 0 | 0 |
| ***Spatuloricaria puganensis*** | 0 | 0 | 0 | 0 | 3 | 0 | 3 | 3 | 1 | 1 | 1 | 0 | 2 | 0 | 0 | 1 | 1 | 1 | 4 | 2 | 2 | 3 | 1 | 0 | 1 | 1 |
| ***Aposturisoma myriodon*** | 1 | 0 | 0 | 0 | 1 | 2 | 1 | 1 | 0 | 1 | 1 | 3 | 1 | 0 | 1 | 1 | 1 | 0 | 2 | 0 | 1 | 2 | 0 | 1 | 0 | 0 |
| ***Cteniloricaria platystoma*** | 0 | 0 | 1 | 0 | 1 | 1 | 1 | 2 | 0 | 0 | 0 | 3 | 1 | 0 | 0 | 0 | 0 | 0 | 3 | 0 | 1 | 1 | 0 | 0 | 1 | 1 |
| ***Farlowella acus*** | 1 | 0 | ? | 0 | 1 | 2 | 0 | 1 | 0 | 1 | 2 | - | 2 | 0 | 0 | 1 | 1 | 0 | 1 | 0 | 1 | 1 | 1 | 1 | 1 | 2 |
| ***Farlowella amazonum*** | 1 | 0 | ? | 0 | 2 | 2 | 1 | 1 | 0 | 1 | 1 | 3 | 2 | 0 | 0 | 0 | 0 | 0 | 2 | 1 | 1 | 1 | 1 | 0 | 1 | 0 |
| ***Farlowella curtirostra*** | 1 | 0 | ? | 0 | 1 | 2 | 2 | 1 | 0 | 1 | 1 | 3 | 2 | 0 | 0 | 0 | 0 | 0 | ? | 1 | 1 | 1 | 1 | 0 | 1 | 0 |
| ***Farlowella hahni*** | 1 | 0 | ? | 0 | 1 | 2 | 1 | 1 | 0 | 1 | 1 | 3 | 2 | 0 | 0 | 0 | 0 | 0 | 3 | 0 | 1 | 2 | 1 | 0 | 1 | 0 |
| ***Farlowella hasemani*** | 1 | 0 | 1 | 0 | 1 | 2 | 2 | 1 | 0 | 1 | 2 | 3 | 2 | 0 | 0 | 1 | 1 | 0 | 0 | 1 | 1 | 1 | 1 | 0 | 1 | 0 |
| ***Farlowella henriquei*** | 1 | 0 | 1 | 0 | 1 | 2 | 1 | 1 | 0 | 1 | 1 | 1 | 2 | 0 | 0 | 0 | 0 | 0 | 2 | 1 | 1 | 1 | 1 | 0 | 1 | 2 |
| ***Farlowella isbruckeri*** | 1 | 0 | 1 | 0 | 1 | 2 | 0 | 1 | 0 | 1 | 2 | 3 | 2 | 1 | 0 | 1 | 1 | 0 | 2 | 0 | 1 | 1 | 1 | 0 | 1 | 2 |
| ***Farlowella jauruensis*** | 1 | 0 | 1 | 0 | 2 | 2 | 2 | 1 | 0 | 1 | 2 | 3 | 2 | 0 | 0 | 1 | 1 | 0 | 1 | 0 | 1 | 1 | 1 | 0 | 1 | 2 |
| ***Farlowella knerii*** | 1 | 0 | 0 | 0 | 1 | 2 | 1 | 1 | 0 | 1 | 1 | 1 | 2 | 0 | 0 | 0 | 0 | 0 | 0 | 1 | 1 | 1 | 1 | 0 | 0 | 0 |
| ***Farlowella mariaelenae*** | 1 | 0 | 0 | 0 | 1 | 2 | 2 | 1 | 0 | 1 | 2 | 3 | 2 | 1 | 0 | 1 | 1 | 0 | 1 | 0 | 1 | 1 | 1 | 0 | 1 | 0 |
| ***Farlowella nattereri*** | 1 | 0 | 1 | 0 | 1 | 2 | 1 | 1 | 0 | 1 | 1 | 1 | 2 | 0 | 0 | 0 | 0 | 0 | 0 | 1 | 1 | 1 | 1 | 0 | 1 | 0 |
| ***Farlowella oxyrryncha*** | 1 | 0 | 1 | 0 | 1 | 2 | 2 | 1 | 0 | 1 | 2 | 3 | 2 | 0 | 0 | 1 | 1 | 0 | 0 | 1 | 1 | 1 | 1 | 0 | 1 | 0 |
| ***Farlowella paraguayensis*** | 1 | 0 | 0 | 0 | 1 | 2 | 1 | 1 | 0 | 1 | 1 | 1 | 2 | 0 | 0 | 0 | 0 | 0 | 2 | 1 | 1 | 1 | 1 | 0 | 1 | 0 |
| ***Farlowella* aff. *amazonum*** | 1 | 0 | 1 | 0 | 1 | 2 | 1 | 1 | 0 | 1 | 1 | 0 | 2 | 0 | 0 | 1 | 1 | 0 | 0 | 1 | 1 | 2 | 1 | 0 | 1 | 0 |
| ***Farlowella reticulata*** | 1 | 0 | 1 | 0 | 2 | 2 | 2 | 1 | 0 | 1 | 1 | 3 | 2 | 0 | 0 | 1 | 1 | 0 | 1 | 0 | 1 | 1 | 1 | 0 | 1 | 0 |
| ***Farlowella rugosa*** | 1 | 0 | 1 | 0 | 1 | 2 | 2 | 1 | 0 | 1 | 1 | 1 | 2 | 0 | 0 | 0 | 0 | 0 | 2 | 2 | 1 | 1 | 1 | 0 | 1 | 0 |
| ***Farlowella schreitmuelleri*** | 1 | 0 | 0 | 0 | 1 | 2 | 1 | 1 | 0 | 1 | 1 | 1 | 2 | 0 | 0 | 0 | 0 | 0 | 0 | 1 | 1 | 1 | 1 | 0 | 1 | 0 |

| **Taxon/Character** | **27** | **28** | **29** | **30** | **31** | **32** | **33** | **34** | **35** | **36** | **37** | **38** | **39** | **40** | **41** | **42** | **43** | **44** | **45** | **46** | **47** | **48** | **49** | **50** |
|---|---|---|---|---|---|---|---|---|---|---|---|---|---|---|---|---|---|---|---|---|---|---|---|---|
| ***Rineloricaria quadrensis*** | 1 | 0 | 1 | 1 | 1 | 0 | 1 | 1 | 0 | 0 | 0 | 3 | 1 | 1 | 2 | 1 | 0 | 1 | 0 | 2 | 3 | 1 | 2 | 1 |
| ***Spatuloricaria puganensis*** | 1 | 0 | 1 | 1 | 1 | 0 | 4 | 1 | 0 | 0 | 0 | 3 | 1 | 2 | 0 | 0 | 0 | 1 | 0 | 2 | 3 | 1 | 2 | 1 |
| ***Aposturisoma myriodon*** | 1 | 1 | 0 | 0 | 0 | 0 | 0 | 2 | 1 | 0 | 0 | 4 | 0 | 2 | 1 | 2 | 0 | 0 | 0 | 0 | 0 | 0 | 0 | 0 |
| ***Cteniloricaria platystoma*** | 0 | 0 | 1 | 1 | 1 | 0 | 2 | 2 | 1 | 0 | 0 | 4 | 0 | 2 | 1 | 2 | 1 | 0 | 1 | 0 | 1 | 0 | 1 | 0 |
| ***Farlowella acus*** | 0 | 1 | 1 | 1 | ? | ? | 1 | 2 | 0 | 0 | 0 | 2 | 1 | 1 | 0 | 2 | 0 | 0 | ? | 1 | 1 | 0 | 2 | 0 |
| ***Farlowella amazonum*** | 0 | 1 | 0 | 1 | 0 | 0 | 1 | 2 | 0 | 0 | 0 | 2 | 1 | 1 | 0 | 2 | 1 | 0 | 0 | 1 | 1 | 0 | 0 | 0 |
| ***Farlowella curtirostra*** | 0 | 1 | 0 | 1 | 0 | ? | 1 | 2 | 0 | 0 | 0 | 3 | 1 | 1 | 1 | 2 | 1 | 0 | ? | ? | 1 | 0 | 0 | 0 |
| ***Farlowella hahni*** | 1 | 1 | 0 | 0 | 1 | ? | 0 | 2 | 0 | 0 | 0 | 3 | 1 | 1 | 1 | 2 | 1 | 0 | ? | 1 | 1 | 0 | 0 | 0 |
| ***Farlowella hasemani*** | 0 | 1 | ? | 1 | ? | 2 | 0 | 2 | 0 | 0 | 0 | 3 | 1 | 2 | 1 | 2 | 1 | 0 | 0 | 1 | 1 | 0 | 0 | 0 |
| ***Farlowella henriquei*** | 1 | 1 | 0 | 1 | 0 | 0 | 1 | 2 | 0 | 1 | 0 | 3 | 1 | 1 | 1 | 0 | 1 | 0 | 0 | 1 | 1 | 0 | 0 | 0 |
| ***Farlowella isbruckeri*** | 1 | 1 | 0 | 1 | 0 | 2 | 1 | 2 | 0 | 0 | 0 | 3 | 1 | 2 | 1 | 2 | 1 | 0 | 0 | 1 | 1 | 0 | 0 | 0 |
| ***Farlowella jauruensis*** | 0 | 1 | 0 | 1 | 0 | 0 | 0 | 2 | 0 | 0 | 0 | 3 | 1 | 1 | 1 | 0 | 1 | 0 | 0 | 1 | 1 | 0 | 0 | 0 |
| ***Farlowella knerii*** | 0 | 1 | 0 | 1 | 0 | ? | 0 | 2 | 0 | 0 | 0 | 3 | 1 | 2 | 1 | 2 | 1 | 0 | 0 | 1 | 1 | 0 | 0 | 0 |
| ***Farlowella mariaelenae*** | 1 | 1 | 0 | 1 | 0 | 0 | 0 | 2 | 0 | 0 | 0 | 4 | 1 | 1 | 1 | 2 | 1 | 0 | 0 | 1 | 1 | 0 | 0 | 0 |
| ***Farlowella nattereri*** | 1 | 1 | 0 | 1 | 0 | ? | 0 | 2 | 0 | 0 | 0 | 4 | 1 | 2 | 1 | 2 | 1 | 0 | 0 | 1 | 1 | 0 | 0 | 0 |
| ***Farlowella oxyrryncha*** | 1 | 1 | 0 | 1 | 0 | 1 | 0 | 2 | 0 | 0 | 0 | 3 | 1 | 1 | 0 | 0 | 1 | 0 | 0 | 1 | 2 | 0 | 0 | 0 |
| ***Farlowella paraguayensis*** | 0 | 1 | 0 | 1 | 0 | 2 | 3 | 2 | 0 | 0 | 0 | 3 | 1 | 1 | 1 | 0 | 0 | 0 | 0 | 1 | 1 | 0 | 0 | 0 |
| ***Farlowella* aff. *amazonum*** | 0 | 1 | 0 | 1 | 0 | 1 | 1 | 2 | 1 | 0 | 0 | 4 | 1 | 2 | 1 | 2 | 1 | 0 | 0 | 1 | 1 | 0 | 0 | 0 |
| ***Farlowella reticulata*** | 0 | 1 | 0 | 1 | 0 | 0 | 1 | 2 | 0 | 0 | 1 | 4 | 1 | 1 | 1 | 2 | 1 | 0 | 0 | 1 | 1 | 0 | 0 | 0 |
| ***Farlowella rugosa*** | 0 | 1 | 0 | 1 | 0 | 0 | 1 | 2 | 0 | 1 | 0 | 4 | 1 | 1 | 1 | 2 | 1 | 0 | 0 | 1 | 2 | 0 | 0 | 0 |
| ***Farlowella schreitmuelleri*** | 0 | 1 | 0 | 1 | 0 | 0 | 1 | 2 | 0 | 0 | 0 | 4 | 1 | 1 | 1 | 2 | 1 | 0 | 0 | 0 | 1 | 0 | 0 | 0 |

| **Taxon/Character** | **51** | **52** | **53** | **54** | **55** | **56** | **57** | **58** | **59** | **60** | **61** | **62** | **63** | **64** | **65** | **66** | **67** | **68** | **69** | **70** | **71** | **72** | **73** | **74** |
|---|---|---|---|---|---|---|---|---|---|---|---|---|---|---|---|---|---|---|---|---|---|---|---|---|
| ***Rineloricaria quadrensis*** | 2 | 2 | 2 | 1 | 0 | 0 | 0 | 1 | 2 | 2 | 1 | 0 | 1 | 0 | 1 | 1 | 1 | 1 | 0 | 1 | 1 | 0 | 2 | 3 |
| ***Spatuloricaria puganensis*** | 2 | 3 | 2 | 1 | 0 | 1 | 1 | 1 | 2 | 2 | 1 | 1 | 1 | 1 | 0 | 1 | 1 | 1 | 0 | 1 | 1 | 0 | 2 | 3 |
| ***Aposturisoma myriodon*** | 1 | 0 | 1 | 0 | 1 | 0 | 0 | 0 | 1 | 0 | 1 | 1 | 0 | 0 | 0 | 1 | 2 | 0 | 1 | 0 | 0 | 1 | 1 | 0 |
| ***Ctenilorcaria platystoma*** | 0 | 1 | 1 | 0 | 1 | 0 | 0 | 0 | 2 | 1 | 1 | 2 | 0 | 0 | 1 | 1 | 2 | 1 | 0 | 0 | 0 | 0 | 1 | 1 |
| ***Farlowella acus*** | 1 | 2 | 1 | 0 | 1 | 0 | 0 | 0 | 2 | 2 | 1 | 1 | 1 | 0 | 1 | 1 | 1 | 0 | 1 | 0 | 1 | ? | 1 | 1 |
| ***Farlowella amazonum*** | 2 | 2 | 1 | 0 | 1 | 0 | 0 | 0 | 2 | 2 | 1 | 1 | 0 | 0 | 0 | 1 | 1 | 0 | 1 | 0 | 0 | 1 | 1 | 1 |
| ***Farlowella curtirostra*** | 2 | 2 | 1 | 0 | 1 | 0 | 0 | 0 | 2 | 1 | 1 | 1 | 1 | 0 | 1 | 1 | 1 | 0 | 1 | 0 | 0 | 1 | 1 | 1 |
| ***Farlowella hahni*** | 2 | 2 | 1 | 0 | 1 | 0 | 0 | 0 | 2 | 2 | 1 | 2 | 0 | 1 | 1 | 1 | 1 | 1 | 1 | 0 | 1 | 0 | 1 | 1 |
| ***Farlowella hasemani*** | 2 | 2 | 1 | 0 | 1 | 0 | 0 | 0 | 2 | 2 | 1 | 2 | 0 | 1 | 1 | 1 | 1 | 1 | 1 | ? | 1 | ? | 1 | 1 |
| ***Farlowella henriquei*** | 2 | 2 | 1 | 0 | 1 | 0 | 0 | 0 | 2 | 2 | 1 | 1 | 0 | 0 | 0 | 1 | 1 | 1 | 1 | 0 | 1 | 1 | 1 | 1 |
| ***Farlowella isbruckeri*** | 2 | 2 | 1 | 0 | 1 | 0 | 0 | 0 | 2 | 1 | 1 | 2 | 1 | 1 | 0 | 1 | 1 | 0 | 1 | 0 | 1 | 1 | 1 | 1 |
| ***Farlowella jauruensis*** | 2 | 2 | 1 | 0 | 1 | 0 | 0 | 0 | 2 | 2 | 1 | 1 | 1 | 0 | 0 | 1 | 1 | 0 | 1 | 0 | 1 | 1 | 1 | 1 |
| ***Farlowella knerii*** | 2 | 2 | 1 | 0 | 1 | 0 | 0 | 0 | 2 | 2 | 1 | 2 | 0 | 0 | 0 | 1 | 1 | 1 | 1 | 0 | 1 | 1 | 1 | 1 |
| ***Farlowella mariaelenae*** | 2 | 2 | 1 | 0 | 1 | 0 | 0 | 0 | 2 | 2 | 1 | 2 | 1 | 0 | 0 | 1 | 1 | 0 | 1 | 0 | 0 | 1 | 1 | 1 |
| ***Farlowella nattereri*** | 2 | 2 | 1 | 0 | 1 | 0 | 0 | 0 | 2 | 1 | 1 | 1 | 0 | 0 | 0 | 1 | 1 | 1 | 1 | 0 | 1 | 1 | 1 | 1 |
| ***Farlowella oxyrryncha*** | 2 | 1 | 1 | 0 | 1 | 0 | 0 | 0 | 2 | 1 | 1 | 2 | 0 | 1 | 1 | 1 | 1 | 0 | 1 | 0 | 0 | 1 | 1 | 1 |
| ***Farlowella paraguayensis*** | 2 | 2 | 1 | 0 | 1 | 0 | 0 | 0 | 2 | 2 | 1 | 2 | 0 | 1 | 0 | 1 | 1 | 0 | 1 | 0 | 1 | 1 | 1 | 1 |
| ***Farlowella* aff. *amazonum*** | 2 | 1 | 1 | 0 | 1 | 0 | 0 | 0 | 2 | 2 | 1 | 2 | 0 | 0 | 1 | 1 | 1 | 0 | 1 | 0 | 1 | 1 | 1 | 1 |
| ***Farlowella reticulata*** | 2 | 2 | 1 | 0 | 1 | 0 | 0 | 0 | 2 | 1 | 1 | 1 | 0 | 0 | 1 | 1 | 1 | 0 | 1 | 0 | 1 | 1 | 1 | 1 |
| ***Farlowella rugosa*** | 2 | 2 | 1 | 0 | 1 | 0 | 0 | 0 | 2 | 1 | 1 | 0 | 0 | 0 | 1 | 1 | 1 | 1 | 1 | 0 | 1 | 1 | 1 | 1 |
| ***Farlowella schreitmuelleri*** | 2 | 1 | 1 | 0 | 1 | 0 | 0 | 0 | 2 | 2 | 1 | 2 | 1 | 0 | 1 | 1 | 1 | 0 | 1 | 0 | 1 | 1 | 1 | 1 |

| **Taxon/Character** | **75** | **76** | **77** | **78** | **79** | **80** | **81** | **82** | **83** | **84** | **85** | **86** | **87** | **88** | **89** | **90** | **91** | **92** | **93** | **94** | **95** | **96** | **97** | **98** | **99** |
|---|---|---|---|---|---|---|---|---|---|---|---|---|---|---|---|---|---|---|---|---|---|---|---|---|---|
| ***Rineloricaria quadrensis*** | 2 | 0 | 1 | 2 | 1 | 1 | 0 | 2 | 0 | 1 | 0 | 3 | 0 | 2 | 1 | 1 | 0 | 2 | - | 0 | 0 | 1 | 1 | 0 | 1 |
| ***Spatuloricaria puganensis*** | 2 | 0 | 1 | 2 | 1 | 1 | 0 | 2 | 2 | 0 | 0 | 3 | 1 | ? | ? | ? | 0 | 2 | - | 0 | 3 | 1 | 2 | 0 | 1 |
| ***Aposturisoma myriodon*** | 0 | 1 | 2 | 1 | 0 | 0 | 0 | 1 | 0 | 1 | 0 | 2 | 0 | 2 | 1 | 0 | 0 | 0 | 1 | 1 | 2 | 0 | 3 | 1 | - |
| ***Cteniloricaria platystoma*** | 1 | 1 | 0 | 0 | 0 | 1 | 0 | 3 | 1 | 0 | 1 | 2 | 1 | 1 | 1 | 0 | 0 | 1 | 1 | 2 | 0 | 0 | 0 | 0 | 0 |
| ***Farlowella acus*** | 1 | 1 | 2 | ? | 1 | 1 | 1 | 2 | 0 | 1 | 0 | 1 | 1 | 2 | 1 | 1 | 0 | 0 | 1 | 0 | 0 | 0 | 0 | 0 | 2 |
| ***Farlowella amazonum*** | 1 | 1 | 2 | 0 | 1 | 1 | 0 | 2 | 0 | 1 | 0 | 1 | 1 | 2 | 1 | 1 | 0 | 0 | 1 | 0 | 0 | 0 | 0 | 0 | 2 |
| ***Farlowella curtirostra*** | 1 | 1 | 2 | ? | 1 | 1 | 1 | 2 | 0 | 1 | 0 | 1 | 1 | 2 | 1 | 0 | 0 | 0 | 1 | 0 | 0 | 0 | 0 | 0 | 2 |
| ***Farlowella hahni*** | 1 | 1 | 2 | 3 | 1 | 1 | 0 | 2 | 0 | 1 | 0 | 1 | 1 | 2 | 1 | 1 | 0 | 0 | 1 | 2 | 0 | 0 | 3 | 0 | 2 |
| ***Farlowella hasemani*** | 1 | 1 | 2 | ? | 1 | 1 | 1 | 2 | 0 | 1 | 0 | 1 | 1 | 2 | 1 | 1 | 0 | 0 | 1 | 0 | 0 | 0 | 0 | 0 | 2 |
| ***Farlowella henriquei*** | 1 | 1 | 2 | 0 | 1 | 1 | 1 | 2 | 0 | 1 | 0 | 1 | 1 | 2 | 1 | 1 | 0 | 0 | 1 | 0 | 0 | 0 | 0 | 1 | - |
| ***Farlowella isbruckeri*** | 1 | 1 | 2 | 0 | 1 | 1 | 0 | 2 | 0 | 1 | 0 | 1 | 1 | 2 | 1 | 1 | 0 | 0 | 1 | 0 | 0 | 0 | 0 | 1 | - |
| ***Farlowella jauruensis*** | 1 | 1 | 1 | 0 | 1 | 1 | 0 | 2 | 0 | 1 | 0 | 1 | 1 | 2 | 1 | 1 | 0 | 0 | 1 | 0 | 0 | 0 | 0 | 1 | - |
| ***Farlowella knerii*** | 1 | 1 | 2 | 0 | 1 | 1 | 0 | 2 | 0 | 1 | 0 | 1 | 1 | 2 | 1 | 1 | 0 | 0 | 1 | 2 | 0 | 0 | 0 | 1 | - |
| ***Farlowella mariaelenae*** | 1 | 1 | 2 | 0 | 1 | 1 | 0 | 2 | 0 | 1 | 0 | 1 | 1 | 2 | 1 | 1 | 0 | 0 | 1 | 0 | 0 | 0 | 0 | 0 | 2 |
| ***Farlowella nattereri*** | 1 | 1 | 2 | 0 | 1 | 1 | 0 | 2 | 0 | 1 | 0 | 1 | 1 | 2 | 1 | 1 | 0 | 0 | 1 | 2 | 0 | 0 | 0 | 1 | - |
| ***Farlowella oxyrryncha*** | 1 | 1 | 2 | 0 | 1 | 1 | 0 | 2 | 2 | 1 | 0 | 1 | 1 | 2 | 1 | 1 | 0 | 0 | 1 | 0 | 0 | 0 | 0 | 0 | 1 |
| ***Farlowella paraguayensis*** | 1 | 1 | 2 | 0 | 1 | 1 | 0 | 2 | 0 | 1 | 0 | 1 | 1 | 2 | 1 | 1 | 0 | 0 | 1 | 0 | 0 | 0 | 0 | 1 | - |
| ***Farlowella* aff. *amazonum*** | 1 | 1 | 2 | 0 | 1 | 1 | 0 | 2 | 0 | 1 | 0 | 1 | 1 | 2 | 1 | 1 | 0 | 0 | 1 | 2 | 0 | 0 | 0 | 0 | 0 |
| ***Farlowella reticulata*** | 1 | 1 | 2 | 0 | 1 | 1 | 0 | 2 | 0 | 1 | 0 | 1 | 1 | 2 | 1 | 1 | 0 | 0 | 1 | 2 | 0 | 0 | 0 | 0 | 1 |
| ***Farlowella rugosa*** | 1 | 1 | 2 | 0 | 1 | 1 | 0 | 2 | 2 | 1 | 0 | 1 | 1 | 2 | 1 | 1 | 0 | 0 | 1 | 2 | 0 | 0 | 0 | 0 | 2 |
| ***Farlowella schreitmuelleri*** | 1 | 1 | 2 | 0 | 1 | 1 | 0 | 2 | 2 | 1 | 0 | 1 | 1 | 2 | 1 | 1 | 0 | 0 | 1 | 0 | 0 | 0 | 0 | 0 | 2 |

| Taxon/Character | 100 | 101 | 102 | 103 | 104 | 105 | 106 | 107 | 108 | 109 | 110 | 111 | 112 | 113 | 114 | 115 | 116 | 117 | 118 | 119 | 120 | 121 | 122 | 123 |
|---|---|---|---|---|---|---|---|---|---|---|---|---|---|---|---|---|---|---|---|---|---|---|---|---|
| *Rineloricaria quadrensis* | 1 | - | 0 | 3 | 3 | 3 | 1 | 0 | 0 | 0 | 0 | 0 | 0 | 2 | 2 | 1 | 0 | 0 | 1 | 0 | 1 | 0 | 0 | 1 |
| *Spatuloricaria puganensis* | 1 | - | 0 | 1 | 3 | 2 | 1 | 0 | 0 | 0 | 0 | 0 | 0 | 1 | 2 | 0 | 1 | 0 | 2 | 0 | 1 | 0 | 3 | 0 |
| *Aposturisoma myriodon* | 0 | 0 | 1 | 2 | 1 | 2 | 0 | 2 | 1 | 0 | 0 | 0 | 0 | 0 | 2 | 1 | 0 | ? | 3 | 0 | 2 | 1 | 0 | 0 |
| *Cteniloricaria platystoma* | 0 | 1 | 1 | 4 | 1 | 0 | 0 | 1 | 1 | 0 | 1 | 0 | 0 | 0 | 2 | 1 | 0 | 1 | 0 | 0 | 1 | 0 | 1 | 1 |
| *Farlowella acus* | 0 | 0 | 1 | 2 | 1 | 3 | 0 | 2 | 1 | 1 | 0 | 0 | 0 | 0 | 1 | 0 | 0 | ? | ? | 0 | 2 | 1 | 3 | 0 |
| *Farlowella amazonum* | 1 | - | 0 | 3 | 1 | 3 | 0 | 2 | 1 | 1 | 0 | 0 | 0 | 0 | 1 | 0 | 0 | 2 | 3 | 0 | 2 | 1 | 3 | 0 |
| *Farlowella curtirostra* | 0 | 2 | 1 | 2 | 1 | 3 | 0 | 2 | 1 | 1 | 0 | 0 | 0 | 0 | 1 | 0 | 0 | 2 | 3 | 0 | 2 | 1 | 3 | 0 |
| *Farlowella hahni* | 0 | 2 | 1 | 3 | 1 | 2 | 0 | 2 | 1 | 1 | 0 | 0 | 0 | 0 | 1 | 0 | 0 | 2 | 3 | 0 | 2 | 1 | 3 | 0 |
| *Farlowella hasemani* | 0 | 0 | 1 | 2 | 1 | 2 | 0 | 2 | 1 | 1 | 0 | 0 | 0 | 0 | 1 | 0 | 0 | 2 | 3 | 0 | 2 | 1 | 3 | 0 |
| *Farlowella henriquei* | 0 | 0 | 1 | 2 | 1 | 0 | 0 | 2 | 1 | 1 | 0 | 0 | 0 | 0 | 1 | 0 | 0 | 2 | 3 | 0 | 2 | 1 | 3 | 0 |
| *Farlowella isbruckeri* | 1 | - | 1 | 3 | 1 | 2 | 0 | 2 | 1 | 1 | 0 | 0 | 0 | 1 | 1 | 0 | 0 | 2 | 3 | 0 | 2 | 1 | 1 | 0 |
| *Farlowella jauruensis* | 1 | - | 1 | 3 | 1 | 2 | 0 | 2 | 1 | 1 | 0 | 0 | 0 | 0 | 1 | 0 | 1 | 2 | 3 | 0 | 2 | 1 | 2 | 0 |
| *Farlowella knerii* | 1 | - | 1 | 2 | 1 | 2 | 0 | 2 | 1 | 1 | 0 | 0 | 0 | 0 | 1 | 0 | 1 | 2 | 3 | 0 | 2 | 1 | 3 | 0 |
| *Farlowella mariaelenae* | 0 | 0 | 1 | 2 | 1 | 2 | 0 | 2 | 1 | 1 | 0 | 0 | 0 | 0 | 1 | 0 | 0 | 1 | 3 | 0 | 2 | 1 | 3 | 0 |
| *Farlowella nattereri* | 0 | 0 | 1 | 1 | 1 | 2 | 0 | 2 | 1 | 1 | 0 | 0 | 0 | 0 | 1 | 0 | 0 | 2 | 3 | 0 | 2 | 1 | 3 | 0 |
| *Farlowella oxyrryncha* | 1 | - | 0 | 3 | 1 | 2 | 0 | 2 | 1 | 1 | 0 | 0 | 0 | 0 | 1 | 0 | 0 | 2 | 3 | 0 | 2 | 1 | 3 | 0 |
| *Farlowella paraguayensis* | 0 | 2 | 0 | 2 | 1 | 2 | 0 | 2 | 1 | 1 | 0 | 0 | 0 | 1 | 1 | 0 | 0 | 2 | 3 | 0 | 2 | 1 | 3 | 0 |
| *Farlowella* aff. *amazonum* | 0 | 0 | 0 | 3 | 1 | 3 | 0 | 2 | 1 | 1 | 0 | 0 | 0 | 1 | 1 | 0 | 0 | 2 | 3 | 0 | 2 | 1 | 3 | 0 |
| *Farlowella reticulata* | 0 | 2 | 0 | 3 | 1 | 2 | 0 | 2 | 1 | 1 | 0 | 0 | 0 | 0 | 1 | 0 | 0 | 2 | 3 | 0 | 2 | 1 | 2 | 0 |
| *Farlowella rugosa* | 0 | 0 | 1 | 1 | 1 | 2 | 0 | 2 | 1 | 1 | 0 | 0 | 0 | 0 | 1 | 0 | 0 | 2 | 3 | 0 | 2 | 1 | 3 | 0 |
| *Farlowella schreitmuelleri* | 0 | 2 | 0 | 3 | 1 | 2 | 0 | 2 | 1 | 1 | 0 | 0 | 0 | 0 | 1 | 0 | 0 | 2 | 3 | 0 | 2 | 1 | 3 | 0 |

| **Taxon/Character** | **124** | **125** | **126** | **127** | **128** | **129** | **130** | **131** | **132** | **133** | **134** | **135** | **136** | **137** | **138** | **139** | **140** | **141** | **142** | **143** | **144** | **145** | **146** | **147** |
|---|---|---|---|---|---|---|---|---|---|---|---|---|---|---|---|---|---|---|---|---|---|---|---|---|
| ***Rineloricaria quadrensis*** | 1 | 3 | 0 | 0 | 3 | 0 | 2 | 1 | 0 | 1 | 1 | 0 | 1 | 0 | 0 | 1 | 1 | 0 | 1 | 0 | 1 | 1 | 1 | 0 |
| ***Spatuloricaria puganensis*** | 1 | 3 | 1 | 1 | 3 | 0 | 2 | 1 | 0 | 1 | 1 | 0 | 1 | 0 | 0 | 1 | 1 | 0 | ? | 0 | 0 | 1 | 0 | 0 |
| ***Aposturisoma myriodon*** | 1 | 3 | 1 | 1 | 3 | 1 | 2 | 1 | 0 | 1 | 1 | 1 | 1 | 0 | 0 | 0 | 0 | 0 | 1 | 1 | 0 | 1 | 0 | 0 |
| ***Cteniloricaria platystoma*** | 0 | 1 | 0 | 0 | 3 | 0 | 1 | 1 | 1 | 1 | 1 | 1 | 1 | 0 | 1 | 0 | 1 | 0 | 1 | 1 | 0 | 0 | 1 | 0 |
| ***Farlowella acus*** | 1 | 3 | 1 | 1 | 1 | 1 | 0 | 1 | 0 | 1 | 1 | 1 | 1 | 0 | 0 | 0 | 0 | 0 | ? | 2 | 1 | 1 | 0 | 0 |
| ***Farlowella amazonum*** | 1 | 3 | 1 | 1 | 2 | 1 | 0 | 1 | 0 | 1 | 1 | 1 | 1 | 0 | 0 | 0 | 0 | 0 | 1 | 0 | 1 | 1 | 1 | 0 |
| ***Farlowella curtirostra*** | 1 | 3 | 1 | 1 | 2 | 1 | 0 | 1 | 0 | 1 | 1 | 1 | 1 | 0 | 0 | 0 | 1 | 0 | 1 | 0 | 1 | 1 | 0 | 0 |
| ***Farlowella hahni*** | 1 | 3 | 1 | 1 | 2 | 1 | 0 | 1 | 0 | 1 | 1 | 1 | 1 | 0 | 0 | 0 | 1 | 0 | 1 | 0 | 1 | 1 | 0 | 0 |
| ***Farlowella hasemani*** | 1 | 3 | 1 | 1 | 2 | 1 | 1 | 1 | 0 | 1 | 1 | 1 | 1 | 0 | 0 | 0 | 0 | 0 | ? | 2 | 1 | 1 | 1 | 0 |
| ***Farlowella henriquei*** | 1 | 3 | 0 | 0 | 2 | 1 | 2 | 1 | 0 | 1 | 1 | 1 | 1 | 0 | 0 | 0 | 0 | 0 | 1 | 2 | 1 | 1 | 0 | 0 |
| ***Farlowella isbruckeri*** | 1 | 3 | 0 | 0 | 3 | 1 | 0 | 1 | 0 | 1 | 1 | 1 | 1 | 0 | 0 | 0 | 1 | 0 | 1 | 0 | 1 | 1 | 1 | 0 |
| ***Farlowella jauruensis*** | 1 | 3 | 0 | 0 | 2 | 1 | 0 | 1 | 0 | 1 | 1 | 1 | 1 | 0 | 0 | 0 | 0 | 0 | 1 | 1 | 1 | 1 | 0 | 0 |
| ***Farlowella knerii*** | 1 | 2 | 0 | 0 | 2 | 1 | 0 | 1 | 0 | 1 | 1 | 1 | 1 | 0 | 0 | 0 | 0 | 0 | 1 | 0 | 1 | 1 | 1 | 0 |
| ***Farlowella mariaelenae*** | 1 | 3 | 0 | 0 | 3 | 1 | 0 | 1 | 0 | 1 | 1 | 1 | 1 | 0 | 0 | 0 | 1 | 0 | 2 | 0 | 1 | 1 | 0 | 0 |
| ***Farlowella nattereri*** | 1 | 3 | 0 | 0 | 2 | 1 | 2 | 1 | 0 | 1 | 1 | 1 | 1 | 0 | 0 | 0 | 1 | 0 | ? | 0 | 1 | 1 | 0 | 0 |
| ***Farlowella oxyrryncha*** | 1 | 3 | 1 | 1 | 3 | 1 | 0 | 1 | 0 | 1 | 1 | 1 | 1 | 0 | 0 | 0 | 0 | 0 | 1 | 0 | 1 | 1 | 1 | 0 |
| ***Farlowella paraguayensis*** | 1 | 3 | 0 | 0 | 3 | 1 | 0 | 1 | 0 | 1 | 1 | 1 | 1 | 0 | 0 | 0 | 0 | 0 | ? | 0 | 1 | 1 | 1 | 0 |
| ***Farlowella* aff. *amazonum*** | 1 | 3 | 1 | 1 | 3 | 1 | 0 | 1 | 0 | 1 | 1 | 1 | 1 | 0 | 0 | 0 | 0 | 0 | 2 | 2 | 1 | 1 | 0 | 0 |
| ***Farlowella reticulata*** | 1 | 3 | 0 | 0 | 3 | 1 | 1 | 1 | 0 | 1 | 1 | 1 | 1 | 0 | 0 | 0 | 0 | 0 | 1 | 2 | 1 | 1 | 1 | 0 |
| ***Farlowella rugosa*** | 1 | 3 | 0 | 0 | 3 | 1 | 0 | 1 | 0 | 1 | 1 | 1 | 1 | 0 | 0 | 0 | 0 | 0 | 1 | 0 | 1 | 1 | 0 | 0 |
| ***Farlowella schreitmuelleri*** | 1 | 3 | 0 | 0 | 3 | 1 | 0 | 1 | 1 | 1 | 1 | 1 | 1 | 0 | 0 | 0 | 0 | 0 | 1 | 0 | 1 | 1 | 0 | 0 |

| **Taxon/Character** | **148** | **149** | **150** | **151** | **152** | **153** | **154** | **155** | **156** | **157** | **158** | **159** | **160** | **161** | **162** | **163** | **164** | **165** | **166** | **167** | **168** | **169** | **170** | **171** |
|---|---|---|---|---|---|---|---|---|---|---|---|---|---|---|---|---|---|---|---|---|---|---|---|---|
| ***Rineloricaria quadrensis*** | 2 | 0 | 2 | 0 | 0 | 0 | 3 | 0 | 0 | 0 | 1 | 0 | 2 | 0 | 0 | 1 | 1 | 1 | 1 | 0 | 1 | 1 | 0 | 0 |
| ***Spatuloricaria puganensis*** | 2 | 0 | 2 | 0 | 0 | 0 | 3 | 0 | 0 | 2 | 1 | 0 | 2 | 1 | 1 | 2 | 0 | 1 | 2 | 1 | 1 | 1 | 0 | 0 |
| ***Aposturisoma myriodon*** | 2 | 0 | 2 | 0 | 0 | 0 | 2 | 1 | 0 | 2 | 1 | 2 | 2 | 1 | 0 | 2 | 2 | 1 | 0 | 0 | 1 | 1 | 0 | 1 |
| ***Ctenilorica platystoma*** | 2 | 0 | 1 | 1 | 1 | 0 | 1 | 0 | 1 | - | 1 | 1 | 2 | 0 | 0 | 2 | 2 | 1 | 2 | 1 | 0 | 2 | 0 | 0 |
| ***Farlowella acus*** | 1 | 0 | 2 | 0 | 0 | 0 | 2 | 1 | 1 | - | 1 | 1 | 0 | 1 | 0 | 2 | 2 | 1 | 1 | 0 | 2 | 1 | 0 | 1 |
| ***Farlowella amazonum*** | 1 | 0 | 2 | 0 | 0 | 0 | 2 | 1 | 1 | - | 1 | 1 | 0 | 1 | 1 | 2 | 2 | 1 | 1 | 1 | 2 | 1 | 0 | 1 |
| ***Farlowella curtirostra*** | 1 | 0 | 2 | 0 | 0 | 0 | 2 | 1 | 1 | - | 1 | 1 | 0 | 1 | 1 | 2 | 2 | 1 | 1 | 2 | 1 | 2 | 0 | 1 |
| ***Farlowella hahni*** | 1 | 0 | 2 | 0 | 0 | 0 | 2 | 1 | 0 | 2 | 1 | 1 | 0 | 1 | 1 | 2 | 2 | 1 | 1 | 1 | 2 | 1 | 0 | 1 |
| ***Farlowella hasemani*** | 1 | 1 | 2 | 0 | 0 | 0 | 2 | 1 | 0 | 1 | 1 | 2 | 0 | 2 | 1 | 2 | 2 | 1 | 1 | 1 | 2 | 1 | 0 | 1 |
| ***Farlowella henriquei*** | 1 | 1 | 1 | 0 | 0 | 0 | 2 | 1 | 0 | 2 | 1 | 2 | 0 | 0 | 0 | 2 | 0 | 1 | 1 | 2 | 2 | 1 | 0 | 1 |
| ***Farlowella isbruckeri*** | 1 | 0 | 2 | 0 | 0 | 0 | 2 | 1 | 1 | - | 1 | 1 | 0 | 1 | 0 | 2 | 0 | 1 | 1 | 1 | 2 | 1 | 0 | 1 |
| ***Farlowella jauruensis*** | 1 | 0 | 2 | 0 | 0 | 0 | 2 | 1 | 0 | 1 | 1 | 1 | 0 | 0 | 1 | 2 | 2 | 1 | 1 | 0 | 2 | 1 | 0 | 1 |
| ***Farlowella knerii*** | 1 | 0 | 1 | 0 | 0 | 0 | 2 | 1 | 0 | 2 | 1 | 2 | 0 | 0 | 0 | 2 | 0 | 1 | 1 | 1 | 2 | 1 | 0 | 1 |
| ***Farlowella mariaelenae*** | 1 | 0 | 1 | 0 | 0 | 0 | 2 | 1 | 0 | 2 | 1 | 1 | 0 | 1 | 1 | 2 | 0 | 1 | 1 | 0 | 2 | 1 | 0 | 1 |
| ***Farlowella nattereri*** | 1 | 1 | 2 | 0 | 0 | 0 | 2 | 1 | 0 | 2 | 1 | 1 | 0 | 0 | 0 | 2 | 1 | 1 | 1 | 1 | 2 | 1 | 0 | 1 |
| ***Farlowella oxyrryncha*** | 1 | 0 | 2 | 0 | 1 | 0 | 2 | 1 | 0 | 1 | 1 | 2 | 0 | 2 | 1 | 2 | 0 | 1 | 1 | 2 | 2 | 1 | 0 | 1 |
| ***Farlowella paraguayensis*** | 1 | 0 | 2 | 0 | 0 | 0 | 2 | 1 | 0 | 1 | 1 | 1 | 0 | 0 | 1 | 2 | 2 | 1 | 1 | 2 | 2 | 1 | 0 | 1 |
| ***Farlowella* aff. *amazonum*** | 1 | 1 | 2 | 0 | 0 | 0 | 2 | 1 | 0 | 1 | 1 | 2 | 0 | 0 | 0 | 2 | 2 | 1 | 1 | 0 | 2 | 1 | 0 | 1 |
| ***Farlowella reticulata*** | 1 | 0 | 1 | 0 | 0 | 0 | 2 | 1 | 1 | - | 1 | 2 | 0 | 0 | 0 | 2 | 0 | 1 | 1 | 0 | 2 | 1 | 0 | 1 |
| ***Farlowella rugosa*** | 1 | 0 | 1 | 0 | 0 | 0 | 2 | 1 | 0 | 2 | 1 | 2 | 0 | 0 | 1 | 2 | 2 | 1 | 1 | 2 | 2 | 1 | 0 | 1 |
| ***Farlowella schreitmuelleri*** | 1 | 0 | 2 | 0 | 0 | 0 | 2 | 1 | 0 | 2 | 1 | 2 | 0 | 0 | 1 | 2 | 0 | 1 | 1 | 0 | 2 | 1 | 0 | 1 |

| Taxon/Character | 172 | 173 | 174 | 175 | 176 | 177 | 178 | 179 | 180 | 181 | 182 | 183 | 184 | 185 | 186 | 187 | 188 | 189 | 190 | 191 | 192 | 193 | 194 | 195 | 196 |
|---|---|---|---|---|---|---|---|---|---|---|---|---|---|---|---|---|---|---|---|---|---|---|---|---|---|
| ***Rineloricaria quadrensis*** | 1 | 0 | 1 | 0 | 3 | 0 | 1 | 0 | 2 | 0 | 2 | 0 | 0 | 0 | 1 | 1 | 1 | 0 | 1 | 3 | 0 | 1 | 0 | 1 | 1 |
| ***Spatuloricaria puganensis*** | 2 | 0 | 1 | 0 | 3 | 0 | 1 | 0 | 0 | 0 | 1 | 0 | 0 | 2 | 1 | 1 | 1 | 0 | 1 | 3 | 2 | 1 | 0 | 1 | 1 |
| ***Aposturisoma myriodon*** | 0 | ? | 1 | 0 | 0 | ? | 1 | 1 | 3 | 1 | 2 | 0 | 0 | 0 | 1 | 1 | 1 | 0 | 1 | 2 | 0 | 2 | 0 | 2 | 0 |
| ***Cteniloricaria platystoma*** | 0 | 0 | 1 | 0 | 0 | 0 | 1 | 0 | 1 | 0 | 2 | 0 | 0 | 1 | 0 | 1 | 1 | 0 | 1 | 1 | 0 | 1 | 1 | 2 | 0 |
| ***Farlowella acus*** | 3 | 0 | 0 | 1 | 3 | 3 | 1 | 1 | 3 | - | 2 | 0 | 0 | 0 | 1 | 1 | 1 | 0 | 1 | 3 | 0 | 2 | 0 | 1 | 1 |
| ***Farlowella amazonum*** | 3 | 0 | 0 | 1 | 3 | 3 | 1 | 1 | 3 | 1 | 2 | 0 | 0 | 0 | 1 | 1 | 1 | 0 | 1 | 2 | 0 | 2 | 0 | 1 | 1 |
| ***Farlowella curtirostra*** | 3 | 1 | 0 | 1 | 3 | 3 | 1 | 1 | 3 | 1 | 2 | 0 | 0 | 0 | 1 | 1 | 1 | 0 | 1 | 3 | 0 | 2 | 0 | 1 | 1 |
| ***Farlowella hahni*** | 0 | 1 | 1 | 1 | 3 | 3 | 1 | 1 | 3 | 1 | 2 | 0 | 0 | 0 | 1 | 1 | 1 | 0 | 1 | 2 | 0 | 2 | 0 | 1 | 1 |
| ***Farlowella hasemani*** | 3 | 1 | 1 | 1 | 3 | 3 | 1 | 1 | 3 | 1 | 2 | 0 | 0 | 0 | 1 | 1 | 1 | 0 | 1 | 2 | 1 | 2 | 0 | 1 | 1 |
| ***Farlowella henriquei*** | 3 | 0 | 1 | 1 | 3 | 3 | 1 | 1 | 3 | 1 | 2 | 0 | 0 | 0 | 1 | 1 | 1 | 0 | 1 | 3 | 1 | 2 | 0 | 1 | 1 |
| ***Farlowella isbruckeri*** | 0 | 1 | 1 | 1 | 3 | 3 | 1 | 1 | 3 | 1 | 2 | 0 | 0 | 0 | 1 | 1 | 1 | 0 | 1 | 2 | 0 | 2 | 0 | 1 | 1 |
| ***Farlowella jauruensis*** | 3 | 1 | 1 | 1 | 3 | 3 | 1 | 1 | 3 | 1 | 2 | 0 | 0 | 0 | 1 | 1 | 1 | 0 | 1 | 2 | 1 | 2 | 0 | 1 | 1 |
| ***Farlowella knerii*** | 0 | 1 | 1 | 1 | 3 | 3 | 1 | 1 | 3 | 1 | 2 | 0 | 0 | 0 | 1 | 1 | 1 | 0 | 1 | 2 | 0 | 2 | 0 | 1 | 1 |
| ***Farlowella mariaelenae*** | 3 | 1 | 1 | 1 | 3 | 3 | 1 | 1 | 3 | 1 | 2 | 0 | 0 | 0 | 1 | 1 | 1 | 0 | 1 | 2 | 1 | 2 | 0 | 1 | 1 |
| ***Farlowella nattereri*** | 3 | 1 | 1 | 1 | 3 | 3 | 1 | 1 | 3 | 1 | 2 | 0 | 0 | 0 | 1 | 1 | 1 | 0 | 1 | 3 | 1 | 2 | 0 | 1 | 1 |
| ***Farlowella oxyrryncha*** | 0 | 1 | 1 | 1 | 3 | 3 | 1 | 1 | 3 | 1 | 2 | 0 | 0 | 0 | 1 | 1 | 1 | 0 | 1 | 2 | 0 | 2 | 0 | 1 | 1 |
| ***Farlowella paraguayensis*** | 3 | 1 | 1 | 1 | 3 | 3 | 1 | 1 | 3 | 1 | 2 | 0 | 0 | 0 | 1 | 1 | 1 | 0 | 1 | 2 | 1 | 2 | 0 | 1 | 1 |
| ***Farlowella* aff. *amazonum*** | 3 | 0 | 1 | 1 | 3 | 3 | 1 | 1 | 3 | 1 | 1 | 0 | 0 | 0 | 1 | 1 | 1 | 0 | 1 | 2 | 0 | 2 | 0 | 1 | 1 |
| ***Farlowella reticulata*** | 3 | 0 | 1 | 1 | 3 | 3 | 1 | 1 | 3 | 1 | 2 | 0 | 0 | 0 | 1 | 1 | 1 | 0 | 1 | 2 | 0 | 2 | 0 | 1 | 1 |
| ***Farlowella rugosa*** | 0 | 1 | 1 | 1 | 3 | 3 | 1 | 1 | 3 | 1 | 2 | 0 | 0 | 0 | 1 | 1 | 1 | 0 | 1 | 3 | 1 | 2 | 0 | 1 | 1 |
| ***Farlowella schreitmuelleri*** | 0 | 1 | 1 | 1 | 3 | 3 | 1 | 1 | 3 | 1 | 2 | 0 | 0 | 0 | 1 | 1 | 1 | 0 | 1 | 2 | 1 | 2 | 0 | 1 | 1 |

| **Taxon/Character** | **1** | **2** | **3** | **4** | **5** | **6** | **7** | **8** | **9** | **10** | **11** | **12** | **13** | **14** | **15** | **16** | **17** | **18** | **19** | **20** | **21** | **22** | **23** | **24** | **25** | **26** |
|---|---|---|---|---|---|---|---|---|---|---|---|---|---|---|---|---|---|---|---|---|---|---|---|---|---|---|
| ***Farlowella smithi*** | 1 | 0 | 0 | 0 | 2 | 2 | 2 | 1 | 0 | 1 | 2 | 3 | 2 | 0 | 0 | 0 | 0 | 0 | 0 | 0 | 1 | 1 | 1 | 0 | 1 | 0 |
| ***Farlowella venezuelensis*** | 1 | 0 | 1 | 0 | 1 | 2 | 1 | 1 | 0 | 1 | 1 | 3 | 2 | 0 | 0 | 0 | 0 | 0 | 0 | 2 | 1 | 1 | 1 | 0 | 1 | 2 |
| ***Farlowella vittata*** | 1 | 0 | 1 | 0 | 1 | 2 | 2 | 1 | 0 | 1 | 1 | 3 | 2 | 0 | 0 | 0 | 0 | 0 | 1 | 1 | 1 | 1 | 1 | 0 | 1 | 0 |
| ***Harttia carvalhoi*** | 0 | 1 | 0 | 0 | 1 | 1 | 1 | 2 | 0 | 0 | 0 | 1 | 1 | 0 | 0 | 0 | 0 | 0 | 3 | 1 | 2 | - | 0 | 1 | ? | 0 |
| ***Harttia dissidens*** | 0 | 0 | 0 | 0 | 1 | 1 | 0 | 1 | 0 | 0 | 0 | 1 | 1 | 0 | 0 | 0 | 0 | 0 | 3 | 1 | 2 | - | 0 | 0 | 1 | 0 |
| ***Harttia duriventris*** | 0 | 0 | 0 | 0 | 1 | 1 | 1 | 1 | 0 | 0 | 0 | 1 | 1 | 0 | 0 | 1 | 1 | 0 | 3 | 1 | 2 | - | 1 | 1 | 1 | 0 |
| ***Harttia fluminensis*** | 0 | 0 | 0 | 0 | 1 | 1 | 0 | 2 | 0 | 0 | 0 | 0 | 1 | 0 | 0 | 0 | 0 | 0 | 3 | 0 | 2 | - | 0 | 0 | 1 | 0 |
| ***Harttia fowleri*** | 0 | 0 | 0 | 0 | 1 | 1 | 0 | 1 | 0 | 0 | 0 | 1 | 1 | 0 | 0 | 0 | 0 | 0 | 3 | 0 | 2 | - | 1 | 0 | 1 | 0 |
| ***Harttia garavelloi*** | 0 | 0 | 0 | 0 | 1 | 1 | 0 | 1 | 0 | 0 | 0 | 1 | 1 | 0 | 0 | 1 | 1 | 0 | 3 | 1 | 2 | - | 1 | 0 | 1 | 0 |
| ***Harttia gracilis*** | 0 | 0 | 0 | 0 | 1 | 1 | 0 | 1 | 0 | 0 | 0 | 1 | 1 | 0 | 0 | 0 | 0 | 0 | 3 | 1 | 2 | - | 0 | 1 | 1 | 0 |
| ***Harttia guianensis*** | 0 | 0 | 0 | 0 | 1 | 1 | 0 | 1 | 0 | 0 | 0 | 1 | 1 | 0 | 0 | 0 | 0 | 0 | 3 | 0 | 2 | - | 0 | 0 | 1 | 0 |
| ***Harttia kronei*** | 0 | 0 | 0 | 0 | 1 | 1 | 0 | 1 | 0 | 0 | 0 | 1 | 1 | 0 | 0 | 1 | 1 | 0 | 3 | 1 | 2 | - | 0 | 0 | 1 | 0 |
| ***Harttia leiopleura*** | 0 | 0 | 0 | 0 | 1 | 1 | 1 | 1 | 0 | 0 | 0 | 0 | 1 | 0 | 0 | 1 | 1 | 0 | 3 | 1 | 2 | - | 0 | 1 | 1 | 0 |
| ***Harttia longipinna*** | 0 | 0 | 0 | 0 | 1 | 1 | 1 | 1 | 0 | 0 | 0 | 1 | 1 | 0 | 0 | 0 | 0 | 0 | 3 | 1 | 2 | - | 0 | 0 | 1 | 0 |
| ***Harttia loricariformis*** | 0 | 0 | 0 | 0 | 1 | 1 | 0 | 2 | 0 | 0 | 0 | 1 | 1 | 0 | 0 | 0 | 0 | 0 | 3 | 1 | 2 | - | 0 | 0 | 1 | 0 |
| ***Harttia novalimensis*** | 0 | 0 | 0 | 0 | 1 | 1 | 1 | 2 | 0 | 0 | 0 | 1 | 1 | 0 | 0 | 1 | 1 | 0 | 3 | 1 | 2 | - | 0 | 0 | 1 | 0 |
| ***Harttia punctata*** | 0 | 0 | 1 | 0 | 1 | 1 | 1 | 2 | 0 | 0 | 0 | 0 | 1 | 0 | 0 | 1 | 1 | 0 | 3 | 0 | 2 | - | 0 | 0 | 0 | 0 |
| ***Harttia rhombocephala*** | 0 | 0 | 0 | 0 | 1 | 1 | 1 | 2 | 0 | 0 | 0 | 1 | 1 | 0 | 0 | 0 | 0 | 0 | 3 | 1 | 2 | - | 0 | 0 | 0 | 0 |
| ***Harttia torrenticola*** | 0 | 0 | 0 | 0 | 1 | 1 | 0 | 2 | 0 | 0 | 0 | 1 | 1 | 0 | 0 | 1 | 1 | 0 | 3 | 1 | 2 | - | 0 | 1 | 1 | 0 |
| ***Harttia trombetensis*** | 0 | 0 | 0 | 0 | 1 | 1 | 1 | 2 | 0 | 0 | 0 | 1 | 1 | 0 | 0 | 0 | 0 | 0 | 3 | 1 | 2 | - | 0 | 1 | 1 | 0 |
| ***Harttiella crassicauda*** | 0 | 0 | 0 | 0 | 1 | 1 | 0 | 1 | 0 | 1 | 0 | 2 | 1 | 0 | 0 | 1 | 0 | 0 | 3 | 0 | 2 | - | 0 | 1 | 0 | 1 |
| ***Harttiella longicauda*** | 0 | 1 | 0 | 0 | 1 | 1 | 0 | 1 | 0 | 1 | 0 | 0 | 1 | 0 | 0 | 1 | 0 | 0 | 3 | 1 | 2 | - | 0 | 1 | 0 | 0 |
| ***Lamontichthys avacanoeiro*** | 0 | 0 | 0 | 0 | 1 | 1 | 0 | 2 | 0 | 1 | 1 | 1 | 1 | 0 | 0 | 1 | 1 | 1 | 2 | 0 | 1 | 1 | 0 | 0 | 1 | 0 |
| ***Lamontichthys filamentosus*** | 1 | 1 | 0 | 0 | 1 | 1 | 0 | 2 | 0 | 1 | 1 | 1 | 0 | 0 | 0 | 0 | 0 | 0 | 3 | 1 | 2 | - | 0 | 0 | 1 | 0 |
| ***Lamontichthys llanero*** | 0 | 1 | 0 | 0 | 1 | 1 | 0 | 1 | 0 | 1 | 0 | 1 | 0 | 0 | 0 | 0 | 0 | 1 | 2 | 0 | 1 | 1 | 0 | 0 | 1 | 0 |

| **Taxon/Character** | **27** | **28** | **29** | **30** | **31** | **32** | **33** | **34** | **35** | **36** | **37** | **38** | **39** | **40** | **41** | **42** | **43** | **44** | **45** | **46** | **47** | **48** | **49** | **50** |
|---|---|---|---|---|---|---|---|---|---|---|---|---|---|---|---|---|---|---|---|---|---|---|---|---|
| ***Farlowella smithi*** | 0 | 1 | 0 | 1 | 0 | 2 | 0 | 2 | 0 | 0 | 0 | 4 | 1 | 2 | 1 | 0 | 1 | 0 | 0 | 1 | 1 | 0 | 0 | 0 |
| ***Farlowella venezuelensis*** | 0 | 1 | 0 | 1 | 0 | ? | 0 | 2 | 0 | 0 | 0 | 4 | 1 | 1 | 1 | 2 | 1 | 0 | 0 | 1 | 2 | 0 | 0 | 0 |
| ***Farlowella vittata*** | 1 | 1 | 0 | 1 | 0 | 0 | 1 | 2 | 0 | 0 | 1 | 4 | 1 | 2 | 1 | 2 | 1 | 0 | 0 | 0 | 1 | 0 | 0 | 0 |
| ***Harttia carvalhoi*** | 0 | 0 | 1 | 1 | 1 | 0 | 2 | 2 | 1 | 0 | 0 | 4 | 1 | 2 | 1 | 2 | 1 | 0 | 1 | 0 | 0 | 0 | 1 | 0 |
| ***Harttia dissidens*** | 0 | 0 | 1 | 0 | 1 | 2 | 0 | 2 | 1 | 0 | 0 | 3 | 1 | 2 | 1 | 2 | 1 | 0 | 1 | 0 | 0 | 0 | 1 | 0 |
| ***Harttia duriventris*** | 0 | 0 | 1 | 0 | 1 | 0 | 2 | 2 | 1 | 0 | 0 | 4 | 1 | 2 | 1 | 2 | 1 | 0 | 0 | 0 | 0 | 0 | 0 | 0 |
| ***Harttia fluminensis*** | 0 | 0 | 1 | 1 | 0 | 0 | 2 | 2 | 1 | 0 | 0 | 4 | 1 | 2 | 1 | 2 | 1 | 0 | 1 | 0 | 0 | 0 | 1 | 0 |
| ***Harttia fowleri*** | 0 | 0 | 1 | 1 | 1 | 0 | 2 | 2 | 1 | 0 | 0 | 2 | 1 | 2 | 1 | 2 | 1 | 0 | 0 | 0 | 0 | 0 | 1 | 0 |
| ***Harttia garavelloi*** | 0 | 0 | 1 | 1 | 0 | 0 | 1 | 2 | 1 | 0 | 0 | 3 | 1 | 1 | 1 | 2 | 1 | 0 | 1 | 0 | 0 | 0 | 0 | 0 |
| ***Harttia gracilis*** | 0 | 0 | 1 | 1 | 1 | 0 | 1 | 2 | 1 | 0 | 0 | 4 | 1 | 2 | 1 | 2 | 1 | 0 | 1 | 0 | 0 | 0 | 0 | 0 |
| ***Harttia guianensis*** | 0 | 0 | 1 | 1 | 0 | 0 | 2 | 2 | 1 | 0 | 0 | 4 | 1 | 2 | 1 | 2 | 1 | 0 | 1 | 0 | 0 | 0 | 0 | 0 |
| ***Harttia kronei*** | 0 | 0 | 1 | 0 | 1 | 0 | 2 | 2 | 1 | 0 | 0 | 4 | 1 | 1 | 1 | 2 | 1 | 0 | 1 | 0 | 0 | 0 | 0 | 0 |
| ***Harttia leiopleura*** | 1 | 0 | 1 | 1 | 1 | 0 | 1 | 2 | ? | ? | 0 | 3 | 1 | 2 | 1 | 2 | 1 | 0 | 1 | 0 | 0 | 0 | 0 | 0 |
| ***Harttia longipinna*** | 0 | 0 | 1 | 1 | 1 | 0 | 1 | 2 | ? | ? | 0 | 4 | 1 | 2 | 1 | 2 | 1 | 0 | 0 | 0 | 0 | 0 | 0 | 0 |
| ***Harttia loricariformis*** | 0 | 0 | 1 | 0 | 0 | 0 | 1 | 2 | 1 | 0 | 0 | 0 | 1 | 0 | 0 | 2 | 1 | 0 | 1 | 0 | 0 | 0 | 0 | 0 |
| ***Harttia novalimensis*** | 0 | 0 | 1 | 1 | 0 | 0 | 2 | 2 | 1 | 0 | 0 | 4 | 1 | 2 | 0 | 2 | 1 | 0 | 1 | 0 | 0 | 0 | 0 | 0 |
| ***Harttia punctata*** | 0 | 0 | 1 | 0 | 1 | 2 | 2 | 2 | 1 | 0 | 0 | 4 | 1 | 2 | 1 | 2 | 1 | 0 | 1 | 0 | 0 | 0 | 1 | 0 |
| ***Harttia rhombocephala*** | 0 | 0 | 1 | 1 | 1 | 0 | 2 | 2 | 1 | 0 | 0 | 4 | 1 | 2 | 1 | 2 | 1 | 0 | 1 | 0 | 0 | 0 | 1 | 0 |
| ***Harttia torrenticola*** | 0 | 0 | 1 | 1 | 1 | 0 | 2 | 2 | 1 | 0 | 0 | 4 | 1 | 2 | 1 | 2 | 1 | 0 | 1 | 0 | 0 | 0 | 0 | 0 |
| ***Harttia trombetensis*** | 0 | 0 | 1 | 0 | 1 | 0 | 2 | 2 | 1 | 0 | 0 | 4 | 1 | 2 | 1 | 2 | 1 | 0 | 1 | 0 | 0 | 0 | 0 | 0 |
| ***Harttiella crassicauda*** | 1 | 0 | 1 | 1 | 0 | 2 | 2 | 2 | 2 | 1 | 0 | 4 | 1 | 1 | 0 | 2 | 1 | 0 | 1 | 0 | 1 | 0 | 0 | 0 |
| ***Harttiella longicauda*** | 0 | 0 | 0 | 1 | 0 | 2 | 2 | 2 | 2 | 1 | 0 | 3 | 0 | 2 | 0 | 2 | 1 | 0 | 1 | 0 | 1 | 0 | 0 | 0 |
| ***Lamontichthys avacanoeiro*** | 0 | 0 | 0 | 0 | 1 | 0 | 3 | 2 | 1 | 0 | 0 | 3 | 0 | 2 | 0 | 2 | 0 | 0 | 1 | 0 | 1 | 0 | 2 | 0 |
| ***Lamontichthys filamentosus*** | 0 | 0 | 1 | 1 | 1 | 0 | 3 | 2 | 1 | 0 | 0 | 3 | 0 | 1 | 1 | 2 | 1 | 0 | 1 | 0 | 1 | 0 | 2 | 0 |
| ***Lamontichthys llanero*** | 0 | 0 | 1 | 0 | 1 | 0 | 2 | 2 | 1 | 0 | 0 | 3 | 0 | 1 | 1 | 2 | 1 | 0 | 0 | 0 | 1 | 0 | 2 | 0 |

| **Taxon/Character** | **51** | **52** | **53** | **54** | **55** | **56** | **57** | **58** | **59** | **60** | **61** | **62** | **63** | **64** | **65** | **66** | **67** | **68** | **69** | **70** | **71** | **72** | **73** | **74** |
|---|---|---|---|---|---|---|---|---|---|---|---|---|---|---|---|---|---|---|---|---|---|---|---|---|
| ***Farlowella smithi*** | 2 | 1 | 1 | 0 | 1 | 0 | 0 | 0 | 2 | 2 | 1 | 2 | 1 | 0 | 1 | 1 | 1 | 0 | 1 | 0 | 1 | 1 | 1 | 1 |
| ***Farlowella venezuelensis*** | 1 | 1 | 1 | 0 | 1 | 0 | 0 | 0 | 2 | 1 | 1 | 1 | 1 | 1 | 1 | 1 | 1 | 0 | 1 | 0 | 1 | 1 | 1 | 1 |
| ***Farlowella vittata*** | 1 | 2 | 1 | 0 | 1 | 0 | 0 | 0 | 2 | 2 | 1 | 2 | 1 | 1 | 1 | 1 | 1 | 0 | 1 | 0 | 1 | 1 | 1 | 1 |
| ***Harttia carvalhoi*** | 0 | 0 | 1 | 0 | 1 | 0 | 0 | 0 | 2 | 2 | 1 | 1 | 1 | 1 | 1 | 1 | 2 | 1 | 0 | 0 | 1 | 0 | 1 | 0 |
| ***Harttia dissidens*** | 0 | 1 | 1 | 0 | 1 | 0 | 0 | 0 | 2 | 2 | 1 | 1 | 0 | 0 | 1 | 1 | 2 | 1 | 0 | 1 | 1 | 0 | 1 | 0 |
| ***Harttia duriventris*** | 0 | 1 | 1 | 0 | 1 | 0 | 0 | 0 | 1 | 2 | 1 | 2 | 0 | 1 | 1 | 1 | 2 | 1 | 0 | 1 | 1 | 0 | 1 | 0 |
| ***Harttia fluminensis*** | 0 | 0 | 1 | 0 | 1 | 0 | 0 | 0 | 2 | 0 | 1 | 1 | 0 | 1 | 1 | 1 | 2 | 0 | 0 | 0 | 1 | 1 | 1 | 0 |
| ***Harttia fowleri*** | 0 | 1 | 1 | 0 | 1 | 0 | 0 | 0 | 1 | 0 | 1 | 1 | 1 | 0 | 1 | 1 | 2 | 0 | 0 | 0 | 1 | 0 | 1 | 0 |
| ***Harttia garavelloi*** | 0 | 0 | 1 | 0 | 1 | 0 | 0 | 0 | 1 | 1 | 1 | 1 | 0 | 1 | 1 | 1 | 2 | 1 | 0 | 0 | 0 | 1 | 1 | 0 |
| ***Harttia gracilis*** | 0 | 0 | 1 | 0 | 1 | 0 | 0 | 0 | 1 | 0 | 1 | 1 | 1 | 1 | 1 | 1 | 2 | 1 | 0 | 1 | 1 | 0 | 1 | 0 |
| ***Harttia guianensis*** | 0 | 0 | 1 | 0 | 1 | 0 | 0 | 0 | 2 | 1 | 1 | 2 | 1 | 1 | 1 | 1 | 2 | 1 | 0 | 0 | 1 | 1 | 1 | 0 |
| ***Harttia kronei*** | 0 | 1 | 1 | 0 | 1 | 0 | 0 | 0 | 1 | 2 | 1 | 2 | 1 | 1 | 1 | 1 | 2 | 0 | 0 | 0 | 0 | 0 | 1 | 0 |
| ***Harttia leiopleura*** | 0 | 0 | 1 | 0 | 1 | 0 | 0 | 0 | 1 | 1 | 1 | 2 | 1 | 1 | 1 | 1 | 2 | 1 | 0 | 1 | 1 | 0 | 1 | 0 |
| ***Harttia longipinna*** | 0 | 1 | 1 | 0 | 1 | 0 | 0 | 0 | 2 | 1 | 1 | 1 | 0 | 1 | 1 | 1 | 2 | 1 | 0 | 0 | 0 | 0 | 1 | 0 |
| ***Harttia loricariformis*** | 0 | 0 | 1 | 0 | 1 | 0 | 0 | 0 | 2 | 2 | 1 | 2 | 0 | 0 | 1 | 1 | 2 | 1 | 0 | 0 | 1 | 1 | 1 | 0 |
| ***Harttia novalimensis*** | 0 | 2 | 1 | 0 | 1 | 0 | 0 | 0 | 1 | 1 | 1 | 1 | 1 | 1 | 1 | 1 | 2 | 1 | 0 | 0 | 0 | 1 | 1 | 0 |
| ***Harttia punctata*** | 0 | 1 | 1 | 0 | 1 | 0 | 0 | 0 | 1 | 1 | 1 | 1 | 1 | 1 | 1 | 1 | 2 | 0 | 0 | 0 | 1 | 0 | 1 | 0 |
| ***Harttia rhombocephala*** | 0 | 1 | 1 | 0 | 1 | 0 | 0 | 0 | 1 | 0 | 1 | 2 | 1 | 0 | 1 | 1 | 2 | 0 | 0 | 0 | 1 | 0 | 1 | 0 |
| ***Harttia torrenticola*** | 0 | 1 | 1 | 0 | 1 | 0 | 0 | 0 | 2 | 2 | 1 | 1 | 0 | 1 | 1 | 1 | 2 | 1 | 0 | 1 | 1 | 0 | 1 | 0 |
| ***Harttia trombetensis*** | 0 | 0 | 1 | 0 | 1 | 0 | 0 | 0 | 1 | 1 | 1 | 2 | 0 | 1 | 1 | 1 | 2 | 1 | 0 | 1 | 1 | 0 | 1 | 0 |
| ***Harttiella crassicauda*** | 0 | 1 | 2 | 0 | 1 | 0 | 0 | 0 | 2 | 2 | 1 | 1 | 0 | 1 | 1 | 1 | 2 | 1 | 1 | 1 | 1 | 1 | 1 | 0 |
| ***Harttiella longicauda*** | 0 | 1 | 2 | 0 | 1 | 0 | 0 | 0 | 2 | 2 | 1 | 1 | 1 | 1 | 1 | 1 | 2 | 1 | 1 | 0 | 0 | 1 | 1 | 0 |
| ***Lamontichthys avacanoeiro*** | 1 | 1 | 1 | 0 | 1 | 0 | 0 | 0 | 1 | 1 | 1 | 2 | 0 | 1 | 0 | 1 | 2 | 1 | 0 | 0 | 1 | 0 | 1 | 0 |
| ***Lamontichthys filamentosus*** | 1 | 1 | 1 | 0 | 1 | 0 | 0 | 0 | 1 | 1 | 1 | 2 | 1 | 1 | 0 | 1 | 2 | 1 | 0 | 1 | 0 | 0 | 1 | 0 |
| ***Lamontichthys llanero*** | 1 | 1 | 0 | 0 | 1 | 0 | 0 | 0 | 1 | 1 | 1 | 2 | 1 | 1 | 1 | 1 | 2 | 1 | 0 | 1 | 0 | 0 | 1 | 0 |

| Taxon/Character | 75 | 76 | 77 | 78 | 79 | 80 | 81 | 82 | 83 | 84 | 85 | 86 | 87 | 88 | 89 | 90 | 91 | 92 | 93 | 94 | 95 | 96 | 97 | 98 | 99 |
|---|---|---|---|---|---|---|---|---|---|---|---|---|---|---|---|---|---|---|---|---|---|---|---|---|---|
| *Farlowella smithi* | 1 | 1 | 1 | 0 | 1 | 1 | 0 | 2 | 2 | 1 | 0 | 1 | 1 | 2 | 1 | 1 | 0 | 0 | 1 | 0 | 0 | 0 | 0 | 1 | - |
| *Farlowella venezuelensis* | 1 | 1 | 2 | ? | 1 | 1 | 0 | 2 | 0 | 1 | 0 | 1 | 1 | 2 | 1 | 1 | 0 | 0 | 1 | 0 | 0 | 0 | 0 | 1 | - |
| *Farlowella vittata* | 1 | 1 | 2 | 0 | 1 | 1 | 0 | 2 | 0 | 1 | 0 | 1 | 1 | 2 | 1 | 1 | 0 | 0 | 1 | 0 | 0 | 0 | 0 | 0 | 2 |
| *Harttia carvalhoi* | 0 | 1 | 0 | 0 | 0 | 0 | 0 | 3 | 1 | 1 | 1 | 2 | 0 | 1 | 1 | 0 | 0 | 1 | 1 | 2 | 0 | 0 | 0 | 0 | 1 |
| *Harttia dissidens* | 0 | 1 | 0 | 0 | 0 | 0 | 0 | 3 | 1 | 0 | 1 | 2 | 0 | 1 | 1 | 1 | 0 | 1 | 1 | 2 | 0 | 0 | 0 | 1 | - |
| *Harttia duriventris* | 0 | 1 | 0 | 0 | 0 | 0 | 0 | 3 | 1 | 1 | 1 | 2 | 0 | 1 | 1 | 0 | 0 | 1 | 1 | 2 | 0 | 0 | 0 | 1 | - |
| *Harttia fluminensis* | 0 | 1 | 0 | 0 | 0 | 0 | 0 | 3 | 1 | 1 | 0 | 2 | 0 | 1 | 1 | 0 | 0 | 1 | 1 | 2 | 0 | 0 | 0 | 0 | 0 |
| *Harttia fowleri* | 0 | 1 | 0 | 0 | 0 | 0 | 0 | 3 | 1 | 0 | 1 | 2 | 0 | 1 | 1 | 0 | 0 | 1 | 1 | 2 | 0 | 0 | 0 | 1 | - |
| *Harttia garavelloi* | 0 | 1 | 0 | 0 | 0 | 0 | 0 | 3 | 1 | 1 | 0 | 2 | 0 | 1 | 1 | 0 | 1 | 1 | 1 | 2 | 0 | 0 | 0 | 0 | 2 |
| *Harttia gracilis* | 0 | 1 | 0 | 0 | 0 | 0 | 0 | 3 | 1 | 1 | 0 | 2 | 0 | 1 | 1 | 1 | 0 | 1 | 1 | 2 | 0 | 0 | 0 | 0 | 2 |
| *Harttia guianensis* | 0 | 1 | 0 | 0 | 0 | 0 | 0 | 3 | 1 | 0 | 1 | 2 | 0 | 1 | 1 | 0 | 0 | 1 | 1 | 1 | 0 | 0 | 0 | 1 | - |
| *Harttia kronei* | 0 | 1 | 0 | 0 | 0 | 0 | 0 | 3 | 1 | 1 | 0 | 2 | 0 | 1 | 0 | 0 | 1 | 1 | 1 | 2 | 0 | 0 | 0 | 0 | 1 |
| *Harttia leiopleura* | 0 | 1 | 0 | 0 | 0 | 0 | 0 | 3 | 1 | 1 | 0 | 2 | 0 | 1 | 1 | 0 | 1 | 1 | 1 | 2 | 0 | 0 | 0 | 1 | - |
| *Harttia longipinna* | 0 | 1 | 0 | 0 | 0 | 0 | 0 | 3 | 1 | 1 | 0 | 2 | 0 | 1 | 1 | 0 | 1 | 1 | 1 | 2 | 0 | 0 | 0 | 0 | 2 |
| *Harttia loricariformis* | 0 | 1 | 0 | 0 | 0 | 0 | 0 | 3 | 1 | 1 | 0 | 2 | 0 | 1 | 1 | 0 | 1 | 1 | 1 | 2 | 0 | 0 | 0 | 0 | 1 |
| *Harttia novalimensis* | 0 | 1 | 1 | 0 | 0 | 0 | 0 | 3 | 1 | 1 | 0 | 2 | 0 | 1 | 1 | 0 | 0 | 1 | 1 | 2 | 0 | 0 | 0 | 0 | 2 |
| *Harttia punctata* | 0 | 1 | 0 | 0 | 0 | 0 | 0 | 3 | 1 | 0 | 1 | 2 | 0 | 1 | 1 | 0 | 0 | 1 | 1 | 2 | 0 | 0 | 0 | 1 | - |
| *Harttia rhombocephala* | 0 | 1 | 0 | 0 | 0 | 0 | 0 | 3 | 1 | 0 | 1 | 2 | 0 | 1 | 1 | 0 | 0 | 1 | 1 | 2 | 0 | 0 | 0 | 1 | - |
| *Harttia torrenticola* | 0 | 1 | 1 | 0 | 0 | 0 | 0 | 3 | 1 | 1 | 0 | 2 | 0 | 1 | 1 | 0 | 1 | 1 | 1 | 2 | 0 | 0 | 0 | 0 | 1 |
| *Harttia trombetensis* | 0 | 1 | 0 | 0 | 0 | 0 | 0 | 3 | 1 | 1 | 1 | 2 | 0 | 1 | 1 | 0 | 0 | 1 | 1 | 2 | 0 | 0 | 0 | 1 | - |
| *Harttiella crassicauda* | 0 | 1 | 1 | 0 | 0 | 0 | 0 | 3 | 1 | 1 | 1 | 2 | 1 | 2 | 0 | 0 | 1 | 1 | 1 | 1 | 1 | 0 | 3 | 1 | - |
| *Harttiella longicauda* | 0 | 1 | 1 | 0 | 0 | 0 | 0 | 3 | 1 | 1 | 0 | 2 | 1 | 2 | 0 | 0 | 0 | 1 | 1 | 1 | 1 | 0 | 3 | 1 | - |
| *Lamontichthys avacanoeiro* | 0 | 1 | 0 | 1 | 0 | 0 | 0 | 1 | 2 | 1 | 0 | 3 | 1 | 2 | 1 | 0 | 0 | 0 | 1 | 0 | 2 | 0 | 0 | 0 | 0 |
| *Lamontichthys filamentosus* | 0 | 1 | ? | 0 | 0 | 1 | 0 | 1 | 2 | 1 | 0 | 3 | 1 | 2 | 1 | 0 | 1 | 0 | 1 | 2 | 1 | 0 | 0 | 0 | 0 |
| *Lamontichthys llanero* | 0 | 1 | 0 | ? | 0 | 0 | 0 | 1 | 2 | 0 | 0 | 3 | 1 | 2 | 1 | 1 | 0 | 0 | 1 | ? | 2 | 0 | 0 | 0 | 0 |

| **Taxon/Character** | **100** | **101** | **102** | **103** | **104** | **105** | **106** | **107** | **108** | **109** | **110** | **111** | **112** | **113** | **114** | **115** | **116** | **117** | **118** | **119** | **120** | **121** | **122** | **123** |
|---|---|---|---|---|---|---|---|---|---|---|---|---|---|---|---|---|---|---|---|---|---|---|---|---|
| ***Farlowella smithi*** | 0 | 0 | 1 | 3 | 1 | 2 | 0 | 2 | 1 | 1 | 0 | 0 | 0 | 0 | 1 | 0 | 0 | 2 | 3 | 0 | 2 | 1 | 3 | 0 |
| ***Farlowella venezuelensis*** | 0 | 0 | 1 | 1 | 1 | 2 | 0 | 2 | 1 | 1 | 0 | 0 | 0 | 1 | 1 | 0 | 0 | 2 | 3 | 0 | 2 | 1 | 2 | 0 |
| ***Farlowella vittata*** | 1 | - | 1 | 2 | 1 | 2 | 0 | 2 | 1 | 1 | 0 | 0 | 0 | 0 | 1 | 0 | 0 | 2 | 3 | 0 | 2 | 1 | 1 | 0 |
| ***Harttia carvalhoi*** | 1 | - | 1 | 4 | 2 | 0 | 0 | 1 | 1 | 0 | 1 | 0 | 1 | 0 | 2 | 1 | 0 | 1 | 0 | 0 | 2 | 0 | 1 | 0 |
| ***Harttia dissidens*** | 0 | 1 | 1 | 4 | 2 | 0 | 0 | 1 | 1 | 0 | 1 | 0 | 1 | 0 | 2 | 1 | 0 | 1 | 3 | 0 | 1 | 0 | 1 | 0 |
| ***Harttia duriventris*** | 0 | 1 | 1 | 4 | 2 | 0 | 0 | 1 | 1 | 0 | 1 | 0 | 1 | 0 | 2 | 1 | 0 | 0 | 0 | 0 | 1 | 0 | 1 | 0 |
| ***Harttia fluminensis*** | 0 | 1 | 1 | 4 | 2 | 0 | 0 | 1 | 1 | 0 | 1 | 0 | 1 | 0 | 2 | 1 | 0 | 1 | 0 | 0 | 2 | 0 | 1 | 0 |
| ***Harttia fowleri*** | 0 | 1 | 1 | 4 | 2 | 0 | 0 | 1 | 1 | 0 | 1 | 0 | 1 | 0 | 2 | 1 | 0 | 1 | 0 | 0 | 1 | 0 | 1 | 0 |
| ***Harttia garavelloi*** | 0 | 0 | 1 | 4 | 2 | 0 | 0 | 1 | 1 | 0 | 1 | 0 | 1 | 0 | 2 | 1 | 0 | 0 | 3 | 0 | 2 | 0 | 1 | 0 |
| ***Harttia gracilis*** | 1 | - | 1 | 4 | 2 | 0 | 0 | 1 | 1 | 0 | 1 | 0 | 1 | 0 | 0 | 1 | 0 | 1 | 3 | 0 | 1 | 0 | 1 | 0 |
| ***Harttia guianensis*** | 0 | 0 | 1 | 4 | 2 | 0 | 0 | 1 | 1 | 0 | 1 | 0 | 1 | 0 | 2 | 1 | 0 | 1 | 0 | 0 | 2 | 0 | 1 | 0 |
| ***Harttia kronei*** | 0 | 1 | 1 | 4 | 2 | 0 | 0 | 1 | 1 | 0 | 1 | 0 | 1 | 0 | 2 | 1 | 0 | 1 | 0 | 0 | 2 | 0 | 1 | 0 |
| ***Harttia leiopleura*** | 0 | 1 | 1 | 4 | 2 | 0 | 0 | 1 | 1 | 0 | 1 | 0 | 1 | 0 | 0 | 0 | 0 | 1 | 3 | 0 | 2 | 0 | 1 | 0 |
| ***Harttia longipinna*** | 0 | 0 | 1 | 4 | 2 | 0 | 0 | 1 | 1 | 0 | 1 | 0 | 1 | 0 | 0 | 1 | 0 | 0 | 3 | 0 | 2 | 0 | 1 | 0 |
| ***Harttia loricariformis*** | 0 | 1 | 1 | 4 | 2 | 0 | 0 | 1 | 1 | 0 | 1 | 0 | 1 | 0 | 0 | 1 | 0 | 1 | 3 | 0 | 2 | 0 | 1 | 0 |
| ***Harttia novalimensis*** | 0 | 1 | 1 | 4 | 2 | 0 | 0 | 1 | 1 | 0 | 1 | 0 | 1 | 0 | 0 | 1 | 0 | 0 | 3 | 0 | 2 | 0 | 1 | 0 |
| ***Harttia punctata*** | 0 | 1 | 1 | 4 | 2 | 0 | 0 | 1 | 1 | 0 | 1 | 0 | 1 | 0 | 2 | 1 | 0 | 0 | 0 | 0 | 1 | 0 | 1 | 0 |
| ***Harttia rhombocephala*** | 0 | 1 | 1 | 4 | 2 | 0 | 0 | 1 | 1 | 0 | 1 | 0 | 1 | 0 | 0 | 0 | 0 | 0 | 3 | 0 | 1 | 0 | 1 | 0 |
| ***Harttia torrenticola*** | 0 | 0 | 1 | 4 | 2 | 0 | 0 | 1 | 1 | 0 | 1 | 0 | 1 | 0 | 2 | 1 | 0 | 0 | 3 | 0 | 2 | 0 | 1 | 0 |
| ***Harttia trombetensis*** | 0 | 1 | 1 | 4 | 2 | 0 | 0 | 1 | 1 | 0 | 1 | 0 | 1 | 0 | 2 | 1 | 0 | 0 | 0 | 0 | 2 | 0 | 1 | 0 |
| ***Harttiella crassicauda*** | 0 | 0 | 1 | 0 | 2 | 0 | 0 | 1 | 1 | 0 | 1 | 0 | 0 | 1 | 2 | 0 | 0 | 0 | 3 | 0 | 2 | 1 | 1 | 0 |
| ***Harttiella longicauda*** | 0 | 0 | 0 | 0 | 2 | 0 | 0 | 1 | 1 | 0 | 1 | 0 | 0 | 1 | 2 | 0 | 0 | 0 | 3 | 0 | 2 | 1 | 1 | 0 |
| ***Lamontichthys avacanoeiro*** | 0 | 2 | 0 | 0 | 1 | 1 | 0 | 2 | 1 | 0 | 1 | 0 | 0 | 0 | 2 | 1 | 1 | 1 | 0 | 0 | 1 | 0 | 1 | 1 |
| ***Lamontichthys filamentosus*** | 0 | 1 | 0 | 0 | 1 | 1 | 0 | 2 | 0 | 0 | 1 | 0 | 1 | 0 | 2 | 1 | 1 | 0 | 0 | 0 | 1 | 0 | 1 | 1 |
| ***Lamontichthys llanero*** | 0 | 2 | 0 | 0 | 1 | 1 | 0 | 2 | 0 | 0 | 1 | 0 | 1 | 0 | 2 | 1 | 1 | 0 | 0 | 0 | 1 | 0 | 1 | 1 |

| **Taxon/Character** | **124** | **125** | **126** | **127** | **128** | **129** | **130** | **131** | **132** | **133** | **134** | **135** | **136** | **137** | **138** | **139** | **140** | **141** | **142** | **143** | **144** | **145** | **146** | **147** |
|---|---|---|---|---|---|---|---|---|---|---|---|---|---|---|---|---|---|---|---|---|---|---|---|---|
| ***Farlowella smithi*** | 1 | 3 | 1 | 1 | 2 | 1 | 1 | 1 | 0 | 1 | 1 | 1 | 1 | 0 | 0 | 0 | 1 | 0 | 1 | 0 | 1 | 1 | 0 | 0 |
| ***Farlowella venezuelensis*** | 1 | 3 | 0 | 0 | 3 | 1 | 1 | 1 | 0 | 1 | 1 | 1 | 1 | 0 | 0 | 0 | 0 | 0 | ? | 0 | 1 | 1 | 0 | 0 |
| ***Farlowella vittata*** | 1 | 3 | 0 | 0 | 2 | 1 | 1 | 1 | 0 | 1 | 1 | 1 | 1 | 0 | 0 | 0 | 0 | 0 | 1 | 0 | 1 | 1 | 0 | 0 |
| ***Harttia carvalhoi*** | 0 | 3 | 1 | 1 | 3 | 0 | 2 | 1 | 0 | 1 | 0 | 1 | 1 | 0 | 1 | 0 | 1 | 0 | 2 | 1 | 0 | 0 | 0 | 0 |
| ***Harttia dissidens*** | 0 | 3 | 1 | 1 | 3 | 0 | 2 | 1 | 0 | 0 | 0 | 1 | 1 | 0 | 1 | 0 | 1 | 0 | 2 | 1 | 0 | 0 | 0 | 0 |
| ***Harttia duriventris*** | 0 | 3 | 1 | 1 | 2 | 0 | 2 | 1 | 0 | 1 | 0 | 1 | 1 | 0 | 1 | 0 | 1 | 0 | 1 | 1 | 0 | 0 | 1 | 0 |
| ***Harttia fluminensis*** | 0 | 3 | 0 | 0 | 2 | 0 | 2 | 1 | 1 | 0 | 0 | 1 | 1 | 0 | 1 | 0 | 1 | 0 | 1 | 1 | 0 | 0 | 0 | 0 |
| ***Harttia fowleri*** | 0 | 3 | 0 | 0 | 3 | 0 | 2 | 1 | 0 | 0 | 0 | 1 | 1 | 0 | 1 | 0 | 1 | 0 | 1 | 1 | 0 | 0 | 0 | 0 |
| ***Harttia garavelloi*** | 0 | 3 | 1 | 1 | 1 | 0 | 2 | 1 | 1 | 1 | 0 | 1 | 1 | 0 | 1 | 0 | 1 | 0 | 2 | 1 | 0 | 0 | 1 | 0 |
| ***Harttia gracilis*** | 0 | 3 | 1 | 1 | 2 | 0 | 2 | 1 | 0 | 1 | 0 | 1 | 1 | 0 | 1 | 0 | 1 | 0 | 2 | 1 | 0 | 0 | 0 | 0 |
| ***Harttia guianensis*** | 0 | 3 | 1 | 1 | 2 | 0 | 1 | 1 | 0 | 0 | 0 | 1 | 1 | 0 | 1 | 0 | 1 | 0 | ? | 1 | 0 | 0 | 0 | 0 |
| ***Harttia kronei*** | 0 | 3 | 1 | 1 | 3 | 0 | 1 | 1 | 1 | 0 | 0 | 1 | 1 | 0 | 1 | 0 | 1 | 0 | 1 | 1 | 0 | 0 | 1 | 0 |
| ***Harttia leiopleura*** | 0 | 3 | 1 | 1 | 3 | 0 | 2 | 1 | 0 | 1 | 0 | 1 | 1 | 0 | 1 | 0 | 1 | 0 | 1 | 0 | 0 | 0 | 0 | 0 |
| ***Harttia longipinna*** | 0 | 3 | 1 | 1 | 2 | 0 | 2 | 1 | 1 | 1 | 0 | 1 | 1 | 0 | 1 | 0 | 1 | 0 | 1 | 0 | 0 | 0 | 0 | 0 |
| ***Harttia loricariformis*** | 0 | 3 | 1 | 1 | 2 | 0 | 1 | 1 | 1 | 1 | 0 | 1 | 1 | 0 | 1 | 0 | 1 | 0 | 2 | 1 | 0 | 0 | 1 | 0 |
| ***Harttia novalimensis*** | 0 | 3 | 1 | 1 | 2 | 0 | 2 | 1 | 0 | 0 | 0 | 1 | 1 | 0 | 1 | 0 | 1 | 0 | 2 | 1 | 0 | 0 | 0 | 0 |
| ***Harttia punctata*** | 0 | 1 | 1 | 1 | 2 | 0 | 1 | 1 | 0 | 0 | 0 | 0 | 1 | 0 | 1 | 0 | 1 | 0 | 1 | 1 | 0 | 0 | 0 | 0 |
| ***Harttia rhombocephala*** | 0 | 3 | 1 | 1 | 2 | 0 | 2 | 1 | 0 | 1 | 0 | 1 | 1 | 0 | 1 | 0 | 1 | 0 | 1 | 1 | 0 | 0 | 1 | 0 |
| ***Harttia torrenticola*** | 0 | 3 | 1 | 1 | 3 | 0 | 1 | 1 | 0 | 1 | 0 | 1 | 1 | 0 | 1 | 0 | 1 | 0 | ? | 0 | 0 | 0 | 1 | 0 |
| ***Harttia trombetensis*** | 0 | 3 | 1 | 1 | 2 | 0 | 1 | 1 | 0 | 0 | 0 | 1 | 1 | 0 | 1 | 0 | 1 | 0 | 1 | 1 | 0 | 0 | 1 | 0 |
| ***Harttiella crassicauda*** | 0 | 3 | 0 | 0 | 3 | 0 | 2 | 1 | 1 | 0 | 1 | 1 | 1 | 0 | 1 | 0 | 1 | 0 | 1 | 1 | 0 | 0 | 0 | 1 |
| ***Harttiella longicauda*** | 0 | 3 | 0 | 0 | 3 | 0 | 1 | 1 | 0 | 0 | 1 | 1 | 1 | 0 | 1 | 0 | 1 | 0 | 1 | 1 | 0 | 0 | 1 | 1 |
| ***Lamontichthys avacanoeiro*** | 1 | 1 | 0 | 0 | 2 | 0 | 2 | 1 | 0 | 1 | 1 | 1 | 1 | 1 | 0 | 0 | 0 | 0 | 1 | 1 | 1 | 1 | 1 | 0 |
| ***Lamontichthys filamentosus*** | 1 | 1 | 0 | 0 | 3 | 0 | 2 | 1 | 0 | 1 | 1 | 1 | 1 | 1 | 0 | 0 | 0 | 0 | 0 | 1 | 1 | 1 | 1 | 0 |
| ***Lamontichthys llanero*** | 1 | 3 | 0 | 0 | 3 | 0 | 2 | 1 | 0 | 1 | 1 | 1 | 1 | 1 | 0 | 0 | 0 | 0 | 1 | 1 | 1 | 1 | 1 | 0 |

| **Taxon/Character** | **148** | **149** | **150** | **151** | **152** | **153** | **154** | **155** | **156** | **157** | **158** | **159** | **160** | **161** | **162** | **163** | **164** | **165** | **166** | **167** | **168** | **169** | **170** | **171** |
|---|---|---|---|---|---|---|---|---|---|---|---|---|---|---|---|---|---|---|---|---|---|---|---|---|
| ***Farlowella smithi*** | 1 | 0 | 1 | 0 | 0 | 0 | 2 | 1 | 0 | 1 | 1 | 1 | 0 | 1 | 0 | 2 | 0 | 1 | 1 | 2 | 2 | 1 | 0 | 1 |
| ***Farlowella venezuelensis*** | 1 | 1 | 2 | 0 | 0 | 0 | 2 | 1 | 0 | 2 | 1 | 1 | 0 | 0 | 1 | 2 | 0 | 1 | 1 | 0 | 2 | 1 | 0 | 1 |
| ***Farlowella vittata*** | 1 | 2 | 2 | 0 | 0 | 0 | 2 | 1 | 0 | 1 | 1 | 2 | 0 | 0 | 0 | 2 | 2 | 1 | 1 | 0 | 2 | 1 | 0 | 1 |
| ***Harttia carvalhoi*** | 2 | 0 | 1 | 1 | 1 | 0 | 1 | 0 | 0 | 0 | 1 | 1 | 2 | 1 | 0 | 2 | 2 | 1 | 0 | 0 | 0 | 0 | 0 | 0 |
| ***Harttia dissidens*** | 2 | 0 | 1 | 1 | 1 | 0 | 2 | 3 | 0 | 0 | 1 | 1 | 2 | 0 | 0 | 2 | 2 | 0 | 2 | 0 | 0 | 0 | 0 | 0 |
| ***Harttia duriventris*** | 2 | 0 | 1 | 1 | 1 | 0 | 2 | 0 | 0 | 0 | 1 | 1 | 2 | 0 | 0 | 2 | 2 | 0 | 2 | 1 | 0 | 0 | 0 | 0 |
| ***Harttia fluminensis*** | 2 | 0 | 1 | 1 | 1 | 0 | 1 | 3 | 1 | - | 1 | 1 | 2 | 1 | 0 | 2 | 2 | 0 | 0 | 0 | 0 | 0 | 0 | 0 |
| ***Harttia fowleri*** | 2 | 0 | 1 | 1 | 1 | 0 | 1 | 3 | 1 | - | 1 | 1 | 2 | 0 | 0 | 2 | 2 | 0 | 0 | 1 | 0 | 0 | 0 | 0 |
| ***Harttia garavelloi*** | 2 | 0 | 1 | 1 | 1 | 0 | 1 | 3 | 0 | 0 | 1 | 1 | 2 | 1 | 0 | 2 | 2 | 0 | 0 | 1 | 0 | 0 | 0 | 0 |
| ***Harttia gracilis*** | 2 | 0 | 1 | 1 | 1 | 0 | 1 | 3 | 0 | 0 | 1 | 1 | 2 | 0 | 0 | 2 | 2 | 0 | 0 | 1 | 0 | 0 | 0 | 0 |
| ***Harttia guianensis*** | 2 | 0 | 1 | 1 | 1 | 0 | 1 | 0 | 1 | - | 1 | 1 | 2 | 0 | 0 | 2 | 2 | 0 | 2 | 1 | 0 | 0 | 0 | 0 |
| ***Harttia kronei*** | 2 | 0 | 1 | 1 | 1 | 0 | 1 | 0 | 0 | 0 | 1 | 1 | 2 | 1 | 0 | 2 | 2 | 0 | 2 | 0 | 0 | 0 | 0 | 0 |
| ***Harttia leiopleura*** | 2 | 0 | 1 | 1 | 1 | 0 | 1 | 3 | 0 | 0 | 1 | 1 | 2 | 1 | 0 | 2 | 2 | 1 | 0 | 1 | 0 | 0 | 0 | 0 |
| ***Harttia longipinna*** | 2 | 0 | 1 | 1 | 1 | 0 | 2 | 0 | 0 | 0 | 1 | 1 | 2 | 1 | 0 | 2 | 2 | 0 | 2 | 0 | 0 | 0 | 0 | 0 |
| ***Harttia loricariformis*** | 2 | 0 | 1 | 1 | 1 | 0 | 1 | 3 | 0 | 0 | 1 | 1 | 2 | 1 | 0 | 2 | 2 | 0 | 0 | 1 | 0 | 0 | 0 | 0 |
| ***Harttia novalimensis*** | 2 | 0 | 1 | 1 | 1 | 0 | 2 | 0 | 0 | 0 | 1 | 1 | 2 | 1 | 0 | 2 | 2 | 0 | 2 | 1 | 0 | 0 | 0 | 0 |
| ***Harttia punctata*** | 2 | 0 | 1 | 1 | 1 | 0 | 1 | 3 | 0 | 2 | 1 | 1 | 2 | 1 | 0 | 2 | 2 | 0 | 0 | 1 | 0 | 0 | 0 | 0 |
| ***Harttia rhombocephala*** | 2 | 0 | 1 | 1 | 1 | 0 | 1 | 3 | 0 | 0 | 1 | 1 | 2 | 0 | 0 | 2 | 2 | 1 | 0 | 1 | 0 | 0 | 0 | 0 |
| ***Harttia torrenticola*** | 2 | 0 | 1 | 1 | 1 | 0 | 1 | 3 | 0 | 0 | 1 | 1 | 2 | 1 | 0 | 2 | 2 | 1 | 0 | 0 | 0 | 0 | ? | ? |
| ***Harttia trombetensis*** | 2 | 0 | 1 | 1 | 1 | 0 | 1 | 0 | 1 | - | 1 | 1 | 2 | 1 | 0 | 2 | 2 | 0 | 0 | 1 | 0 | 0 | ? | ? |
| ***Harttiella crassicauda*** | 1 | 0 | 1 | 1 | 1 | 0 | 2 | 0 | 0 | 0 | 1 | 0 | 2 | 2 | 0 | 2 | 2 | 0 | 1 | 0 | 0 | 0 | 0 | 0 |
| ***Harttiella longicauda*** | 1 | 0 | 1 | 0 | 1 | 0 | 2 | 0 | 0 | 0 | 1 | 0 | 2 | 0 | 0 | 2 | 2 | 0 | 1 | 1 | 0 | 0 | 0 | 0 |
| ***Lamontichthys avacanoeiro*** | 2 | 0 | 2 | 0 | 0 | 0 | 2 | 0 | 0 | 0 | 1 | 1 | 2 | 1 | 1 | 2 | 1 | 0 | 1 | 1 | 0 | 2 | 1 | 1 |
| ***Lamontichthys filamentosus*** | 2 | 0 | 2 | 0 | 0 | 0 | 2 | 0 | 0 | 0 | 1 | 1 | 2 | 0 | 0 | 2 | 1 | 0 | 1 | 0 | 0 | 0 | 0 | 1 |
| ***Lamontichthys llanero*** | 2 | 0 | 2 | 0 | 0 | 0 | 2 | 0 | 0 | 0 | 1 | 1 | 2 | 1 | 1 | 2 | 1 | 0 | 1 | 1 | 0 | 0 | 0 | 1 |

| Taxon/Character | 172 | 173 | 174 | 175 | 176 | 177 | 178 | 179 | 180 | 181 | 182 | 183 | 184 | 185 | 186 | 187 | 188 | 189 | 190 | 191 | 192 | 193 | 194 | 195 | 196 |
|---|---|---|---|---|---|---|---|---|---|---|---|---|---|---|---|---|---|---|---|---|---|---|---|---|---|
| ***Farlowella smithi*** | 3 | 1 | 1 | 1 | 3 | 3 | 1 | 1 | 3 | 1 | 2 | 0 | 0 | 0 | 1 | 1 | 1 | 0 | 1 | 2 | 1 | 2 | 0 | 1 | 1 |
| ***Farlowella venezuelensis*** | 0 | 1 | 1 | 1 | 3 | 3 | 1 | 1 | 3 | - | 2 | 0 | 0 | 0 | 1 | 1 | 1 | 0 | 1 | 3 | 0 | 2 | 0 | 1 | 1 |
| ***Farlowella vittata*** | 0 | 1 | 1 | 1 | 3 | 3 | 1 | 1 | 3 | - | 2 | 0 | 0 | 0 | 1 | 1 | 1 | 0 | 1 | 3 | 1 | 2 | 0 | 1 | 1 |
| ***Harttia carvalhoi*** | 0 | 0 | 0 | 0 | 3 | 0 | 0 | 0 | - | - | 1 | 0 | 0 | 1 | 0 | 0 | 1 | 0 | 1 | 1 | 0 | 1 | 0 | 0 | 0 |
| ***Harttia dissidens*** | 0 | 0 | 0 | 0 | 2 | 0 | 1 | 0 | 1 | 0 | 2 | 0 | 0 | 1 | 0 | 1 | 1 | 1 | 1 | 1 | 0 | 1 | 0 | 1 | 0 |
| ***Harttia duriventris*** | 0 | 0 | 0 | 0 | 2 | 0 | 1 | 0 | 1 | 0 | 2 | 0 | 0 | 0 | 0 | 1 | 1 | 1 | 1 | 1 | 0 | 1 | 0 | 1 | 0 |
| ***Harttia fluminensis*** | 0 | 0 | 0 | 0 | 2 | 0 | 0 | 0 | - | - | 2 | 0 | 0 | 0 | 0 | 1 | 1 | 1 | 1 | 1 | 0 | 1 | 0 | 0 | 0 |
| ***Harttia fowleri*** | 0 | 0 | 0 | 0 | 2 | 1 | 1 | 0 | 1 | 0 | 2 | 0 | 0 | 1 | 0 | 1 | 1 | 1 | 1 | 1 | 0 | 1 | 0 | 0 | 0 |
| ***Harttia garavelloi*** | 3 | 0 | 0 | 0 | 2 | 2 | 0 | 0 | - | - | 2 | 0 | 0 | 1 | 2 | 0 | 1 | 1 | 1 | 1 | 0 | 1 | 0 | 0 | 0 |
| ***Harttia gracilis*** | 3 | 0 | 0 | 0 | 2 | 0 | 0 | 0 | - | - | 2 | 0 | 0 | 0 | 0 | 1 | 1 | 1 | 1 | 1 | 0 | 1 | 0 | 1 | 0 |
| ***Harttia guianensis*** | 0 | 0 | 0 | 0 | 2 | 2 | 0 | 0 | - | - | 2 | 0 | 0 | 0 | 0 | 1 | 1 | 1 | 1 | 1 | 0 | 1 | 0 | 0 | 0 |
| ***Harttia kronei*** | 3 | 0 | 0 | 0 | 3 | 2 | 0 | 0 | - | - | 1 | 0 | 0 | 0 | 0 | 0 | 1 | 0 | 1 | 1 | 0 | 1 | 0 | 0 | 0 |
| ***Harttia leiopleura*** | 3 | 0 | 0 | 0 | 2 | 0 | 0 | 0 | - | - | 2 | 0 | 0 | 0 | 0 | 0 | 1 | 0 | 1 | 1 | 0 | 1 | 0 | 0 | 0 |
| ***Harttia longipinna*** | 0 | 0 | 0 | 0 | 2 | 2 | 1 | 0 | 1 | 0 | 2 | 0 | 0 | 0 | 0 | 1 | 1 | 1 | 1 | 1 | 0 | 1 | 0 | 1 | 0 |
| ***Harttia loricariformis*** | 3 | 0 | 0 | 0 | 2 | 2 | 0 | 0 | - | - | 2 | 0 | 0 | 0 | 0 | 1 | 1 | 1 | 1 | 1 | 0 | 1 | 0 | 0 | 0 |
| ***Harttia novalimensis*** | 3 | 0 | 0 | 0 | 2 | 2 | 0 | 0 | - | - | 2 | 0 | 0 | 0 | 0 | 0 | 1 | 1 | 1 | 1 | 0 | 1 | 0 | 0 | 0 |
| ***Harttia punctata*** | 0 | 0 | 0 | 0 | 2 | 0 | 0 | 0 | - | - | 2 | 0 | 0 | 2 | 0 | 1 | 1 | 1 | 1 | 1 | 0 | 1 | 0 | 1 | 0 |
| ***Harttia rhombocephala*** | 0 | 0 | 0 | 0 | 2 | 0 | 1 | 0 | 1 | 0 | 2 | 0 | 0 | 0 | 0 | 1 | 1 | 1 | 1 | 1 | 0 | 1 | 0 | 1 | 0 |
| ***Harttia torrenticola*** | ? | ? | 0 | 0 | 2 | ? | 0 | 0 | - | - | ? | 0 | 0 | 0 | 0 | 1 | 1 | 0 | 1 | 1 | 0 | 1 | 0 | 0 | 0 |
| ***Harttia trombetensis*** | ? | ? | 0 | 0 | 2 | 0 | 1 | 0 | 0 | 0 | 2 | 0 | 0 | ? | ? | 1 | 1 | 1 | 1 | 1 | 0 | 1 | 0 | 0 | 0 |
| ***Harttiella crassicauda*** | 0 | 0 | 0 | 0 | 2 | 2 | 0 | 0 | - | - | 1 | 0 | 0 | 1 | 2 | 0 | 1 | 0 | 1 | 1 | 0 | 1 | 0 | 0 | 0 |
| ***Harttiella longicauda*** | 0 | 0 | 0 | 0 | 3 | 0 | 0 | 0 | - | - | 1 | 0 | 0 | 1 | 1 | 1 | 1 | 0 | 1 | 1 | 0 | 1 | 0 | 0 | 0 |
| ***Lamontichthys avacanoeiro*** | 0 | 0 | 1 | 1 | 3 | 1 | 1 | 1 | 1 | 0 | 2 | 0 | 0 | 2 | 2 | 1 | 1 | 0 | 1 | 1 | 0 | 1 | 0 | 2 | 1 |
| ***Lamontichthys filamentosus*** | 0 | 0 | 1 | 1 | 2 | 1 | 1 | 1 | 1 | 0 | 2 | 1 | 0 | 2 | 0 | 1 | 1 | 0 | 1 | 1 | 2 | 1 | 0 | 2 | 1 |
| ***Lamontichthys llanero*** | 0 | 0 | 1 | 1 | 2 | 1 | 1 | 1 | 1 | 0 | 2 | 1 | 0 | 2 | 0 | 1 | 1 | 0 | 1 | 1 | 2 | 1 | 0 | 2 | 1 |

| **Taxon/Character** | **1** | **2** | **3** | **4** | **5** | **6** | **7** | **8** | **9** | **10** | **11** | **12** | **13** | **14** | **15** | **16** | **17** | **18** | **19** | **20** | **21** | **22** | **23** | **24** | **25** |
|---|---|---|---|---|---|---|---|---|---|---|---|---|---|---|---|---|---|---|---|---|---|---|---|---|---|
| ***Lamontichthys parakana*** | 0 | 0 | 0 | 0 | 1 | 1 | 0 | 2 | 0 | 1 | 1 | 1 | 1 | 0 | 0 | 0 | 0 | 1 | 3 | 0 | 1 | 1 | 0 | 0 | 1 |
| ***Metaloricaria nijsseni*** | 0 | 1 | 0 | 0 | 3 | 0 | 3 | 2 | 0 | 1 | 0 | 0 | 4 | 1 | 0 | 0 | 0 | 1 | 3 | 0 | 1 | 2 | 1 | 0 | 0 |
| ***Metaloricaria paucidens*** | 0 | 1 | 0 | 0 | 3 | 0 | 3 | 2 | 0 | 1 | 0 | 0 | 4 | 1 | 0 | 0 | 0 | 1 | 3 | 0 | 1 | 2 | 1 | 0 | 0 |
| ***Pterosturisoma microps*** | 1 | 0 | 1 | 1 | 1 | 1 | 0 | 1 | 0 | 1 | 0 | 3 | 4 | 0 | 0 | 0 | 0 | 1 | 3 | 1 | 2 | - | 1 | 0 | 1 |
| ***Sturisoma barbatum*** | 1 | 0 | 0 | 0 | 2 | 2 | 2 | 0 | 0 | 1 | 1 | 1 | 1 | 0 | 0 | 1 | 1 | 1 | 3 | 0 | 1 | 2 | 0 | 0 | 1 |
| ***Sturisoma guentheri*** | 1 | 0 | 1 | 1 | 2 | 2 | 2 | 1 | 0 | 1 | 1 | 3 | 1 | 0 | 0 | 1 | 1 | 0 | 2 | 0 | 1 | 2 | 0 | 0 | 1 |
| ***Sturisoma lyra*** | 1 | 0 | 1 | 1 | 2 | 2 | 2 | 0 | 0 | 1 | 0 | 3 | 1 | 0 | 0 | 1 | 1 | 1 | 3 | 1 | 1 | 2 | 0 | 0 | 1 |
| ***Sturisoma monopelte*** | 1 | 0 | 0 | 1 | 2 | 2 | 2 | 0 | 0 | 1 | 1 | 3 | 1 | 0 | 0 | 1 | 1 | 1 | 3 | 1 | 1 | 1 | 1 | 0 | 1 |
| ***Sturisoma nigrirostrum*** | 1 | 0 | 0 | 1 | 2 | 2 | 2 | 0 | 0 | 1 | 1 | 1 | 1 | 0 | 0 | 1 | 1 | 1 | 3 | 0 | 1 | 1 | 0 | 0 | 1 |
| ***Sturisoma robustum*** | 1 | 0 | 0 | 1 | 2 | 2 | 2 | 0 | 0 | 1 | 1 | 3 | 4 | 0 | 0 | 1 | 1 | 0 | 3 | 0 | 1 | 1 | 1 | 0 | 1 |
| ***Sturisoma rostratum*** | 1 | 0 | 0 | 0 | 2 | 2 | 2 | 0 | 0 | 1 | 1 | 3 | 1 | 0 | 0 | 1 | 1 | 0 | 3 | 0 | ? | ? | 0 | 0 | 1 |
| ***Sturisoma* aff. *tenuirostre*** | 1 | 0 | 1 | 1 | 2 | 2 | 2 | 0 | 0 | 1 | 0 | 1 | 1 | 0 | 0 | 1 | 1 | 0 | 3 | 0 | 1 | 2 | 1 | 0 | 1 |
| ***Sturisoma graffini*** | 1 | 0 | 1 | 1 | 2 | 2 | 2 | 1 | 0 | 1 | 1 | 3 | 1 | 0 | 0 | 1 | 1 | 0 | 2 | 0 | 1 | 1 | 1 | 0 | 1 |
| ***Sturisomatichthys aureus*** | 1 | 0 | 0 | 1 | 1 | 2 | 2 | 0 | 0 | 1 | 0 | 3 | 2 | 0 | 0 | 0 | 0 | 1 | 3 | 0 | 1 | 1 | 0 | 1 | 1 |
| ***Sturisomatichthys citurensis*** | 0 | 0 | ? | 1 | 1 | 2 | 1 | 2 | 0 | 1 | 0 | 3 | 2 | 0 | 0 | 0 | 0 | 0 | 2 | 0 | 1 | 1 | 0 | 1 | 1 |
| ***Sturisomatichthys dariensis*** | 1 | 0 | 0 | 1 | 1 | 2 | 1 | 0 | 0 | 1 | 0 | 3 | 2 | 0 | 0 | 1 | 1 | 0 | 1 | 0 | 1 | 2 | 0 | 1 | 1 |
| ***Sturisomatichthys festivus*** | 1 | 0 | 0 | 0 | 1 | 2 | 1 | 0 | 0 | 1 | 0 | 3 | 2 | 0 | 0 | 1 | 1 | 0 | 1 | 1 | 1 | 2 | 0 | 0 | 1 |
| ***Sturisomatichthys frenatus*** | 1 | 0 | 0 | 1 | 1 | 2 | 1 | 0 | 0 | 1 | 0 | 3 | 2 | 0 | 0 | 1 | 1 | 0 | 2 | 0 | 1 | 1 | 0 | 0 | 1 |
| ***Sturisomatichthys kneri*** | 1 | 0 | 1 | 0 | 2 | 2 | 2 | 0 | 0 | 1 | 0 | 3 | 2 | 0 | 0 | 1 | 1 | 0 | 1 | 1 | 1 | 1 | 1 | 0 | 1 |
| ***Sturisomatichthys leightoni*** | 1 | 0 | 0 | 1 | 1 | 2 | 1 | 1 | 0 | 1 | 0 | 3 | 2 | 0 | 0 | 0 | 0 | 0 | 0 | 0 | 1 | 1 | 0 | 0 | 1 |
| ***Sturisomatichthys panamensis*** | 1 | 0 | 0 | 1 | 1 | 2 | 1 | 0 | 0 | 1 | 0 | 1 | 2 | 0 | 0 | 1 | 1 | 1 | 2 | 1 | 1 | 2 | 0 | 0 | 1 |
| ***Sturisomatichthys tamanae*** | 0 | 0 | ? | 1 | 1 | 2 | 1 | 2 | 0 | 1 | 0 | 3 | 2 | 0 | 0 | 0 | 0 | 0 | 2 | 0 | 1 | 1 | 0 | 0 | 1 |
| ***Sturisomatichthys varii*** | 1 | 0 | 1 | 1 | 1 | 2 | 1 | 0 | 0 | 1 | 0 | 1 | 2 | 0 | 0 | 1 | 1 | 0 | 1 | 0 | 1 | 1 | 0 | 0 | 1 |
| ***Sturisomatichthys reinae*** | 1 | 0 | ? | 1 | 1 | 2 | 1 | 0 | 0 | 1 | 0 | 1 | 2 | 0 | 0 | 0 | 0 | 0 | 1 | 1 | 1 | 1 | 0 | 0 | 1 |

| Taxon/Character | 27 | 28 | 29 | 30 | 31 | 32 | 33 | 34 | 35 | 36 | 37 | 38 | 39 | 40 | 41 | 42 | 43 | 44 | 45 | 46 | 47 | 48 | 49 | 50 |
|---|---|---|---|---|---|---|---|---|---|---|---|---|---|---|---|---|---|---|---|---|---|---|---|---|
| *Lamontichthys parakana* | 0 | 0 | 1 | 0 | 0 | 2 | 3 | 2 | 1 | 0 | 0 | 4 | 1 | 1 | 0 | 2 | 0 | 0 | 1 | 1 | 1 | 0 | 2 | 0 |
| *Metaloricaria nijsseni* | 0 | 0 | 1 | 1 | 0 | 0 | 0 | 1 | 0 | 0 | 0 | 4 | 0 | 2 | 2 | 1 | 0 | 1 | 1 | 2 | ? | 1 | 2 | 1 |
| *Metaloricaria paucidens* | 0 | 0 | 1 | 1 | 0 | 0 | 0 | 1 | 0 | 0 | 0 | 4 | 0 | 2 | 2 | 1 | 0 | 1 | 1 | 2 | ? | 1 | 2 | 1 |
| *Pterosturisoma microps* | 0 | 0 | 2 | 1 | 0 | 0 | 1 | 2 | 1 | 0 | 0 | 3 | 0 | 1 | 1 | 2 | 1 | 0 | 0 | 1 | 1 | 0 | 2 | 0 |
| *Sturisoma barbatum* | 1 | 0 | 1 | 1 | 1 | 0 | 0 | 2 | 0 | 0 | 0 | 4 | 0 | 2 | 1 | 2 | 1 | 0 | 1 | 1 | 2 | 0 | 2 | 0 |
| *Sturisoma guentheri* | 1 | 1 | 0 | 1 | 1 | 0 | 1 | 2 | 1 | 0 | 1 | 3 | 1 | 2 | 1 | 2 | 1 | 0 | 0 | 0 | 2 | 0 | 2 | 0 |
| *Sturisoma lyra* | 1 | 1 | 0 | 1 | 1 | 0 | 0 | 2 | 0 | 0 | 1 | 4 | 0 | 2 | 1 | 0 | 1 | 0 | 1 | 1 | 3 | 0 | 2 | 0 |
| *Sturisoma monopelte* | 1 | 1 | 0 | 1 | 1 | 0 | 0 | 2 | 0 | 0 | 0 | 3 | 1 | 2 | 1 | 2 | 1 | 0 | 0 | 0 | 1 | 0 | 2 | 0 |
| *Sturisoma nigrirostrum* | 0 | 1 | 0 | 0 | 1 | 0 | 2 | 2 | 1 | 0 | 0 | 3 | 1 | 2 | 1 | 2 | 1 | 0 | 0 | 1 | 2 | 0 | 0 | 0 |
| *Sturisoma robustum* | 1 | 1 | 0 | 1 | 1 | 0 | 1 | 2 | 0 | 0 | 1 | 3 | 1 | 2 | 1 | 0 | 1 | 0 | 0 | 0 | 1 | 0 | 2 | 0 |
| *Sturisoma rostratum* | 1 | ? | ? | 0 | ? | 0 | 0 | ? | ? | ? | 0 | 4 | 0 | 2 | 1 | 0 | 1 | 0 | 0 | 1 | 2 | 0 | 2 | 0 |
| *Sturisoma* aff. *tenuirostre* | 1 | 1 | 0 | 1 | 1 | 1 | 1 | 2 | 1 | 0 | 0 | 3 | 1 | 2 | 1 | 0 | 1 | 0 | 0 | 1 | 2 | 0 | 2 | 0 |
| *Sturisoma graffini* | 1 | 1 | 0 | 0 | 1 | 1 | 2 | 2 | 1 | 0 | 0 | 3 | 1 | 2 | 1 | 2 | 1 | 0 | 0 | 1 | 2 | 0 | 2 | 0 |
| *Sturisomatichthys aureus* | 0 | 1 | 0 | 0 | 0 | 0 | 1 | 2 | 1 | 0 | 0 | 4 | 1 | 1 | 0 | 0 | 1 | 1 | 1 | 0 | 2 | 0 | 0 | 0 |
| *Sturisomatichthys citurensis* | 0 | 1 | 0 | 0 | 1 | ? | 1 | 1 | 0 | 0 | 0 | 3 | 1 | 2 | 0 | 2 | 1 | 1 | ? | 1 | 1 | 0 | 1 | 0 |
| *Sturisomatichthys dariensis* | 0 | 1 | 0 | 0 | 0 | 0 | 1 | 2 | 0 | 0 | 0 | 3 | 1 | 2 | 0 | 2 | 1 | 0 | 0 | 1 | 2 | 0 | 2 | 0 |
| *Sturisomatichthys festivus* | 0 | 1 | 0 | 1 | 1 | 0 | 0 | 2 | 1 | 0 | 0 | 2 | 1 | 1 | 0 | 2 | 1 | 1 | 1 | 1 | 2 | 0 | 0 | 0 |
| *Sturisomatichthys frenatus* | 0 | 1 | 0 | 0 | 1 | 0 | 0 | ? | ? | 0 | 0 | 4 | 1 | 2 | 0 | 2 | 1 | 1 | 0 | 1 | 2 | 0 | 0 | 0 |
| *Sturisomatichthys kneri* | 0 | 1 | 0 | 1 | 0 | 0 | 1 | 2 | 1 | 0 | 1 | 3 | 1 | 2 | 0 | 0 | 1 | 1 | 0 | 1 | 3 | 0 | 0 | 0 |
| *Sturisomatichthys leightoni* | 0 | 1 | 0 | 1 | 0 | ? | 1 | 2 | 1 | 0 | 0 | 4 | 1 | 1 | 1 | 2 | 1 | 1 | 0 | 0 | 1 | 0 | 1 | 0 |
| *Sturisomatichthys panamensis* | 0 | 1 | 0 | 0 | 0 | 0 | 1 | 2 | 0 | 1 | 0 | 4 | 1 | 2 | 0 | 0 | 1 | 1 | 0 | 1 | 2 | 0 | 0 | 0 |
| *Sturisomatichthys tamanae* | 0 | 1 | 0 | 1 | 1 | ? | 1 | 1 | 0 | 0 | 0 | 4 | 1 | 2 | 0 | 2 | 1 | 0 | 0 | ? | 1 | 0 | 1 | 0 |
| *Sturisomatichthys varii* | 0 | 1 | 0 | 1 | 1 | 0 | 1 | 2 | 1 | 0 | 0 | 4 | 1 | 2 | 1 | 2 | 1 | 1 | 0 | 0 | 2 | 0 | 0 | 0 |
| *Sturisomatichthys reinae* | 0 | 1 | 0 | 1 | 0 | 0 | 2 | 2 | 1 | 0 | 0 | 4 | 1 | 2 | 0 | 0 | 1 | 0 | 1 | 1 | 2 | 0 | 2 | 0 |

| **Taxon/Character** | **51** | **52** | **53** | **54** | **55** | **56** | **57** | **58** | **59** | **60** | **61** | **62** | **63** | **64** | **65** | **66** | **67** | **68** | **69** | **70** | **71** | **72** | **73** | **74** |
|---|---|---|---|---|---|---|---|---|---|---|---|---|---|---|---|---|---|---|---|---|---|---|---|---|
| ***Lamontichthys parakana*** | 1 | 1 | 1 | 0 | 1 | 0 | 0 | 0 | 1 | 1 | 1 | 2 | 1 | 1 | 0 | 1 | 2 | 1 | 0 | 0 | 1 | 1 | 1 | 0 |
| ***Metaloricaria nijsseni*** | 2 | 2 | 2 | 1 | 0 | 0 | 1 | 1 | 2 | 2 | 1 | 0 | 0 | 1 | 1 | 1 | 3 | 0 | 0 | 0 | 1 | 1 | 2 | 1 |
| ***Metaloricaria paucidens*** | 2 | 2 | 2 | 1 | 0 | 0 | 1 | 1 | 2 | 2 | 1 | 0 | 0 | 1 | 1 | 1 | 3 | 0 | 0 | 0 | 1 | 1 | 2 | 1 |
| ***Pterosturisoma microps*** | 1 | 0 | 2 | 0 | 1 | 0 | 0 | 0 | 0 | 1 | 1 | 2 | 0 | 1 | 1 | 1 | 1 | 0 | 0 | 0 | 0 | 1 | 1 | 0 |
| ***Sturisoma barbatum*** | 0 | 1 | 0 | 0 | 1 | 0 | 0 | 0 | 2 | 2 | 1 | 2 | 0 | 0 | 1 | 1 | 0 | 0 | 1 | 1 | 1 | 0 | 1 | 1 |
| ***Sturisoma guentheri*** | 0 | 1 | 1 | 0 | 1 | 0 | 0 | 0 | 2 | 2 | 1 | 2 | 1 | 1 | 1 | 1 | 0 | 0 | 1 | 1 | 1 | 0 | 1 | 1 |
| ***Sturisoma lyra*** | 0 | 1 | 0 | 0 | 1 | 0 | 0 | 0 | 2 | 2 | 1 | 2 | 0 | 0 | 1 | 1 | 0 | 0 | 1 | 1 | 1 | 0 | 1 | 1 |
| ***Sturisoma monopelte*** | 0 | 1 | 0 | 0 | 1 | 0 | 0 | 0 | 2 | 2 | 1 | 2 | 1 | 0 | 1 | 1 | 0 | 0 | 1 | 0 | 1 | 0 | 1 | 1 |
| ***Sturisoma nigrirostrum*** | 0 | 1 | 0 | 0 | 1 | 0 | 0 | 0 | 2 | 1 | 1 | 2 | 1 | 0 | 1 | 1 | 0 | 0 | 1 | 0 | 1 | 1 | 1 | 1 |
| ***Sturisoma robustum*** | 1 | 1 | 0 | 0 | 1 | 0 | 0 | 0 | 2 | 2 | 1 | 1 | 1 | 1 | 0 | 1 | 0 | 0 | 1 | 0 | 0 | 0 | 1 | 1 |
| ***Sturisoma rostratum*** | 1 | 1 | 0 | 0 | 1 | 0 | 0 | 0 | 2 | 2 | 1 | 2 | 0 | 0 | 0 | 1 | 0 | 0 | 1 | ? | 0 | ? | 2 | 1 |
| ***Sturisoma* aff. *tenuirostre*** | 1 | 1 | 0 | 0 | 1 | 0 | 0 | 0 | 2 | 2 | 1 | 1 | 0 | 0 | 1 | 1 | 0 | 0 | 1 | 1 | 1 | 1 | 1 | 1 |
| ***Sturisoma graffini*** | 1 | 1 | 0 | 0 | 1 | 0 | 0 | 0 | 2 | 2 | 1 | 2 | 1 | 0 | 1 | 1 | 0 | 0 | 1 | 0 | 1 | 1 | 1 | 1 |
| ***Sturisomatichthys aureus*** | 1 | 0 | 1 | 0 | 1 | 0 | 0 | 0 | 2 | 2 | 1 | 2 | 1 | 1 | 1 | 1 | 0 | 1 | 0 | 0 | 0 | 1 | 0 | 1 |
| ***Sturisomatichthys citurensis*** | 0 | 1 | 1 | 0 | 1 | 0 | 0 | 0 | 1 | 1 | 1 | 2 | 1 | 1 | 0 | 1 | 0 | 0 | 0 | 1 | 0 | 0 | 1 | 1 |
| ***Sturisomatichthys dariensis*** | 0 | 2 | 1 | 0 | 1 | 0 | 0 | 0 | 2 | 1 | 1 | 2 | 1 | 1 | 1 | 1 | 0 | 0 | 0 | 0 | 0 | 1 | 0 | 1 |
| ***Sturisomatichthys festivus*** | 1 | 0 | 1 | 0 | 1 | 0 | 0 | 0 | 2 | 2 | 1 | 2 | 1 | 1 | 1 | 1 | 0 | 0 | 0 | 1 | 1 | 0 | 0 | 1 |
| ***Sturisomatichthys frenatus*** | 0 | 0 | 1 | 0 | 1 | 0 | 0 | 0 | 2 | 1 | 1 | 2 | 1 | 1 | 1 | 1 | 0 | 0 | 0 | 0 | 1 | 1 | 0 | 1 |
| ***Sturisomatichthys kneri*** | 0 | 0 | 1 | 0 | 1 | 0 | 0 | 0 | 2 | 2 | 1 | 2 | 1 | 1 | 1 | 1 | 0 | 0 | 0 | 1 | 1 | 1 | 0 | 1 |
| ***Sturisomatichthys leightoni*** | 0 | 0 | 1 | 0 | 1 | 0 | 0 | 0 | 2 | 1 | 1 | 2 | 0 | 1 | 1 | 1 | 0 | 1 | 0 | 0 | 0 | 1 | 1 | 1 |
| ***Sturisomatichthys panamensis*** | 1 | 2 | 1 | 0 | 1 | 0 | 0 | 0 | 2 | 2 | 1 | 2 | 1 | 1 | 1 | 1 | 0 | 0 | 0 | 0 | 1 | 1 | 1 | 1 |
| ***Sturisomatichthys tamanae*** | 0 | 0 | 1 | 0 | 1 | 0 | 0 | 0 | 2 | 1 | 1 | 2 | 1 | 1 | 0 | 1 | 0 | 0 | 0 | 1 | 0 | 1 | 1 | 1 |
| ***Sturisomatichthys varii*** | 1 | 0 | 1 | 0 | 1 | 0 | 0 | 0 | 2 | 1 | 1 | 2 | 1 | 0 | 1 | 1 | 0 | 0 | 0 | 0 | 0 | 0 | 0 | 1 |
| ***Sturisomatichthys reinae*** | 0 | 0 | 1 | 0 | 1 | 0 | 0 | 0 | 2 | 2 | 1 | 2 | 1 | 0 | 1 | 1 | 0 | 0 | 0 | 0 | 0 | 1 | 1 | 1 |

| **Taxon/Character** | **75** | **76** | **77** | **78** | **79** | **80** | **81** | **82** | **83** | **84** | **85** | **86** | **87** | **88** | **89** | **90** | **91** | **92** | **93** | **94** | **95** | **96** | **97** | **98** | **99** |
|---|---|---|---|---|---|---|---|---|---|---|---|---|---|---|---|---|---|---|---|---|---|---|---|---|---|
| ***Lamontichthys parakana*** | 0 | 1 | 0 | 1 | 0 | 1 | 0 | 1 | 2 | 1 | 0 | 3 | 1 | 2 | 1 | 0 | 0 | 0 | 1 | 0 | 2 | 0 | 0 | 0 | 0 |
| ***Metaloricaria nijsseni*** | 1 | 0 | 0 | 2 | 1 | 1 | 0 | ? | ? | 1 | 0 | 3 | 1 | 2 | ? | ? | ? | 2 | - | 0 | 0 | 0 | 0 | 0 | 1 |
| ***Metaloricaria paucidens*** | 1 | 0 | 0 | 2 | 1 | 1 | 0 | ? | ? | 1 | 0 | 3 | 1 | 2 | ? | ? | ? | 2 | - | 0 | 0 | 0 | 0 | 0 | 1 |
| ***Pterosturisoma microps*** | 0 | 1 | 0 | 1 | 0 | 1 | 0 | 2 | 1 | 1 | 0 | 3 | 1 | 2 | 1 | 0 | 0 | 2 | - | 2 | 2 | 0 | 3 | 0 | 1 |
| ***Sturisoma barbatum*** | 1 | 1 | 1 | 3 | 1 | 0 | 0 | 1 | 2 | 1 | 0 | 3 | 1 | 2 | 1 | 0 | 1 | 0 | 1 | 2 | 2 | 0 | 0 | 1 | - |
| ***Sturisoma guentheri*** | 1 | 1 | 2 | 3 | 1 | 0 | 0 | 1 | 2 | 1 | 0 | 3 | 1 | 2 | 1 | 1 | 0 | 0 | 1 | 2 | 2 | 0 | 0 | 1 | - |
| ***Sturisoma lyra*** | 1 | 1 | 2 | 3 | 1 | 0 | 0 | 1 | 2 | 1 | 0 | 3 | 1 | 2 | 1 | 0 | 1 | 0 | 1 | 2 | 2 | 0 | 0 | 1 | - |
| ***Sturisoma monopelte*** | 1 | 1 | 2 | 3 | 1 | 0 | 0 | 3 | 2 | 1 | 0 | 3 | 1 | 2 | 1 | 1 | 0 | 0 | 1 | 2 | 2 | 0 | 0 | 1 | - |
| ***Sturisoma nigrirostrum*** | 1 | 1 | 2 | 0 | 1 | 0 | 0 | 1 | 2 | 0 | 0 | 1 | 1 | 2 | 1 | 1 | 0 | 0 | 1 | 2 | 2 | 0 | 0 | 0 | 0 |
| ***Sturisoma robustum*** | 1 | 1 | 0 | 0 | 1 | 0 | 0 | 3 | 2 | 1 | 0 | 3 | 1 | 2 | 1 | 0 | 0 | 0 | 1 | 2 | 2 | 0 | 0 | 1 | - |
| ***Sturisoma rostratum*** | 1 | 1 | 0 | 3 | 1 | 0 | 0 | 1 | 2 | 1 | 0 | 3 | 1 | 2 | 1 | 0 | 1 | 0 | 1 | 2 | 2 | 0 | 0 | 1 | - |
| ***Sturisoma* aff. *tenuirostre*** | 1 | 1 | 2 | 0 | 1 | 0 | 0 | 0 | 2 | 1 | 0 | 3 | 1 | 2 | 1 | 0 | 0 | 0 | 1 | 2 | 2 | 0 | 0 | 1 | - |
| ***Sturisoma graffini*** | 1 | 1 | 1 | 3 | 1 | 0 | 0 | 0 | 2 | 1 | 0 | 3 | 1 | 2 | 1 | 0 | 0 | 0 | 1 | 2 | 2 | 0 | 0 | 1 | - |
| ***Sturisomatichthys aureus*** | 1 | 1 | 0 | 0 | 1 | 1 | 0 | 3 | 0 | 1 | 0 | 1 | 1 | 2 | 1 | 0 | 0 | 0 | 1 | 0 | 0 | 0 | 0 | 0 | 2 |
| ***Sturisomatichthys citurensis*** | 1 | 1 | ? | ? | 1 | 1 | 0 | 1 | 1 | 0 | 0 | 1 | 1 | 2 | 1 | 0 | 0 | 0 | 1 | 0 | 1 | 0 | 0 | 0 | 1 |
| ***Sturisomatichthys dariensis*** | 1 | 1 | 0 | 0 | 1 | 1 | 0 | 1 | 1 | 1 | 0 | 1 | 1 | 2 | 1 | 1 | 0 | 0 | 1 | 0 | 0 | 0 | 0 | 0 | 2 |
| ***Sturisomatichthys festivus*** | 1 | 1 | 0 | 0 | 1 | 1 | 0 | 1 | 1 | 1 | 0 | 1 | 1 | 2 | 1 | 0 | 0 | 0 | 1 | 0 | 0 | 0 | 0 | 0 | 1 |
| ***Sturisomatichthys frenatus*** | 1 | 1 | 0 | 0 | 1 | 1 | 0 | 1 | 1 | 1 | 0 | 1 | 1 | 2 | 1 | 0 | 0 | 0 | 1 | 0 | 0 | 0 | 0 | 0 | 2 |
| ***Sturisomatichthys kneri*** | 1 | 1 | 2 | 0 | 1 | 1 | 0 | 1 | 1 | 0 | 0 | 1 | 1 | 2 | 1 | 0 | 0 | 0 | 1 | 0 | 0 | 0 | 0 | 0 | 2 |
| ***Sturisomatichthys leightoni*** | 1 | 1 | ? | 0 | 1 | 1 | 0 | 1 | 1 | 0 | 0 | 1 | 1 | 2 | 1 | 0 | 0 | 0 | 1 | 0 | 0 | 0 | 0 | 0 | 1 |
| ***Sturisomatichthys panamensis*** | 1 | 1 | 1 | 0 | 1 | 1 | 0 | 1 | 1 | 1 | 0 | 1 | 1 | 0 | 1 | 0 | 0 | 0 | 1 | 0 | 0 | 0 | 0 | 0 | 2 |
| ***Sturisomatichthys tamanae*** | 1 | 1 | ? | 3 | 1 | 1 | 0 | 1 | 1 | 1 | 0 | 1 | 1 | 2 | 1 | 0 | 0 | 0 | 1 | 0 | 0 | 0 | 0 | 0 | 1 |
| ***Sturisomatichthys varii*** | 1 | 1 | 1 | 0 | 1 | 1 | 0 | 1 | 1 | 1 | 0 | 1 | 1 | 2 | 1 | 0 | 1 | 0 | 1 | 0 | 1 | 0 | 0 | 0 | 2 |
| ***Sturisomatichthys reinae*** | 1 | 1 | 1 | 0 | 1 | 1 | 0 | 1 | 1 | 1 | 0 | 1 | 1 | 2 | 1 | 0 | 0 | 0 | 1 | 0 | 0 | 0 | 0 | 0 | 2 |

| **Taxon/Character** | **100** | **101** | **102** | **103** | **104** | **105** | **106** | **107** | **108** | **109** | **110** | **111** | **112** | **113** | **114** | **115** | **116** | **117** | **118** | **119** | **120** | **121** | **122** | **123** |
|---|---|---|---|---|---|---|---|---|---|---|---|---|---|---|---|---|---|---|---|---|---|---|---|---|
| ***Lamontichthys parakana*** | 0 | 2 | 0 | 0 | 1 | 1 | 0 | 2 | 1 | 0 | 1 | 0 | 1 | 0 | 2 | 1 | 1 | 1 | 0 | 0 | 1 | 0 | 1 | 1 |
| ***Metaloricaria nijsseni*** | 1 | - | 0 | 0 | 3 | 3 | 0 | 0 | 0 | 0 | 0 | 0 | 0 | 2 | 0 | 1 | 0 | 1 | 0 | 0 | 1 | 1 | 0 | 0 |
| ***Metaloricaria paucidens*** | 1 | - | 0 | 0 | 3 | 3 | 0 | 0 | 0 | 0 | 0 | 0 | 0 | 2 | 0 | 1 | 0 | 1 | 0 | 0 | 1 | 1 | 0 | 0 |
| ***Pterosturisoma microps*** | 0 | 2 | 1 | 4 | 1 | 1 | 0 | 2 | 1 | 0 | 0 | 0 | 0 | 0 | 2 | 1 | 0 | 1 | 0 | 0 | 1 | 1 | 0 | 1 |
| ***Sturisoma barbatum*** | 0 | 2 | 0 | 3 | 1 | 3 | 0 | 3 | 1 | 0 | 0 | 0 | 0 | 0 | 2 | 1 | 0 | ? | 1 | 1 | 1 | 1 | 0 | 1 |
| ***Sturisoma guentheri*** | 0 | 2 | 0 | 3 | 1 | 3 | 0 | 3 | 1 | 0 | 0 | 0 | 0 | 0 | 2 | 1 | 0 | 1 | 1 | 1 | 1 | 1 | 0 | 1 |
| ***Sturisoma lyra*** | 0 | 2 | 0 | 3 | 1 | 3 | 0 | 3 | 1 | 0 | 0 | 0 | 0 | 0 | 2 | 0 | 0 | 1 | 1 | 1 | 1 | 1 | 1 | 0 |
| ***Sturisoma monopelte*** | 0 | 2 | 0 | 3 | 1 | 3 | 0 | 3 | 1 | 0 | 0 | 0 | 0 | 1 | 2 | 2 | 0 | 1 | 2 | 1 | 1 | 1 | 0 | 1 |
| ***Sturisoma nigrirostrum*** | 0 | 2 | 0 | 3 | 1 | 3 | 0 | 3 | 1 | 0 | 0 | 0 | 0 | 1 | 2 | 1 | 0 | 1 | 2 | 1 | 1 | 1 | 0 | 0 |
| ***Sturisoma robustum*** | 0 | 0 | 0 | 3 | 1 | 3 | 0 | 3 | 1 | 0 | 0 | 0 | 0 | 0 | 2 | 1 | 0 | 1 | 2 | 1 | 1 | 0 | 0 | 0 |
| ***Sturisoma rostratum*** | 0 | 2 | 0 | 3 | 1 | 3 | 0 | 3 | 1 | 0 | ? | ? | ? | 0 | 2 | 1 | 0 | 1 | 1 | 1 | 1 | 1 | 0 | 1 |
| ***Sturisoma* aff. *tenuirostre*** | 0 | 0 | 0 | 3 | 1 | 3 | 0 | 3 | 1 | 0 | 0 | 0 | 0 | 0 | 2 | 1 | 0 | 1 | 2 | 0 | 1 | 1 | 0 | 1 |
| ***Sturisoma graffini*** | 1 | - | 0 | 3 | 1 | 3 | 0 | 3 | 1 | 0 | 0 | 0 | 0 | 0 | 2 | 1 | 0 | 1 | 2 | 0 | 1 | 1 | 0 | 1 |
| ***Sturisomatichthys aureus*** | 1 | - | 0 | 0 | 1 | 1 | 0 | 2 | 0 | 0 | 0 | 1 | 0 | 0 | 2 | 1 | 0 | 1 | 0 | 0 | 1 | 2 | 3 | 0 |
| ***Sturisomatichthys citurensis*** | 1 | - | 0 | 0 | 1 | 1 | 0 | 2 | 0 | 0 | 0 | 1 | 0 | 0 | 2 | 1 | 1 | 1 | 2 | 0 | 1 | 2 | 1 | 0 |
| ***Sturisomatichthys dariensis*** | 1 | - | 0 | 0 | 1 | 1 | 0 | 2 | 0 | 0 | 0 | 1 | 0 | 0 | 2 | 1 | 0 | 1 | 3 | 0 | 1 | 2 | 0 | 0 |
| ***Sturisomatichthys festivus*** | 1 | - | 0 | 0 | 1 | 1 | 0 | 2 | 0 | 0 | 0 | 1 | 0 | 0 | 2 | 1 | 0 | 1 | 0 | 0 | 1 | 2 | 2 | 0 |
| ***Sturisomatichthys frenatus*** | 1 | - | 0 | 0 | 1 | 1 | 0 | 2 | 0 | 0 | 0 | 1 | 0 | 0 | 2 | 1 | 0 | 1 | 2 | 0 | 1 | 2 | 1 | 0 |
| ***Sturisomatichthys kneri*** | 1 | - | 0 | 0 | 1 | 1 | 0 | 2 | 0 | 0 | 0 | 1 | 0 | 0 | 2 | 2 | 0 | 1 | 0 | 0 | 1 | 2 | 1 | 0 |
| ***Sturisomatichthys leightoni*** | 1 | - | 0 | 0 | 1 | 1 | 0 | 2 | 0 | 0 | 0 | 1 | 0 | 0 | 2 | 2 | 0 | 1 | 2 | 0 | 1 | 2 | 3 | 0 |
| ***Sturisomatichthys panamensis*** | 1 | - | 0 | 0 | 1 | 1 | 0 | 2 | 0 | 0 | 0 | 1 | 1 | 0 | 2 | 1 | 0 | ? | 0 | 0 | 1 | 2 | 3 | 0 |
| ***Sturisomatichthys tamanae*** | 1 | - | 0 | 0 | 1 | 1 | 0 | 2 | 0 | 0 | 0 | 1 | 0 | 0 | 2 | 1 | 0 | 1 | 0 | 0 | 1 | 2 | 3 | 0 |
| ***Sturisomatichthys varii*** | 1 | - | 0 | 0 | 1 | 1 | 0 | 2 | 0 | 0 | 0 | 1 | 0 | 0 | 2 | 1 | 0 | 1 | 2 | 0 | 1 | 2 | 1 | 0 |
| ***Sturisomatichthys reinae*** | 1 | - | 0 | 0 | 1 | 1 | 0 | 2 | 0 | 0 | 0 | 1 | 0 | 0 | 2 | 1 | 0 | 1 | 0 | 0 | 1 | 2 | 0 | 0 |

| Taxon/Character | 124 | 125 | 126 | 127 | 128 | 129 | 130 | 131 | 132 | 133 | 134 | 135 | 136 | 137 | 138 | 139 | 140 | 141 | 142 | 143 | 144 | 145 | 146 | 147 |
|---|---|---|---|---|---|---|---|---|---|---|---|---|---|---|---|---|---|---|---|---|---|---|---|---|
| ***Lamontichthys parakana*** | 1 | 1 | 0 | 0 | 3 | 0 | 2 | 1 | 0 | 1 | 1 | 1 | 1 | 1 | 0 | 0 | 0 | 0 | 1 | 1 | 1 | 1 | 1 | 0 |
| ***Metaloricaria nijsseni*** | 1 | 0 | 0 | 0 | 3 | 0 | 2 | 1 | 0 | 0 | 1 | 2 | 1 | 0 | 0 | 1 | 1 | 0 | 2 | 2 | 0 | 1 | 0 | 1 |
| ***Metaloricaria paucidens*** | 1 | 0 | 0 | 0 | 3 | 0 | 2 | 1 | 0 | 0 | 1 | 2 | 1 | 0 | 0 | 1 | 1 | 0 | 2 | 2 | 0 | 1 | 0 | 1 |
| ***Pterosturisoma microps*** | 1 | 0 | 0 | 0 | 2 | 0 | 1 | 1 | 0 | 1 | 1 | 1 | 1 | 0 | 1 | 0 | 1 | 0 | 1 | 1 | 1 | 1 | 1 | 0 |
| ***Sturisoma barbatum*** | 1 | 3 | 0 | 0 | 3 | 0 | 2 | 1 | 0 | 1 | 1 | 1 | 1 | 0 | 0 | 0 | 1 | 0 | 2 | 1 | 1 | 1 | 1 | 0 |
| ***Sturisoma guentheri*** | 1 | 3 | 1 | 1 | 3 | 0 | 1 | 1 | 0 | 1 | 1 | 1 | 1 | 0 | 0 | 0 | 1 | 0 | 2 | 1 | 1 | 1 | 1 | 0 |
| ***Sturisoma lyra*** | 1 | 3 | 0 | 0 | 3 | 0 | 2 | 1 | 0 | 1 | 1 | 1 | 1 | 0 | 0 | 0 | 1 | 0 | 2 | 1 | 1 | 1 | 0 | 0 |
| ***Sturisoma monopelte*** | 1 | 3 | 0 | 0 | 2 | 0 | 1 | 1 | 0 | 1 | 1 | 1 | 1 | 0 | 0 | 0 | 0 | 0 | 2 | 1 | 1 | 1 | 1 | 0 |
| ***Sturisoma nigrirostrum*** | 1 | 3 | 0 | 0 | 3 | 0 | 0 | 1 | 0 | 1 | 1 | 1 | 1 | 0 | 0 | 0 | 0 | 0 | 2 | 1 | 1 | 1 | 1 | 0 |
| ***Sturisoma robustum*** | 1 | 3 | 0 | 0 | 2 | 0 | 1 | 1 | 0 | 1 | 1 | 1 | 1 | 0 | 0 | 0 | 1 | 0 | 2 | 1 | 1 | 1 | 1 | 0 |
| ***Sturisoma rostratum*** | 1 | 3 | 0 | 0 | 3 | 0 | 2 | 1 | 0 | 1 | 1 | 1 | 1 | 0 | 0 | 0 | 1 | 0 | 2 | 1 | 1 | 1 | 1 | 0 |
| ***Sturisoma* aff. *tenuirostre*** | 1 | 3 | 0 | 0 | 2 | 0 | 1 | 1 | 0 | 1 | 1 | 1 | 1 | 0 | 0 | 0 | 0 | 0 | 2 | 1 | 1 | 1 | 1 | 0 |
| ***Sturisoma graffini*** | 1 | 3 | 0 | 0 | 2 | 0 | 1 | 1 | 0 | 1 | 1 | 1 | 1 | 0 | 0 | 0 | 0 | 0 | 2 | 1 | 1 | 1 | 1 | 0 |
| ***Sturisomatichthys aureus*** | 1 | 3 | 0 | 0 | 3 | 0 | 2 | 1 | 0 | 0 | 1 | 1 | 1 | 0 | 0 | 0 | 0 | 0 | 0 | 1 | 1 | 1 | 1 | 0 |
| ***Sturisomatichthys citurensis*** | 1 | 0 | 1 | 1 | 3 | 0 | 1 | 1 | 0 | 0 | 1 | 1 | 1 | 0 | 0 | 0 | 1 | 0 | 0 | 1 | 1 | 1 | 0 | 0 |
| ***Sturisomatichthys dariensis*** | 1 | 3 | 0 | 0 | 3 | 0 | 2 | 1 | 0 | 0 | 1 | 1 | 1 | 0 | 0 | 0 | 1 | 0 | 0 | 1 | 1 | 1 | 0 | 0 |
| ***Sturisomatichthys festivus*** | 1 | 2 | 0 | 0 | 3 | 0 | 1 | 1 | 0 | 1 | 1 | 1 | 1 | 0 | 0 | 0 | 0 | 0 | 0 | 1 | 1 | 1 | 1 | 0 |
| ***Sturisomatichthys frenatus*** | 1 | 3 | 1 | 1 | 2 | 0 | 1 | 1 | 0 | 0 | 1 | 1 | 1 | 0 | 0 | 0 | 0 | 0 | 0 | 1 | 1 | 1 | 1 | 0 |
| ***Sturisomatichthys kneri*** | 1 | 3 | 1 | 1 | 3 | 0 | 1 | 1 | 0 | 0 | 1 | 1 | 1 | 0 | 0 | 0 | 0 | 0 | 0 | 1 | 1 | 1 | 1 | 0 |
| ***Sturisomatichthys leightoni*** | 1 | 3 | 0 | 0 | 2 | 0 | 2 | 1 | 0 | 0 | 1 | 1 | 1 | 0 | 0 | 0 | 1 | 0 | 0 | 0 | 1 | 1 | 0 | 0 |
| ***Sturisomatichthys panamensis*** | 1 | 3 | 0 | 0 | 2 | 0 | 2 | 1 | 0 | 0 | 1 | 1 | 1 | 0 | 0 | 0 | 0 | 0 | 0 | 1 | 1 | 1 | 0 | 0 |
| ***Sturisomatichthys tamanae*** | 1 | 0 | 0 | 0 | 3 | 0 | 1 | 1 | 0 | 0 | 1 | 1 | 1 | 0 | 0 | 0 | 0 | 0 | 0 | 1 | 1 | 1 | 0 | 0 |
| ***Sturisomatichthys varii*** | 1 | 3 | 0 | 0 | 2 | 0 | 1 | 1 | 0 | 0 | 1 | 1 | 1 | 0 | 0 | 0 | 1 | 0 | 0 | 1 | 1 | 1 | 1 | 0 |
| ***Sturisomatichthys reinae*** | 1 | 3 | 0 | 0 | 1 | 0 | 1 | 1 | 0 | 0 | 1 | 1 | 1 | 0 | 0 | 0 | 0 | 0 | 0 | 1 | 1 | 1 | 1 | 0 |

| **Taxon/Character** | **148** | **149** | **150** | **151** | **152** | **153** | **154** | **155** | **156** | **157** | **158** | **159** | **160** | **161** | **162** | **163** | **164** | **165** | **166** | **167** | **168** | **169** | **170** | **171** |
|---|---|---|---|---|---|---|---|---|---|---|---|---|---|---|---|---|---|---|---|---|---|---|---|---|
| ***Lamontichthys parakana*** | 2 | 0 | 2 | 0 | 0 | 0 | 2 | 0 | 0 | 0 | 1 | 1 | 2 | 1 | 1 | 2 | 1 | 0 | 0 | 1 | 0 | 0 | 1 | 1 |
| ***Metaloricaria nijsseni*** | 0 | 0 | 3 | 0 | 0 | 0 | 1 | 0 | 0 | 0 | 1 | 0 | 2 | 0 | 1 | 1 | 2 | 1 | 2 | 1 | 1 | 2 | 0 | 1 |
| ***Metaloricaria paucidens*** | 0 | 0 | 3 | 0 | 0 | 0 | 1 | 0 | 0 | 0 | 1 | 0 | 2 | 0 | 1 | 1 | 2 | 1 | 2 | 1 | 1 | 1 | 0 | 1 |
| ***Pterosturisoma microps*** | 2 | 0 | 1 | 0 | 0 | 0 | 2 | 3 | 0 | 2 | 1 | 1 | 2 | 0 | 0 | 2 | 2 | 0 | 2 | 0 | 1 | 2 | 0 | 1 |
| ***Sturisoma barbatum*** | 0 | 0 | 2 | 0 | 0 | 0 | 1 | 1 | 0 | 2 | 1 | 0 | 2 | 2 | 0 | 2 | 2 | 1 | 2 | 1 | 2 | 1 | 0 | 1 |
| ***Sturisoma guentheri*** | 0 | 0 | 2 | 0 | 0 | 0 | 2 | 1 | 0 | 2 | 1 | 0 | 2 | 1 | 0 | 2 | 2 | 1 | 2 | 1 | 2 | 1 | 0 | 1 |
| ***Sturisoma lyra*** | 0 | 0 | 2 | 0 | 0 | 0 | 2 | 0 | 0 | 2 | 1 | 0 | 2 | 1 | 0 | 2 | 2 | 1 | 2 | 1 | 2 | 1 | 1 | 1 |
| ***Sturisoma monopelte*** | 0 | 0 | 2 | 0 | 0 | 0 | 2 | 0 | 0 | 2 | 1 | 0 | 2 | 1 | 0 | 2 | 2 | 1 | 2 | 1 | 2 | 1 | 0 | 1 |
| ***Sturisoma nigrirostrum*** | 0 | 0 | 2 | 0 | 0 | 0 | 2 | 0 | 0 | 2 | 1 | 1 | 2 | 1 | 1 | 2 | 2 | 1 | 2 | 1 | 2 | 1 | 0 | 1 |
| ***Sturisoma robustum*** | 0 | 0 | 2 | 0 | 0 | 0 | 2 | 1 | 0 | 1 | 1 | 0 | 2 | 0 | 0 | 2 | 2 | 1 | 2 | 1 | 2 | 1 | 0 | 1 |
| ***Sturisoma rostratum*** | 0 | 0 | 2 | 0 | 0 | 0 | 2 | 1 | 0 | 2 | 1 | 0 | 2 | 1 | 1 | 2 | 2 | 1 | 2 | 1 | 2 | 1 | 0 | 1 |
| ***Sturisoma* aff. *tenuirostre*** | 0 | 0 | 2 | 0 | 0 | 0 | 2 | 0 | 0 | 2 | 1 | 1 | 2 | 1 | 0 | 2 | 2 | 1 | 2 | 1 | 2 | 1 | 0 | 1 |
| ***Sturisoma graffini*** | 0 | 0 | 2 | 0 | 0 | 0 | 2 | 0 | 0 | 2 | 1 | 1 | 2 | 2 | 0 | 2 | 2 | 1 | 2 | 1 | 2 | 1 | 0 | 1 |
| ***Sturisomatichthys aureus*** | 2 | 0 | 0 | 0 | 0 | 0 | 2 | 0 | 0 | 1 | 1 | 2 | 2 | 1 | 1 | 2 | 2 | 1 | 1 | 0 | 2 | 1 | 0 | 1 |
| ***Sturisomatichthys citurensis*** | 2 | 0 | 1 | 0 | 1 | 0 | 2 | 1 | 0 | 2 | 1 | 1 | 2 | 1 | 1 | 2 | 2 | 1 | 1 | 0 | 1 | 1 | 0 | 1 |
| ***Sturisomatichthys dariensis*** | 2 | 0 | 2 | 0 | 0 | 0 | 2 | 1 | 0 | 1 | 1 | 1 | 2 | 0 | 1 | 2 | 0 | 1 | 1 | 0 | 2 | 1 | 0 | 1 |
| ***Sturisomatichthys festivus*** | 2 | 2 | 1 | 0 | 0 | 0 | 2 | 0 | 0 | 2 | 1 | 1 | 2 | 1 | 1 | 2 | 0 | 1 | 1 | 0 | 2 | 1 | 0 | 1 |
| ***Sturisomatichthys frenatus*** | 2 | 0 | 2 | 0 | 0 | 0 | 2 | 0 | 0 | 1 | 1 | 1 | 2 | 1 | 1 | 2 | 0 | 1 | 1 | 0 | 2 | 1 | 0 | 1 |
| ***Sturisomatichthys kneri*** | 2 | 0 | 2 | 0 | 0 | 0 | 2 | 0 | 0 | 2 | 1 | 1 | 2 | 1 | 1 | 2 | 2 | 1 | 1 | 0 | 2 | 1 | ? | 1 |
| ***Sturisomatichthys leightoni*** | 2 | 2 | 1 | 0 | 0 | 0 | 2 | 0 | 0 | 1 | 1 | 2 | 2 | 1 | 1 | 2 | 2 | 1 | 1 | 0 | 2 | 2 | 0 | 1 |
| ***Sturisomatichthys panamensis*** | 2 | 0 | 2 | 0 | 0 | 0 | 2 | 1 | 0 | 2 | 1 | 2 | 2 | 1 | 0 | 2 | 2 | 1 | 1 | 0 | 2 | 1 | 0 | 1 |
| ***Sturisomatichthys tamanae*** | 2 | 0 | 1 | 0 | 0 | 0 | 2 | 0 | 0 | 1 | 1 | 1 | 2 | 1 | 1 | 2 | 2 | 1 | 1 | 0 | 1 | 1 | 0 | 1 |
| ***Sturisomatichthys varii*** | 2 | 0 | 1 | 0 | 0 | 0 | 2 | 1 | 0 | 1 | 1 | 0 | 2 | 1 | 1 | 2 | 2 | 1 | 1 | 0 | 2 | 1 | 0 | 1 |
| ***Sturisomatichthys reinae*** | 2 | 0 | 1 | 0 | 0 | 0 | 2 | 1 | 0 | 1 | 1 | 0 | 2 | 1 | 1 | 2 | 2 | 1 | 1 | 1 | 2 | 1 | 0 | 1 |

| **Taxon/Character** | **172** | **173** | **174** | **175** | **176** | **177** | **178** | **179** | **180** | **181** | **182** | **183** | **184** | **185** | **186** | **187** | **188** | **189** | **190** | **191** | **192** | **193** | **194** | **195** | **196** |
|---|---|---|---|---|---|---|---|---|---|---|---|---|---|---|---|---|---|---|---|---|---|---|---|---|---|
| ***Lamontichthys parakana*** | 0 | 0 | 1 | 1 | 3 | 1 | 1 | 1 | 1 | 0 | 2 | 0 | 0 | 1 | 0 | 1 | 1 | 0 | 1 | 1 | 1 | 1 | 0 | 2 | 1 |
| ***Metaloricaria nijsseni*** | 1 | 0 | 1 | 0 | 0 | 0 | 1 | 0 | 2 | 0 | 1 | 0 | 0 | 0 | 0 | 1 | 1 | 0 | 1 | 1 | 0 | 0 | 0 | 1 | 0 |
| ***Metaloricaria paucidens*** | 0 | 0 | 1 | 0 | 0 | 0 | 1 | 0 | 2 | 0 | 1 | 0 | 0 | 0 | 0 | 1 | 1 | 0 | 1 | 1 | 0 | 0 | 0 | 0 | 0 |
| ***Pterosturisoma microps*** | 0 | 0 | 1 | ? | 0 | 2 | 1 | 1 | 2 | 0 | 2 | 0 | 0 | 2 | 1 | 1 | 1 | 0 | 1 | 1 | 2 | 3 | 1 | 2 | 0 |
| ***Sturisoma barbatum*** | 1 | 1 | 1 | 2 | 3 | 0 | 1 | 1 | 3 | 1 | 2 | 0 | 0 | 1 | 0 | 1 | 1 | 0 | 1 | 1 | 0 | 2 | 0 | 1 | 1 |
| ***Sturisoma guentheri*** | 1 | 1 | 1 | 2 | 3 | 0 | 1 | 1 | 3 | 1 | 2 | 0 | 0 | 1 | 0 | 1 | 1 | 0 | 1 | 1 | 1 | 2 | 0 | 2 | 1 |
| ***Sturisoma lyra*** | 1 | 1 | 1 | 2 | 3 | 0 | 1 | 1 | 3 | 1 | 2 | 0 | 0 | 1 | 0 | 1 | 1 | 0 | 1 | 1 | 0 | 2 | 0 | 1 | 1 |
| ***Sturisoma monopelte*** | 1 | 1 | 1 | 2 | 3 | 0 | 1 | 1 | 3 | 1 | 2 | 0 | 0 | 1 | 0 | 1 | 1 | 0 | 1 | 1 | 1 | 2 | 0 | 0 | 1 |
| ***Sturisoma nigrirostrum*** | 1 | 1 | 1 | 2 | 3 | 0 | 1 | 1 | 3 | 1 | 2 | 0 | 0 | 1 | 0 | 1 | 1 | 0 | 1 | 1 | 0 | 2 | 0 | 0 | 1 |
| ***Sturisoma robustum*** | 0 | 1 | 1 | 2 | 3 | 0 | 1 | 1 | 3 | 1 | 2 | 1 | 0 | 1 | 0 | 1 | 1 | 0 | 1 | 1 | 1 | 2 | 0 | 1 | 1 |
| ***Sturisoma rostratum*** | 1 | 1 | 1 | 2 | 3 | 0 | 1 | 1 | 3 | 1 | 2 | 0 | 0 | 1 | 0 | 1 | 1 | 0 | 1 | 1 | 0 | 2 | 0 | 1 | 1 |
| ***Sturisoma* aff. *tenuirostre*** | 1 | 1 | 1 | 2 | 3 | 0 | 1 | 1 | 3 | 1 | 2 | 0 | 0 | 1 | 0 | 1 | 1 | 0 | 1 | 1 | 1 | 2 | 0 | 0 | 1 |
| ***Sturisoma graffini*** | 1 | 1 | 1 | 2 | 3 | 0 | 1 | 1 | 3 | 1 | 2 | 0 | 0 | 1 | 0 | 1 | 1 | 0 | 1 | 1 | 1 | 2 | 0 | 1 | 1 |
| ***Sturisomatichthys aureus*** | 1 | 1 | 1 | 2 | 3 | 1 | 1 | 1 | 3 | 0 | 2 | 0 | 0 | 1 | 1 | 1 | 1 | 0 | 1 | 1 | 0 | 2 | 1 | 2 | 1 |
| ***Sturisomatichthys citurensis*** | 0 | 0 | 1 | 2 | 3 | 1 | 1 | 1 | 3 | 0 | 2 | 0 | 0 | 1 | 1 | 1 | 1 | 0 | 1 | 1 | 0 | 2 | 1 | 2 | 1 |
| ***Sturisomatichthys dariensis*** | 1 | 1 | 1 | 2 | 2 | 1 | 1 | 1 | 3 | 0 | 2 | 0 | 0 | 2 | 1 | 1 | 1 | 0 | 1 | 1 | 0 | 2 | 1 | 3 | 1 |
| ***Sturisomatichthys festivus*** | 1 | 1 | 1 | 2 | 0 | 1 | 1 | 1 | 3 | 0 | 2 | 1 | 0 | 2 | 1 | 1 | 1 | 0 | 1 | 1 | 1 | 2 | 1 | 2 | 1 |
| ***Sturisomatichthys frenatus*** | 0 | 1 | 1 | 2 | 2 | 1 | 1 | 1 | 3 | 0 | 2 | 0 | 0 | 2 | 1 | 1 | 1 | 0 | 1 | 1 | 0 | 2 | 1 | 3 | 1 |
| ***Sturisomatichthys kneri*** | ? | ? | 1 | 2 | 3 | 1 | 1 | 1 | 3 | 0 | 2 | 0 | 0 | 1 | 1 | 1 | 1 | 0 | 1 | 1 | 1 | 2 | 1 | 0 | 1 |
| ***Sturisomatichthys leightoni*** | 0 | 1 | 1 | 2 | 3 | 1 | 1 | 1 | 3 | 0 | 2 | 0 | 0 | 1 | 1 | 1 | 1 | 0 | 1 | 1 | 0 | 2 | 1 | 2 | 1 |
| ***Sturisomatichthys panamensis*** | 0 | 1 | 1 | 2 | 0 | 1 | 1 | 1 | 3 | 0 | 2 | 0 | 0 | 1 | 1 | 1 | 1 | 0 | 1 | 1 | 0 | 2 | 1 | 2 | 1 |
| ***Sturisomatichthys tamanae*** | 0 | 0 | 1 | 2 | 3 | 1 | 1 | 1 | 3 | 0 | 2 | 0 | 0 | 1 | 1 | 1 | 1 | 0 | 1 | 1 | 0 | 2 | 1 | 2 | 1 |
| ***Sturisomatichthys varii*** | 0 | 1 | 1 | 2 | 3 | 1 | 1 | 1 | 3 | 0 | 2 | 0 | 0 | 1 | 1 | 1 | 1 | 0 | 1 | 1 | 0 | 2 | 1 | 3 | 1 |
| ***Sturisomatichthys reinae*** | 0 | 1 | 1 | 2 | 3 | 1 | 1 | 1 | 3 | 0 | 2 | 0 | 0 | 2 | 1 | 1 | 1 | 0 | 1 | 1 | 1 | 2 | 1 | 3 | 1 |
