# Supplementary figures and images for: A combined molecular and morphological phylogeny of the Loricariinae (Siluriformes: Loricariidae), with emphasis on the Harttiini and Farlowellini
Source: PLoS One. 2021 Mar 15;16(3):e0247747. doi: 10.1371/journal.pone.0247747 (PMC7959404; doi:10.1371/journal.pone.0247747)

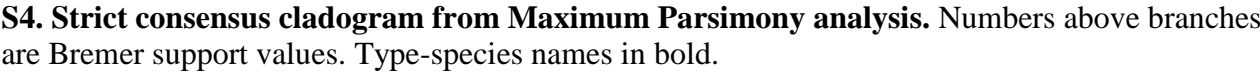

Supplement: S1 Fig — Numbers above branches are Bremer support values. Type-species names in bold. (PDF) [file pone.0247747.s004.pdf]

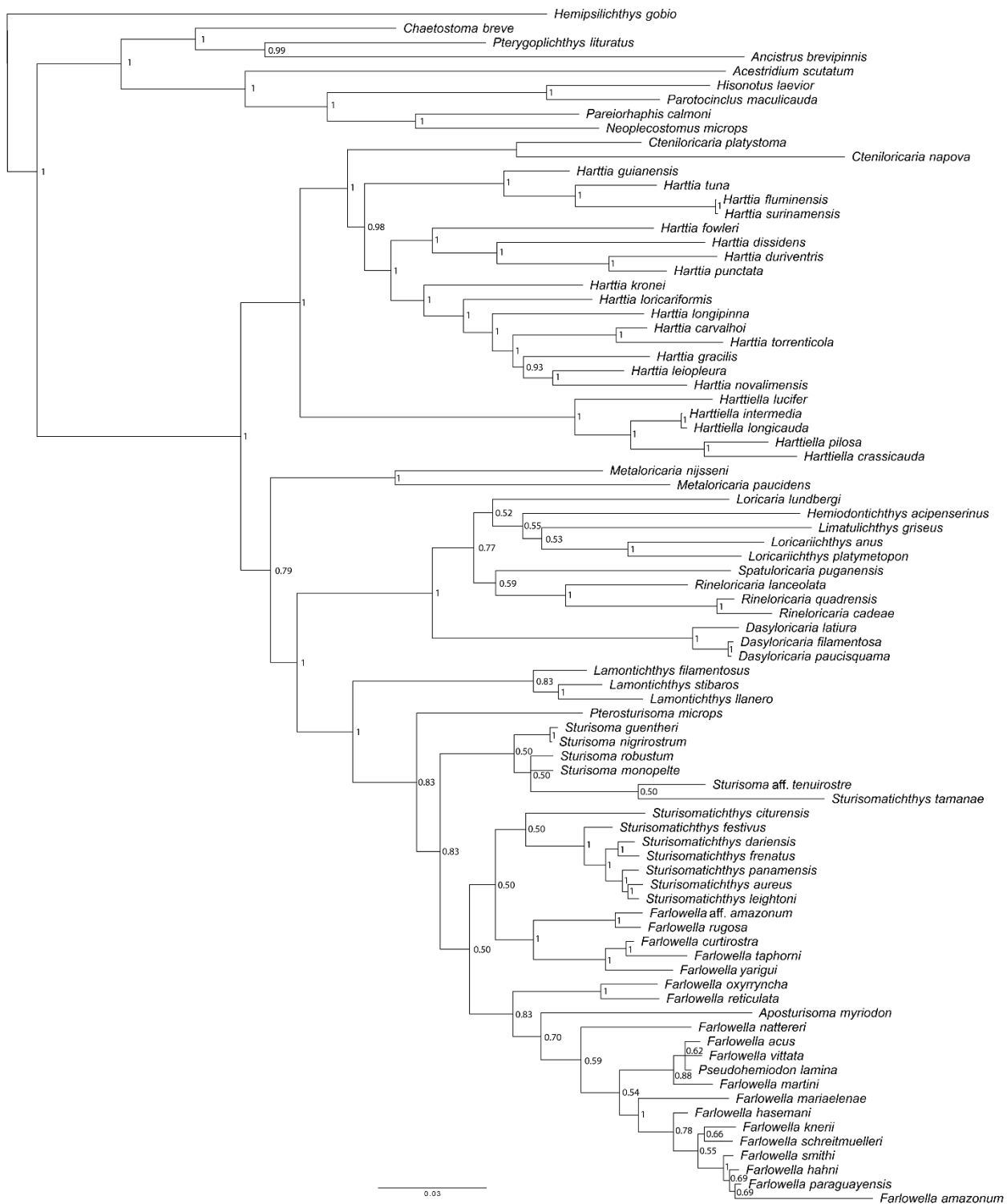

**S6. DNA-only Bayesian Tree.** Numbers at branches are Posterior Probabilities.

Supplement: S3 Fig — Numbers at branches are Posterior Probabilities. (PDF) [file pone.0247747.s006.pdf]
