# Supplementary material for: A combined molecular and morphological phylogeny of the Loricariinae (Siluriformes: Loricariidae), with emphasis on the Harttiini and Farlowellini
Source: PLoS One. 2021 Mar 15;16(3):e0247747. doi: 10.1371/journal.pone.0247747 (PMC7959404; doi:10.1371/journal.pone.0247747)
Supplement: S2 Fig — Numbers at branches are bootstrap frequencies. (PDF) [file pone.0247747.s005.pdf]

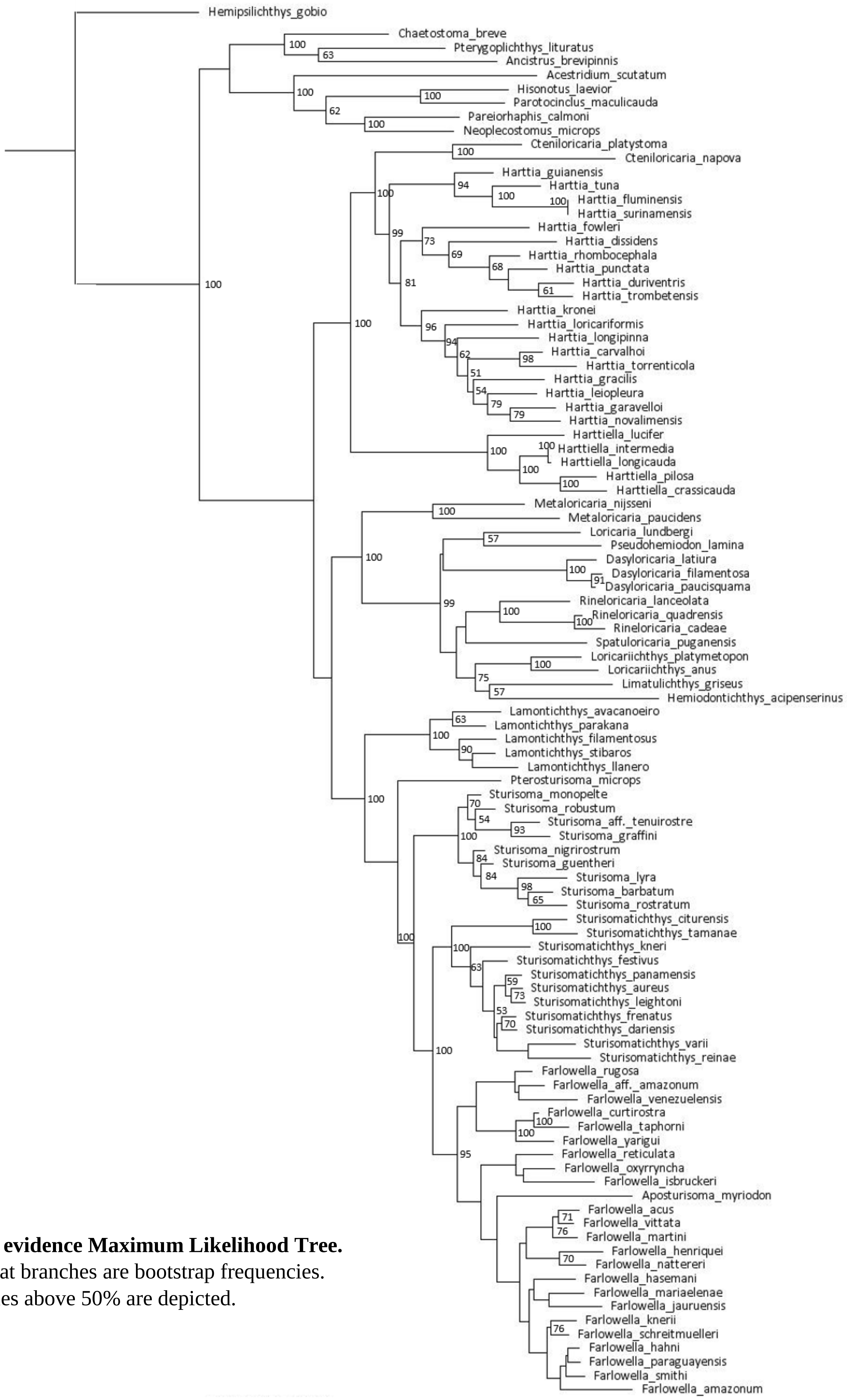

S5. Total evidence Maximum Likelihood Tree.
Numbers at branches are bootstrap frequencies.
Only values above 50% are depicted.
